# PET and SPECT Tracer Development via Copper-Mediated Radiohalogenation of Divergent and Stable Aryl-Boronic Esters

**DOI:** 10.3390/pharmaceutics17070837

**Published:** 2025-06-26

**Authors:** Austin Craig, Frederik J. Sachse, Markus Laube, Florian Brandt, Klaus Kopka, Sven Stadlbauer

**Affiliations:** 1 Institute of Radiopharmaceutical Cancer Research, Helmholtz-Zentrum Dresden-Rossendorf, Bautzner Landstraße 400, D-01328 Dresden, Germanyk.kopka@hzdr.de (K.K.); 2 Faculty of Chemistry and Food Chemistry, School of Science, Technische Universität Dresden, D-01062 Dresden, Germany; 3 Universitätsklinikum Carl Gustav Carus Dresden, Fetscherstraße 74, D-01307 Dresden, Germany

**Keywords:** copper-mediated radiohalogenation (CMRH), ^18^F-fluorination, ^123^I-iodination, positron emission tomography (PET), radiohalogenation, single-photon emission computed tomography (SPECT), prosthetic group

## Abstract

**Background/Objectives**: Positron emission tomography (PET) and single-photon emission computed tomography (SPECT) are highly sensitive clinical imaging modalities, frequently employed in conjunction with magnetic resonance imaging (MRI) or computed tomography (CT) for diagnosing a wide range of disorders. Efficient and robust radiolabeling methods are needed to accommodate the increasing demand for PET and SPECT tracer development. Copper-mediated radiohalogenation (CMRH) reactions enable rapid late-stage preparation of radiolabeled arenes, yet synthetic challenges and radiolabeling precursors’ instability can limit the applications of CMRH approaches. **Methods**: A series of aryl-boronic acids were converted into their corresponding aryl-boronic acid 1,1,2,2-tetraethylethylene glycol esters [ArB(Epin)s] and aryl-boronic acid 1,1,2,2-tetrapropylethylene glycol esters [ArB(Ppin)s] as stable and versatile precursor building blocks for radiolabeling via CMRH. General protocols for the preparation of ^18^F-labeled and ^123^I-labeled arenes utilizing CMRH of these substrates were developed and applied. The radiochemical conversions (RCC) were determined by radio-(U)HPLC. **Results**: Both ArB(Epin)s and ArB(Ppin)s-based radiolabeling precursors were prepared in a one-step synthesis with chemical yields of 49–99%. Radiolabeling of the aryl-boronic esters with fluorine-18 or iodine-123 via CMRH furnished the corresponding radiolabeled arenes with RCC of 7–99% and 10–99%, respectively. Notably, a radiohalogenated prosthetic group containing a vinyl sulfone motif was obtained with an activity yield (AY) of 18 ± 3%, and applied towards the preparation of two clinically relevant PET tracers. **Conclusions**: This approach enables the synthesis of stable radiolabeling precursors and thus provides increased versatility in the application of CMRH, thereby supporting the development of novel PET and SPECT radiotracers.

## 1. Introduction

Positron emission tomography (PET) and single-photon emission computed tomography (SPECT) are diagnostic non-invasive imaging modalities typically combined with magnetic resonance imaging (MRI) or computed tomography (CT) imaging modalities in nuclear medicine for the real-time hybrid visualization of metabolic disorders and malignancies with high precision [1,2,3,4,5,6]. In addition to radiometal-based radiopharmaceuticals, numerous PET and SPECT tracers are radiohalogenated compounds. Radiohalogenated molecules are also routinely used to obtain biodistribution and pharmacokinetic information in drug discovery and development [7,8,9,10].

Fluorine-18, the most predominant diagnostic radionuclide for PET imaging, has a suitable half-life (*t*_1/2_ = 109.7 min) for multi-step radiosynthesis and PET tracer transport between institutions [11]. Additionally, the high positron decay ratio (>97% β^+^) and low positron energy of fluorine-18 (0.635 MeV) afford high-resolution PET images. As of June 2025, out of 19 FDA-approved PET radiopharmaceuticals, 12 contain fluorine-18 [12,13]. The most clinically prevalent PET tracer for oncological applications, 2-deoxy-2-[^18^F]fluoro-d-glucose, or commonly named [^18^F]fluorodeoxyglucose ([^18^F]FDG), is a glucose analog where fluorine-18 replaces a hydroxyl substituent in the two-position as a bioisostere [14].

In contrast to fluorine-18, which functions solely as a positron-emitting radionuclide, compounds containing radioiodine offer more functional versatility, and can be used as PET or SPECT tracers, and for therapeutic applications. Radioiodinated compounds, irrespective of the radionuclide (e.g., iodine-123, iodine-124, or iodine-131) employed, are chemically indistinguishable and therefore exhibit identical pharmacological properties [15]. Iodine-123, (*t*_1/2_ = 13.2 h), decays via electron capture (EC) and is used primarily for SPECT imaging. Notable clinical applications of ^123^I-labeled SPECT tracers include [^123^I]NaI for thyroid disease imaging [16], *meta*-[^123^I]iodobenzylguanidine ([^123^I]MIBG) for cardiac imaging [17,18], and [^123^I]Ioflupane for Parkinson’s disease diagnosis [19]. Further, ^123^I-labeled PARP inhibitors have also been used for Auger–Meitner electron-based radiotherapeutic applications [20]. Iodine-124 serves as a long-lived β^+^-emitting radionuclide (*t*_1/2_ = 4.2 d), which is particularly useful for long-term clinical and small animal studies [21]. Iodine-125 (*t*_1/2_ = 59.4 d) is typically used as an Auger–Meitner electron emitter for brachytherapy, and for preclinical radioligand assays [15,22]. Iodine-131 decays via EC and β^−^-emission (*t*_1/2_ = 8.02 days) and is considered the first example of a true theranostic radionuclide, due to its imaging and radiotherapeutic applications for thyroid diseases, such as thyrotoxicosis and thyroid cancer [23,24,25]. Currently, eight FDA-approved radiopharmaceuticals contain radioiodine [26].

Due to their multi-faceted utility in nuclear medicine applications, radiohalogenation reactions have been the focus of a plethora of radiosynthetic procedures [11,27,28,29,30,31,32,33]. However, translating halogenation techniques from conventional organic synthesis at higher concentrations (e.g., 0.1–1 M) to radiohalogenations is problematic due to the significantly different reactivity profiles of radiohalogens at the low concentrations typically used (1–10 mM and below). Therefore, novel and robust radiohalogenation techniques that may facilitate the development of future radiopharmaceuticals are highly sought after.

Radiolabeled peptides are vital tools for PET and SPECT imaging, owing to their high selectivity for cell surface receptors that are typically overexpressed in various cancers. Moreover, radiolabeled peptides are powerful biovectors for targeted radiotherapeutic applications (e.g., the theranostic approach) [34,35,36,37]. Consequently, robust radiolabeling protocols for the efficient development of radiohalogenated peptides hold significant potential for clinical applications. Despite the advancements in radiohalogenation techniques, the majority of radiohalogenated peptide preparations are performed through conjugation with prosthetic groups [32,38,39,40,41,42]. Indirect radiolabeling approaches are typically achieved via bioconjugation of a radiolabeled prosthetic group with lysine or cysteine residues [43,44]. Cysteine-selective thiol-based conjugation strategies are generally favored over lysine modification, due to increased regioselectivity and minimization of potential side-product formation [45,46]. Recently, Murphy et al. developed a promising prosthetic group containing a vinyl sulfone motif, which offers a number of radiosynthetic advantages compared to cysteine-specific ^18^F-labeling of peptides via maleimide-based synthons [32,47,48,49].

**Scheme 1:**
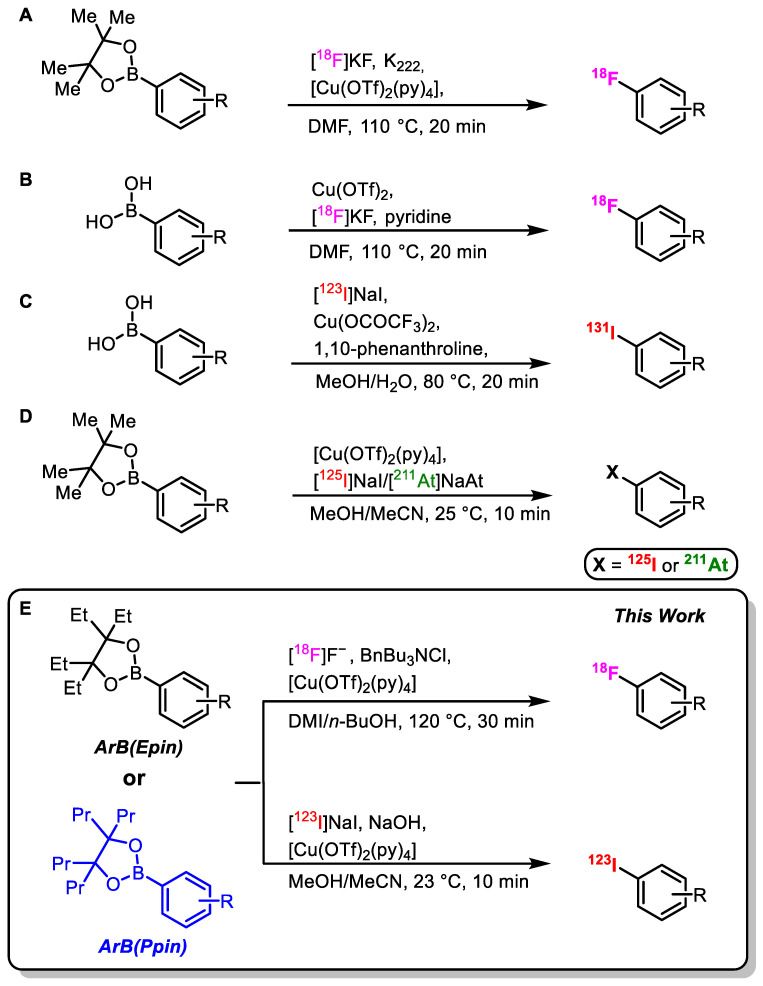
(**A**–**D**) Previous CMRH preparations of radiolabeled arenes. (**E**) This work: Preparation of radiolabeled arenes via CMRH of divergent ArB(Epin/Ppin) substrates.

Cu-mediated radiohalogenation (CMRH) protocols, which focus on the preparation of radiohalogenated arenes from aryl-stannanes or aryl-boronates, have proven to be efficient, robust, and practical for radiopharmaceutical preparations [50,51,52,53,54,55,56]. Gouverneur et al. applied the Cu-mediated radiofluorination (CMRF) approach towards the preparation of ^18^F-labeled arenes using electron-rich, electron-neutral, and electron-poor aryl-boronate ester substrates (Scheme 1A) [57]. The use of aryl-boronic acids by Scott et al. further expanded the scope of CMRF reactions (Scheme 1B) [51]. Radioiodination, radiobromination, and radioastatination via CMRH were also demonstrated to be feasible (Scheme 1C,D) [58,59,60]. Despite the progress of CMRH protocols, radiolabeling precursor synthesis can be challenging due to the inherent toxicity of stannyl compounds or to the relative instability of aryl-boronic ester groups, which may decompose during silica gel chromatography purification or in aqueous conditions. Oka et al. reported the use of aryl-boronic acid 1,1,2,2-tetraethylethylene glycol esters [ArB(Epin)s] as more stable substrates for Suzuki-Miyaura coupling reactions compared to the respective aryl-boronic acid and aryl-boronic acid 1,1,2,2-tetramethylethylene glycol ester [ArB(pin)s] counterparts [61]. The work attributed the increased stability to the four ethyl groups within ArB(Epin) compounds, which could dynamically protect the otherwise exposed empty orbital of the boron atom. The elevated stability presents practical synthetic advantages, as purification of the ArB(Epin) substrates using silica-based chromatographic techniques is facilitated, compound decomposition under alkaline conditions is significantly reduced, and the reactivity of the ArB(Epin) towards Suzuki-Miyaura coupling with aryl bromides was increased [62,63].

This work seeks to expand the substrate scope of CMRH protocols by utilizing the enhanced stability of ArB(Epin)s and novel aryl-boronic acid 1,1,2,2-tetrapropylethylene glycol esters (ArB(Ppin)) substrates towards efficient PET and SPECT radiotracer development (Scheme 1E).

## 2. Materials and Methods

### 2.1. Materials

Reagents and solvents were obtained from commercial suppliers and used as received without additional purification unless indicated. All solvents were of HPLC or analytical grade, and water used in all procedures was ultrapure (>18.2 MΩ·cm).

### 2.2. General Information

Unless otherwise stated, all moisture- and/or oxygen-sensitive reactions were carried out using the Schlenk technique under an argon atmosphere. For this, glass reaction vessels were heated several times under high vacuum (10^−3^ bar) with a heat gun and filled with argon. Solvents or liquid chemicals were added via septa using stainless steel cannulas. Thin-layer chromatography (TLC) was carried out using silica-coated Carl Roth plates (TLC plates ROTI^®^ChromaPlate Alu 60 UV, Art. No.: 1A18.1, Carl Roth GmbH + Co. KG, Karlsruhe, Germany) and visualization took place under UV light (254 nm and 366 nm) or with vanillin staining reagent. Crude products were purified via flash column chromatography at the Biotage^®^ Selekt System (SEL-2SV using Software Version 1.6.1.2, Biotage Sweden AB, Uppsala, Sweden) using BÜCHI FlashPure EcoFlex (BÜCHI Labortechnik AG, Flawil, Switzerland) chromatography cartridges. Beforehand, the crude products were dissolved in CH_2_Cl_2_, silica was added, and the mixture was evaporated under reduced pressure, and the crude product adsorbed on silica was dry loaded via a precolumn onto the system. The products were collected by tracking the UV activity in the range of 200 to 400 nm.

All NMR spectra were recorded at 25 °C using a Bruker Avance III 400 MHz/Agilent DD2-400 MHz (^1^H: 400 MHz, ^13^C: 101 MHz, ^19^F: 376 MHz) or Bruker Avance 600 Hz/Agilent DD2–600 MHz (^1^H: 600 MHz, ^13^C: 151 MHz). ^13^C-NMR spectra were recorded decoupled from ^1^H-NMR spectra. The evaluation of the NMR spectra was carried out using the program Mestrelab MestReNova (version 15.0.1).

High-resolution mass spectra were obtained on a Q-TOF MS using electrospray ionization: Agilent 1260 Infinity II HPLC (Santa Clara, CA, USA; pump G7104C, autosampler G7129C, column oven G7116A, DAD detector G7117C) coupled to γ detector Gabi Star (Raytest Isotopenmeßgeräte GmbH, Straubenhardt, Germany) followed by accurate mass Revident Q-TOF LC/Q-TOF G6575A. Unless otherwise stated, the measurements were performed in bypass mode using an eluent consisting of (A): MeCN and (B): 0.1% formic acid in H_2_O; flow rate 0.2 mL/min. A reference mass solution containing hexakis(1H,1H,3H-tetrafluoropropoxy)phosphazene, and purine was continuously co-injected via dual AJS ESI source. The system was operated using Agilent Masshunter Workstation 3.6—LC/MS data acquisition software (Version 12.0), and data evaluation was performed using Agilent Masshunter Workstation 3.6 Qualitative Analysis software (Version 12.0 Update 1).

An Acquity I-Class UPLC system (binary gradient pump BSM, autosampler FTN, column manager CM, and diode array detector PDAeλ) with a Xevo TQ-S mass spectrometer (Waters, Milford, MA, USA) was used to acquire low-resolution mass spectra and assess the purity of the prepared substances. Unless otherwise stated, mass spectra were obtained by positive electrospray ionization (ESI+) in bypass mode and purity analysis was performed by UPLC analysis (ACQUITY UPLC BEH C18 column (1.7 µm, 130 Å, 100 × 2.1 mm with respective VanGuard precolumn 5 × 2.1 mm) at a flow rate of 0.4 mL/min and gradient elution (mobile phase: A: 0.1% acetic acid in water (*v*/*v*) B: CH_3_OH/CH_3_CN/acetic acid 50/50/0.1 (*v*/*v*/*v*), t_0min_ 95/5–t_0.5min_ 95/5–t_5.5min_ 5/95–t_7.0min_ 5/95–t_8.0min_ 95/5–t_8.5min_ 95/5)). The UPLC chromatograms and mass spectra were analyzed using MassLynx 4.1 software.

All radiochemistry experiments were conducted at the Institute of Radiopharmaceutical Cancer Research, Helmholtz-Zentrum Dresden-Rossendorf (HZDR). [^18^F]Fluoride was generated via a (p,n) reaction by irradiating [^18^O]H_2_O with 18 MeV protons using the in-house TR-Flex cyclotron (Advanced Cyclotron Systems Inc., ACSI, Richmond, BC, Canada) [64]. No-carrier-added sodium [^123^I]iodide (Na [^123^I]I) was produced using the in-house TR-Flex cyclotron (ACSI) and the gas target KIPROS 200 from ZAG Zyklotron AG (Eggenstein-Leopoldshafen, Germany) by bombardment of highly enriched ^124^Xe gas with 30 MeV protons via, amongst others, the nuclear reaction ^124^Xe(p, pn)^123^Xe → ^123^I. Concentration of crude [^123^I]iodide and formulation in 0.02 M aqueous NaOH was performed by ROTOP Pharmaka GmbH at the HZDR campus. Aliquots containing [^123^I]iodide in an activity concentration of ~10 MBq/µL were used for further experiments and diluted accordingly with NaOH (0.02 M). Semi-preparative HPLC for determination of isolated radiochemical yield for radioiodinated compounds was performed on the following system: JASCO HPLC (interface LC-Net II/ADC, pump PU-2080Plus, gradient mixer LG-980-02; degasser DG-980-50, UV detector UV-2075Plus; γ detector Gabi Star (Raytest Isotopenmeßgeräte GmbH, Straubenhardt, Germany) with a 6-way column switching valve BESTA and fractionation valve BESTA; column Onyx Monolithic Semi-PREP C18, LC Column 100 × 10 mm, Tokyo, Japan); eluent: (A): 0.1% trifluoroacetic acid in H_2_O, (B): 0.1% trifluoroacetic acid in MeCN. flow rate 5 mL/min, gradient (eluent A/B): t_0min_ 95/5–t_2min_ 95/5–t_27min_ 5/95–t_30min_ 5/95–t_31min_ 95/5–t_40min_ 95/5.

Analytical Radio-(U)HPLC was conducted using a Shimadzu Nexera X2 UHPLC system (Shimadzu Corporation, Kyoto, Japan), equipped with the following components: degasser DGU-20A_3R_ and DGU-20A_5R_, pump LC-30AD, autosampler SIL-30AC, column oven CTO-20AC with two column switching valves FCV-14AH, diode array detector SPD-M30A, fluorescence detector RF-20A, γ detector Gabi Star (Raytest Isotopenmeßgeräte GmbH, Straubenhardt, Germany), communication bus module CBM-20A; UPLC column Kinetex C-18 (Phenomenex Inc., Torrance, CA, USA; 50 × 2.1 mm, 1.7 µm, 100 Å with Security Guard precolumn) or HPLC column Kinetex C-18 (Phenomenex Inc., Torrance, CA, USA; 250 × 4.6 mm, 5 µm, 100 Å with Security Guard precolumn), Gradient A Radio-UHPLC: flow rate 0.5 mL/min, eluent (A): 0.1% trifluoroacetic acid in H_2_O, (B): MeCN. (eluent A/B): t_0min_ 95/5–t_0.3min_ 95/5–t_4.5min_ 5/95–t_5.5min_ 5/95–t_6.0min_ 95/5–t_7.5min_ 95/5. Gradient B: Radio-HPLC 40 min: flow rate 1.0 mL/min, gradient (eluent A/B): t_0min_ 95/5–t_3.0min_ 95/5–t_28.0min_ 5/95–t_34.0min_ 5/95–t_35.0min_ 95/5–t_40.0min_ 95/5. Gradient C: Radio-HPLC 30 min: flow rate 1.0 mL/min, gradient (eluent A/B): t_0min_ 95/5–t_1.0min_ 95/5–t_21.0min_ 5/95–t_23.5min_ 5/95–t_25.0min_ 95/5–t_30.0min_ 95/5.

Semi-preparative HPLC for determination of isolated radiochemical yield for radiofluorinated compounds was performed on the following system: Agilent Series 1100 HPLC (interface Hewlett Packard 35900, pump G1311A, thermostatted column compartment G1316A; degasser G1322A, Variable Wavelength Detector (VWD) G1314A; γ detector Gabi Star (Raytest Isotopenmeßgeräte GmbH, Straubenhardt, Germany) with an external 4 port column switching valve VICI (AG); column Agilent ZORBAX SB-C18, 5 µm, 9.4 × 250 mm; eluent: (A): 0.1% trifluoroacetic acid in H_2_O, (B): 0.1% trifluoroacetic acid in MeCN. Flow rate 4 mL/min, gradient (eluent A/B): t_0min_ 90/10–t_3min_ 90/10–t_23min_ 5/95–t_27min_ 5/95–t_28min_ 90/10–t_33min_ 90/10.

Radiochemical conversion (RCC) was assessed by radio-(U)HPLC analysis of an aliquot taken from the crude radiofluorination reaction mixture (in MeCN), diluted with a 1:1 mixture of MeCN and H_2_O. RCC values were calculated from the relative peak areas of the chromatogram detected by the γ-detector. The identity of radiolabeled products was confirmed by comparing their radio-(U)HPLC traces with the UV-(U)HPLC traces of corresponding non-radioactive reference compounds. Determination of radiochemical purity (RCP) was carried out by peak integration within the chromatograms obtained from radio-(U)HPLC analysis. The isolated activity yield (AY) of a radiolabeled compound is the non-decay-corrected (n.d.c.) yield expressed as a percentage, calculated by dividing the activity of the product at the end of synthesis (E.O.S.) by the initial starting activity, then multiplying by one hundred. The molar activity (A_m_) was determined by HPLC through injection of a sample with a known activity amount, followed by quantification of the amount of substance injected. This was achieved by measuring the UV peak area corresponding to the radiolabeled product and calculating the concentration of the non-radioactive “carrier” using a calibration curve (see Supporting Information Figure S300).

### 2.3. General Procedure for the Synthesis of B(Epin) and B(Ppin) Compounds Starting from the Corresponding Boronic Acids

In a 10 mL one-neck flask, 2 mL of CH_2_Cl_2_ were placed under an argon atmosphere. 3,4-Diethylhexane-3,4-diol (**2**, Epin, 209 mg, 1.20 mmol, 1.2 eq.) or 4,5-dipropyloctane-4,5-diol (**4**, Ppin, 276 mg, 1.20 mmol, 1.2 eq.), the corresponding boronic acid (1.00 mmol, 1.0 eq.), and Na_2_SO_4_ (284 mg, 2.00 mmol, 2.0 eq.) were successively added (Scheme 3). The suspension was warmed and refluxed for 16 h. After cooling to room temperature, the reaction mixture was transferred to a separatory funnel with EtOAc and washed with water. The phases were separated and the aqueous phase was washed three times with EtOAc. The combined organic phases were dried over Na_2_SO_4_, filtered, and the solvent was removed in vacuo. After adsorption on silica gel, the crude product was purified by column chromatography on silica using a Biotage Select system (mobile phase gradient: 0–20% EtOAc in cyclohexane).

### 2.4. General Procedure for the Preparation of Radiolabeled Arenes via CMRF

The radiosynthetic procedure towards [^18^F]**33** and [^18^F]**56**–**74** was initiated with the adsorption of [^18^F]fluoride (50–10,000 MBq) on an anion exchange resin (QMA light carbonate cartridge, Waters Corp.). The cartridge was subsequently washed twice with dry MeOH (2 × 0.7 mL) from the male side, and ^18^F was eluted from the female side using 3.0 mg (9.6 µmol) BnBu_3_NCl in 0.7 mL MeOH (Scheme 5). The solvent was removed under reduced pressure at 70 °C for 5 min. The radiolabeling precursor (10 µmol) in 1.2 mL DMI/*n*-BuOH (2:1, *v*/*v*) was added to the cooled reaction vessel, and the reaction was conducted at 120 °C for 30 min under continuous stirring. The resulting crude reaction mixture, containing the radiofluorinated products, was quenched with 2 mL H_2_O and subjected to analytical radio-(U)HPLC for product identification and determination of the RCC. For this, an aliquot (5–20 µL) of the crude, quenched reaction mixture was taken, added to a mixture of (50–200 µL) H_2_O/MeCN (1:1, *v*/*v*), and subsequently analyzed. The RCC value was determined by integrating the peak area corresponding to the radiolabeled product, dividing this value by the total integrated peak area values, and multiplying the result by one hundred. Unless stated otherwise, each experiment was performed in triplicate.

For the purification of selected radiofluorinated compounds, dilution of the crude reaction mixture with 5 mL H_2_O was performed before loading the mixture onto an HLB cartridge solid-phase extraction (SPE) cartridge (hydrophilic lipophilic balance, Oasis^TM^ HLB Plus LP extraction cartridge, Waters Corp.). The HLB cartridge was washed with 10 mL H_2_O. Thereafter, elution of the radiolabeled product with 2 mL EtOH was performed prior to semi-preparative HPLC purification (mobile phase gradient: 10–95% MeCN in H_2_O, system Agilent 1100).

### 2.5. General Procedure for the Preparation of Radiolabeled Arenes via CMRI

The CMRI was performed according to a previously reported method [60,65]. Screening of substrate scope was performed for each substrate in duplicate by adding 0.5–2 MBq [^123^I]iodide in 0.02 M NaOH (1 µL) to a solution of the radiolabeling precursor in MeCN (10 µL, final concentration 0.8 mM, (U)HPLC grade) and [Cu(OTf)_2_(py)_4_] in MeOH (40 µL, final concentration: 4 mM, (U)HPLC grade). Without stirring, the reaction mixture was allowed to react for 10 min at room temperature. Afterwards, the solution was diluted with 200 µL H_2_O/MeCN (1:1, *v*/*v*). The reaction mixture was analyzed by radio-HPLC (System Shimadzu UPLC Gradient C) and identification was achieved by coinjection with the authentic non-radioactive reference at a concentration of 100 µg/mL.

EPin-based radiolabeling precursor **36** was radioiodinated applying the general procedure at a total volume of 306 µL (~73 MBq [^123^I]iodide in 6 µL 0.02 M NaOH; 300 µL 0.8 mM **36** and 4 mM [Cu(OTf)_2_(py)_4_] in MeOH/MeCN, 4:1) followed by dilution with 1.5 mL MeCN/H_2_O and semi-preparative HPLC (system JASCO HPLC, t_R_ ~15 min). Isolated radiochemical yield (RCY) was determined based on activity of product obtained after semi-preparative HPLC related to the starting activity.

## 3. Results

### 3.1. Organic Synthesis of Radiolabeling Precursors and Reference Compounds

**Scheme 2:**
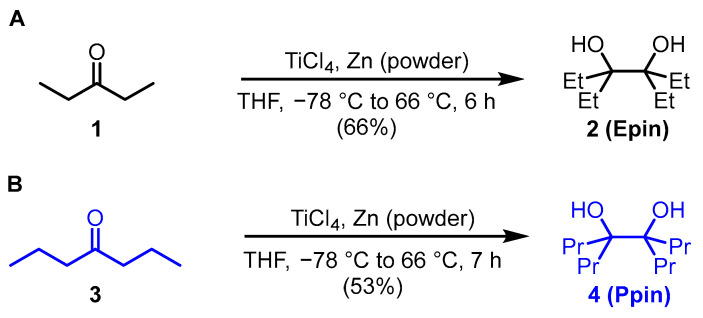
Preparation of (**A**) Epin (**2**) and (**B**) Ppin (**4**) from commercially available reagents.

**Scheme 3:**
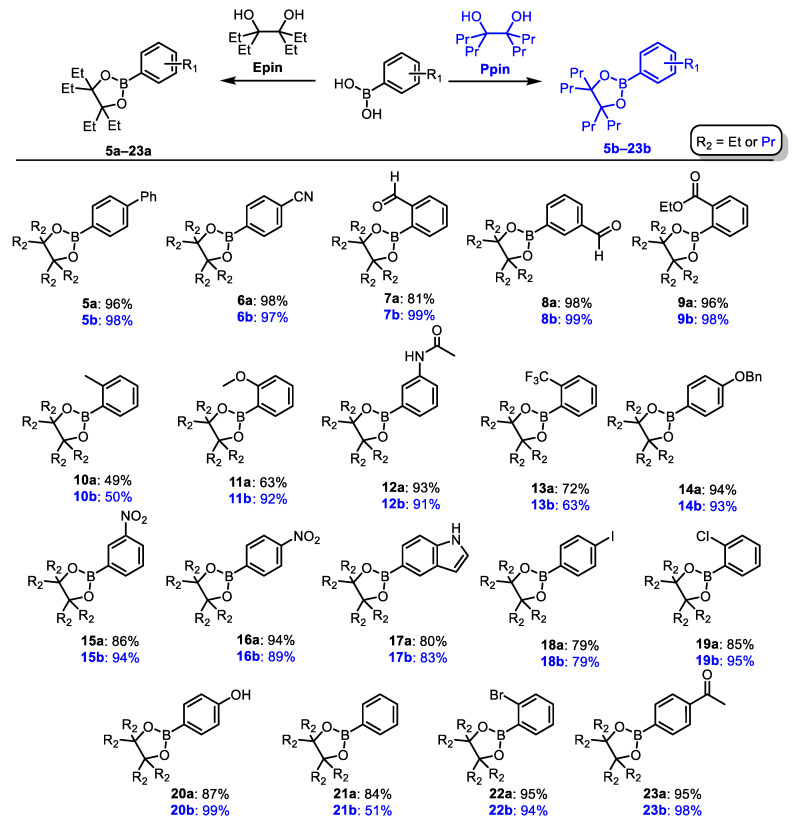
Preparation of ArB(Epin) and ArB(Ppin) radiolabeling precursors (**5a**–**23a**) and (**5b**–**23b**), respectively, from aryl-boronic acids. Conditions: ArB(OH)_2_ 1 (1.0 equiv), Epin (1.2 equiv), and Na_2_SO_4_ (2.0 equiv.) in CH_2_Cl_2_ (0.50 M) at 40 °C for 16 h.

The synthetic routes towards ArB(Epin) (**5a**–**23a**) and ArB(Ppin) (**5b**–**23b**) radiolabeling precursors started with the preparation of Epin (**2**) and Ppin (**4**) using a McMurry reaction following a slightly modified literature procedure (Scheme 2) [66]. The radiolabeling precursors were obtained following the dehydrative esterification of commercially available aryl-boronic acids with yields ranging from 49 to 99% (Scheme 3).

**Scheme 4:**
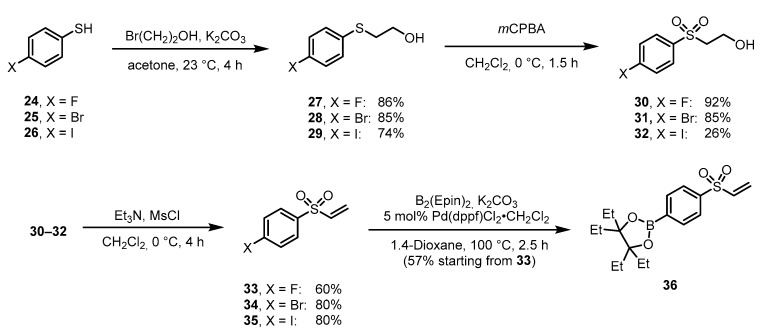
Preparation of divergent radiolabeling precursor **36** and reference compounds **33** and **35**.

The synthesis of the prosthetic group radiolabeling precursor **36**, for cysteine-specific bioconjugation of peptides, and the reference compounds **33** and **35** started from the thiophenols **24**–**26**, which were first alkylated using 2-bromoethanol (Scheme 4). Oxidation of **27**–**29** to the sulfones with *m*CPBA followed by dehydration, provided the reference substances **33** and **35** with overall yields of 47% and 15%, respectively. Thereafter, **34** was subsequently borylated with bis(ethylpinacolato)diboron to give the radiolabeling precursor **36** with an overall yield of 33%.

**Figure 1:**
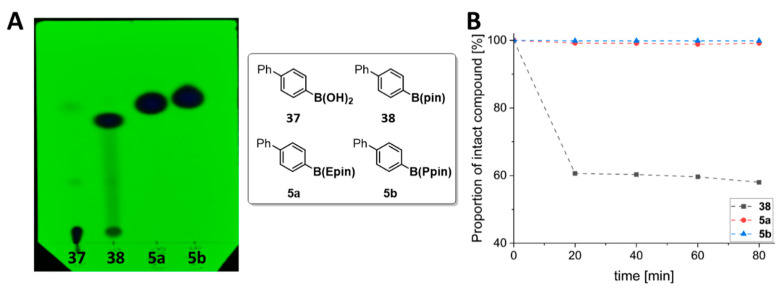
(**A**) UV-visualized TLC experiment using radiolabeling precursors **37**, **38**, **5a,** and **5b**, mobile phase: cyclohexane/EtOAc (4:1); (**B**) Stability studies using aliquots of **38**, **5a,** and **5b** in MeCN/H_2_O (1:1).

**Figure 2:**
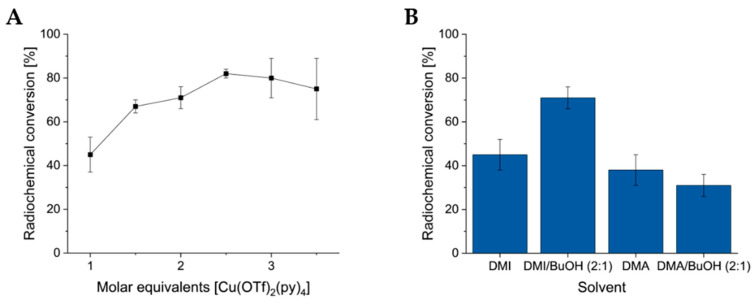
(**A**) ^18^F-Fluorination optimization with varying Cu mediator equivalents; and (**B**) CMRF optimization with various reaction solvents.

To investigate the stability of the ArB(Epin) and ArB(Ppin) compounds compared to their aryl-boronic acid and ArBpin counterparts, we evaluated the chromatographic behavior on TLC plates and the stability in aqueous acetonitrile (Figure 1). The stability of the four biphenyl compounds on silica-based thin-layer chromatography (TLC) plates was observed using UV-visualization (Figure 1A). The boronic acid **37** was not eluted by the mobile phase as expected, showing an R_f_ value of ~0. This finding suggests that the purification of aryl-boronic acids by normal phase chromatography (using this mobile phase system) could prove challenging. For Bpin **38**, a clear decomposition was observed as indicated by multiple spots and smearing on the TLC plate, with partial formation of **37** from **38** likely following deesterification. The ArB(Epin) (**5a**) and ArB(Ppin) (**5b**) derivatives were observed to be stable on TLC. Similarly, the radiolabeled precursor compounds **5a**–**23a** and **5b**–**23b** were observed to be stable on silica following TLC analysis (see Supporting Information Figure S216). Moreover, the stability of **38**, **5a,** and **5c** was evaluated when incubated in MeCN/H_2_O (1:1), a standard mobile phase system for reversed-phase chromatographic separations and for semi-preparative HPLC purifications (Figure 2B). Within only 20 min, approximately 40% compound degradation of the ArBpin derivative (**38**) to the corresponding aryl-boronic acid (**37**) was observed. This finding underlines that semi-preparative HPLC purification of radiolabeling precursors containing an ArBpin may prove challenging due to decomposition [54]. From our experiments, we have observed that both ArB(Epin) and ArB(Ppin) compounds have a similarly high stability on silica and in aqueous acetonitrile.

### 3.2. Substrate Scope of CMRI

**Scheme 5:**
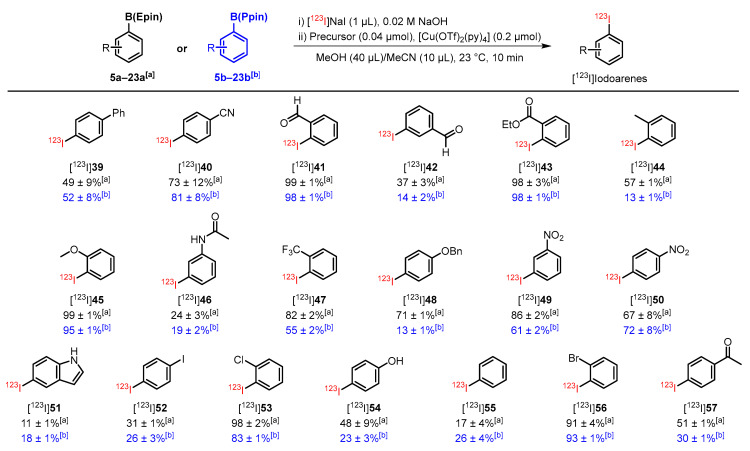
Substrate scope for the CMRI of ArB(Epin)s and ArB(Ppin)s. Radiolabeled products were prepared from the respective [a] (hetero)ArB(Epin), or [b] (hetero)ArB(Ppin) precursors. If not otherwise stated, mean (±) standard deviation values for RCC are provided. All experiments were performed at least in duplicate.

CMRI was performed as recently described to screen the substrate scope of ArB(Epin) and ArB(Ppin) precursors [60,65,67]. RCC was determined by radio-HPLC, and product identification was performed by coinjection of the authentic non-radioactive reference. Generally, radioiodination furnished the anticipated ^123^I-labeled products in RCC between 11 and 99%, while for ArB(Epin) substituted compounds, the RCC were typically comparable or higher compared to the respective ArB(Ppin)-substituted analogs (Scheme 5). Interestingly, the unsubstituted arenes phenyl-B(Epin) (**21a**) and phenyl-B(Ppin) (**21b**) afforded [^123^I]**55** only in low RCC values of 17% and 26%, respectively. For both ArB(Epin) and ArB(Ppin), substituents in the ortho-position were generally well-tolerated and afforded >80% RCC values, irrespective of the electron-withdrawing -CHO ([^123^I]**41**), -CO_2_Et ([^123^I]**43**), -CF_3_ ([^123^I]**47**), -Cl ([^123^I]**53**), or -Br ([^123^I]**56**) groups or of electron-donating -OMe ([^123^I]**45**). The only exception was [^123^I]**44** having a methyl group in the ortho-position to the -B(Epin) and -B(Ppin) groups which afforded lower RCC values of 57% and 13%, respectively. Para-substituted ArB(Epin) and ArB(Ppin) compounds with electron-donating groups like -Ph ([^123^I]**39**), -BnO ([^123^I]**48**) or -OH ([^123^I]**54**) as well as electron-withdrawing groups like -I ([^123^I]**52**), -COMe ([^123^I]**57**), -CN ([^123^I]**40**) or -NO_2_ ([^123^I]**50**) gave mostly lower but still moderate RCC values of 26–81%. For meta-substituted compounds, only a few examples have been employed here that give no obvious trend. Meta-substitution with -CHO ([^123^I]**42**) or -NHCOMe ([^123^I]**46**) was not well-tolerated, as indicated by RCC values of 19–37% while the meta-NO_2_ substituted compound [^123^I]**49** gave a high RCC of 86% from the Epin and 61% from the Ppin precursor. The only bicyclic heterocycle indole [^123^I]**51** in this substrate set was obtained in only a low RCC of 11% from the Epin and 18% from the Ppin precursor. Of note, for radioiodination of ArB(Ppin) substituted compounds in some cases an unidentified but common radioiodinated byproduct was formed in varying amounts which gives rise to the hypothesis that a stabilized [^123^I]IB(Ppin) species [68]. Forms in concurrence with the CMRI product (see Supporting Information Figure S218A–D).

In conclusion, in CMRI, both ArB(Epin) and ArB(Ppin) showed high tolerance for a broad aromatic substitution pattern. No aryl substituent completely prohibited reactivity in CMRI. However, ortho-substitution seems favored over para-substitution at this stage of the explored substrate scope.

### 3.3. Optimization of CMRF and Substrate Scope

**Scheme 6:**
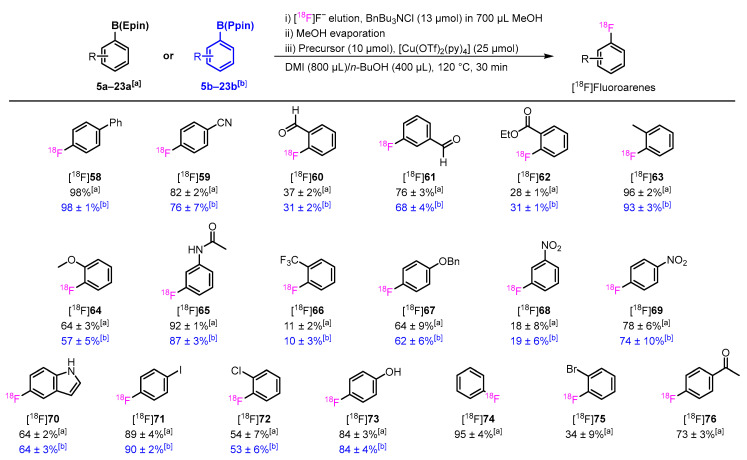
Substrate scope for the CMRF of ArB(Epin)s and ArB(Ppin)s. Radiolabeled products were prepared from the respective [a] (hetero)ArB(Epin), or [b] (hetero)ArB(Ppin) precursors. If not otherwise stated, mean (±) standard deviation values for RCC are provided. All experiments were performed at least in triplicate.

The optimization of the CMRF was performed by modifying one variable at a time using **6a** (NC-ArB(Epin) as a model substrate. The optimal molar equivalents of the [Cu(OTf)_2_(py)_4_] mediator were found to be 25 µmol with respect to 10 µmol **6a** (Figure 2A) for reactions in 1.2 mL. The highest RCC values were found when using the DMI/*n*-BuOH solvent (2:1) system (Figure 2B). Additionally, the optimal reaction temperature for the CMRF was found to be 120 °C for 30 min (see Supporting Information Table S8). The phase transfer agent (PTA) BnBu_3_NCl (3 mg, 13.1 µmol) provided slightly higher RCC with smaller deviations compared to conventional PTAs Et_4_NHCO_3_ (1.8 mg, 9.6 µmol) and Et_4_NOTf (2.68 mg, 9.6 µmol), although the overall values hardly differed (See Supporting Information Table S2) [11]. Using the optimized conditions, for both ArB(Epin) and ArB(Ppin), substituents in the para-position were generally well-tolerated and resulted in RCC values of >70% (Scheme 6), independent of the electron-withdrawing groups -CN ([^18^F]**59**), -NO_2_ ([^18^F]**69**), -I ([^18^F]**71**) or -COMe ([^18^F]**76**) or the electron-donating -Ph ([^18^F]**58**) or -OH([^18^F]**73**). The only exception was [^18^F]**67** with a benzyloxy group in para-position to the -B(Epin) and -B(Ppin) groups, which gave lower RCC values of 64% and 62%, respectively. Ortho-substituted ArB(Epin) and ArB(Ppin) compounds with stronger electron-withdrawing groups such as -CHO ([^18^F]**60**), -CO_2_Et ([^18^F]**62**) or -CF_3_ ([^18^F]**66**) were not well accepted, as shown by RCC values of 10–37%. The weakly electron-withdrawing group -Cl ([^18^F]**72**) and the electron-donating group -OMe ([^18^F]**64**) also showed only moderate RCCs of 53–64%. Only the simple -Me derivative ([^18^F]**63**) showed very good RCCs with 96% and 93% from the Epin and the Ppin precursor, respectively. Among the meta-substituted compounds, the electron-donating group -NHCOMe ([^18^F]**65**) was clearly better accepted than the electron-withdrawing groups -CHO ([^18^F]**61**) and -NO_2_ ([^18^F]**68**). The only bicyclic heterocycle indole [^18^F]**70** in this substrate set gave only moderate RCC values of 64% each from Epin and the Ppin precursor. Generally, the RCCs differed only marginally when the Epin or the Ppin precursor of the same derivative was used.

### 3.4. Preparation of Radiohalogenated Prosthetic Groups Towards Peptide Radiolabeling

**Scheme 7:**
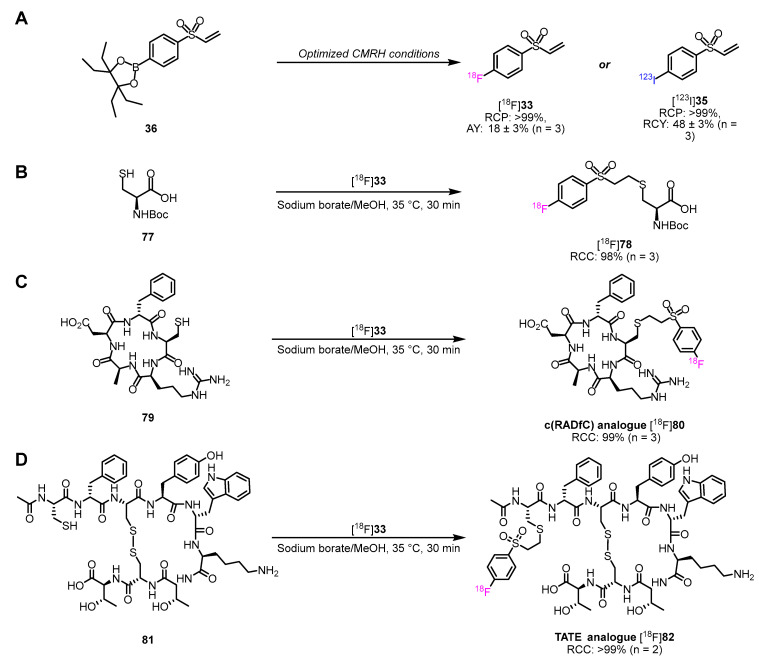
(**A**) Preparation of [^18^F]**33**; (**B**–**D**) Regioselective ^18^F-fluorination compounds via cysteine bioconjugation using [^18^F]**33**. Reaction conditions: Cys-containing compound (0.5–3.0 mg), [^18^F]**33** (50–500 MBq), sodium borate buffer pH 8.5/MeOH (1:1, 1–2 mL), 35 °C, 30 min. Radio-HPLC was used to determine the non-isolated RCC values of the radiolabeled products. The RCC was calculated by the division of the integrated product peak area by the total integrated ^18^F-labeled peaks. The identity of the ^18^F-labeled products was confirmed by coinjection with the “cold” ^19^F-reference standard.

Utilizing the optimized CMRH conditions for manual radiosynthesis, [^18^F]fluoro-4-(vinylsulfonyl)benzene ([^18^F]**33**) and [^123^I]iodo-4-(vinylsulfonyl)benzene ([^123^I]**35**) could be obtained from the radiolabeling precursor **36** with RCC values of 83 ± 2% and 26 ± 7%, respectively (Scheme 7A). Semi-preparative purification of [^18^F]**33** afforded the isolated product with 18 ± 3% AY. Similarly, following semi-preparative HPLC purification, the radioiodinated prosthetic group [^123^I]**33** was obtained with 48.7 ± 2.6% RCY. The bioconjugation using [^18^F]**33** was evaluated using three thiol-containing substrates in a similar manner as previously reported (Scheme 7B–D) [47]. *N*-Boc-cysteine and the cyclic cysteine-containing RGD analog peptide (RADfC, **79**) were applied as commercially available model substances and showed high RCC of 98% and 99%, respectively. Of note, RGD displays high affinity for the biomarker integrin α_v_β_3_ and radiolabeled RGD analogs have been used in PET for angiogenesis imaging [69,70]. Further, a cysteine-containing TATE analog (**81**) was designed and used for bioconjugation with [^18^F]**33**, allowing [^18^F]**82** as a potential ^18^F-labeled SSTR2 ligand in RCC of 99%. Currently, there exists no FDA or EMA-approved clinical ^18^F-labeled PET tracer for neuroendocrine tumor (NET) imaging as an alternative to the existing radiometal-based counterparts (e.g., [^68^Ga]Ga-DOTA-TATE, [^68^Ga]Ga-DOTA-TOC, or [^64^Cu]Cu-DOTA-TATE). However, [^18^F]SiTATE and [^18^F]AlF-NOTA-octreotide have both been under clinical investigation and early access programs in specific centers [71,72].

## 4. Discussion

The physicochemical properties of radiohalogens enable broad functional capabilities suited for diagnostic and therapeutic applications in nuclear medicine. Thus, radiohalogenation strategies play a significant role in the production and development of radiopharmaceuticals. Despite the advantages of CMRH protocols, the utility of the radiolabeling methodology can be hindered by the necessity to prepare and handle potentially unstable ArB(OH)_2_ or ArBpin and/or toxic aryl-stannane substrates in suitable quantities assuming 10–60 µmol of radiolabeling precursor needed per radiolabeling experiment [51,55,57,73,74]. For example, the carbon-boron (C-B) bond in radiolabeling precursor compounds containing ArB(OH)_2_ may cleave during functional group transformations [61]. Unfortunately, also purification of aryl-boronic acids or their ArBpin derivatives can in certain cases cause problems. Under acidic conditions or during chromatographic purification, hydrolysis of ArBpin esters to aryl-boronic acids as well as the formation of proto-deborylated or hydroxy-deborylated side products is frequently observed [62,63]. Moreover, the purification of ArB(OH)_2_ is non-trivial due to the strong absorption onto the silica gel stationary phase. However, reversed-phase purification of ArB(OH)_2_ may be employed to circumvent this limitation. Despite the relatively enhanced additional stability provided by ArB(pin) substrates, the purification of radiolabeling precursors containing ArB(pin) on silica gel should be performed rapidly in order to prevent significant losses of overall yield. Isobe et al. have developed a robust method for the purification of ArB(pin) derivatives on silica gel preconditioned with boric acid (B-silica gel) [75]; however, the resolution efficiency of B-silica gel is considerably lower than that of standard silica gel chromatography making purification using this technique a challenge [61].

The preparation of the ArB(Epin/Ppin)-substituted arenes (**5a**–**23a** and **5b**–**23b**) was achieved via one-step dehydrative esterification of commercially available aryl-boronic acids with either Epin (**2**) or Ppin (**4**). Oka et al. reported numerous methods of furnishing ArB(Epin)s via dehydrative esterification using various temperatures and solvents [61]. Pd-catalyzed Miyaura borylation of aryl bromides using a B_2_(Epin_2_) reagent, and metalation of aryl bromides also reportedly afforded ArB(Epin)s in high yields. Importantly, the stability of ArB(Epin) and ArB(Ppin) radiolabeling precursors facilitated purification via silica gel column chromatography, and the compounds provided clearly defined spots on silica gel thin-layer chromatography (TLC) compared to ArBpin compounds (Figure 2A).

The application of stable ArB(Epin) and novel ArB(Ppin) substrates allowed the facile preparation of radiohalogenated arenes via CMRH. From the corresponding aryl-boronic ester substrates, a small library of 19 radiofluorinated compounds [^18^F]**58**–**76**, and 19 radioiodinated compounds [^123^I]**39**–**57** containing various substituents and diverse electronic properties were obtained with RCC values of 7–99%, indicating excellent functional group tolerability. The RCC values obtained were comparable with the values previously reported in the literature, and only minor deviations in RCC values between ArB(Epin) and ArB(Ppin) substrates were observed in most cases. This observation was anticipated due to the CMRH reaction mechanism being the same for both substrate classes [76,77]. The optimization of the radiofluorination was performed by varying the reaction temperature, the molar equivalents of [Cu(OTf)_2_(py)_4_] with respect to the radiolabeling precursor, the PTA used for ^18^F-elution, and the reaction solvent. In nearly all cases, longer reaction times resulted in higher RCC values, likely due to the formation of the strong aryl-^18^F bond, which remained stable. Our findings identified the optimal CMRF conditions to include: 30 min reaction time, a reaction temperature of 120 °C, a reaction solvent of 1200 µL DMI/*n*-BuOH (2:1), BnBu_3_NCl as the optimal PTA, and 10 µmol radiolabeling precursor (10 µmol) and 25 µmol [Cu(OTf)_2_(py)_4_]. CMRH conditions were also applied towards selected aryl-boronic acids and ArBpins for comparison (see Supporting Information Tables S9–12). For the preparation of [^123^I]**44**, it was found that the aryl-boronic acid and ArBpin precursor derivatives afforded higher RCC values compared to the RCC values obtained using the precursors **10a** and **10b**. In this case, a radioiodinated side product was observed (see Supporting Information Figure S218). This side-product formation was also observed for selected radioiodination experiments. However, very similar RCC values were observed upon the preparation of [^18^F]**58** from **38**.

Aside from the enhanced stability of the radiolabeling precursors, the capacity of either ArB(Epin) and ArB(Ppin) radiolabeling precursors to be subjected to either CMRF or CMRI, allows either substrate to be used for the preparation of either a PET or SPECT radiotracer depending on the desired application [60]. Notably, due to the chemically identical nature of the various radioiodides,(15) CMRH of ArB(Epin) and ArB(Ppin) substrates using iodine-124/-125/-131 using our optimized CMRH protocol remains a distinct prospect, and may be the focus of a future study. Furthermore, the CMRH protocol may also be applied towards radiobromination and astatination of ArB(Epin)s and ArB(Ppin)s due to the similar CMRH mechanism as seen in earlier works [59,76,78,79].

Using the optimized radiolabeling conditions, we have furnished both an ^18^F-labeled and ^123^I-labeled prosthetic groups ([^18^F]**33** and [^123^I]**35**) from the starting radiolabeling precursor **36** (Scheme 7A) in high RCP. The optimized site-specific bioconjugation of [^18^F]**33** upon clinically relevant Cys-containing peptides (**79** and **81**) allows rapid access to both radiolabeled RGD and TATE analogs ([^18^F]**80** and [^18^F]**82**, respectively).

## 5. Conclusions

Our findings broaden the applicability of CMRH by introducing two new substrate classes, ArB(Epin)s and ArB(Ppin)s, to the radiochemist’s toolkit. Key advantages include improved precursor stability and moderate-to-high RCC values achieved through CMRH. This protocol provides a robust method for preparing radiohalogenated molecules for both diagnostic and potential therapeutic applications. Importantly, the enhanced stability of the B(Epin) and B(Ppin) groups offers greater flexibility in preparing radiolabeling precursors, which can improve PET and SPECT radiotracer development.

## Data Availability

Data is contained within the article and Supplementary Material.

## Supplementary Materials

The following supporting information can be downloaded at https://www.mdpi.com/article/10.3390/pharmaceutics17070837/s1. Experimental Procedures for Organic Syntheses, Copies of radio-(U)HPLC, HRMS, and NMR data are provided within the supporting information. References [47,61,64,80,81,82,83,84] are cited in the Supplementary Materials.
