# Supplementary material for: PET and SPECT Tracer Development via Copper-Mediated Radiohalogenation of Divergent and Stable Aryl-Boronic Esters
Source: Pharmaceutics. 2025 Jun 26;17(7):837. doi: 10.3390/pharmaceutics17070837 (PMC12298831; doi:10.3390/pharmaceutics17070837)
Supplement: Supplementary file 1 [file pharmaceutics-17-00837-s001.zip › pharmaceutics-3714996-supplementary.pdf]

Supporting Information

Contents

Organic Synthesis 2

General Experimental Section 2

Preparation of Epin Compounds 5

Preparation of PPin Compounds 30

Preparation of Compound 81 44

^{1}H, ^{13}C and ^{19}F NMR Spectra of EPin Compounds 45

^{1}H, ^{13}C and ^{19}F NMR Spectra of PPin Compounds 78

IR-Spectra of Novel Compounds 99

HR-MS of Novel Compounds 121

HPLC runs of the labeling precursors and determination of their purity 134

Stability testing on TLC 148

Radiochemistry 148

CMRH of established radiolabeling precursors 152

Chromatograms showing radioiodinated byproduct 153

Radio-HPLC and Radio-UHPLC Chromatograms 154

Determination of Molar Activity 185

References 186

Organic Synthesis

General Experimental Section

Unless stated otherwise, all solvents and reagents were obtained from commercial vendors, and utilized without additional purification. All solvents used in experiments were of HPLC or analytical grade, with the exception of water which was ultrapure (>18.2 MΩ cm^{-1}). Unless otherwise stated, all moisture and/or oxygen sensitive reactions were carried out using the Schlenk technique under an argon atmosphere. For this, glass reaction vessels were heated several times under high vacuum (10^{-3} bar) with a heat gun and filled with argon. Solvents or liquid chemicals were added via septa using stainless steel cannulas. Thin layer chromatography (TLC) was carried out using silica coated Carl Roth plates (TLC plates ROTI®ChromaPlate Alu 60 UV, Art. No.: 1A18.1) and visualization took place under UV light (254 nm and 366 nm) or with vanillin staining reagent. Crude products were purified via flash column chromatography at the Biotage® Selekt System using BÜCHI FlashPure EcoFlex chromatography cartridges. Beforehand, the crude products were dissolved in CH_2Cl_2, silica was added, and the mixture was evaporated under reduced pressure and the crude product adsorbed on silica was dry loaded via a pre-column onto the system. The products were collected by tracking the UV activity in the range of 200 to 400 nm. All NMR spectra were recorded at 25 °C using a Bruker Avance III 400 MHz/Agilent DD2-400 MHz (^{1}H: 400 MHz, ^{13}C: 101 MHz, ^{19}F: 376 MHz) or Bruker Avance 600 Hz/Agilent DD2–600 MHz (^{1}H: 600 MHz, ^{13}C: 151 MHz). ^{13}C-NMR spectra were recorded decoupled from ^{1}H-NMR spectra. The evaluation of the NMR spectra was carried out using the program Mestrelab MestReNova (version 15.0.1). ^{1}H and ^{13}C chemical shifts are reported in ppm relative to chloroform-d_3 (δ_H = 7.26; δ_C = 77.36). The observed signal multiplicities are characterized as follows: s = singlet, d = doublet, t = triplet, q = quartet, quin = quintet, m= multiplet, and br = broad. Coupling constants (*J* values) were reported in hertz (Hz). High-resolution mass spectra were obtained on a Q-TOF MS using electrospray ionization: Agilent 1260 Infinity II HPLC (Santa Clara, California, USA; pump G7104C, autosampler G7129C, column oven G7116A, DAD detector G7117C) coupled to γ detector Gabi Star (Raytest Isotopenmeßgeräte GmbH, Straubenhardt, Germany) followed by accurate mass Revident Q-TOF LC/Q-TOF G6575A. Unless otherwise stated, the measurements were performed in bypass mode using an eluent consisting of (A): MeCN and (B): 0.1% formic acid in H_2O; flow rate 0.2 mL/min). A reference mass solution containing hexakis(1H,1H,3H-tetrafluoropropoxy)phosphazene, and purine was continuously co-injected via dual AJS ESI source. The system was operated using Agilent Masshunter Workstation 3.6 – LC/MS data aquisition software (Version 12.0) and data evaluation was

performed using Agilent Masshunter Workstation 3.6 Qualitative Analysis software (Version 12.0 Update 1). An Acquity I-Class UPLC system (binary gradient pump BSM, autosampler FTN, column manager CM, and diode array detector PDAeλ) with a Waters Xevo TQ-S mass spectrometer (Waters, Milford, Massachusetts, USA), was used to acquire low-resolution mass spectra and assess the purity of the prepared substances. Unless otherwise stated, mass spectra were obtained by positive electrospray ionization (ESI+) in bypass mode and purity analysis was performed by UPLC analysis (ACQUITY UPLC BEH C18 column (1.7 µm, 130 Å, 100x2.1 mm with respective VanGuard precolumn 5x2.1 mm) at a flow rate of 0.4 ml/min and gradient elution (mobile phase: A: 0.1% acetic acid in water (*v*/*v*) B: CH_3OH/CH_3CN/acetic acid 50/50/0.1 (*v*/*v*/*v*), t_0 min 95/5 - $t_{0.5}$ min 95/5 - $t_{5.5}$ min 5/95 –$t_{7.0}$ min 5/95 - $t_{8.0}$ min 95/5 – $t_{8.5}$ min 95/5)). The UPLC chromatograms and mass spectra were analyzed using MassLynx 4.1 software.

All radiochemistry experiments were performed at the Institute of Radiopharmaceutical Cancer Research, Helmholtz-Zentrum Dresden-Rossendorf (HZDR). [^{18}F]Fluoride was produced via the (p,n) reaction using the in-house TR-Flex (Advanced Cyclotron Systems Inc., ACSI, Canada) cyclotron by irradiating [^{18}O]H_2O with 18 MeV protons.[1] No-carrier-added sodium [^{123}I]iodide (Na[^{123}I]I) was produced using the in-house TR-Flex cyclotron (ACSI) and the gas target KIPROS 200 from ZAG Zyklotron AG (Eggenstein-Leopoldshafen, Germany) by bombardment of highly enriched ^{124}Xe gas with 30 MeV protons via, amongst others, the nuclear reaction ^{124}Xe(p, pn)^{123}Xe → ^{123}I. Concentration of crude [^{123}I]iodide and formulation in 0.02 M aqueous NaOH was performed by ROTOP Pharmaka GmbH at the HZDR campus. Aliquots containing [^{123}I]iodide in an activity concentration of ~20–50 MBq/µL were used for further experiments and diluted accordingly with NaOH (0.02 M).

Semi-preparative HPLC for determination of isolated radiochemical yield was performed on the following system: Jasco HPLC (interface LC-Net II/ADC, pump PU-2080Plus, gradient mixer LG-980-02; degasser DG-980-50, UV detector UV-2075Plus; γ detector Gabi Star (Raytest Isotopenmeßgeräte GmbH, Straubenhardt, Germany) with a 6-way column switching valve BESTA and fractionation valve BESTA; column Onyx Monolithic Semi-PREP C18, LC Column 100 x 10 mm); eluent: (A): 0.1% trifluoroacetic acid in H_2O, (B): 0.1% trifluoroacetic acid in MeCN. flow rate 5 mL/min, gradient (eluent A/B): $t_{0\,min}$ 95/5 - $t_{2\,min}$ 95/5 – $t_{27\,min}$ 5/95 – $t_{30\,min}$ 5/95 – $t_{31\,min}$ 95/5 – $t_{40\,min}$ 95/5.

Analytical Radio-(U)HPLC was performed on the following system: Shimadzu Nexera X2 UHPLC system (Shimadzu Corporation, Kyoto, Japan; degasser DGU-20A_{3R} and DGU-20A_{5R}, pump LC-30AD, autosampler SIL-30AC, column oven CTO-20AC with two column switching valves FCV-14AH, diode array detector SPD-M30A, fluorescence detector RF-20A, γ detector Gabi Star (Raytest Isotopenmeßgeräte GmbH, Straubenhardt, Germany), communication bus

module CBM-20A; UPLC column Kinetex C-18 (Phenomenex Inc., Torrance, United States of America; 50 × 2.1 mm, 1.7 µm, 100 Å with Security Guard precolumn) or HPLC column Kinetex C-18 (Phenomenex Inc., Torrance, United States of America; 250 × 4.6 mm, 5 µm, 100 Å with Security Guard precolumn), Gradient A: Radio-UHPLC: flow rate 0.5 mL/min, eluent (A): 0.1% trifluoroacetic acid in H_2O, (B): MeCN. (eluent A/B): $t_{0\,min}$ 95/5 - $t_{0.3\,min}$ 95/5 - $t_{4.5\,min}$ 5/95 – $t_{5.5\,min}$ 5/95 - $t_{6.0\,min}$ 95/5 – $t_{7.5\,min}$ 95/5. Gradient B: Radio-HPLC 40 min: flow rate 1.0 mL/min, gradient (eluent A/B): $t_{0\,min}$ 95/5 – $t_{3.0\,min}$ 95/5- $t_{28.0\,min}$ 5/95 – $t_{34.0\,min}$ 5/95 – $t_{35.0\,min}$ 95/5 – $t_{40.0\,min}$ 95/5. Gradient C: Radio-HPLC 30 min: flow rate 1.0 mL/min, gradient (eluent A/B): $t_{0\,min}$ 95/5 – $t_{1.0\,min}$ 95/5- $t_{21.0\,min}$ 5/95 – $t_{23.5\,min}$ 5/95 – $t_{25.0\,min}$ 95/5 – $t_{30.0\,min}$ 95/5.

The radiochemical conversion (RCC) based on radio-UHPLC was determined by analyzing an aliquot after dilution of the crude radiofluorination reaction mixture (MeCN) with MeCN/H_2O (1:1) and are based on relative peak areas of the γ detector channel of the chromatogram. The identity of the radiolabeled products was determined by associating the UV-(U)HPLC traces of the suitable unlabeled reference compounds with the radio-(U)HPLC traces of the radiolabeled products. The radiochemical purity (RCP) of radiolabeled products was determined based on the integration of the peaks in the chromatogram in the radio-(U)HPLC. The isolated activity yield (AY) of a radiolabeled product is the non-decay-corrected (n.d.c.) yield given as a percentage value, which is determined by dividing the product activity at the end of radiosynthesis (E.O.S.) by the initial starting activity and multiplying by one hundred. The molar activity (A_m) was determined by HPLC analysis based on injection of a known activity amount followed by determination of the injected amount of substance. For that, the UV peak area corresponding to the radiolabeled product was determined and concentration of the non-radiolabeled “carrier” compound was calculated based on a calibration curve.

Preparation of Epin Compounds

3,4-Diethylhexane-3,4-diol (Epin, 2)

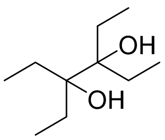

2

A 250 mL three-necked flask with a reflux condenser attached was heated three times under vacuum, placed under argon and pentan-3-one (3.45 g, 40.0 mmol, 1.0 eq.) in THF (100 mL) was added. The solution was cooled to -78 °C and $TiCl_4$ (6.59 mL, 11.4 g, 60.0 mmol, 1.5 eq.) was slowly added to the reaction mixture and stirring was continued at -78 °C for one hour. Then, zinc powder (7.85 g, 120 mmol, 3.0 eq) was added in small portions, the suspension was warmed and refluxed for 5 h. The reaction mixture was quenched by cooling to 0 °C and addition of 50 mL of saturated K_2CO_3 solution. The reaction mixture was transferred to a separatory funnel with plenty of EtOAc and washed with water and brine. The combined aqueous phases were extracted three times with EtOAc and the combined organic phases were dried over Na_2SO_4, filtered and the solvent was removed under reduced pressure. The crude product was adsorbed on silica gel and purified by column chromatography (silica, mobile phase: EtOAc:cyclohexane = 1:7). The product was obtained as a colorless oil (2.30 g, 13.1 mmol, **66%**).

R_f=0.33 (cyclohexane:EtOAc 4:1)

^{1}H NMR (400 MHz, $CDCl_3$) δ 1.87 (s, 2H), 1.71 – 1.53 (m, 8H), 0.94 (t, J = 7.5 Hz, 12H).

^{13}C NMR (101 MHz, $CDCl_3$) δ 79.00, 27.47, 9.17.

ATR FT-IR: $\tilde{\nu}$ [cm^{-1}] = 3472 (m), 3464 (m), 3454 (m), 3446 (m), 2967 (s), 2941 (s), 2882 (m), 1462 (m), 1383 (m), 1335 (w), 1265 (m), 1151 (w), 1122 (m), 1059 (w), 1038 (w), 1021 (w), 947 (m), 915 (m), 849 (w), 654 (w), 613 (w), 609 (w), 596 (w), 595 (w), 585 (w), 582 (w), 575 (w), 571 (w), 566 (w), 563 (w), 558 (w), 555 (w), 551 (w).

The NMR spectra are consistent with the literature.[2]

4,4,4',4',5,5,5',5'-Octaethyl-2,2'-bi(1,3,2-dioxaborolane) (B_2(Epin)$_2$, S1)

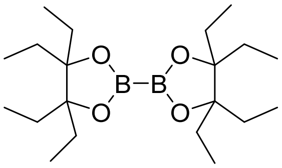

S1

A flask fitted with a Dean-Stark apparatus and reflux condenser was placed under argon. Toluene (30 mL) was added to the flask and the Dean-Stark apparatus (amount dependent on the volume of the apparatus). $B_2(OH)_4$ (300 mg, 3.35 mmol, 1.0 eq.), 3,4-diethylhexane-3,4-diol (Epin, 1.17 g, 6.69 mmol, 2.0 eq.) and KOAc (854 mg, 8.70 mmol, 2.6 eq.) were added, the suspension was heated and refluxed for 19 h. After cooling to 23 °C, the reaction mixture was filtered through Celite® 545 and the solvent is removed *in vacuo*. The crude product was adsorbed on silica gel and purified by column chromatography (silica, mobile phase: cyclohexane). The product was obtained as a colorless solid (396 mg, 1.08 mmol, **32%**).

R_f=0.57 (cyclohexane:EtOAc 4:1)

Melting point: 65.3-67.5 °C (open capillary)

^{1}H NMR (400 MHz, $CDCl_3$) δ 1.77 – 1.55 (m, 16H), 0.90 (t, J = 7.5 Hz, 24H).

^{13}C NMR (101 MHz, $CDCl_3$) δ 88.46, 26.55, 9.10.

ATR FT-IR: $\tilde{\nu}$ [cm^{-1}] = 2973 (s), 2948 (m), 2884 (m), 2859 (w), 2736 (w), 2359 (w), 2323 (w), 1475 (w), 1456 (m), 1436 (w), 1377 (w), 1356 (w), 1343 (m), 1310 (w), 1298 (w), 1267 (m), 1241 (w), 1222 (w), 1200 (w), 1182 (m), 1170 (m), 1140 (w), 1105 (m), 1066 (w), 1057 (w), 1037 (w), 1025 (w), 964 (w), 951 (w), 925 (m), 906 (m), 850 (w), 793 (w), 769 (w), 741 (w).

Exact mass calculated for $[M + Na]^+$: m/z = 389.3005; measured: m/z = 389.3009.

The NMR spectra are consistent with the literature.[3]

General procedure for the synthesis of B(Epin) compounds starting from the corresponding boronic acids

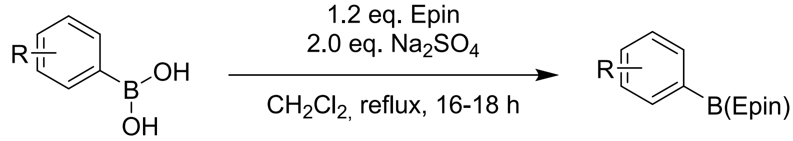

CH_2Cl_2 (2 mL) was placed in a 10 mL one-neck round-bottomed flask which has been evacuated three times and filled with argon. Under argon atmosphere, 3,4-Diethylhexane-3,4-diol (**2**, 209 mg, 1.20 mmol, 1.2 eq.), the corresponding boronic acid (1.00 mmol, 1.0 eq.), and Na_2SO_4 (284 mg, 2.00 mmol, 2.0 eq.) were successively added. The suspension was warmed and refluxed for 16 to 18 h. After cooling to room temperature (23 °C), the reaction mixture was transferred to a separatory funnel with EtOAc and washed with water. The phases were separated and the aqueous phase was washed three times with EtOAc. The combined organic phases were dried over Na_2SO_4, filtered, and the solvent was removed *in vacuo*. After adsorption on silica gel, the crude product was purified by column chromatography (silica, Biotage Select; eluent: EtOAc in cyclohexane 0% to 20%).

2-([1,1'-Biphenyl]-4-yl)-4,4,5,5-tetraethyl-1,3,2-dioxaborolane (5a)

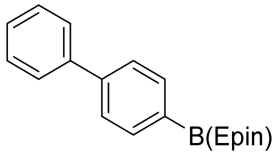

5a

Yield: 323.4 mg (962 µmol, **96%**)

Appearance: pale yellow solid

Purity$_{HPLC}$: 100%

R_f=0.61 (cyclohexane:EtOAc 4:1)

Melting point: 53.8-56.1 °C (open capillary)

^{1}H NMR (400 MHz, $CDCl_3$) δ 7.94 – 7.87 (m, 2H), 7.65 – 7.57 (m, 4H), 7.49 – 7.40 (m, 2H), 7.40 – 7.31 (m, 1H), 1.89 – 1.68 (m, 8H), 0.99 (t, *J* = 7.5 Hz, 12H).

^{13}C NMR (101 MHz, $CDCl_3$) δ 143.90, 141.30, 135.44, 128.90, 127.64, 127.40, 126.60, 88.95, 26.64, 9.02.

ATR FT-IR: $\tilde{\nu}$ [cm^{-1}] = 3080 (w), 3054 (w), 3039 (w), 3027 (w), 2994 (w), 2980 (m), 2967 (m), 2942 (m), 2880 (m), 2860 (w), 1610 (m), 1599 (m), 1549 (w), 1523 (w), 1477 (w), 1455 (m),

1446 (w), 1441 (w), 1401 (m), 1385 (m), 1367 (s), 1347 (s), 1312 (m), 1298 (m), 1292 (m), 1281 (m), 1260 (m), 1183 (w), 1140 (w), 1113 (w), 1096 (m), 1074 (w), 1065 (w), 1056 (w), 1037 (w), 1024 (w), 1010 (w), 956 (w), 918 (m), 857 (w), 852 (w), 844 (m), 821 (w), 765 (m), 740 (m), 698 (m), 674 (w), 657 (m), 588 (w).

Exact mass calculated for $[M + H]^+$: m/z = 337.2334; measured: m/z = 337.2337.

4-(4,4,5,5-Tetraethyl-1,3,2-dioxaborolan-2-yl)benzonitrile (6a)

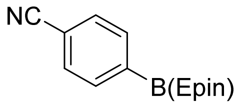

6a

Yield: 278.9 mg (978 µmol, **98%**)

Appearance: pale yellow solid

Purity$_{HPLC}$: 100%

R_f=0.49 (cyclohexane:EtOAc 4:1)

Melting point: 65.1-72.5 °C (open capillary)

^{1}H NMR (400 MHz, $CDCl_3$) δ 7.90 (d, *J* = 8.3 Hz, 2H), 7.64 (d, *J* = 8.4 Hz, 2H), 1.86 – 1.66 (m, 8H), 0.96 (t, *J* = 7.4 Hz, 12H).

^{13}C NMR (101 MHz, $CDCl_3$) δ 135.10, 131.11, 118.96, 114.34, 89.49, 26.39, 8.79.

ATR FT-IR: $\tilde{\nu}$ [cm^{-1}] = 2978 (m), 2948 (m), 2925 (m), 2882 (m), 2359 (w), 2227 (m), 1609 (w), 1505 (w), 1457 (m), 1436 (w), 1417 (w), 1398 (m), 1382 (m), 1366 (s), 1351 (s), 1339 (m), 1316 (m), 1292 (m), 1271 (m), 1183 (w), 1142 (w), 1127 (w), 1113 (w), 1102 (m), 1093 (m), 1088 (m), 1064 (w), 1040 (w), 1019 (m), 953 (w), 914 (m), 858 (w), 839 (m), 824 (w), 770 (w), 737 (w), 664 (w), 647 (m), 551 (w)

Exact mass calculated for $[M + H]^+$: m/z = 286.1973; measured: m/z = 286.1975.

The NMR spectra are consistent with the literature.[4]

2-(4,4,5,5-Tetraethyl-1,3,2-dioxaborolan-2-yl)benzaldehyde (7a)

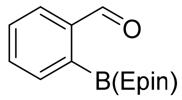

7a

Yield: 233.4 mg (810 µmol, **81%**)

Appearance: colorless solid

Purity$_{HPLC}$: 97%

R_f=0.48 (cyclohexane:EtOAc 4:1)

Melting point: 36.9-38.2 °C (open capillary)

^{1}H NMR (400 MHz, $CDCl_3$) δ 10.66 (d, *J* = 0.8 Hz, 1H), 8.00 – 7.97 (m, 1H), 7.96 – 7.92 (m, 1H), 7.63 – 7.51 (m, 2H), 1.94 – 1.68 (m, 7H), 0.98 (t, *J* = 7.5 Hz, 11H).

^{13}C NMR (101 MHz, $CDCl_3$) δ 195.14, 141.72, 136.31, 133.07, 131.02, 127.36, 89.77, 26.48, 9.03.

ATR FT-IR: $\tilde{\nu}$ [cm^{-1}] = 3061 (w), 3032 (w), 2976 (s), 2944 (m), 2881 (m), 1691 (s), 1663 (w), 1646 (w), 1591 (m), 1566 (w), 1485 (m), 1476 (w), 1456 (m), 1440 (w), 1403 (w), 1391 (m), 1377 (m), 1361 (m), 1344 (s), 1336 (s), 1312 (m), 1290 (m), 1257 (m), 1199 (m), 1185 (m), 1140 (w), 1112 (m), 1071 (m), 1063 (m), 1034 (m), 1009 (w), 964 (w), 957 (w), 917 (m), 853 (w), 833 (w), 806 (m), 768 (m), 737 (w), 681 (w), 669 (w), 651 (m), 633 (w).

3-(4,4,5,5-Tetraethyl-1,3,2-dioxaborolan-2-yl)benzaldehyde (8a)

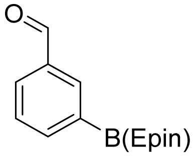

8a

Yield: 281.1 mg (975 µmol, **98%**)

Appearance: colorless solid

Purity$_{HPLC}$: 93%

R_f=0.47 (cyclohexane:EtOAc 4:1)

Melting point: 35.6-36.5 °C (open capillary)

^{1}H NMR (400 MHz, $CDCl_3$) δ 10.05 (s, 1H), 8.31 (s, 1H), 8.11 – 8.04 (m, 1H), 8.01 – 7.94 (m, 1H), 7.53 (t, J = 7.5 Hz, 1H), 1.88 – 1.67 (m, 8H), 0.98 (t, J = 7.5 Hz, 12H).

^{13}C NMR (101 MHz, $CDCl_3$) δ 192.87, 192.82, 140.92, 137.17, 135.91, 131.52, 128.50, 89.44, 26.62, 8.98.

ATR FT-IR: $\tilde{\nu}$ [cm^{-1}] = 2976 (m), 2947 (m), 2882 (m), 2858 (w), 2826 (w), 2789 (w), 2719 (w), 1701 (s), 1670 (w), 1604 (m), 1578 (w), 1488 (w), 1481 (w), 1456 (m), 1434 (m), 1395 (m), 1385 (m), 1368 (m), 1353 (s), 1316 (m), 1292 (m), 1276 (m), 1192 (m), 1166 (w), 1140 (w), 1112 (m), 1101 (m), 1075 (w), 1064 (m), 1024 (w), 1005 (w), 967 (w), 958 (w), 949 (w), 921 (m), 896 (w), 875 (m), 853 (w), 803 (m), 794 (m), 771 (w), 700 (m), 691 (m), 673 (w), 660 (m), 638 (w)

Exact mass calculated for [M + H]$^+$: m/z = 289.1970; measured: m/z = 289.1972.

Ethyl 2-(4,4,5,5-tetraethyl-1,3,2-dioxaborolan-2-yl)benzoate (9a)

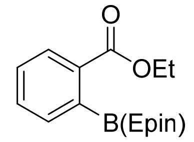

9a

Yield: 320.5 mg (965 µmol, **96%**)

Appearance: colorless oil

Purity$_{HPLC}$: 100%

R$_f$=0.49 (cyclohexane:EtOAc 4:1)

^{1}H NMR (400 MHz, $CDCl_3$) δ 7.90 (d, J = 7.7 Hz, 1H), 7.54 – 7.44 (m, 1H), 7.44 – 7.35 (m, 1H), 4.36 (q, J = 7.1 Hz, 2H), 1.95 (dq, J = 15.2, 7.6 Hz, 3H), 1.79 (dq, J = 14.6, 7.4 Hz, 3H), 1.37 (t, J = 7.1 Hz, 3H), 0.97 (t, J = 7.5 Hz, 11H).

^{13}C NMR (101 MHz, $CDCl_3$) δ 168.21, 134.48, 132.64, 131.66, 128.95, 128.63, 89.40, 61.28, 25.89, 14.50, 8.93.

ATR FT-IR: $\tilde{\nu}$ [cm^{-1}] = 2977 (m), 2943 (m), 2883 (w), 1712 (m), 1599 (w), 1569 (w), 1490 (w), 1476 (w), 1457 (w), 1441 (w), 1385 (m), 1361 (m), 1343 (m), 1274 (s), 1257 (m), 1173 (w), 1162 (w), 1136 (m), 1107 (m), 1060 (m), 1037 (m), 1021 (w), 958 (w), 919 (m), 854 (w), 814 (w), 775 (w), 767 (w), 746 (m), 706 (m), 689 (w), 674 (w), 654 (m), 641 (w).

Exact mass calculated for [M + Na]$^+$: m/z = 355.2051; measured: m/z = 355.2052.

4,4,5,5-Tetraethyl-2-(o-tolyl)-1,3,2-dioxaborolane (10a)

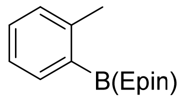

10a

Yield: 135.6 mg (494 µmol, **49%**)

Appearance: colorless liquid

Purity$_{HPLC}$: 100%

R_f=0.62 (cyclohexane:EtOAc 4:1)

^{1}H NMR (400 MHz, $CDCl_3$) δ 7.84 – 7.77 (m, 1H), 7.35 – 7.28 (m, 1H), 7.20 – 7.12 (m, 2H), 2.55 (s, 3H), 1.87 – 1.66 (m, 8H), 0.98 (t, *J* = 7.5 Hz, 12H).

^{13}C NMR (101 MHz, $CDCl_3$) δ 145.15, 136.32, 130.89, 129.92, 124.83, 88.62, 26.55, 22.37, 9.06.

ATR FT-IR: $\tilde{\nu}$ [cm^{-1}] = 3053 (w), 3017 (w), 2974 (m), 2943 (m), 2883 (m), 1601 (m), 1490 (w), 1456 (m), 1439 (m), 1386 (m), 1360 (m), 1342 (s), 1319 (m), 1303 (m), 1291 (m), 1281 (m), 1259 (m), 1185 (w), 1160 (w), 1141 (w), 1115 (m), 1074 (m), 1043 (m), 957 (w), 921 (m), 859 (w), 830 (w), 781 (w), 759 (w), 728 (m), 655 (m), 641 (w).

4,4,5,5-Tetraethyl-2-(2-methoxyphenyl)-1,3,2-dioxaborolane (11a)

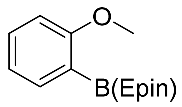

11a

Yield: 183.4 mg (632 µmol, **63%**)

Appearance: colorless oil

Purity$_{HPLC}$: 100%

R_f=0.44 (cyclohexane:EtOAc 4:1)

^{1}H NMR (400 MHz, $CDCl_3$) δ 7.67 (dd, *J* = 7.3, 1.9 Hz, 1H), 7.43 – 7.34 (m, 1H), 6.93 (t, *J* = 7.3 Hz, 1H), 6.85 (d, *J* = 8.3 Hz, 1H), 3.82 (s, 3H), 1.89 – 1.66 (m, 8H), 0.99 (t, *J* = 7.5 Hz, 12H).

^{13}C NMR (101 MHz, $CDCl_3$) δ 164.39, 136.90, 132.47, 120.32, 110.63, 88.64, 55.87, 26.49, 9.01.

ATR FT-IR: $\tilde{\nu}$ [cm^{-1}] = 3021 (w), 2973 (m), 2943 (m), 2883 (m), 2833 (w), 1599 (m), 1575 (m), 1488 (m), 1455 (m), 1431 (m), 1391 (m), 1361 (s), 1349 (s), 1305 (m), 1276 (m), 1245 (s), 1196 (w), 1178 (w), 1162 (w), 1140 (w), 1123 (m), 1115 (m), 1075 (m), 1046 (m), 1026 (m), 958 (w), 921 (m), 857 (w), 827 (w), 780 (w), 757 (m), 655 (m), 636 (w), 574 (w).

Exact mass calculated for $[M + Na]^+$: m/z = 313.1945; measured: m/z = 313.1948.

N-(3-(4,4,5,5-Tetraethyl-1,3,2-dioxaborolan-2-yl)phenyl)acetamide (12a)

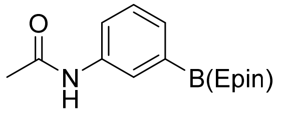

12a

Yield: 296.6 mg (935 µmol, **93%**)

Appearance: colorless solid

$Purity_{HPLC}$: 100%

R_f=0.52 (EtOAc)

Melting point: 106.7-107.8 °C (open capillary)

^1H NMR (600 MHz, $CDCl_3$) δ 7.91 (d, J = 7.8 Hz, 1H), 7.59 (s, 1H), 7.56 (d, J = 7.0 Hz, 1H), 7.37 – 7.32 (m, 1H), 7.13 (s, 1H), 2.16 (s, 3H), 1.83 – 1.67 (m, 8H), 0.96 (t, J = 7.5 Hz, 12H).

^{13}C NMR (101 MHz, $CDCl_3$) δ 168.50, 137.48, 130.84, 128.66, 125.90, 123.17, 89.03, 26.57, 24.66, 8.97.

ATR FT-IR: $\tilde{\nu}$ [cm^{-1}] = 3296 (m), 3218 (w), 3198 (w), 3161 (w), 3140 (w), 3102 (w), 3055 (w), 3031 (w), 2976 (m), 2950 (m), 2938 (m), 2883 (m), 2864 (w), 2810 (w), 1682 (m), 1663 (m), 1611 (m), 1592 (w), 1554 (s), 1507 (w), 1487 (m), 1456 (m), 1436 (w), 1424 (m), 1410 (m), 1399 (m), 1363 (s), 1349 (s), 1316 (m), 1306 (m), 1291 (m), 1257 (m), 1221 (m), 1183 (w), 1140 (w), 1106 (m), 1086 (w), 1075 (w), 1065 (w), 1057 (w), 1036 (w), 1020 (m), 959 (w), 921 (m), 903 (m), 854 (w), 797 (m), 771 (w), 753 (w), 704 (m), 672 (w).

Exact mass calculated for $[M + Na]^+$: m/z = 340.2054; measured: m/z = 340.2057.

4,4,5,5-Tetraethyl-2-(2-(trifluoromethyl)phenyl)-1,3,2-dioxaborolane (13a)

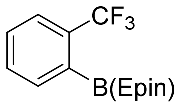

13a

Yield: 236.0 mg (719 µmol, **72%**)

Appearance: colorless liquid

Purity$_{HPLC}$: 100%

R_f=0.55 (cyclohexane:EtOAc 4:1)

^{1}H NMR (400 MHz, $CDCl_3$) δ 7.82 – 7.74 (m, 1H), 7.69 – 7.63 (m, 1H), 7.55 – 7.45 (m, 2H), 1.90 – 1.68 (m, 8H), 0.98 (t, *J* = 7.5 Hz, 12H).

^{13}C NMR (101 MHz, $CDCl_3$) δ 135.51, 130.82, 130.08, 125.39, 89.81, 26.15, 8.85.

^{19}F NMR (376 MHz, $CDCl_3$) δ -60.46.

ATR FT-IR: $\tilde{\nu}$ [cm^{-1}] = 2976 (m), 2946 (m), 2885 (w), 1605 (w), 1499 (w), 1477 (w), 1459 (w), 1384 (m), 1363 (m), 1342 (m), 1313 (s), 1286 (m), 1259 (w), 1161 (m), 1131 (m), 1108 (m), 1097 (m), 1062 (w), 1046 (m), 1034 (m), 957 (w), 917 (m), 823 (w), 772 (m), 749 (w), 678 (w), 655 (w), 598 (w).

2-(4-(Benzyloxy)phenyl)-4,4,5,5-tetraethyl-1,3,2-dioxaborolane (14a)

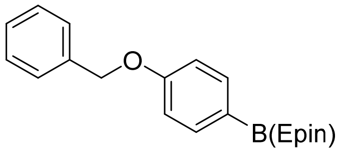

14a

Yield: 344.4 mg (940 µmol, **94%**)

Appearance: colorless oil

Purity$_{HPLC}$: 100%

R_f=0.52 (cyclohexane:EtOAc 4:1)

^{1}H NMR (600 MHz, $CDCl_3$) δ 7.80 – 7.75 (m, 2H), 7.46 – 7.42 (m, 2H), 7.41 – 7.36 (m, 2H), 7.35 – 7.29 (m, 1H), 7.00 – 6.95 (m, 2H), 5.10 (s, 2H), 1.75 (ddt, *J* = 33.3, 14.4, 7.4 Hz, 8H), 0.97 (t, *J* = 7.5 Hz, 12H).

^{13}C NMR (151 MHz, $CDCl_3$) δ 161.37, 137.04, 136.69, 128.72, 128.10, 128.09, 127.60, 127.59, 114.32, 88.67, 69.88, 26.58, 9.00.

ATR FT-IR: $\tilde{\nu}$ [cm^{-1}] = 3034 (w), 2975 (m), 2943 (w), 2883 (w), 1603 (s), 1570 (w), 1456 (w), 1440 (w), 1399 (m), 1382 (m), 1364 (s), 1350 (s), 1309 (m), 1292 (w), 1271 (w), 1240 (s), 1173 (m), 1141 (w), 1129 (w), 1116 (w), 1092 (m), 1065 (w), 1037 (w), 1024 (w), 1011 (w), 964 (w), 960 (w), 920 (m), 857 (w), 832 (w), 816 (w), 734 (m), 696 (w), 667 (w), 651 (m), 639 (w), 587 (w).

Exact mass calculated for $[M + H]^+$: m/z = 367.2439; measured: m/z = 367.2441.

4,4,5,5-Tetraethyl-2-(3-nitrophenyl)-1,3,2-dioxaborolane (15a)

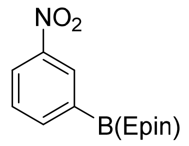

15a

Yield: 261.7 mg (857 µmol, **86%**)

Appearance: colorless solid

Purity$_{HPLC}$: 100%

R_f=0.50 (cyclohexane:EtOAc 4:1)

Melting point: 42.0-43.7 °C (open capillary)

^{1}H NMR (400 MHz, $CDCl_3$) δ 8.63 (d, *J* = 1.5 Hz, 1H), 8.32 – 8.25 (m, 1H), 8.15 – 8.08 (m, 1H), 7.58 – 7.49 (m, 1H), 1.87 – 1.67 (m, 8H), 0.98 (t, *J* = 7.5 Hz, 12H).

^{13}C NMR (101 MHz, $CDCl_3$) δ 148.03, 140.87, 129.50, 128.86, 125.88, 89.78, 26.61, 8.89.

ATR FT-IR: $\tilde{\nu}$ [cm^{-1}] = 3084 (w), 2976 (m), 2944 (m), 2882 (m), 1616 (m), 1571 (w), 1527 (m), 1484 (m), 1456 (m), 1436 (m), 1428 (m), 1399 (m), 1386 (m), 1367 (m), 1357 (m), 1342 (s), 1317 (m), 1301 (m), 1291 (m), 1270 (m), 1182 (w), 1140 (w), 1126 (m), 1109 (m), 1076 (w), 1062 (w), 1038 (w), 1023 (w), 1000 (w), 965 (w), 957 (w), 947 (w), 919 (m), 881 (m), 851 (w), 813 (m), 771 (w), 740 (w), 704 (m), 693 (m), 660 (w), 648 (m).

4,4,5,5-Tetraethyl-2-(4-nitrophenyl)-1,3,2-dioxaborolane (16a)

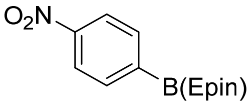

16a

Yield: 285.8 mg (936 µmol, **94%**)

Appearance: colorless solid

Purity$_{HPLC}$: 100%

R_f=0.51 (cyclohexane:EtOAc 4:1)

Melting point: 84.8-88.1 °C (open capillary)

^{1}H NMR (400 MHz, $CDCl_3$) δ 8.19 (d, *J* = 8.7 Hz, 2H), 7.97 (d, *J* = 8.6 Hz, 2H), 1.87 – 1.67 (m, 8H), 0.97 (t, *J* = 7.4 Hz, 12H).

^{13}C NMR (101 MHz, $CDCl_3$) δ 149.94, 135.81, 122.54, 89.81, 26.60, 8.95.

ATR FT-IR: $\tilde{\nu}$ [cm^{-1}] = 2979 (s), 2973 (s), 2949 (m), 2884 (m), 2843 (w), 2737 (w), 1597 (w), 1558 (w), 1540 (w), 1514 (s), 1495 (m), 1475 (w), 1456 (m), 1439 (w), 1399 (m), 1376 (m), 1368 (s), 1342 (s), 1307 (m), 1295 (m), 1279 (m), 1183 (w), 1140 (w), 1118 (w), 1110 (w), 1105 (w), 1091 (m), 1065 (w), 1042 (w), 1027 (w), 1015 (m), 966 (w), 960 (w), 920 (m), 853 (m), 811 (m), 806 (m), 774 (w), 754 (w), 696 (m), 653 (w), 639 (m), 587 (w).

The NMR spectra are consistent with the literature.[4]

5-(4,4,5,5-Tetraethyl-1,3,2-dioxaborolan-2-yl)-1H-indole (17a)

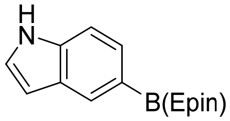

17a

Yield: 239.6 mg (801 µmol, **80%**)

Appearance: colorless solid

Purity$_{HPLC}$: 99%

R_f=0.20 (cyclohexane:EtOAc 4:1)

Melting point: 62.3-64.7 °C (open capillary)

^{1}H NMR (600 MHz, $CDCl_3$) δ 8.21 (d, J = 0.9 Hz, 1H), 8.19 (s, 1H), 7.68 (dd, J = 8.2, 1.2 Hz, 1H), 7.40 – 7.35 (m, 1H), 7.20 – 7.16 (m, 1H), 6.60 – 6.56 (m, 1H), 1.89 – 1.72 (m, 8H), 1.01 (t, J = 7.5 Hz, 12H).

^{13}C NMR (151 MHz, $CDCl_3$) δ 137.90, 128.75, 128.33, 127.73, 124.22, 110.54, 103.25, 88.53, 26.64, 9.05.

ATR FT-IR: $\tilde{\nu}$ [cm^{-1}] = 3389 (s), 3234 (w), 3218 (w), 3204 (w), 3185 (w), 3151 (w), 3133 (w), 3123 (w), 3114 (w), 3099 (w), 3051 (w), 2971 (m), 2950 (m), 2933 (m), 2882 (m), 2742 (w), 2359 (w), 2330 (w), 2322 (w), 1612 (m), 1575 (w), 1514 (m), 1476 (w), 1459 (m), 1443 (w), 1418 (m), 1396 (m), 1376 (m), 1363 (s), 1348 (s), 1318 (m), 1286 (m), 1276 (m), 1260 (m), 1249 (m), 1196 (w), 1187 (m), 1160 (w), 1139 (w), 1126 (m), 1115 (m), 1096 (m), 1072 (m), 1064 (m), 1040 (w), 1025 (w), 968 (w), 959 (w), 949 (w), 929 (m), 907 (m), 901 (m), 856 (w), 821 (w), 813 (w), 772 (w), 764 (w), 742 (m), 728 (m), 698 (w), 682 (m), 669 (w), 640 (w), 621 (w), 576 (w), 569 (w)

Exact mass calculated for $[M + H]^+$: m/z = 300.2130; measured: m/z = 300.2131.

4,4,5,5-Tetraethyl-2-(4-iodophenyl)-1,3,2-dioxaborolane (18a)

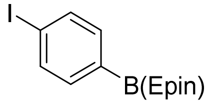

18a

Yield: 303.6 mg (786 µmol, **79%**)

Appearance: orange-brown solid

Purity$_{HPLC}$: 94%

R_f=0.61 (cyclohexane:EtOAc 4:1)

Melting point: 45.5-47.0 °C (open capillary)

^{1}H NMR (400 MHz, $CDCl_3$) δ 7.72 (d, J = 8.3 Hz, 2H), 7.53 (d, J = 8.0 Hz, 2H), 1.85 – 1.65 (m, 8H), 0.96 (t, J = 7.5 Hz, 12H).

^{13}C NMR (101 MHz, $CDCl_3$) δ 137.03, 136.50, 98.76, 89.17, 26.59, 8.97.

ATR FT-IR: $\tilde{\nu}$ [cm^{-1}] = 3046 (w), 2974 (s), 2948 (m), 2916 (m), 2882 (m), 2858 (w), 2781 (w), 2743 (w), 1924 (w), 1585 (m), 1559 (w), 1550 (w), 1475 (w), 1454 (m), 1437 (w), 1396 (m),

1385 (m), 1364 (s), 1346 (s), 1309 (m), 1291 (m), 1254 (m), 1184 (w), 1139 (w), 1114 (w), 1108 (w), 1091 (s), 1064 (w), 1056 (w), 1039 (w), 1023 (w), 1004 (m), 957 (w), 916 (m), 852 (w), 824 (m), 813 (m), 801 (w), 771 (w), 720 (m), 661 (w), 647 (m), 627 (w), 584 (w).

The ^{1}H NMR spectrum is consistent with the literature.[4]

2-(2-Chlorophenyl)-4,4,5,5-tetraethyl-1,3,2-dioxaborolane (19a)

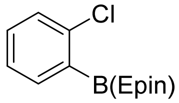

19a

Yield: 249.3 mg (846 µmol, **85%**)

Appearance: colorless liquid

Purity$_{HPLC}$: 100%

R_f=0.59 (cyclohexane:EtOAc 4:1)

^{1}H NMR (400 MHz, $CDCl_3$) δ 7.75 – 7.69 (m, 1H), 7.38 – 7.28 (m, 2H), 7.25 – 7.19 (m, 1H), 1.90 – 1.68 (m, 8H), 0.99 (t, J = 7.5 Hz, 12H).

^{13}C NMR (101 MHz, $CDCl_3$) δ 139.97, 136.91, 131.94, 129.60, 125.91, 89.40, 26.50, 9.00.

ATR FT-IR: $\tilde{\nu}$ [cm^{-1}] = 2974 (m), 2943 (m), 2883 (m), 1592 (m), 1562 (w), 1474 (m), 1458 (m), 1429 (m), 1384 (m), 1361 (s), 1353 (s), 1340 (s), 1307 (m), 1291 (m), 1268 (m), 1250 (w), 1196 (w), 1185 (w), 1160 (w), 1141 (w), 1128 (w), 1105 (s), 1063 (w), 1036 (m), 955 (w), 918 (m), 857 (w), 819 (w), 755 (m), 733 (m), 711 (w), 650 (m), 635 (w).

4-(4,4,5,5-Tetraethyl-1,3,2-dioxaborolan-2-yl)phenol (20a)

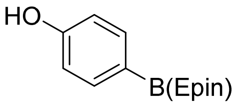

20a

Yield: 240.6 mg (871 µmol, **87%**)

Appearance: colorless solid

Purity$_{HPLC}$: 100%

R_f=0.17 (cyclohexane:EtOAc 4:1)

Melting point: 93.8-97.4 °C (open capillary)

^{1}H NMR (400 MHz, $CDCl_3$) δ 7.76 – 7.69 (m, 2H), 6.85 – 6.78 (m, 2H), 1.86 – 1.65 (m, 8H), 0.96 (t, *J* = 7.5 Hz, 12H).

^{13}C NMR (101 MHz, $CDCl_3$) δ 158.28, 136.97, 114.88, 88.75, 26.57, 8.99.

ATR FT-IR: $\tilde{\nu}$ [cm^{-1}] = 3300 (s), 3191 (w), 3166 (w), 3073 (w), 3038 (w), 2983 (m), 2956 (m), 2940 (m), 2883 (m), 2865 (w), 2797 (w), 2776 (w), 1607 (s), 1583 (m), 1471 (m), 1458 (m), 1428 (m), 1402 (m), 1379 (m), 1366 (m), 1356 (s), 1336 (s), 1311 (m), 1294 (m), 1278 (m), 1254 (m), 1216 (m), 1171 (m), 1143 (w), 1126 (m), 1096 (m), 1082 (m), 1061 (w), 1041 (w), 1031 (w), 1023 (w), 1014 (w), 964 (w), 957 (w), 947 (w), 912 (m), 856 (w), 836 (m), 823 (m), 802 (w), 774 (w), 765 (w), 731 (w), 704 (w), 673 (w), 665 (w), 651 (m), 634 (m), 598 (w), 564 (w).

4,4,5,5-Tetraethyl-2-phenyl-1,3,2-dioxaborolane (21a)

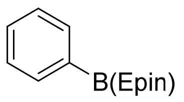

21a

Yield: 217.8 mg (837 µmol, **84%**)

Appearance: colorless liquid

Purity$_{HPLC}$: 100%

R_f=0.57 (cyclohexane:EtOAc 4:1)

^{1}H NMR (400 MHz, $CDCl_3$) δ 7.85 – 7.80 (m, 2H), 7.48 – 7.41 (m, 1H), 7.39 – 7.33 (m, 2H), 1.87 – 1.66 (m, 8H), 0.97 (t, *J* = 7.5 Hz, 12H).

^{13}C NMR (101 MHz, $CDCl_3$) δ 134.94, 131.21, 127.81, 88.89, 26.60, 9.00.

ATR FT-IR: $\tilde{\nu}$ [cm^{-1}] = 3055 (w), 3023 (w), 2974 (m), 2944 (m), 2883 (m), 1604 (m), 1498 (w), 1458 (w), 1439 (m), 1398 (m), 1384 (m), 1364 (s), 1348 (s), 1315 (m), 1303 (m), 1292 (m), 1273 (w), 1264 (m), 1181 (w), 1159 (w), 1141 (w), 1128 (w), 1115 (w), 1092 (m), 1066 (w), 1040 (w), 1027 (m), 958 (w), 919 (m), 857 (w), 814 (w), 760 (w), 698 (m), 669 (w), 652 (m).

2-(2-Bromophenyl)-4,4,5,5-tetraethyl-1,3,2-dioxaborolane (22a)

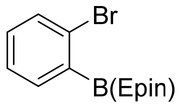

22a

Yield: 322.4 mg (951 µmol, **95%**)

Appearance: colorless liquid

Purity$_{HPLC}$: 100%

R_f=0.56 (cyclohexane:EtOAc 4:1)

^{1}H NMR (400 MHz, $CDCl_3$) δ 7.67 (dd, *J* = 7.1, 2.2 Hz, 1H), 7.56 – 7.51 (m, 1H), 7.31 – 7.20 (m, 2H), 1.90 – 1.68 (m, 8H), 1.00 (t, *J* = 7.4 Hz, 12H).

^{13}C NMR (101 MHz, $CDCl_3$) δ 137.11, 132.92, 132.02, 128.51, 126.40, 89.56, 26.46, 9.03.

ATR FT-IR: $\tilde{\nu}$ [cm^{-1}] = 2974 (m), 2943 (m), 2883 (m), 1588 (m), 1557 (w), 1470 (m), 1457 (m), 1424 (m), 1384 (m), 1361 (s), 1353 (s), 1337 (s), 1306 (m), 1290 (m), 1267 (m), 1248 (w), 1227 (w), 1195 (w), 1185 (w), 1160 (w), 1141 (w), 1129 (w), 1102 (m), 1063 (w), 1036 (m), 1020 (m), 955 (w), 917 (m), 856 (w), 817 (w), 754 (m), 728 (m), 689 (w), 649 (m), 634 (w).

1-(4-(4,4,5,5-Tetraethyl-1,3,2-dioxaborolan-2-yl)phenyl)ethan-1-one (23a)

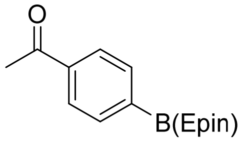

23a

Yield: 286.1 mg (947 µmol, **95%**)

Appearance: colorless oil

Purity$_{HPLC}$: 100%

R_f=0.33 (cyclohexane:EtOAc 4:1)

^{1}H NMR (400 MHz, $CDCl_3$) δ 7.96 – 7.87 (m, 4H), 2.61 (s, 3H), 1.87 – 1.67 (m, 8H), 0.97 (t, *J* = 7.5 Hz, 12H).

^{13}C NMR (101 MHz, $CDCl_3$) δ 198.63, 139.06, 135.10, 127.42, 89.37, 26.61, 8.98.

ATR FT-IR: $\tilde{\nu}$ [cm^{-1}] = 2975 (m), 2944 (m), 2884 (m), 1686 (m), 1557 (w), 1507 (m), 1458 (w), 1438 (w), 1428 (w), 1398 (m), 1385 (m), 1365 (m), 1349 (s), 1310 (m), 1292 (m), 1263 (m), 1195 (w), 1184 (w), 1161 (w), 1142 (w), 1128 (w), 1094 (m), 1065 (w), 1040 (w), 1018 (m), 955 (w), 917 (m), 854 (w), 832 (m), 821 (w), 771 (w), 741 (w), 730 (w), 667 (w), 650 (m), 633 (w), 602 (w), 593 (w)

Exact mass calculated for $[M + H]^+$: m/z = 303.2126; measured: m/z = 303.2128.

The NMR spectra are consistent with the literature.[3]

4-Iodobenzenethiol (26)

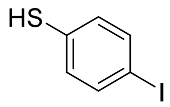

26

A 100 ml Schlenk flask was heated three times under vacuum and flushed with argon. 4-iodobenzenesulfonyl chloride (1.00 g, 3.31 mmol, 1.0 eq.) was initially dissolved in toluene (15 mL), and PPh_3 (2.60 g, 9.92 mmol, 3.0 eq.) was added. The reaction mixture was heated to 60 °C and kept at this temperature for 20 min. It was cooled to room temperature, and water was added. After stirring for a further 10 min, the reaction mixture was transferred to a separatory funnel with CH_2Cl_2 and extracted twice with 1 M NaOH. The combined aqueous phases were extracted three times with CH_2Cl_2, acidified with 1 M HCl, and extracted again three times with CH_2Cl_2. The solvent was removed under vacuum, and the crude product was adsorbed onto silica gel and purified by column chromatography (silica, Biotage Select; eluent: EtOAc in cyclohexane 0% to 50% gradient). 4-iodobenzenethiol (636 mg, 2.70 mmol, **82%**) was obtained as a white solid.

The ^1H NMR spectrum is consistent with the literature.[5]

2-((4-Fluorophenyl)thio)ethan-1-ol (27)

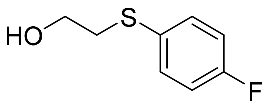

27

2-Bromoethane-1-ol (332 µL, 4.68 mmol, 1.2 eq.) was dissolved in acetone (5.0 mL) in a 25 mL one-neck round-bottomed flask and 4-fluorobenzenethiol (**24**, 500 mg, 3.90 mmol, 1.0 eq.) and K_2CO_3 (1.08 g, 7.80 mmol, 2.0 eq.) were added. The suspension was stirred at room temperature for 2.5 h and the reaction mixture was transferred to a separatory funnel with EtOAc. It was washed with water and the phases were separated. The aqueous phase was extracted three times with EtOAc and the combined organic phases were dried over anhydrous Na_2SO_4. It was filtered and the solvent was removed *in vacuo*. The crude product was adsorbed on silica gel and purified by column chromatography (silica, Biotage Select; eluent: EtOAc in cyclohexane 0% to 50% gradient). The product was obtained as a colorless liquid (579 mg, 3.36 mmol, **86%**).

R_f=0.19 (cyclohexane:EtOAc 4:1)

^{1}H NMR (400 MHz, $CDCl_3$) δ 7.45 – 7.35 (m, 2H), 7.06 – 6.95 (m, 2H), 3.71 (q, J = 6.0 Hz, 2H), 3.05 (t, J = 6.0 Hz, 2H), 2.05 (t, J = 6.1 Hz, 1H).

^{13}C NMR (101 MHz, $CDCl_3$) δ 162.31 (d, J = 247.3 Hz), 133.44 (d, J = 8.1 Hz), 129.78 (d, J = 3.4 Hz), 116.36 (d, J = 21.9 Hz), 60.31, 38.73 (d, J = 0.9 Hz).

^{19}F NMR (376 MHz, $CDCl_3$) δ -114.51 – -114.72 (m, 1F).

The NMR spectra are consistent with the literature.[6]

2-((4-Bromophenyl)thio)ethan-1-ol (28)

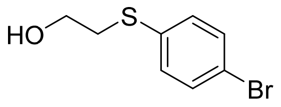

28

2-Bromoethane-1-ol (900 µL, 12.7 mmol, 1.2 eq.) was dissolved in acetone (13.5 mL) in a 50 mL one-neck round-bottomed flask and 4-bromobenzenethiol (**25**, 2.00 g, 10.6 mmol, 1.0 eq.) and K_2CO_3 (2.92 g, 2.12 mmol, 2.0 eq.) were added. The suspension was stirred at room temperature for 4 h and the reaction mixture was transferred to a separatory funnel with EtOAc. It was washed with water and the phases were separated. The aqueous phase was extracted three times with EtOAc and the combined organic phases were dried over anhydrous Na_2SO_4. It was filtered and the solvent was removed *in vacuo*. The crude product was adsorbed on silica gel and purified by column chromatography (silica, Biotage Select; eluent: EtOAc in

cyclohexane 0% to 50% gradient). The product was obtained as a colorless liquid (2.09 g, 8.99 mmol, **85%**).

R_f=0.16 (cyclohexane:EtOAc 4:1)

^{1}H NMR (400 MHz, $CDCl_3$) δ 7.41 (d, *J* = 8.6 Hz, 2H), 7.25 (d, *J* = 8.6 Hz, 2H), 3.75 (t, *J* = 6.0 Hz, 2H), 3.10 (t, *J* = 6.0 Hz, 2H), 1.91 (s, 1H).

^{13}C NMR (101 MHz, $CDCl_3$) δ 134.35, 132.26, 131.73, 120.72, 60.43, 37.45.

2-((4-Iodophenyl)thio)ethan-1-ol (29)

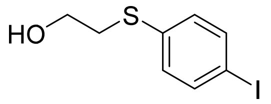

29

2-Bromoethane-1-ol (144 µL, 254 mg, 2.03 mmol, 1.2 eq.) was dissolved in acetone (2.2 mL) in a 25 mL one-neck round-bottomed flask and 4-iodobenzenethiol (**26**, 400 mg, 1.69 mmol, 1.0 eq.) and K_2CO_3 (468 mg, 3.39 mmol, 2.0 eq.) were added. The suspension was stirred at room temperature for 2 h and the reaction mixture was transferred to a separatory funnel with EtOAc. It was washed with water and the phases were separated. The aqueous phase was extracted three times with EtOAc and the combined organic phases were dried over anhydrous Na_2SO_4. It was filtered and the solvent was removed *in vacuo*. The crude product was adsorbed on silica gel and purified by column chromatography (silica, Biotage Select; eluent: EtOAc in cyclohexane 0% to 50% gradient). The product was obtained as a colorless liquid (353 mg, 1.26 mmol, **74%**).

R_f=0.22 (cyclohexane:EtOAc 4:1)

^{1}H NMR (400 MHz, $CDCl_3$) δ 7.64 – 7.56 (m, 2H), 7.15 – 7.08 (m, 2H), 3.75 (t, J = 6.0 Hz, 2H), 3.10 (t, J = 6.0 Hz, 2H), 1.96 (s, 1H).

^{13}C NMR (101 MHz, $CDCl_3$) δ 138.17, 135.30, 131.73, 91.64, 60.45, 37.22.

2-((4-Fluorophenyl)sulfonyl)ethan-1-ol (30)

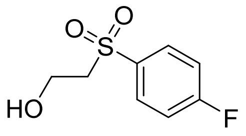

30

In a 50 mL one-neck round-bottomed flask, 2-((4-fluorophenyl)thio)ethan-1-ol (**27**, 523 mg, 3.04 mmol, 1.0 eq.) was dissolved in CH_2Cl_2 (10 mL). *Meta*-chloroperbenzoic acid (1.57 g, 9.12 mmol, 3.0 eq.) was carefully added in portions and the mixture was stirred for a further 1 h. The reaction mixture was adsorbed onto silica gel without further work-up and purified by column chromatography (silica, Biotage Select; eluent: EtOAc in cyclohexane 0% to 100% gradient). The product was obtained as a colorless liquid (573 mg, 2.81 mmol, **92%**).

R_f=0.43 (EtOAc)

^{1}H NMR (400 MHz, $CDCl_3$) δ 8.01 – 7.92 (m, 2H), 7.32 – 7.22 (m, 2H), 4.06 – 3.99 (m, 2H), 3.39 – 3.32 (m, 2H), 2.64 (br. s, 1H).

^{13}C NMR (101 MHz, $CDCl_3$) δ 166.19 (d, J = 257.4 Hz), 135.29 (d, J = 3.3 Hz), 131.09 (d, J = 9.7 Hz), 116.98 (d, J = 22.8 Hz), 58.63, 56.53.

^{19}F NMR (376 MHz, $CDCl_3$) δ -102.62 – -102.75 (m, 1F).

The NMR spectra are consistent with the literature.[6]

2-((4-Bromophenyl)sulfonyl)ethan-1-ol (31)

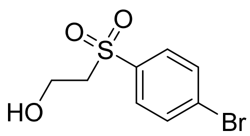

31

In a 50 mL one-neck round-bottomed flask, 2-((4-bromophenyl)thio)ethan-1-ol (**28**, 1.70 g, 7.29 mmol, 1.0 eq.) was dissolved in CH_2Cl_2 (24 mL) and cooled to 0 °C. *Meta*-chloroperbenzoic acid (3.78 g, 21.9 mmol, 3.0 eq.) was carefully added in portions and the mixture was stirred for a further 1.5 h. The reaction mixture was adsorbed onto silica gel without further work-up and purified by column chromatography (silica, Biotage Select; eluent: EtOAc in cyclohexane

0% to 100% gradient). The product was obtained as a colorless liquid (1.65 g, 6.22 mmol, **85%**).

R_f=0.51 (EtOAc)

^{1}H NMR (400 MHz, $CDCl_3$) δ 7.84 – 7.78 (m, 2H), 7.77 – 7.72 (m, 2H), 4.02 (t, *J* = 5.2 Hz, 2H), 3.39 – 3.32 (m, 2H).

^{13}C NMR (101 MHz, $CDCl_3$) δ 138.20, 132.89, 129.66, 129.59, 58.45, 56.35.

The NMR spectra are consistent with the literature.[7]

2-((4-Iodophenyl)sulfonyl)ethan-1-ol (32)

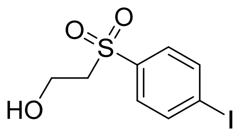

32

In a 50 mL one-neck round-bottomed flask, 2-((4-iodophenyl)thio)ethan-1-ol (**29**, 304 mg, 1.09 mmol, 1.0 eq.) was dissolved in CH_2Cl_2 (3.7 mL) and cooled to 0 °C. *Meta*-chloroperbenzoic acid (562 mg, 3.26 mmol, 3.0 eq.) was carefully added in portions and the mixture was stirred for a further 1.5 h. The reaction mixture was adsorbed onto silica gel without further work-up and purified by column chromatography (silica, Biotage Select; eluent: EtOAc in cyclohexane 0% to 100% gradient). The product was obtained as a colorless solid (89.0 mg, 285 µmol, 26%).

R_f=0.49 (EtOAc)

^{1}H NMR (400 MHz, $CDCl_3$) δ 8.01 – 7.92 (m, 2H), 7.67 – 7.62 (m, 2H), 4.06 – 3.99 (m, 2H), 3.38 – 3.31 (m, 2H).

^{13}C NMR (101 MHz, $CDCl_3$) δ 138.94, 138.87, 129.49, 102.30, 58.48, 56.49.

1-Fluoro-4-(vinylsulfonyl)benzene (33)

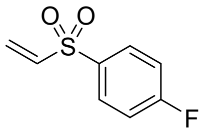

33

A 50 ml round-bottomed flask was heated three times in vacuum and flushed with argon. CH_2Cl_2 (16 mL) was placed it and triethylamine (911 µL, 665 mg, 6.57 mmol, 2.50 eq.) and 2-((4-Fluorophenyl)sulfonyl)ethan-1-ol (**30**, 537 mg, 2.63 mmol, 1.0 eq.) were added. The mixture was cooled to 0 °C. MsCl (407 µL, 602 mg, 5.26 mmol, 2.0 eq.) was added and the reaction mixture was stirred for 4 h at 0 °C. Water (10 mL) was then added, stirring was continued for 1 h, and the phases were transferred to a separatory funnel with CH_2Cl_2 and separated. The aqueous phase was extracted three times with CH_2Cl_2 and the combined organic phases were dried over $MgSO_4$, filtered and the solvent was removed *in vacuo*. The crude product was adsorbed on silica gel and purified by column chromatography (silica, Biotage Select; eluent: EtOAc in cyclohexane 0% to 100% gradient). The product **33** was obtained as a colorless liquid (293 mg, 1.57 mmol, **60%**).

$Purity_{HPLC}$: 100%

R_f=0.28 (cyclohexane:EtOAc 4:1)

^{1}H NMR (400 MHz, $CDCl_3$) δ 7.99 – 7.90 (m, 2H), 7.32 – 7.21 (m, 2H), 6.68 (dd, J = 16.5, 9.7 Hz, 1H), 6.49 (d, J = 16.5 Hz, 1H), 6.08 (d, J = 9.7 Hz, 1H).

^{13}C NMR (101 MHz, $CDCl_3$) δ 165.97 (d, J = 256.4 Hz), 138.55, 135.80 (d, J = 3.1 Hz), 130.97 (d, J = 9.7 Hz), 128.07, 116.84 (d, J = 22.7 Hz).

^{19}F NMR (376 MHz, $CDCl_3$) δ -103.44 – -103.60 (m, 1F).

ATR FT-IR: $\tilde{\nu}$ [cm^{-1}] = 3105 (w), 3062 (w), 1590 (m), 1492 (m), 1404 (w), 1382 (w), 1317 (m), 1290 (m), 1234 (m), 1160 (m), 1142 (s), 1097 (m), 1085 (m), 1012 (w), 975 (m), 833 (m), 818 (m), 742 (m), 707 (m), 674 (m), 609 (w).

The NMR spectra are consistent with the literature.[6]

1-Bromo-4-(vinylsulfonyl)benzene (34)

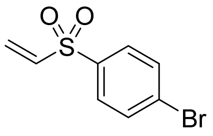

34

A 100 ml Schlenk flask was heated three times in vacuum and flushed with argon. CH_2Cl_2 (36 mL) was placed it and triethylamine (1.96 mL, 1.43 g, 14.1 mmol, 2.50 eq.) and 2-((4-bromophenyl)sulfonyl)ethan-1-ol (**31**, 1.50 g, 5.66 mmol, 1.0 eq.) were added. The mixture was cooled to 0 °C. MsCl (0.876 mL, 1.30 g, 11.3 mmol, 2.0 eq.) was added and the reaction mixture was stirred for 4 h at 0 °C. Water was then added, stirring was continued for 5 min, and the phases were transferred to a separatory funnel with CH_2Cl_2 and separated. The aqueous phase was extracted three times with CH_2Cl_2 and the combined organic phases were dried over $MgSO_4$, filtered and the solvent was removed *in vacuo*. The crude product was adsorbed on silica gel and purified by column chromatography (silica, Biotage Select; eluent: EtOAc in cyclohexane 0% to 100% gradient). The product **34** was obtained as a colorless liquid (1.11 g, 4.50 mmol, **80%**).

R_f=0.32 (cyclohexane:EtOAc 1:1)

^{1}H NMR (400 MHz, $CDCl_3$) δ 7.78 – 7.71 (m, 2H), 7.71 – 7.65 (m, 2H), 6.63 (dd, *J* = 16.5, 9.8 Hz, 1H), 6.46 (d, *J* = 16.7 Hz, 1H), 6.07 (d, *J* = 9.8 Hz, 1H).

^{13}C NMR (101 MHz, $CDCl_3$) δ 138.73, 138.22, 132.80, 129.57, 129.13, 128.54.

ATR FT-IR: $\tilde{\nu}$ [cm^{-1}] = 3088 (w), 3062 (w), 3028 (w), 3017 (w), 3006 (w), 2989 (w), 2937 (w), 2874 (w), 2852 (w), 2770 (w), 2726 (w), 2685 (w), 2679 (w), 2658 (w), 2640 (w), 2600 (w), 2582 (w), 2542 (w), 2505 (w), 2484 (w), 2462 (w), 2414 (w), 2399 (w), 2358 (w), 2341 (w), 2330 (w), 2323 (w), 2311 (w), 2299 (w), 2258 (w), 2232 (w), 2185 (w), 2177 (w), 2168 (w), 2162 (w), 2151 (w), 2122 (w), 2111 (w), 2098 (w), 2088 (w), 2084 (w), 2076 (w), 2074 (w), 2064 (w), 2061 (w), 2057 (w), 2050 (w), 2041 (w), 2037 (w), 2022 (w), 2010 (w), 1999 (w), 1991 (w), 1980 (w), 1964 (w), 1953 (w), 1949 (w), 1939 (w), 1920 (w), 1901 (w), 1575 (m), 1472 (w), 1465 (w), 1423 (w), 1417 (w), 1409 (w), 1392 (m), 1374 (w), 1337 (m), 1326 (m), 1309 (s), 1284 (m), 1257 (m), 1202 (w), 1166 (s), 1164 (s), 1144 (s), 1114 (w), 1085 (m), 1068 (m), 1057 (m), 1025 (m), 1009 (m), 986 (m), 979 (m), 954 (m), 899 (w), 834 (w), 818 (w), 786 (m), 777 (m), 753 (m), 734 (m), 712 (m), 697 (m), 623 (w), 610 (w), 569 (m).

The NMR spectra are consistent with the literature.[7]

1-Iodo-4-(vinylsulfonyl)benzene (35)

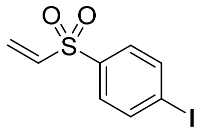

35

A 50 ml round-bottomed flask was heated three times in vacuum and flushed with argon. CH_2Cl_2 (2 mL) was placed it and triethylamine (94.8 µL, 69.2 mg, 684 µmol, 2.50 eq.) and 2-((4-Iodophenyl)sulfonyl)ethan-1-ol (**32**, 85.4 mg, 273 µmol, 1.0 eq.) were added. The mixture was cooled to 0 °C. MsCl (42.4 µL, 62.7 mg, 547 µmol, 2.0 eq.) was added and the reaction mixture was stirred for 6.5 h at 0 °C. Water (5 mL) was then added, stirring was continued for 1 h, and the phases were transferred to a separatory funnel with CH_2Cl_2 and separated. The aqueous phase was extracted three times with CH_2Cl_2 and the combined organic phases were dried over $MgSO_4$, filtered and the solvent was removed *in vacuo*. The crude product was adsorbed on silica gel and purified by column chromatography (silica, Biotage Select; eluent: EtOAc in cyclohexane 0% to 100% gradient). The product **35** was obtained as a colorless liquid (64.0 mg, 217 µmol, **80%**).

Purity$_{HPLC}$: 100%

R_f=0.33 (cyclohexane:EtOAc 4:1)

^{1}H NMR (400 MHz, $CDCl_3$) δ 7.96 – 7.87 (m, 2H), 7.64 – 7.56 (m, 2H), 6.64 (dd, J = 16.5, 9.7 Hz, 1H), 6.47 (d, J = 16.5 Hz, 1H), 6.07 (d, J = 9.7 Hz, 1H).

^{13}C NMR (101 MHz, $CDCl_3$) δ 139.47, 138.81, 138.29, 129.43, 128.51, 101.74.

ATR FT-IR: $\tilde{\nu}$ [cm^{-1}] = 3111 (m), 3089 (m), 3056 (m), 3026 (m), 3014 (m), 2960 (m), 2938 (m), 2919 (m), 2875 (w), 2848 (m), 2758 (w), 2713 (w), 2672 (w), 2646 (w), 2613 (w), 2604 (w), 2586 (w), 2568 (w), 2551 (w), 2527 (w), 2492 (w), 2473 (w), 2449 (w), 2424 (w), 2399 (w), 2389 (w), 2352 (w), 2339 (w), 2321 (w), 2252 (w), 2242 (w), 2225 (w), 2207 (w), 2202 (w), 2200 (w), 2191 (w), 2184 (w), 2177 (w), 2162 (w), 2149 (w), 2142 (w), 2138 (w), 2126 (w), 2111 (w), 2103 (w), 2100 (w), 2085 (w), 2072 (w), 2069 (w), 2061 (w), 2056 (w), 2049 (w), 2034 (w), 2027 (w), 2022 (w), 2015 (w), 1999 (w), 1996 (w), 1988 (w), 1980 (w), 1973 (w), 1964 (w), 1943 (w), 1921 (w), 1915 (w), 1892 (w), 1865 (w), 1842 (w), 1837 (w), 1829 (w), 1823 (w), 1798 (w), 1791 (w), 1784 (w), 1770 (w), 1766 (w), 1758 (w), 1748 (w), 1738

(w), 1732 (w), 1723 (w), 1715 (w), 1704 (w), 1698 (w), 1694 (w), 1682 (w), 1673 (w), 1667 (w), 1651 (w), 1645 (w), 1629 (w), 1615 (w), 1606 (m), 1565 (m), 1538 (w), 1532 (w), 1520 (w), 1516 (w), 1505 (w), 1495 (w), 1471 (m), 1440 (w), 1382 (m), 1368 (w), 1305 (s), 1273 (m), 1250 (m), 1180 (w), 1138 (s), 1106 (m), 1081 (m), 1052 (m), 1003 (m), 974 (m), 968 (m), 951 (m), 833 (w), 814 (m), 794 (w), 750 (m), 714 (m), 690 (m), 667 (w), 651 (m), 624 (w), 563 (m).

4,4,5,5-Tetraethyl-2-(4-(vinylsulfonyl)phenyl)-1,3,2-dioxaborolane (36)

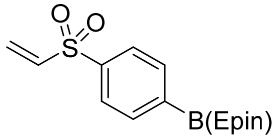

36

1-Bromo-4-(vinylsulfonyl)benzene (**34**, 100 mg, 405 µmol, 1.0 eq.), 4,4,4',4',5,5,5',5'-octaethyl-2,2'-bi(1,3,2-dioxaborolane) ($B_2(Epin)_2$, **S1**, 178 mg, 486 µmol, 1.2 eq.) and KOAc (119 mg, 1.21 mmol, 3.0 eq.) were weighed into a 25 mL Schlenk flask and suspended in 1,4-dioxane. The solvent was degassed for one hour in an argon stream before $Pd(dppf)Cl_2*CH_2Cl_2$ (16.5 mg, 20.2 µmol, 5 mol%) was added. The reaction was stirred for 2.5 h at 100 °C and then cooled to room temperature (23 °C). The reaction mixture was transferred to a separatory funnel with EtOAc and washed with water. The phases were separated and the aqueous phase was extracted three times with EtOAc. The combined organic phases were dried over Na_2SO_4, filtered and the solvent was removed *in vacuo*. The crude product was adsorbed on silica gel and purified by column chromatography (silica, Biotage Select; eluent: EtOAc in cyclohexane 0% to 20%). The product was obtained as a colorless solid (81.4 mg, 232 µmol, **57%**).

$Purity_{HPLC}$: 97%

R_f=0.42 (cyclohexane:EtOAc 4:1)

Melting point: 55.4-56.5 °C (open capillary)

^{1}H NMR (400 MHz, $CDCl_3$) δ 8.01 – 7.95 (m, 2H), 7.92 – 7.82 (m, 2H), 6.65 (dd, *J* = 16.5, 9.8 Hz, 1H), 6.46 (d, *J* = 16.6 Hz, 1H), 6.03 (d, *J* = 9.7 Hz, 1H), 1.86 – 1.66 (m, 8H), 0.96 (t, *J* = 7.5 Hz, 12H).

^{13}C NMR (101 MHz, $CDCl_3$) δ 141.67, 138.60, 135.70, 127.98, 127.00, 89.69, 26.61, 8.96.

ATR FT-IR: $\tilde{\nu}$ [cm^{-1}] = 3108 (w), 3051 (w), 2973 (m), 2945 (m), 2883 (m), 2860 (w), 1601 (w), 1496 (w), 1477 (w), 1456 (m), 1438 (w), 1417 (m), 1396 (m), 1367 (s), 1350 (m), 1313 (s), 1293 (m), 1271 (m), 1252 (w), 1182 (w), 1151 (s), 1114 (m), 1097 (m), 1080 (m), 1065 (m), 1020 (m), 995 (m), 968 (w), 958 (w), 917 (m), 848 (w), 837 (w), 765 (m), 755 (m), 733 (m), 705 (m), 665 (m), 647 (m), 631 (w), 588 (w), 564 (m).

Exact mass calculated for $[M + H]^+$: m/z = 351.1796; measured: m/z = 351.1798.

General procedure for the conjugation of 33 to cysteine-containing peptides

The peptide (1.0 eq.) was placed in a 2 mL Eppendorf tube and dissolved in 500 µL of a 1:1 mixture of methanol and sodium borate buffer (pH 8.5). **33** (1.1 eq.) was added, and the tube was shaken for 1 h at room temperature. The product was purified by HPLC, and the solvent was subsequently removed under vacuum.

HPLC conditions: Knauer Smartline system (Knauer Wissenschaftliche Geräte GmbH, Berlin, Germany) equipped with a Smartline pump 1000, the degasser system Smartline manager 5000 with performance at room temperature, UV-Vis-NIR Smartline detector 2500 for wavelength detection at 220 nm and 254 nm and a C18 column from Phenomenex (Gemini® , 5 µm, 110 Å, LC Column 250x10 mm, System). The purification was performed with the following conditions: 5–95% MeCN/H_2O, 5 min isocratic, then linear gradient in 25 minutes, then 5 min, with constant 0.1% v/v TFA additive and a flow rate of 6.00 mL/min.

N-(tert-butoxycarbonyl)-S-(2-((4-fluorophenyl)sulfonyl)ethyl)-L-cysteine (78)

Mass calculated for $[M - H]^-$: m/z = 406.08; measured: m/z = 406.24.

2-((2S,5R,8R,11S)-5-benzyl-8-(((2-((4-fluorophenyl)sulfonyl)ethyl)thio)methyl)-11-(3-guanidinopropyl)-3,6,9,12,15-pentaoxo-1,4,7,10,13-pentaazacyclopentadecan-2-yl)acetic acid (80)

Exact mass calculated for $[M + H]^+$: m/z = 779.2651; measured: m/z = 779.2661.

((4R,7S,10S,13R,16S,19R)-13-((1H-indol-3-yl)methyl)-19-((R)-2-((R)-2-acetamido-3-((2-((4-fluorophenyl)sulfonyl)ethyl)thio)propanamido)-3-phenylpropanamido)-10-(4-aminobutyl)-16-(4-hydroxybenzyl)-7-((S)-1-hydroxyethyl)-6,9,12,15,18-pentaoxo-1,2-dithia-5,8,11,14,17-pentaazacycloicosane-4-carbonyl)-L-allothreonine (82)

Mass calculated for $[M + H]^+$: m/z = 1379.46; measured: m/z = 1379.89.

Preparation of PPin Compounds

4,5-Dipropyloctane-4,5-diol (Ppin, 4)

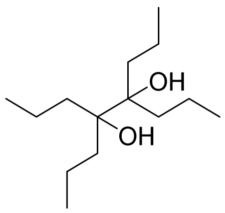

4

A 250 mL three-necked flask with a reflux condenser attached was heated three times under vacuum, placed under argon and heptan-3-one (4.57 g, 40.0 mmol, 1.0 eq.) in THF (100 mL) was added. The solution was cooled to -78 °C and $TiCl_4$ (6.59 mL, 11.4 g, 60.0 mmol, 1.5 eq.) was slowly added to the reaction mixture and stirring was continued at -78 °C for one hour. Then, zinc powder (7.85 g, 120 mmol, 3.0 eq) was added in small portions, the suspension was warmed and refluxed for 6 h. The reaction mixture was quenched by cooling to 0 °C and addition of 50 mL of saturated K_2CO_3 solution. The reaction mixture was transferred to a separatory funnel with plenty of EtOAc and washed with water and brine. The combined aqueous phases were extracted three times with EtOAc and the combined organic phases were dried over Na_2SO_4, filtered and the solvent was removed under reduced pressure. The crude product was adsorbed on silica gel and purified by column chromatography (silica, mobile phase: EtOAc:cyclohexane = 1:7). The product was obtained as a colorless solid (2.44 g, 10.6 mmol, **53%**).

R_f=0.43 (cyclohexane:EtOAc 4:1)

Melting point: 77.4-79.2 °C (open capillary)

^{1}H NMR (400 MHz, $CDCl_3$) δ 1.95 (s, 2H), 1.60 – 1.27 (m, 16H), 0.91 (t, *J* = 7.1 Hz, 12H).

^{13}C NMR (101 MHz, $CDCl_3$) δ 78.91, 38.20, 17.99, 15.23.

ATR FT-IR: $\tilde{\nu}$ [cm^{-1}] = 3428 (m), 3364 (m), 2958 (s), 2933 (m), 2871 (m), 1467 (w), 1456 (m), 1436 (w), 1399 (w), 1386 (m), 1380 (m), 1356 (w), 1318 (w), 1297 (w), 1286 (w), 1259 (w), 1245 (w), 1163 (w), 1139 (m), 1116 (w), 1066 (w), 994 (m), 970 (m), 909 (m), 889 (w), 873 (w), 845 (w), 659 (w), 594 (w), 555 (w).

The NMR spectra are consistent with the literature.[8]

General procedure for the synthesis of B(Ppin) compounds starting from the corresponding boronic acids

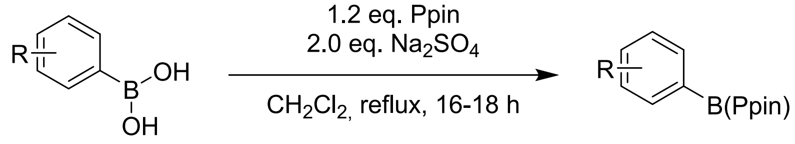

CH_2Cl_2 (2 mL) was placed in a 10 ml one-neck round-bottomed flask which has been evacuated three times and filled with argon. Under argon 4,5-Dipropyloctane-4,5-diol (**4**, 276 mg, 1.20 mmol, 1.2 eq.), the corresponding boronic acid (1.00 mmol, 1.0 eq.), and Na_2SO_4 (284 mg, 2.00 mmol, 2.0 eq.) were successively added. The suspension was warmed and refluxed for 16 to 18 h. After cooling to room temperature, the reaction mixture was transferred to a separatory funnel with EtOAc and washed with water. The phases were separated and the aqueous phase washed three times with EtOAc. The combined organic phases were dried over Na_2SO_4, filtered, and the solvent was removed *in vacuo*. After adsorption on silica gel, the crude product was purified by column chromatography (silica, Biotage Select; eluent: EtOAc in cyclohexane 0 % to 20 %).

2-([1,1'-Biphenyl]-4-yl)-4,4,5,5-tetrapropyl-1,3,2-dioxaborolane (5b)

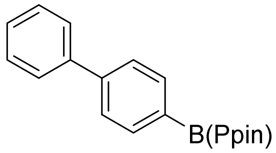

5b

Yield: 383.7 mg (978 µmol, **98%**)

Appearance: colorless oil

Purity$_{HPLC}$: 100%

R_f=0.66 (cyclohexane:EtOAc 4:1)

^{1}H NMR (400 MHz, $CDCl_3$) δ 7.92 – 7.85 (m, 2H), 7.65 – 7.57 (m, 4H), 7.49 – 7.40 (m, 2H), 7.40 – 7.31 (m, 1H), 1.76 – 1.59 (m, 8H), 1.57 – 1.44 (m, 4H), 1.43 – 1.30 (m, 4H), 0.94 (t, J = 7.2 Hz, 12H).

^{13}C NMR (101 MHz, $CDCl_3$) δ 143.94, 141.30, 135.45, 128.91, 127.65, 127.40, 126.61, 88.63, 37.26, 17.84, 15.00.

ATR FT-IR: $\tilde{\nu}$ [cm^{-1}] = 3053 (w), 3028 (w), 2959 (s), 2931 (m), 2872 (m), 1609 (m), 1550 (w), 1523 (w), 1465 (w), 1456 (w), 1399 (m), 1353 (s), 1328 (m), 1313 (m), 1276 (w), 1254 (w),

1185 (w), 1179 (w), 1157 (w), 1145 (w), 1134 (w), 1126 (w), 1090 (m), 1039 (w), 1022 (w), 1008 (w), 953 (w), 909 (w), 838 (m), 820 (w), 765 (m), 733 (m), 696 (m), 672 (w), 656 (m), 636 (w).

Exact mass calculated for $[M + H]^+$: m/z = 393.2960; measured: m/z = 393.2965.

4-(4,4,5,5-Tetrapropyl-1,3,2-dioxaborolan-2-yl)benzonitrile (6b)

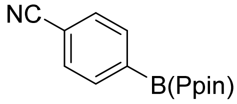

6b

Yield: 330.4 mg (968 µmol, **97%**)

Appearance: colorless solid

Purity$_{HPLC}$: 100%

R_f=0.56 (cyclohexane:EtOAc 4:1)

Melting point: 61.7-64.5 °C (open capillary)

^{1}H NMR (400 MHz, $CDCl_3$) δ 7.91 – 7.84 (m, 2H), 7.66 – 7.60 (m, 2H), 1.73 – 1.58 (m, 8H), 1.54 – 1.40 (m, 4H), 1.40 – 1.24 (m, 4H), 0.94 (t, J = 7.3 Hz, 12H).

^{13}C NMR (101 MHz, $CDCl_3$) δ 135.27, 131.28, 119.09, 114.55, 89.36, 37.15, 17.79, 14.93.

ATR FT-IR: $\tilde{\nu}$ [cm^{-1}] = 2955 (s), 2930 (m), 2902 (m), 2871 (m), 2230 (m), 1609 (w), 1506 (w), 1470 (w), 1455 (w), 1399 (m), 1366 (m), 1352 (s), 1336 (m), 1313 (m), 1287 (w), 1280 (w), 1269 (m), 1259 (w), 1251 (m), 1187 (w), 1157 (w), 1149 (w), 1134 (m), 1119 (w), 1098 (w), 1086 (m), 1070 (w), 1021 (m), 993 (w), 955 (m), 909 (w), 882 (w), 835 (m), 825 (w), 749 (w), 736 (w), 656 (w), 643 (m), 564 (w).

Exact mass calculated for $[M + H]^+$: m/z = 342.2599; measured: m/z = 342.2602.

2-(4,4,5,5-Tetrapropyl-1,3,2-dioxaborolan-2-yl)benzaldehyde (7b)

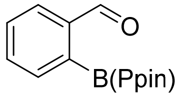

7b

Yield: 344.1 mg (1.00 mmol, **quant.**)

Appearance: pale yellow oil

$Purity_{HPLC}$: 94%

R_f=0.55 (cyclohexane:EtOAc 4:1)

^{1}H NMR (400 MHz, $CDCl_3$) δ 10.65 (s, 1H), 8.02 – 7.96 (m, 1H), 7.95 – 7.89 (m, 1H), 7.63 – 7.51 (m, 2H), 1.78 – 1.61 (m, 8H), 1.54 – 1.41 (m, 4H), 1.41 – 1.29 (m, 4H), 0.95 (t, *J* = 7.3 Hz, 12H).

^{13}C NMR (101 MHz, $CDCl_3$) δ z195.21, 195.19, 141.78, 136.39, 133.06, 131.07, 127.25, 89.38, 37.06, 17.88, 14.94.

ATR FT-IR: $\tilde{\nu}$ [cm^{-1}] = 2959 (s), 2932 (m), 2872 (m), 1695 (m), 1666 (w), 1594 (m), 1567 (w), 1486 (w), 1464 (m), 1456 (m), 1376 (m), 1367 (m), 1342 (s), 1311 (m), 1278 (w), 1254 (m), 1200 (m), 1180 (w), 1158 (w), 1146 (w), 1127 (w), 1109 (m), 1065 (m), 1036 (w), 1009 (w), 996 (w), 952 (w), 929 (w), 908 (w), 881 (w), 834 (w), 809 (w), 764 (w), 738 (w), 667 (w), 652 (w), 635 (w).

3-(4,4,5,5-Tetrapropyl-1,3,2-dioxaborolan-2-yl)benzaldehyde (8b)

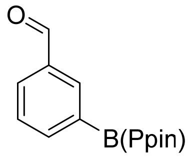

8b

Yield: 344.0 mg (1.00 mmol, **quant.**)

Appearance: colorless solid

$Purity_{HPLC}$: 93%

R_f=0.51 (cyclohexane:EtOAc 4:1)

Melting point: 54.6-56.3 °C (open capillary)

^{1}H NMR (400 MHz, $CDCl_3$) δ 10.05 (s, 1H), 8.29 (s, 1H), 8.09 – 8.02 (m, 1H), 8.01 – 7.94 (m, 1H), 7.53 (t, *J* = 7.4 Hz, 1H), 1.75 – 1.58 (m, 8H), 1.57 – 1.42 (m, 4H), 1.42 – 1.27 (m, 4H), 0.94 (t, *J* = 7.3 Hz, 12H).

^{13}C NMR (101 MHz, $CDCl_3$) δ 192.83, 140.93, 137.19, 135.92, 131.52, 128.55, 89.12, 37.21, 17.82, 14.96.

ATR FT-IR: $\tilde{\nu}$ [cm^{-1}] = 2960 (s), 2931 (m), 2871 (m), 2822 (w), 2811 (m), 2749 (w), 2724 (w), 2663 (w), 2626 (w), 2596 (w), 2358 (w), 1698 (s), 1662 (w), 1600 (m), 1580 (w), 1481 (w), 1469 (m), 1456 (m), 1435 (w), 1403 (m), 1393 (m), 1362 (m), 1348 (s), 1339 (m), 1320 (m), 1310 (m), 1287 (m), 1270 (w), 1256 (m), 1227 (w), 1190 (m), 1168 (m), 1158 (w), 1146 (m), 1133 (m), 1120 (m), 1096 (w), 1086 (w), 1072 (m), 1018 (w), 997 (w), 957 (m), 924 (w), 902 (w), 890 (w), 879 (w), 837 (m), 802 (w), 786 (w), 757 (w), 745 (w), 699 (m), 675 (w), 663 (w), 656 (m), 650 (m), 635 (w), 562 (w).

Exact mass calculated for $[M + H]^+$: m/z = 345.2596; measured: m/z = 345.2597.

Ethyl 2-(4,4,5,5-tetrapropyl-1,3,2-dioxaborolan-2-yl)benzoate (9b)

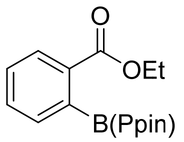

9b

Yield: 379.6 mg (977 µmol, **98%**)

Appearance: colorless solid

Purity$_{HPLC}$: 100%

R_f=0.56 (cyclohexane:EtOAc 4:1)

Melting point: 46.2-47.3 °C (open capillary)

^{1}H NMR (400 MHz, $CDCl_3$) δ 7.93 – 7.86 (m, 1H), 7.53 – 7.44 (m, 2H), 7.44 – 7.33 (m, 1H), 4.36 (q, *J* = 7.1 Hz, 2H), 1.84 (ddd, *J* = 14.0, 12.5, 4.3 Hz, 4H), 1.68 (ddd, *J* = 13.9, 12.0, 4.8 Hz, 4H), 1.53 – 1.25 (m, 11H), 0.97 (t, *J* = 7.3 Hz, 12H).

^{13}C NMR (101 MHz, $CDCl_3$) δ 168.18, 134.44, 132.55, 131.69, 128.91, 128.54, 88.93, 61.22, 36.48, 17.75, 15.00, 14.48.

ATR FT-IR: $\tilde{\nu}$ [cm^{-1}] = 3071 (w), 2957 (s), 2931 (m), 2904 (m), 2870 (m), 1707 (s), 1684 (w), 1601 (w), 1571 (w), 1490 (w), 1471 (w), 1464 (w), 1457 (w), 1436 (w), 1388 (m), 1380 (m), 1367 (m), 1348 (m), 1305 (m), 1296 (m), 1279 (s), 1271 (s), 1259 (m), 1177 (w), 1164 (w), 1144 (m), 1130 (m), 1112 (m), 1064 (m), 1035 (w), 1023 (w), 1011 (w), 954 (m), 917 (w), 908 (w), 879 (w), 841 (w), 813 (w), 748 (w), 736 (m), 700 (m), 688 (w), 675 (w), 656 (m), 648 (w), 593 (w).

Exact mass calculated for $[M + Na]^+$: m/z = 411.2677; measured: m/z = 411.2681.

4,4,5,5-Tetrapropyl-2-(o-tolyl)-1,3,2-dioxaborolane (10b)

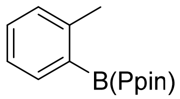

10b

Yield: 163.7 mg (496 µmol, **50%**)

Appearance: colorless oil

$Purity_{HPLC}$: 100%

R_f=0.67 (cyclohexane:EtOAc 4:1)

^{1}H NMR (400 MHz, $CDCl_3$) δ 7.82 – 7.75 (m, 1H), 7.36 – 7.27 (m, 1H), 7.20 – 7.12 (m, 2H), 2.54 (s, 3H), 1.74 – 1.57 (m, 8H), 1.53 – 1.43 (m, 4H), 1.43 – 1.27 (m, 4H), 0.94 (t, *J* = 7.3 Hz, 12H).

^{13}C NMR (101 MHz, $CDCl_3$) δ 145.19, 136.35, 130.91, 129.93, 124.84, 88.26, 37.18, 22.38, 17.90, 14.99.

ATR FT-IR: $\tilde{\nu}$ [cm^{-1}] = 3017 (w), 2959 (s), 2932 (m), 2872 (m), 1601 (m), 1489 (w), 1455 (m), 1440 (m), 1385 (m), 1377 (m), 1365 (m), 1344 (s), 1325 (m), 1310 (m), 1281 (m), 1256 (m), 1178 (w), 1159 (w), 1145 (w), 1134 (w), 1122 (m), 1071 (m), 1043 (m), 955 (w), 908 (w), 840 (w), 830 (w), 760 (w), 728 (m), 672 (w), 658 (m).

2-(2-Methoxyphenyl)-4,4,5,5-tetrapropyl-1,3,2-dioxaborolane (11b)

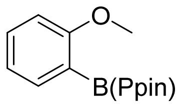

11b

Yield: 318.3 mg (919 µmol, 92 %)

Appearance: colorless oil

$Purity_{HPLC}$: 100%

R_f=0.50 (cyclohexane:EtOAc 4:1)

^{1}H NMR (400 MHz, $CDCl_3$) δ 7.65 (dd, *J* = 7.3, 1.9 Hz, 1H), 7.43 – 7.34 (m, 1H), 6.93 (t, *J* = 7.3 Hz, 1H), 6.85 (d, *J* = 8.3 Hz, 1H), 3.81 (s, 3H), 1.75 – 1.59 (m, 8H), 1.56 – 1.44 (m, 4H), 1.43 – 1.29 (m, 4H), 0.94 (t, *J* = 7.3 Hz, 12H).

^{13}C NMR (101 MHz, $CDCl_3$) δ 164.43, 136.85, 132.43, 120.31, 110.67, 88.25, 55.80, 37.11, 17.82, 15.00.

ATR FT-IR: $\tilde{\nu}$ [cm^{-1}] = 2958 (s), 2932 (m), 2872 (m), 2833 (w), 1600 (m), 1575 (w), 1489 (m), 1455 (m), 1432 (m), 1391 (m), 1349 (s), 1312 (m), 1276 (m), 1245 (m), 1177 (w), 1161 (w), 1145 (w), 1122 (m), 1095 (w), 1072 (m), 1046 (m), 1027 (m), 998 (w), 955 (w), 909 (w), 840 (w), 826 (w), 779 (w), 758 (m), 658 (w), 646 (w).

Exact mass calculated for $[M + Na]^+$: m/z = 369.2571; measured: m/z = 369.2574.

N-(3-(4,4,5,5-Tetrapropyl-1,3,2-dioxaborolan-2-yl)phenyl)acetamide (12b)

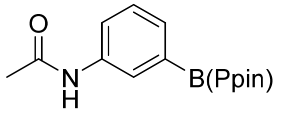

12b

Yield: 339.8 mg (910 µmol, **91%**)

Appearance: colorless solid

$Purity_{HPLC}$: 99%

R_f=0.55 (EtOAc)

Melting point: 119.9-121.7 °C (open capillary)

^1H NMR (600 MHz, $CDCl_3$) δ 7.94 – 7.89 (m, 1H), 7.59 – 7.56 (m, 1H), 7.54 (d, *J* = 7.3 Hz, 1H), 7.37 – 7.32 (m, 1H), 7.19 (s, 1H), 2.16 (s, 3H), 1.70 – 1.58 (m, 8H), 1.52 – 1.41 (m, 4H), 1.39 – 1.26 (m, 4H), 0.93 (t, *J* = 7.3 Hz, 12H).

^{13}C NMR (151 MHz, $CDCl_3$) δ 168.33, 137.44, 130.88, 128.72, 125.83, 123.18, 88.74, 37.19, 24.73, 17.80, 14.96, 14.96.

ATR FT-IR: $\tilde{\nu}$ [cm^{-1}] = 3250 (m), 3154 (w), 3126 (w), 3068 (w), 3051 (w), 3023 (w), 2964 (m), 2932 (m), 2872 (m), 1661 (m), 1608 (w), 1575 (w), 1545 (m), 1507 (w), 1485 (m), 1470 (w), 1457 (m), 1424 (w), 1413 (m), 1390 (m), 1351 (s), 1329 (m), 1303 (m), 1275 (w), 1249 (m), 1221 (m), 1186 (w), 1157 (w), 1145 (w), 1135 (m), 1118 (w), 1101 (w), 1074 (m), 1039 (w), 1016 (w), 998 (w), 957 (m), 918 (w), 902 (w), 890 (w), 841 (w), 806 (w), 796 (w), 743 (w), 712 (w), 703 (m), 669 (w), 642 (w), 606 (w).

Exact mass calculated for $[M + Na]^+$: m/z = 396.2680; measured: m/z = 396.2684.

4,4,5,5-Tetrapropyl-2-(2-(trifluoromethyl)phenyl)-1,3,2-dioxaborolane (13b)

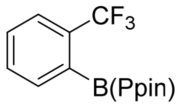

13b

Yield: 242.2 mg (630 µmol, **63%**)

Appearance: colorless oil

Purity$_{HPLC}$: 99%

R_f=0.61 (cyclohexane:EtOAc 4:1)

^{1}H NMR (400 MHz, $CDCl_3$) δ 7.79 – 7.71 (m, 1H), 7.69 – 7.61 (m, 1H), 7.56 – 7.45 (m, 2H), 1.77 – 1.60 (m, 8H), 1.56 – 1.41 (m, 4H), 1.41 – 1.26 (m, 4H), 0.95 (t, J = 7.3 Hz, 12H).

^{13}C NMR (101 MHz, $CDCl_3$) δ 135.55, 130.82, 130.08, 125.39, 89.37, 36.79, 17.70, 14.94.

^{19}F NMR (376 MHz, $CDCl_3$) δ -60.49.

ATR FT-IR: $\tilde{\nu}$ [cm^{-1}] = 2961 (m), 2934 (m), 2875 (m), 1605 (w), 1498 (w), 1466 (w), 1458 (w), 1397 (w), 1371 (m), 1348 (m), 1331 (m), 1314 (s), 1280 (w), 1257 (w), 1161 (m), 1129 (m), 1100 (m), 1073 (w), 1047 (m), 1035 (m), 953 (w), 910 (w), 839 (w), 822 (w), 772 (m), 756 (w), 674 (w), 659 (w), 643 (w), 598 (w).

2-(4-(Benzyloxy)phenyl)-4,4,5,5-tetrapropyl-1,3,2-dioxaborolane (14b)

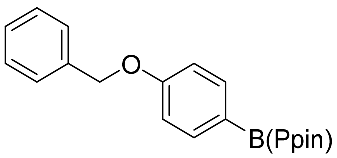

14b

Yield: 394.9 mg (935 µmol, **93%**)

Appearance: colorless oil

Purity$_{HPLC}$: 100%

R_f=0.58 (cyclohexane:EtOAc 4:1)

^{1}H NMR (600 MHz, $CDCl_3$) δ 7.78 – 7.72 (m, 2H), 7.46 – 7.41 (m, 2H), 7.40 – 7.36 (m, 2H), 7.35 – 7.29 (m, 1H), 7.00 – 6.94 (m, 2H), 5.09 (s, 2H), 1.70 – 1.58 (m, 8H), 1.53 – 1.41 (m, 4H), 1.39 – 1.28 (m, 4H), 0.93 (t, J = 7.3 Hz, 12H).

^{13}C NMR (151 MHz, $CDCl_3$) δ 161.39, 137.04, 136.70, 128.73, 128.11, 127.59, 114.32, 88.36, 69.89, 37.22, 17.82, 14.99.

ATR FT-IR: $\tilde{\nu}$ [cm^{-1}] = 3034 (w), 2958 (m), 2931 (m), 2872 (m), 1602 (m), 1570 (w), 1514 (w), 1464 (w), 1455 (m), 1437 (w), 1408 (m), 1399 (m), 1353 (s), 1328 (m), 1311 (m), 1288 (w), 1266 (m), 1240 (m), 1173 (m), 1145 (w), 1134 (w), 1126 (m), 1089 (m), 1037 (w), 1024 (m), 1013 (m), 954 (w), 930 (w), 908 (w), 857 (w), 832 (m), 815 (w), 733 (m), 695 (m), 669 (w), 653 (m).

Exact mass calculated for $[M + H]^+$: m/z = 423.3064; measured: m/z = 423.3067.

2-(3-Nitrophenyl)-4,4,5,5-tetrapropyl-1,3,2-dioxaborolane (15b)

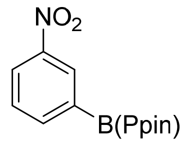

15b

Yield: 340.9 mg (944 µmol, **94%**)

Appearance: colorless solid

$Purity_{HPLC}$: 100%

R_f=0.56 (cyclohexane:EtOAc 4:1)

Melting point: 77.3-80.7 °C (open capillary)

^1H NMR (600 MHz, $CDCl_3$) δ 8.61 (d, *J* = 2.6 Hz, 1H), 8.31 – 8.26 (m, 1H), 8.11 – 8.07 (m, 1H), 7.56 – 7.51 (m, 1H), 1.72 – 1.61 (m, 8H), 1.53 – 1.41 (m, 4H), 1.40 – 1.28 (m, 4H), 0.94 (t, *J* = 7.3 Hz, 12H).

^{13}C NMR (101 MHz, $CDCl_3$) δ 148.04, 140.87, 129.51, 128.87, 125.90, 89.46, 37.18, 17.80, 14.93.

ATR FT-IR: $\tilde{\nu}$ [cm^{-1}] = 3082 (w), 2962 (m), 2931 (m), 2871 (m), 1616 (m), 1529 (m), 1482 (m), 1469 (m), 1456 (w), 1425 (m), 1396 (m), 1368 (m), 1357 (m), 1339 (s), 1320 (m), 1313 (m), 1302 (m), 1287 (m), 1270 (w), 1256 (w), 1184 (w), 1157 (w), 1147 (w), 1133 (w), 1119 (m), 1091 (w), 1072 (w), 1017 (w), 954 (w), 913 (w), 877 (w), 838 (w), 811 (m), 741 (w), 700 (m), 692 (m), 656 (w), 646 (w).

2-(4-Nitrophenyl)-4,4,5,5-tetrapropyl-1,3,2-dioxaborolane (16b)

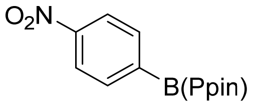

16b

Yield: 321.4 mg (890 µmol, 89 %)

Appearance: colorless solid

Purity$_{HPLC}$: 100%

R_f=0.59 (cyclohexane:EtOAc 4:1)

Melting point: 68.1-70.0 °C (open capillary)

^{1}H NMR (400 MHz, $CDCl_3$) δ 8.19 (d, *J* = 8.7 Hz, 2H), 7.95 (d, *J* = 8.7 Hz, 2H), 1.75 – 1.58 (m, 8H), 1.54 – 1.41 (m, 4H), 1.41 – 1.25 (m, 4H), 0.94 (t, *J* = 7.3 Hz, 12H).

^{13}C NMR (101 MHz, $CDCl_3$) δ 149.96, 135.82, 122.56, 89.49, 37.16, 17.80, 14.93.

ATR FT-IR: $\tilde{\nu}$ [cm^{-1}] = 2959 (m), 2934 (m), 2910 (m), 2874 (m), 2853 (w), 1599 (w), 1518 (m), 1495 (w), 1465 (m), 1436 (w), 1419 (w), 1403 (m), 1377 (m), 1355 (m), 1336 (s), 1319 (m), 1311 (m), 1289 (w), 1270 (m), 1254 (w), 1180 (w), 1146 (w), 1127 (w), 1088 (m), 1018 (w), 947 (w), 914 (w), 879 (w), 856 (m), 840 (w), 810 (w), 746 (w), 702 (m), 657 (w), 646 (m).

Exact mass calculated for $[M + H]^+$: m/z = 362.2497; measured: m/z = 362.2498.

5-(4,4,5,5-Tetrapropyl-1,3,2-dioxaborolan-2-yl)-1H-indole (17b)

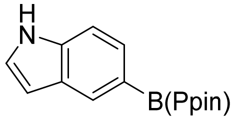

17b

Yield: 293.8 mg (827 µmol, **83%**)

Appearance: colorless solid

Purity$_{HPLC}$: 100%

R_f=0.19 (cyclohexane:EtOAc 4:1)

Melting point: 69.2-71.1 °C (open capillary)

^{1}H NMR (400 MHz, $CDCl_3$) δ 8.18 (s, 2H), 7.65 (d, *J* = 8.2 Hz, 1H), 7.39 (d, *J* = 8.2 Hz, 1H), 7.22 – 7.16 (m, 1H), 6.61 – 6.55 (m, 1H), 1.77 – 1.59 (m, 8H), 1.59 – 1.46 (m, 4H), 1.45 – 1.30 (m, 4H), 0.94 (t, *J* = 7.2 Hz, 12H).

^{13}C NMR (101 MHz, $CDCl_3$) δ 137.91, 128.77, 128.37, 127.73, 124.21, 110.51, 103.31, 88.21, 37.31, 17.86, 15.02.

ATR FT-IR: $\tilde{\nu}$ [cm^{-1}] = 3332 (m), 3049 (w), 2959 (s), 2933 (m), 2872 (m), 1616 (w), 1579 (w), 1517 (w), 1466 (w), 1457 (w), 1424 (m), 1394 (m), 1378 (m), 1362 (m), 1349 (s), 1330 (m), 1313 (m), 1284 (w), 1275 (w), 1251 (m), 1242 (m), 1199 (w), 1187 (w), 1158 (w), 1142 (m), 1134 (m), 1119 (w), 1102 (m), 1070 (m), 1064 (m), 997 (w), 958 (w), 922 (w), 906 (w), 894 (w), 836 (w), 830 (w), 806 (w), 770 (w), 744 (w), 724 (m), 694 (w), 677 (m), 647 (w), 558 (w), 556 (w), 551 (w).

Exact mass calculated for $[M + H]^+$: m/z = 356.2756; measured: m/z = 356.2757.

2-(4-Iodophenyl)-4,4,5,5-tetrapropyl-1,3,2-dioxaborolane (18b)

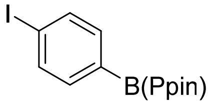

18b

Yield: 349.5 mg (790 µmol, **79%**)

Appearance: colorless oil

R_f=0.71 (cyclohexane:EtOAc 4:1)

^{1}H NMR (400 MHz, $CDCl_3$) δ 7.75 – 7.68 (m, 2H), 7.54 – 7.47 (m, 2H), 1.72 – 1.56 (m, 8H), 1.55 – 1.39 (m, 4H), 1.39 – 1.24 (m, 4H), 0.93 (t, *J* = 7.3 Hz, 12H).

^{13}C NMR (101 MHz, $CDCl_3$) δ 137.04, 136.50, 98.78, 88.86, 37.19, 17.80, 14.96.

ATR FT-IR: $\tilde{\nu}$ [cm^{-1}] = 2958 (s), 2931 (m), 2871 (m), 1586 (m), 1464 (w), 1456 (w), 1397 (m), 1387 (m), 1378 (m), 1352 (s), 1329 (m), 1312 (m), 1297 (w), 1279 (w), 1252 (m), 1179 (w), 1156 (w), 1145 (w), 1135 (w), 1126 (w), 1086 (m), 1006 (m), 952 (w), 908 (w), 838 (w), 815 (m), 749 (w), 720 (m), 662 (w), 647 (m), 627 (w).

2-(2-Chlorophenyl)-4,4,5,5-tetrapropyl-1,3,2-dioxaborolane (19b)

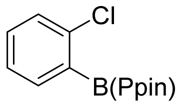

19b

Yield: 331.5 mg (945 µmol, **95%**)

Appearance: pale yellow liquid

Purity$_{HPLC}$: 100%

R_f=0.68 (cyclohexane:EtOAc 4:1)

^{1}H NMR (400 MHz, $CDCl_3$) δ 7.73 – 7.66 (m, 1H), 7.38 – 7.28 (m, 2H), 7.25 – 7.19 (m, 1H), 1.76 – 1.59 (m, 8H), 1.57 – 1.43 (m, 4H), 1.43 – 1.28 (m, 4H), 0.95 (t, *J* = 7.3 Hz, 12H).

^{13}C NMR (101 MHz, $CDCl_3$) δ 140.00, 136.95, 131.95, 129.62, 125.92, 89.01, 37.08, 17.83, 14.97.

ATR FT-IR: $\tilde{\nu}$ [cm^{-1}] = 2959 (s), 2932 (m), 2872 (m), 1593 (m), 1562 (w), 1474 (w), 1466 (m), 1456 (m), 1429 (m), 1391 (m), 1366 (m), 1348 (s), 1328 (m), 1311 (m), 1280 (w), 1268 (m), 1252 (w), 1177 (w), 1158 (w), 1146 (w), 1134 (w), 1120 (w), 1107 (m), 1071 (w), 1040 (m), 953 (w), 909 (w), 839 (w), 818 (w), 755 (m), 734 (w), 710 (w), 665 (w), 652 (w), 641 (w).

4-(4,4,5,5-Tetrapropyl-1,3,2-dioxaborolan-2-yl)phenol (20b)

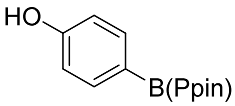

20b

Yield: 331.0 mg (996 µmol, **quant.**)

Appearance: colorless oil

Purity$_{HPLC}$: 99%

R_f=0.21 (cyclohexane:EtOAc 4:1)

^{1}H NMR (400 MHz, $CDCl_3$) δ 7.74 – 7.67 (m, 2H), 6.85 – 6.78 (m, 2H), 1.72 – 1.56 (m, 8H), 1.56 – 1.41 (m, 4H), 1.41 – 1.25 (m, 4H), 0.93 (t, *J* = 7.3 Hz, 12H).

^{13}C NMR (101 MHz, cdcl$_3$) δ 158.26, 136.96, 114.86, 88.39, 37.21, 17.81, 14.99.

ATR FT-IR: $\tilde{\nu}$ [cm^{-1}] = 3428 (w), 3345 (m), 2958 (s), 2931 (m), 2872 (m), 1607 (m), 1584 (m), 1459 (m), 1444 (w), 1435 (w), 1427 (w), 1401 (m), 1375 (m), 1353 (m), 1318 (m), 1307 (m), 1260 (m), 1254 (m), 1212 (m), 1176 (m), 1146 (w), 1126 (m), 1101 (w), 1085 (m), 1014 (w), 996 (w), 970 (w), 949 (m), 911 (m), 879 (w), 833 (m), 822 (w), 752 (w), 733 (w), 728 (w), 694 (w), 691 (w), 669 (w), 653 (m), 636 (w), 596 (w), 585 (w), 557 (w).

2-Phenyl-4,4,5,5-tetrapropyl-1,3,2-dioxaborolane (21b)

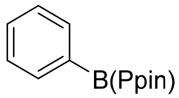

21b

Yield: 162.7 mg (514 µmol, **51%**)

Appearance: colorless solid

Purity$_{HPLC}$: 100%

R_f=0.64 (cyclohexane:EtOAc 4:1)

Melting point: 66.7-71.5 °C (open capillary)

^{1}H NMR (400 MHz, $CDCl_3$) δ 7.84 – 7.77 (m, 2H), 7.48 – 7.42 (m, 1H), 7.40 – 7.32 (m, 2H), 1.74 – 1.58 (m, 8H), 1.54 – 1.42 (m, 4H), 1.42 – 1.25 (m, 4H), 0.94 (t, *J* = 7.2 Hz, 12H).

^{13}C NMR (101 MHz, $CDCl_3$) δ 134.95, 131.22, 127.81, 88.57, 37.22, 17.82, 14.98.

ATR FT-IR: $\tilde{\nu}$ [cm^{-1}] = 3080 (w), 3058 (w), 3041 (w), 3031 (w), 2957 (s), 2931 (m), 2872 (m), 1604 (w), 1497 (w), 1466 (w), 1457 (w), 1439 (m), 1399 (m), 1376 (m), 1353 (s), 1329 (m), 1310 (m), 1277 (w), 1264 (m), 1254 (m), 1178 (w), 1146 (w), 1128 (m), 1102 (w), 1089 (m), 1066 (w), 1028 (w), 951 (m), 914 (w), 879 (w), 839 (w), 814 (w), 755 (w), 745 (w), 699 (m), 669 (w), 653 (m).

2-(2-Bromophenyl)-4,4,5,5-tetrapropyl-1,3,2-dioxaborolane (22b)

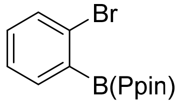

22b

Yield: 369.6 mg (935 µmol, **94%**)

Appearance: yellowish oil

Purity$_{HPLC}$: 100%

R_f=0.63 (cyclohexane:EtOAc 4:1)

^{1}H NMR (400 MHz, $CDCl_3$) δ 7.65 (dd, *J* = 6.9, 2.4 Hz, 1H), 7.54 (dd, *J* = 7.6, 1.5 Hz, 1H), 7.31 – 7.21 (m, 2H), 1.75 – 1.61 (m, 8H), 1.58 – 1.44 (m, 4H), 1.43 – 1.28 (m, 4H), 0.95 (t, *J* = 7.2 Hz, 12H).

^{13}C NMR (101 MHz, $CDCl_3$) δ 137.08, 132.94, 132.02, 128.54, 126.41, 89.16, 37.05, 17.85, 14.97.

ATR FT-IR: $\tilde{\nu}$ [cm^{-1}] = 2959 (s), 2931 (m), 2872 (m), 1588 (m), 1558 (w), 1472 (m), 1465 (m), 1456 (m), 1425 (m), 1391 (m), 1365 (m), 1348 (s), 1327 (m), 1311 (m), 1279 (w), 1267 (m), 1250 (w), 1177 (w), 1158 (w), 1146 (w), 1134 (w), 1128 (w), 1115 (m), 1104 (m), 1071 (w), 1037 (m), 1024 (m), 952 (w), 929 (w), 908 (w), 880 (w), 839 (w), 817 (w), 753 (m), 728 (m), 687 (w), 651 (w), 637 (w).

1-(4-(4,4,5,5-Tetrapropyl-1,3,2-dioxaborolan-2-yl)phenyl)ethan-1-one (23b)

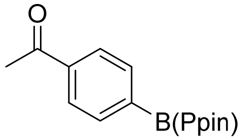

23b

Yield: 352.4 mg (983 µmol, **98%**)

Appearance: colorless oil

Purity$_{HPLC}$: 100%

R_f=0.40 (cyclohexane:EtOAc 4:1)

^{1}H NMR (400 MHz, $CDCl_3$) δ 7.96 – 7.85 (m, 4H), 2.61 (s, 3H), 1.74 – 1.58 (m, 8H), 1.55 – 1.41 (m, 4H), 1.41 – 1.24 (m, 4H), 0.94 (t, *J* = 7.2 Hz, 12H).

^{13}C NMR (101 MHz, $CDCl_3$) δ 198.61, 139.07, 135.11, 127.42, 89.05, 37.20, 26.91, 17.81, 14.95.

ATR FT-IR: $\tilde{\nu}$ [cm^{-1}] = 2959 (m), 2932 (m), 2873 (m), 1687 (m), 1557 (w), 1507 (m), 1465 (w), 1456 (w), 1434 (w), 1399 (m), 1351 (s), 1334 (m), 1307 (m), 1263 (m), 1182 (w), 1157 (w),

1146 (w), 1135 (w), 1127 (w), 1092 (m), 1067 (w), 1018 (m), 952 (m), 909 (w), 833 (w), 821 (w), 747 (w), 742 (w), 730 (w), 668 (w), 653 (m), 602 (w), 593 (w).

Exact mass calculated for $[M + H]^+$: m/z = 359.2752; measured: m/z = 359.2757.

Preparation of Compound 81

The peptide **81** was synthesized on solid support using 0.1 mmol Fmoc-Thr preloaded resin (loading: 0.78 mmol/g) in an automated microwave-assisted peptide synthesizer (Initiator+ Alstra, Biotage). Coupling reactions were performed with 3.5 eq. of the respective Fmoc-protected amino acid (0.2 M), 3.5 eq. of PyBop (0.5 M), and 7 eq. of DIPEA in DMF for 5 min at 75 °C, except both Fmoc-Cys(Acm), which were coupled for 1h at room temperature. Fmoc deprotection was achieved using 20% piperidine in DMF two times for 10 minutes. After Fmoc-D-Phe coupling disulfide bond formation was carried out by oxidation with 10 eq. of iodine in DMF for 1 hour at room temperature. Subsequently, Fmoc-Cys(Trt)-OH was coupled under standard conditions, followed by Fmoc deprotection. The resulting free N-terminus was subsequently acetylated using 10 equivalents of acetic anhydride and 20 equivalents of DIPEA in CH_2Cl_2 for 30 min. The peptide was then cleaved from the resin and globally deprotected using a cleavage cocktail consisting of 95% trifluoroacetic acid (TFA), 2.5% triisopropylsilane (TIPS), and 2.5% water for 2 hours at room temperature. After removal of all volatiles under a gentle stream of nitrogen, the crude product was precipitated by addition into cold diethyl ether and stored overnight at 4 °C. The precipitated peptide was collected by centrifugation and washed with cold diethyl ether. After semi-preparative RP-HPLC (Aeris Peptide XB-C18, 5 µm, 100 Å, 250x21,2 mm) lyophilization yielded 68 mg (52%) of the target peptide as a white solid (yield calculated for the TFA-salt).

^{1}H, ^{13}C and ^{19}F NMR Spectra of EPin Compounds

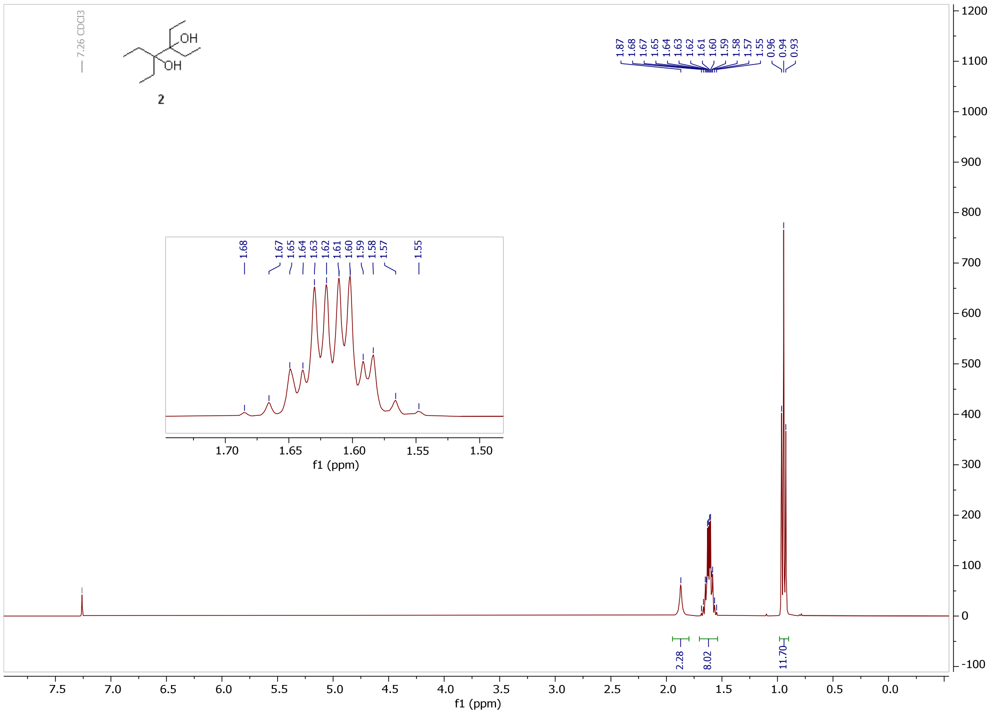

Figure S1: ^{1}H NMR spectrum of compound **2** in $CDCl_3$.

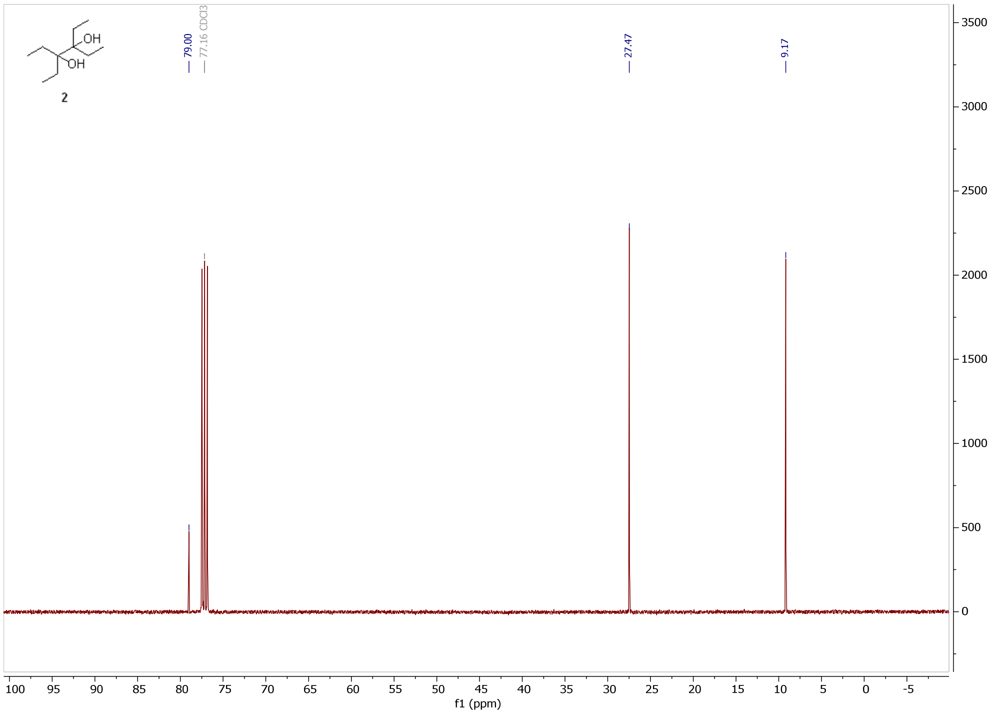

Figure S2: ^{13}C NMR spectrum of compound **2** in $CDCl_3$.

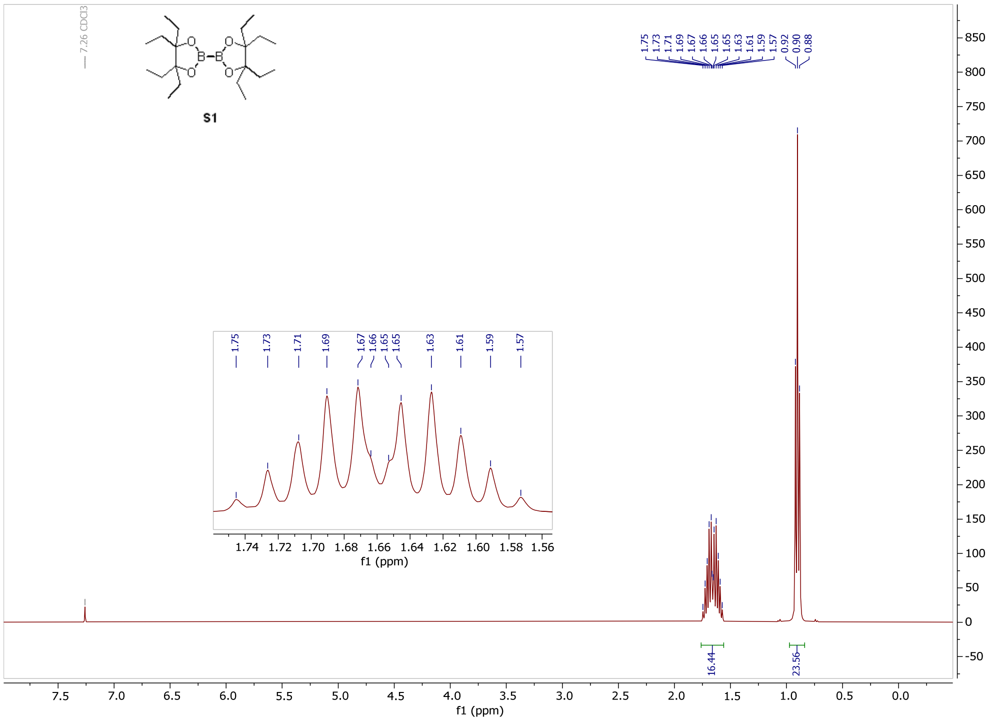

Figure S3: ^{1}H NMR spectrum of compound **S1** in $CDCl_3$.

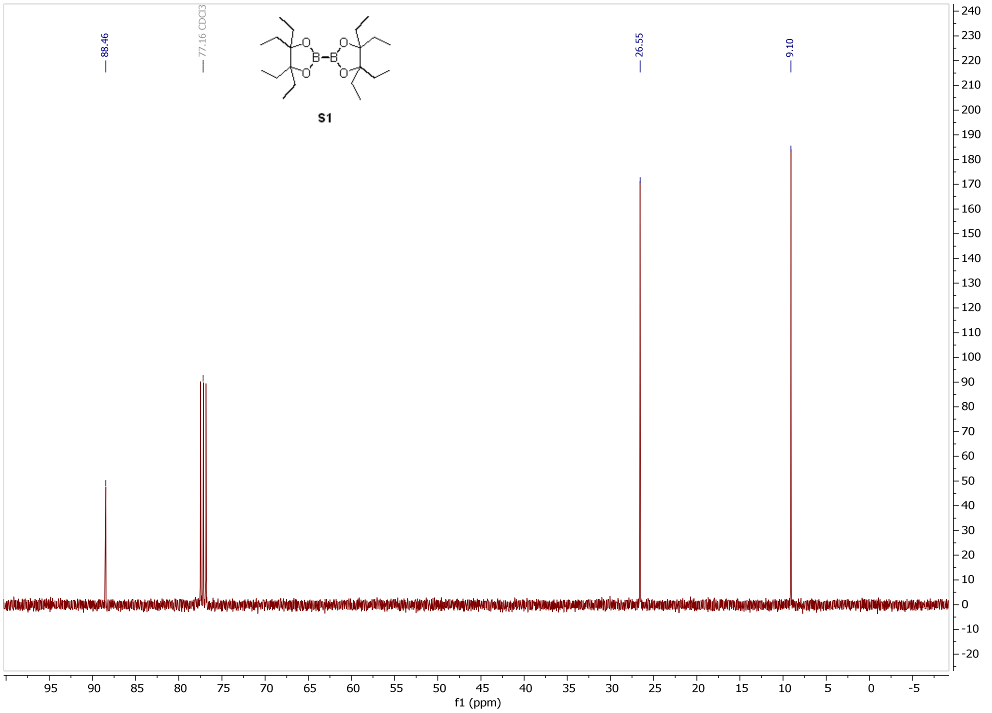

Figure S4: ^{13}C NMR spectrum of compound **S1** in $CDCl_3$.

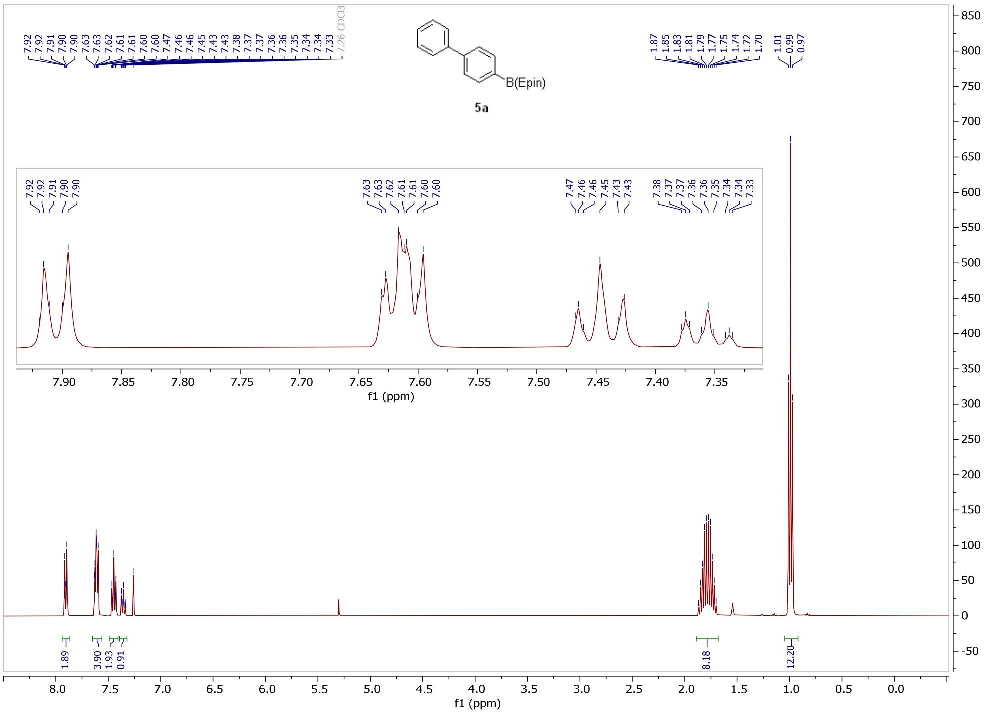

Figure S5: ^{1}H NMR spectrum of compound **5a** in $CDCl_3$.

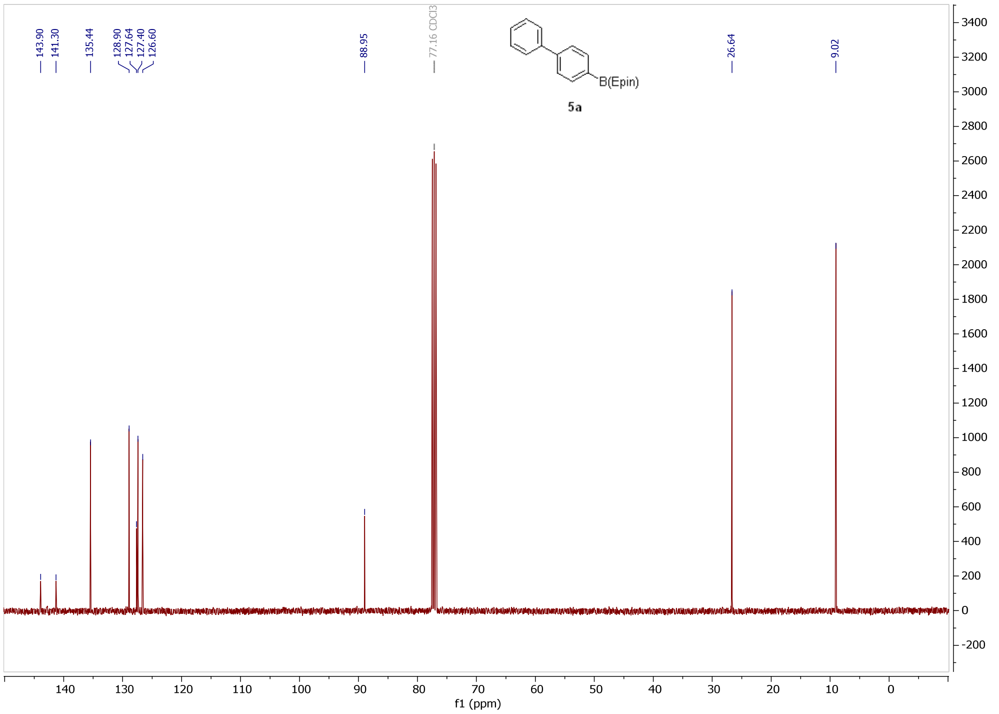

Figure S6: ^{13}C NMR spectrum of compound **5a** in $CDCl_3$.

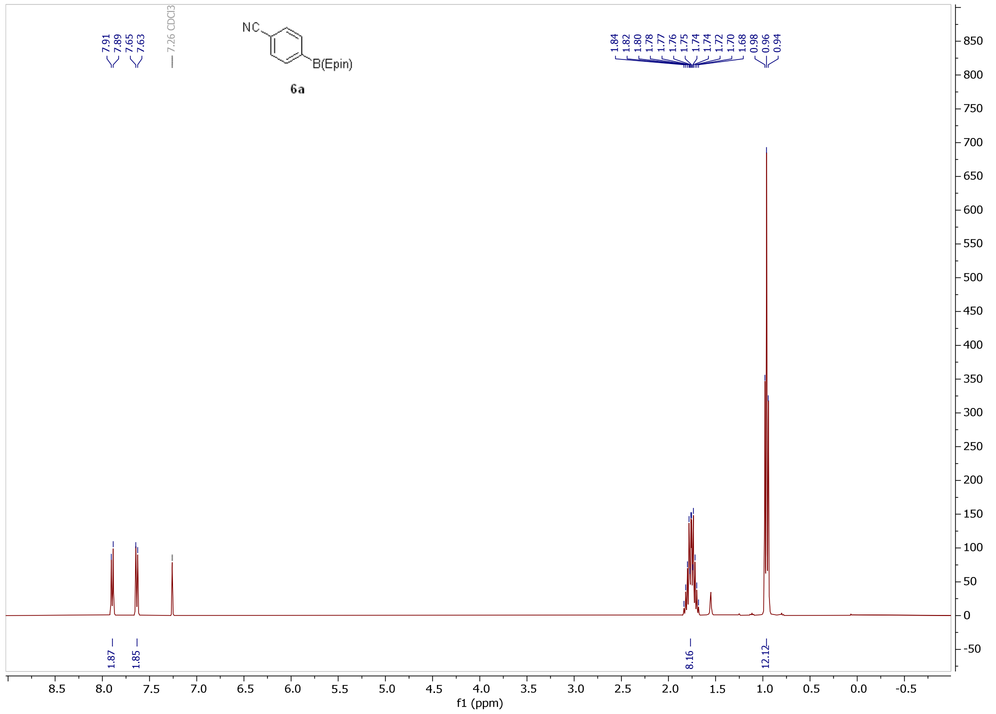

Figure S7: ^{1}H NMR spectrum of compound **6a** in $CDCl_3$.

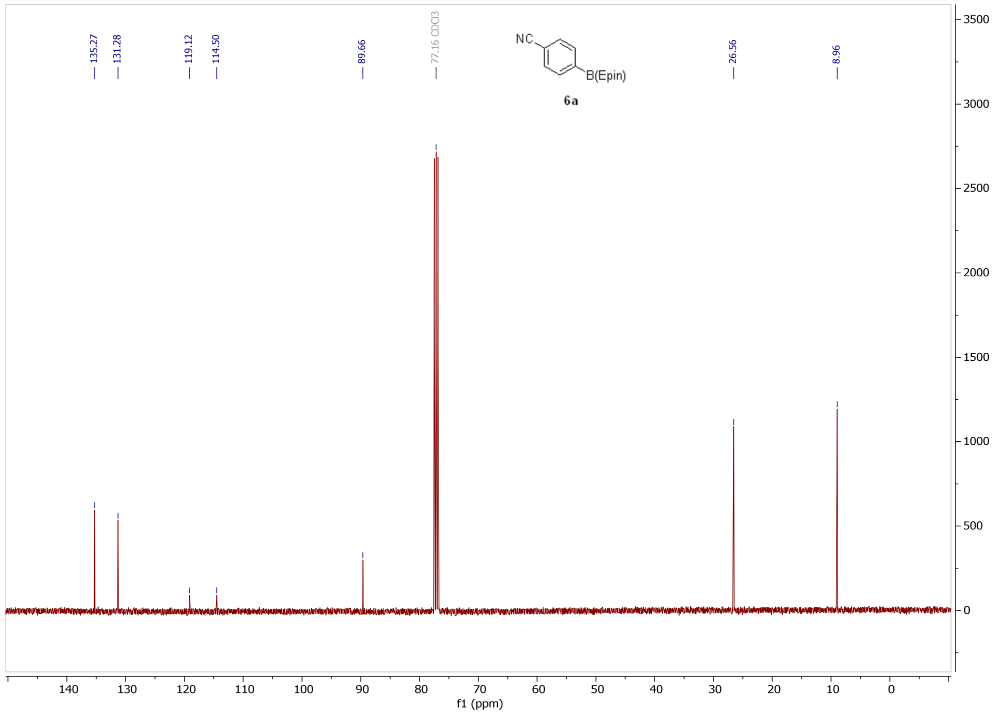

Figure S8: ^{13}C NMR spectrum of compound **6a** in $CDCl_3$.

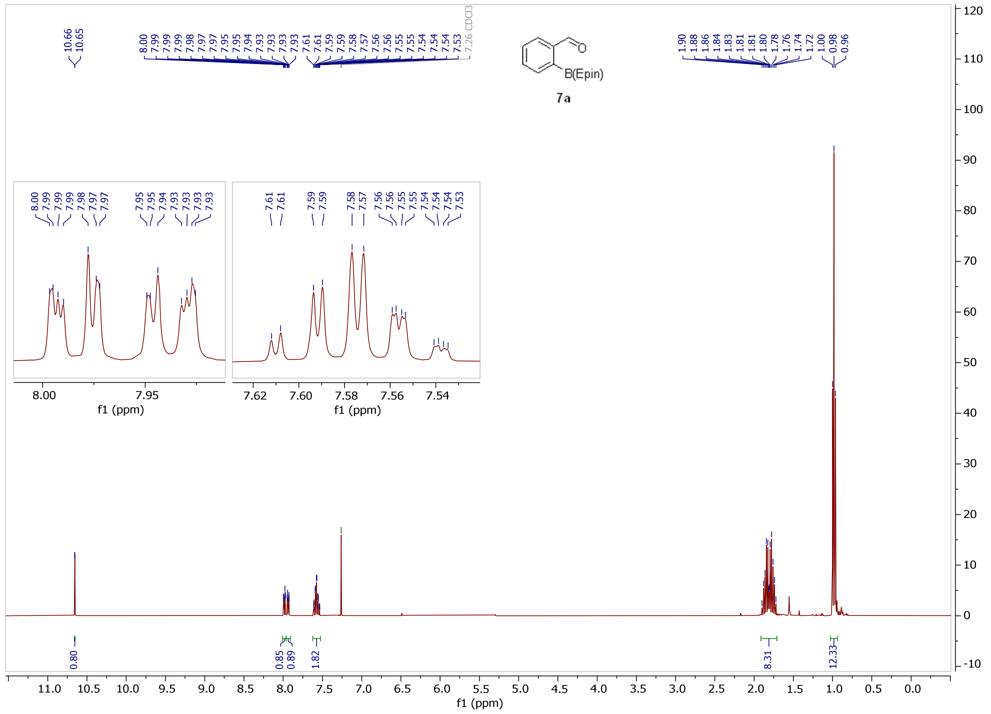

Figure S9: ^{1}H NMR spectrum of compound **7a** in $CDCl_3$.

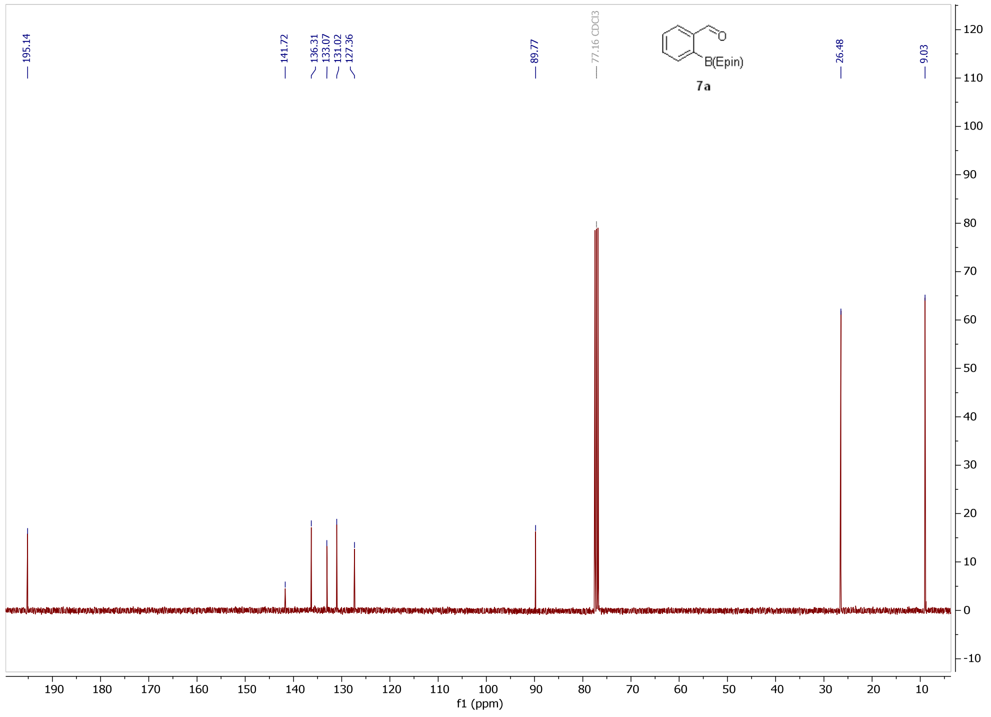

Figure S10: ^{13}C NMR spectrum of compound **7a** in $CDCl_3$.

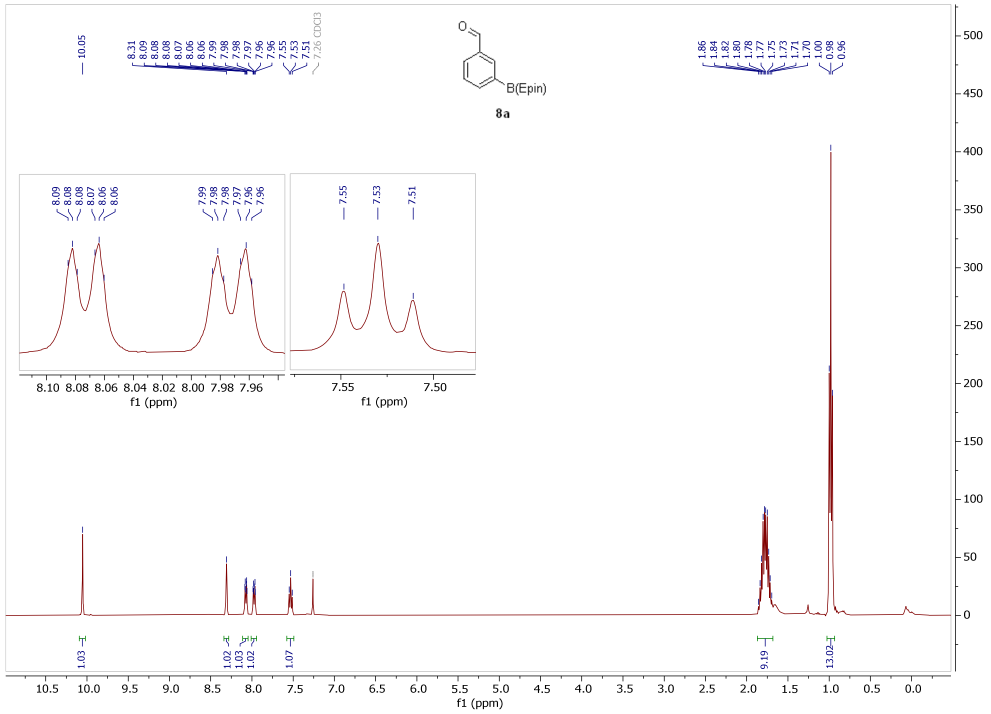

Figure S11: ^{1}H NMR spectrum of compound **8a** in $CDCl_3$.

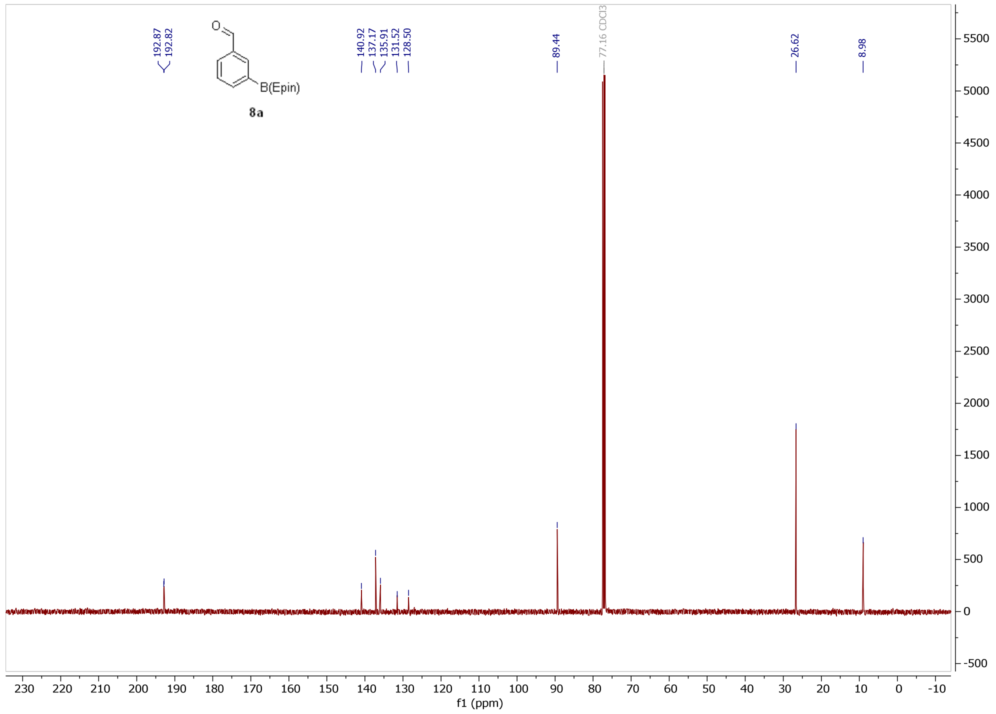

Figure S12: ^{13}C NMR spectrum of compound **8a** in $CDCl_3$.

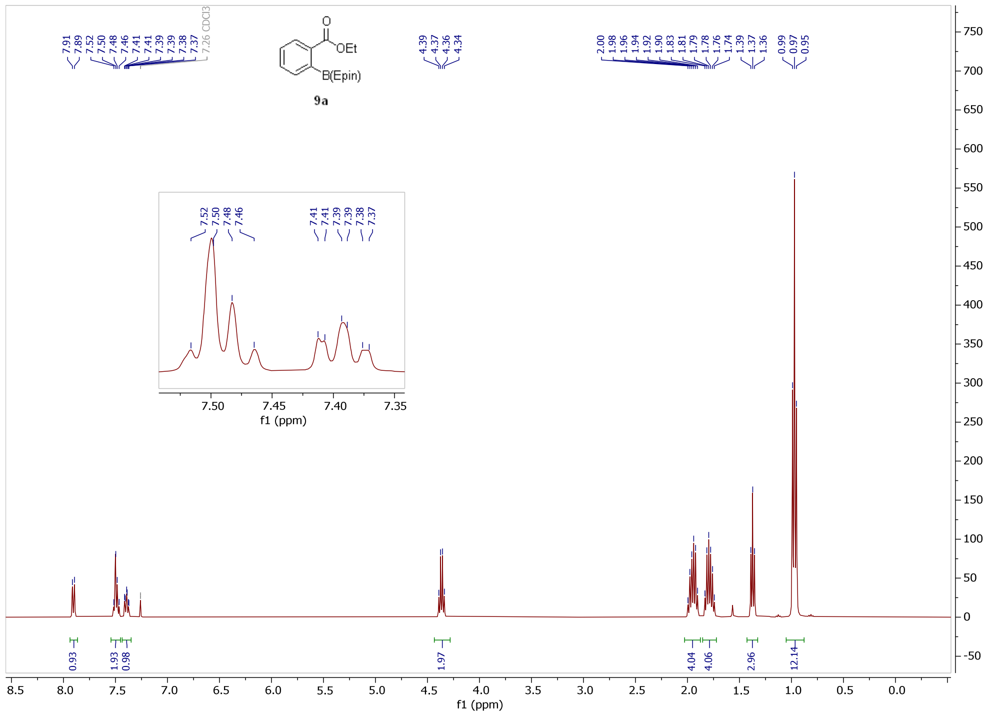

Figure S13: ^{1}H NMR spectrum of compound **9a** in $CDCl_3$.

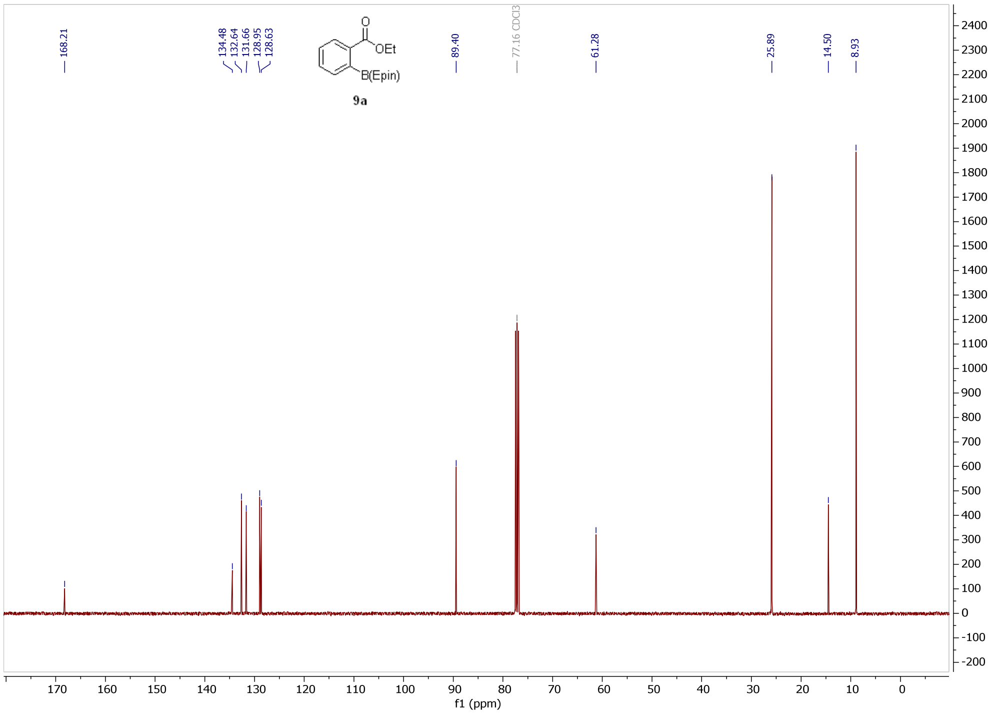

Figure S14: ^{13}C NMR spectrum of compound **9a** in $CDCl_3$.

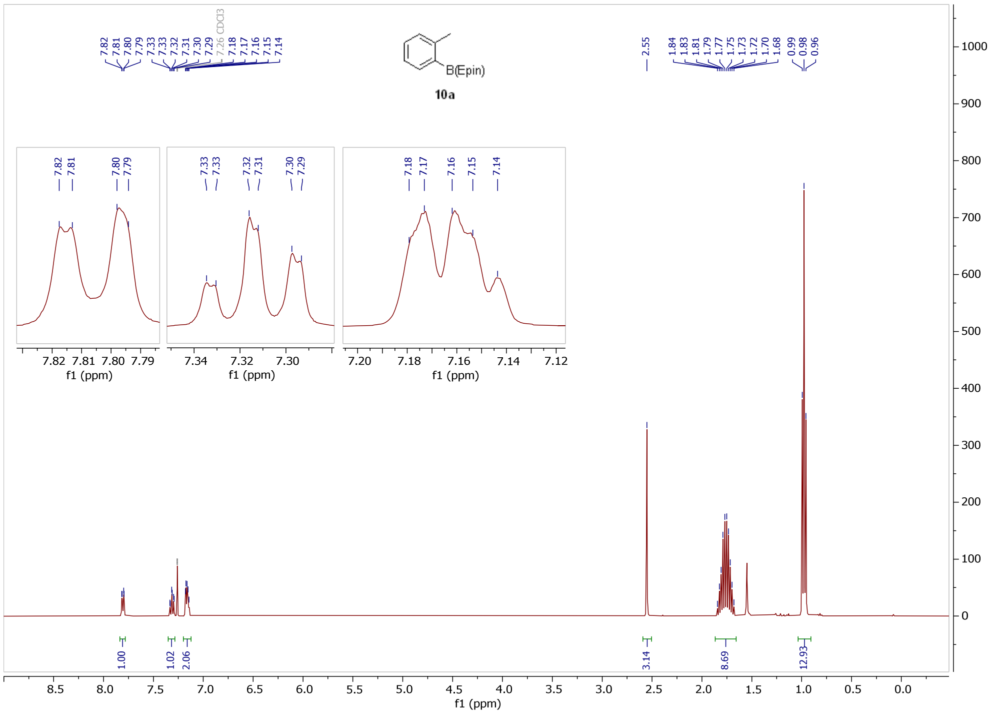

Figure S15: ^{1}H NMR spectrum of compound **10a** in $CDCl_3$.

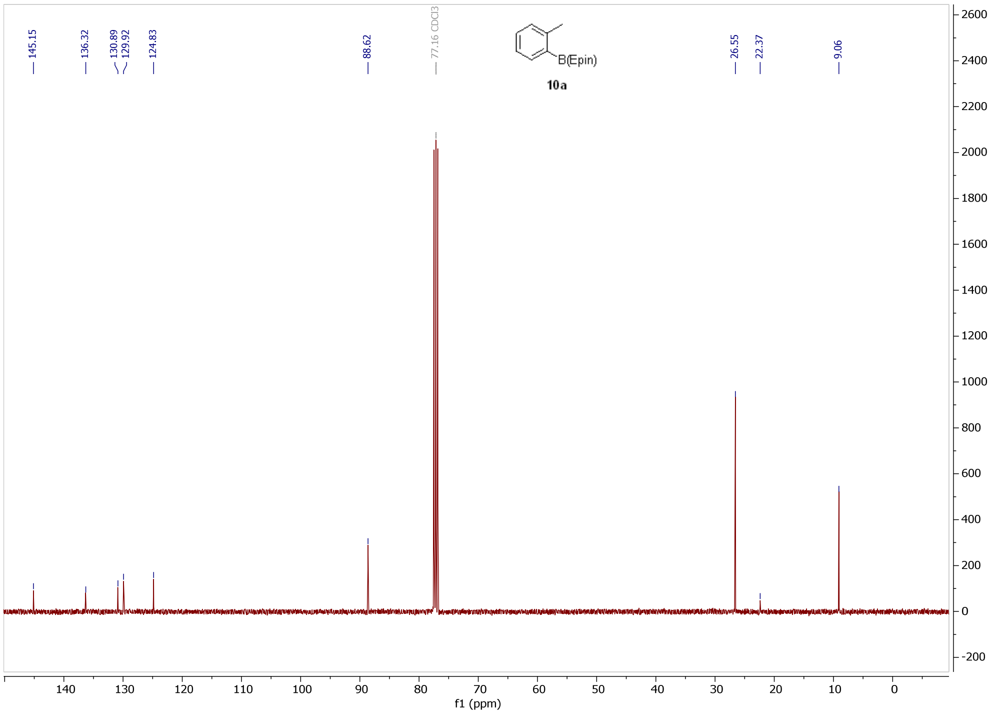

Figure S16: ^{13}C NMR spectrum of compound **10a** in $CDCl_3$.

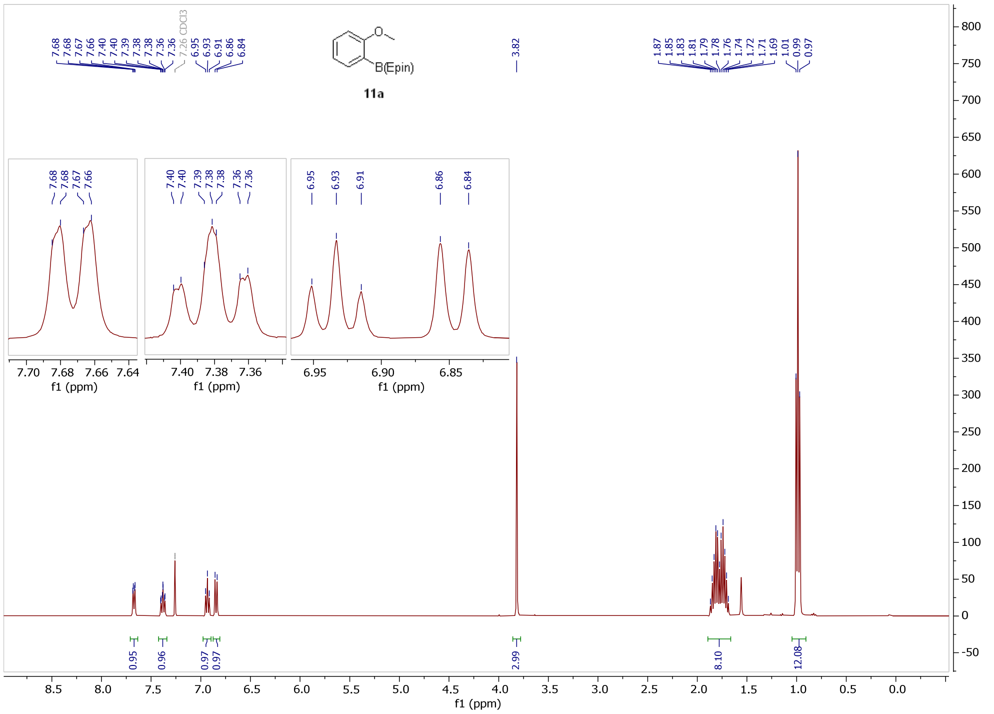

Figure S17: ^{1}H NMR spectrum of compound **11a** in $CDCl_3$.

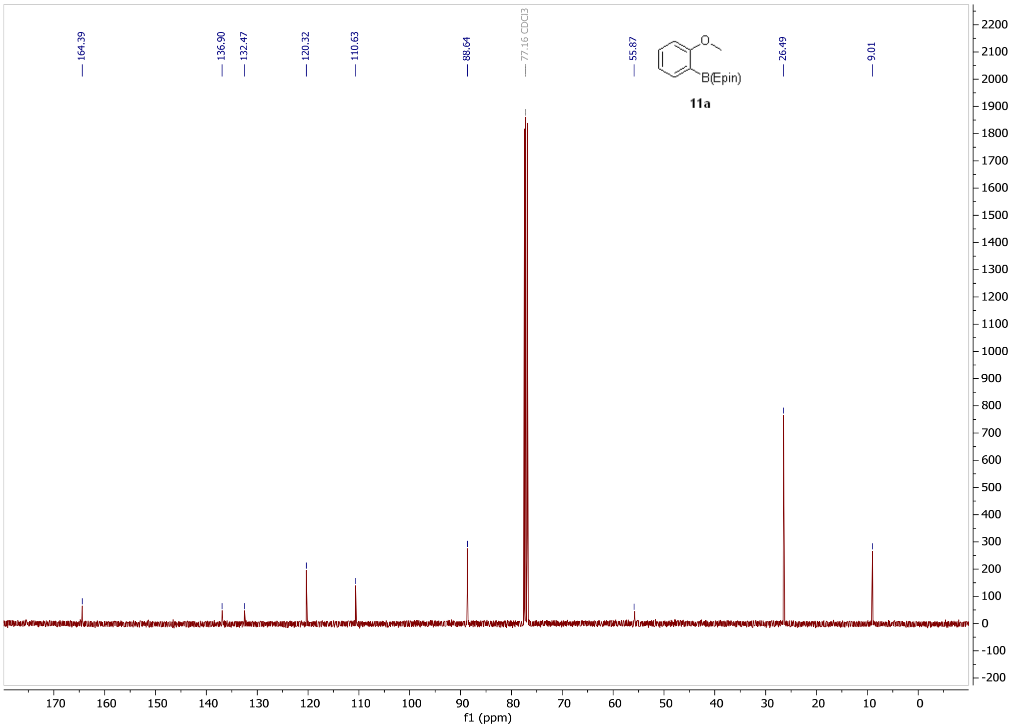

Figure S18: ^{13}C NMR spectrum of compound **11a** in $CDCl_3$.

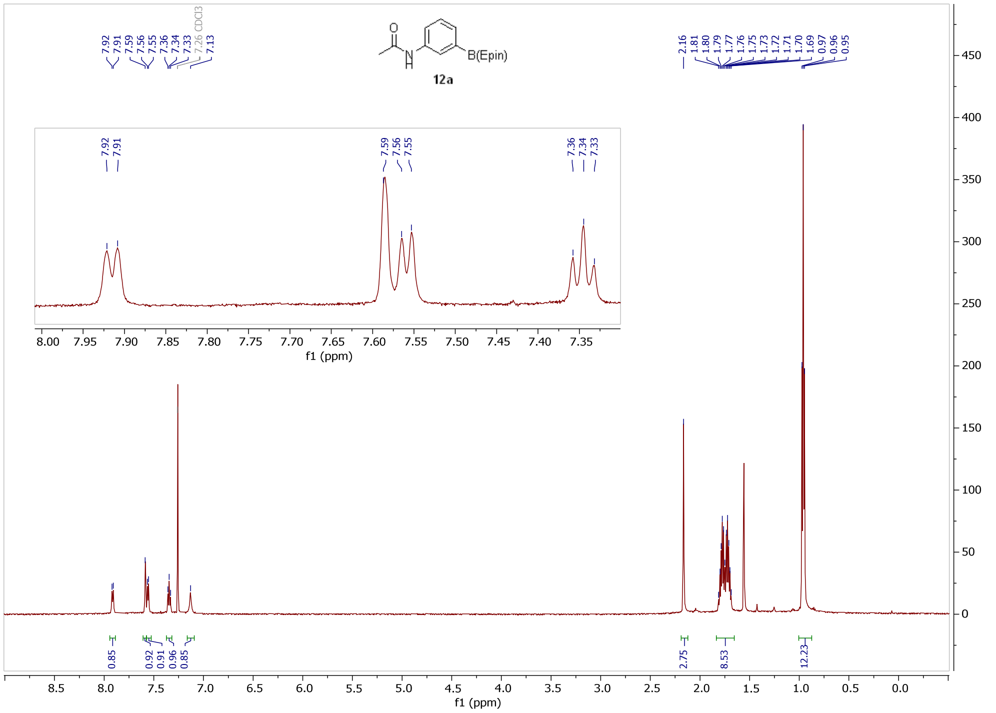

Figure S19: ^{1}H NMR spectrum of compound **12a** in $CDCl_3$.

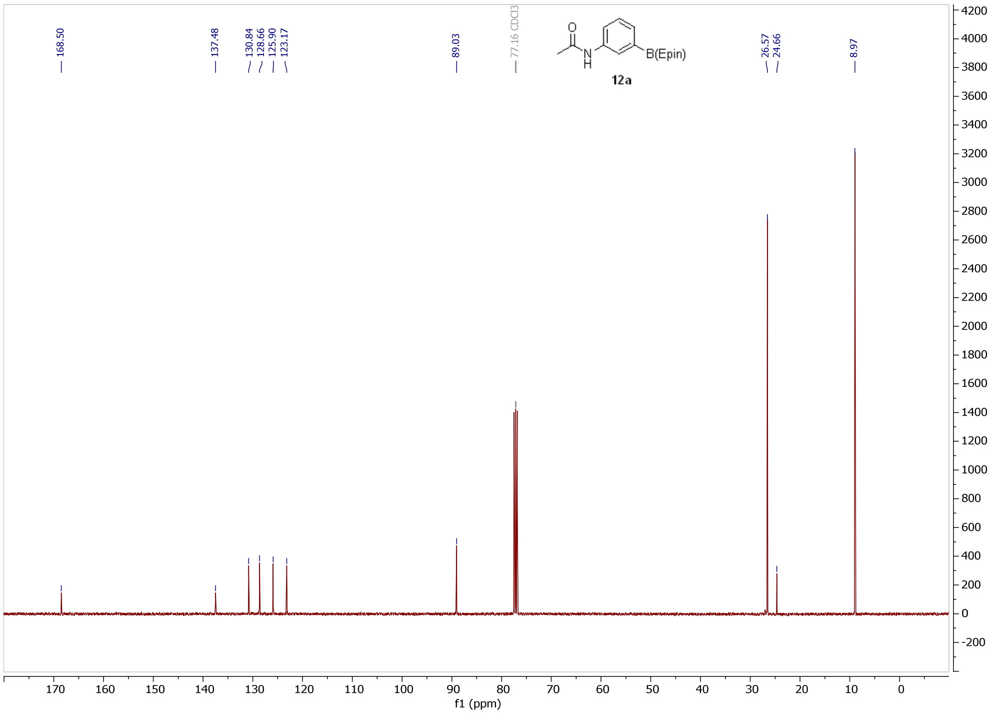

Figure S20: ^{1}H NMR spectrum of compound **12a** in $CDCl_3$.

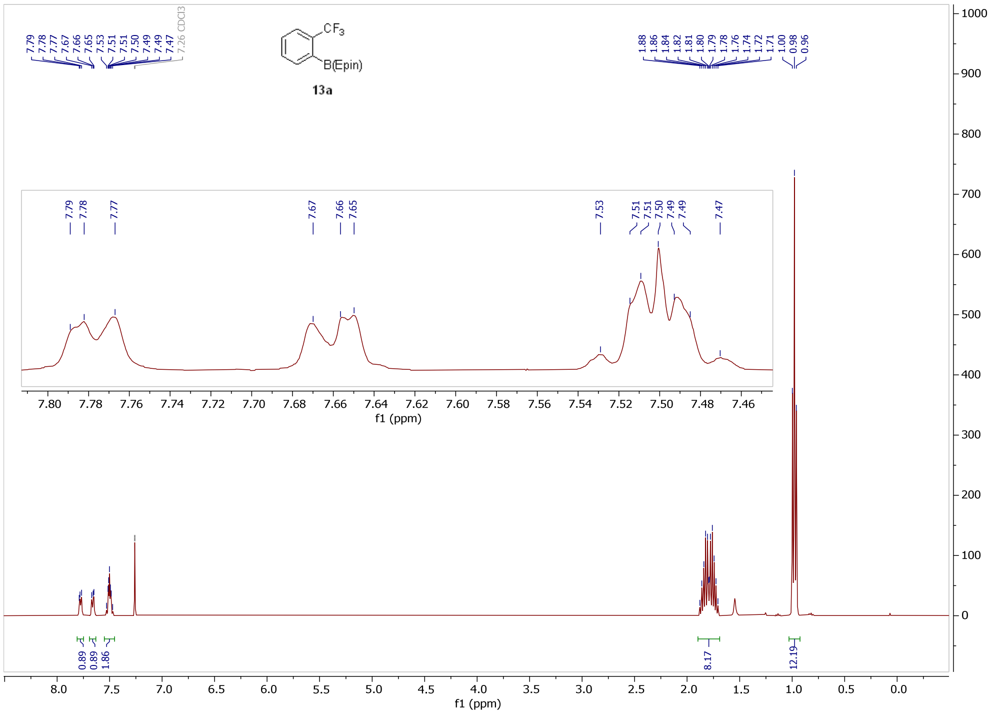

Figure S21: ^{1}H NMR spectrum of compound **13a** in $CDCl_3$.

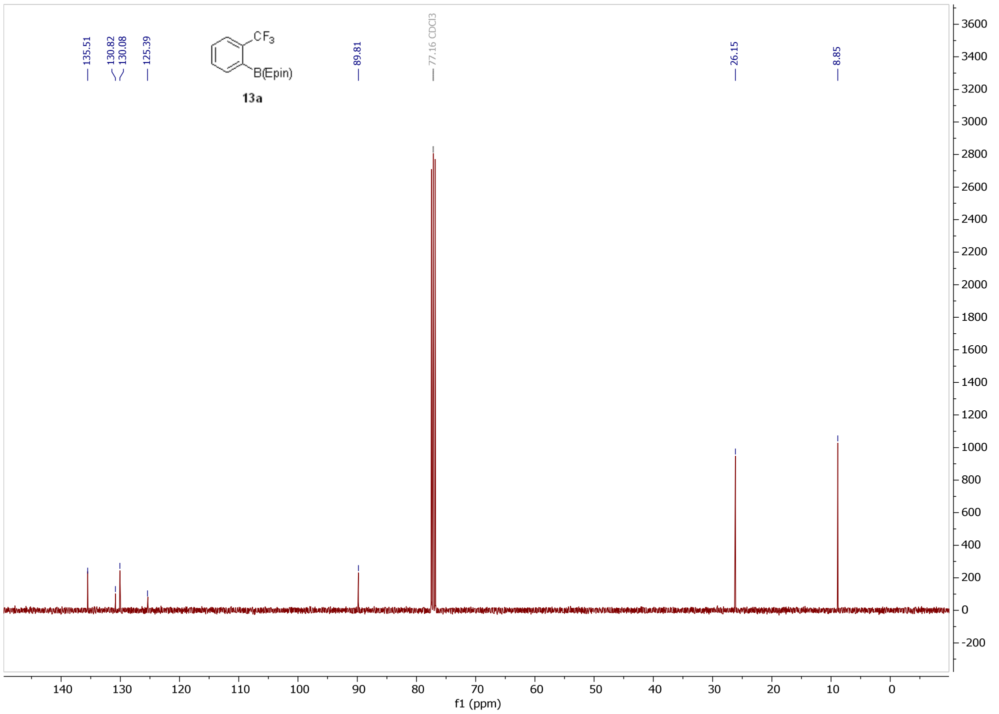

Figure S22: ^{13}C NMR spectrum of compound **13a** in $CDCl_3$.

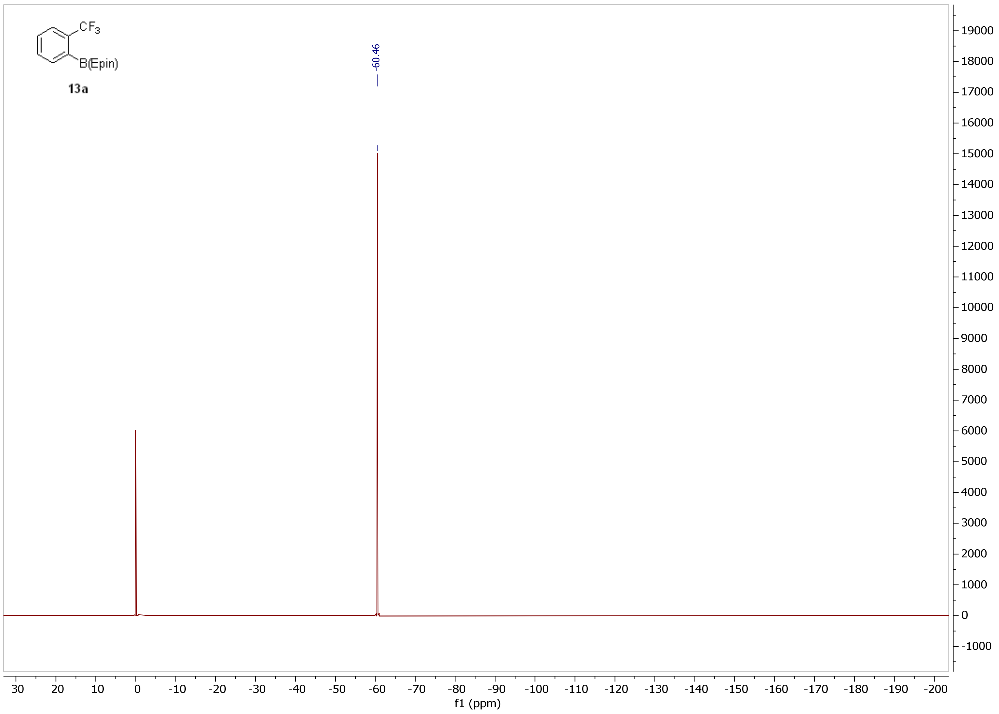

Figure S23: ^{19}F NMR spectrum of compound **13a** in $CDCl_3$.

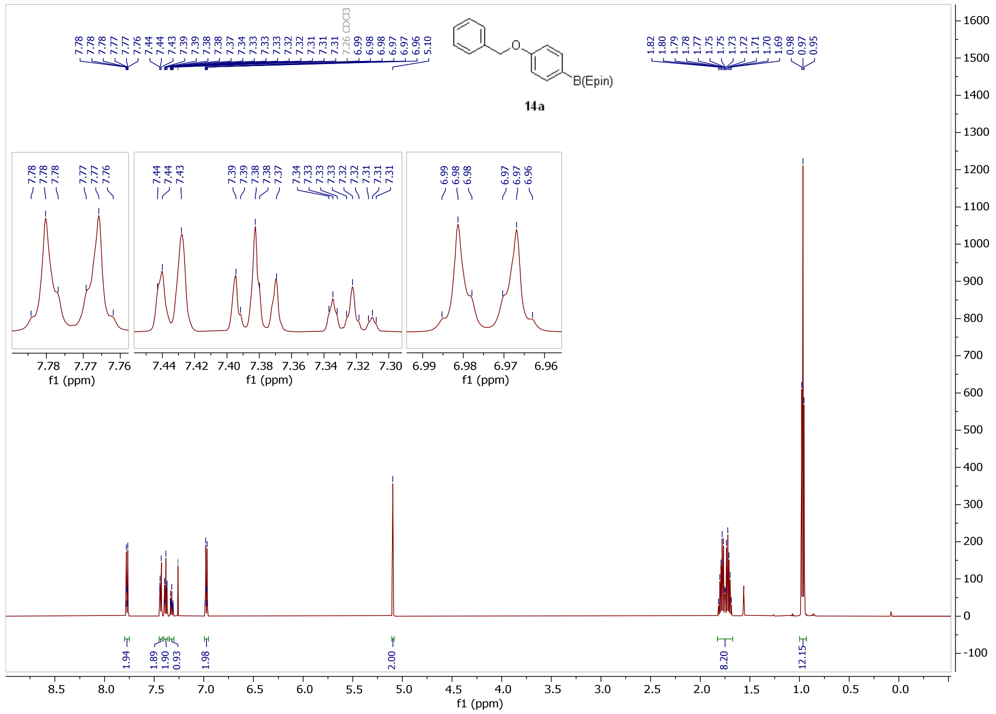

Figure S24: ^{1}H NMR spectrum of compound **14a** in $CDCl_3$.

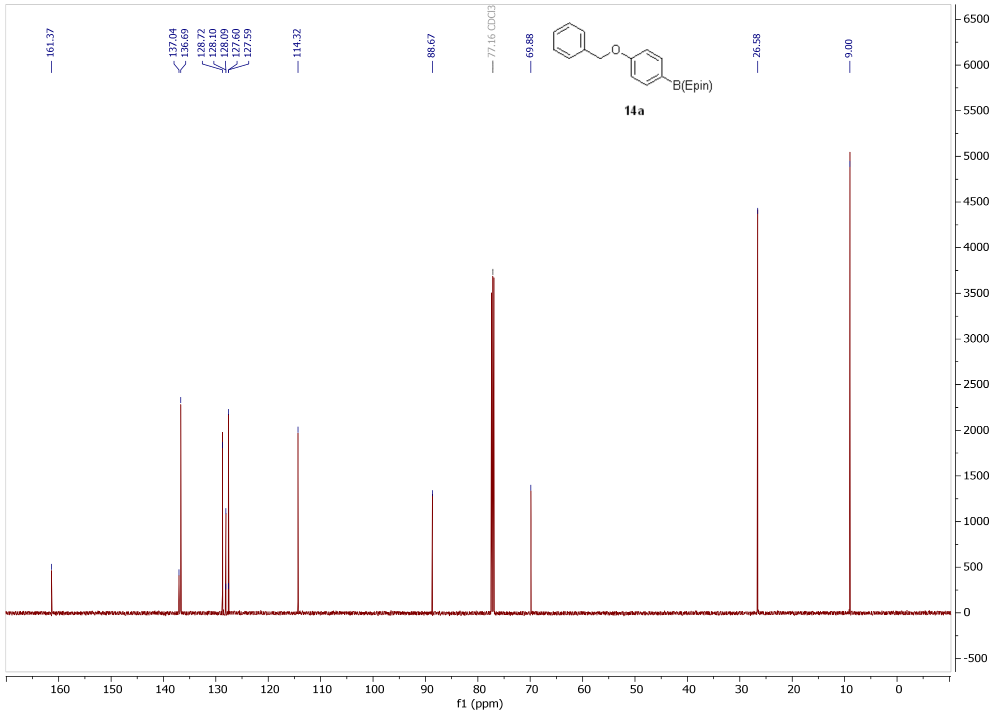

Figure S25: ^{13}C NMR spectrum of compound **14a** in $CDCl_3$.

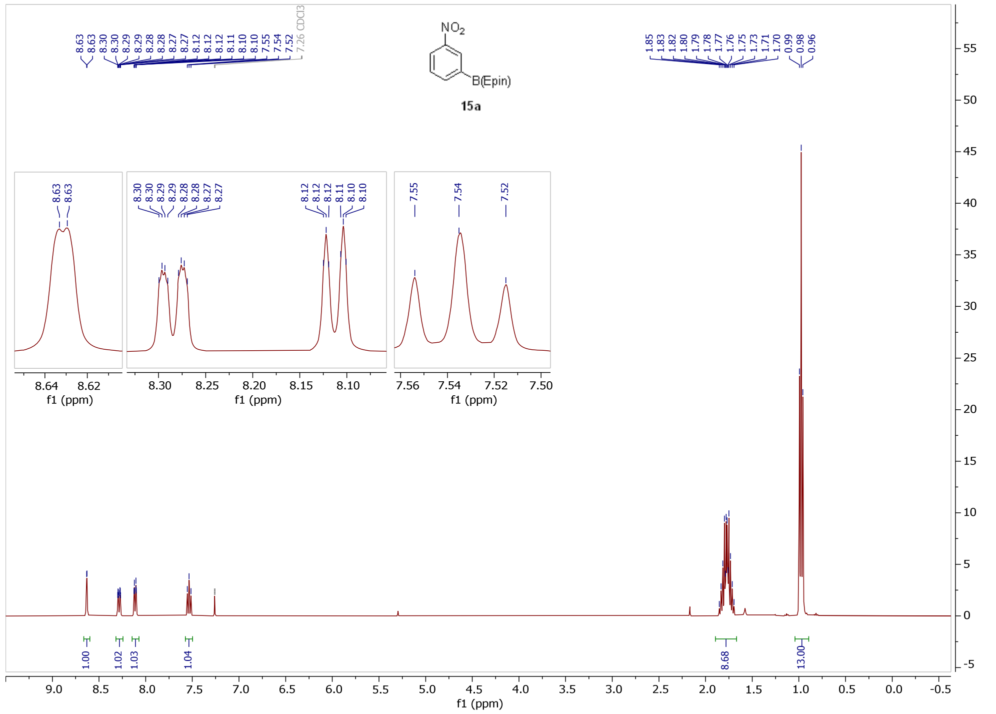

Figure S26: ^{1}H NMR spectrum of compound **15a** in $CDCl_3$.

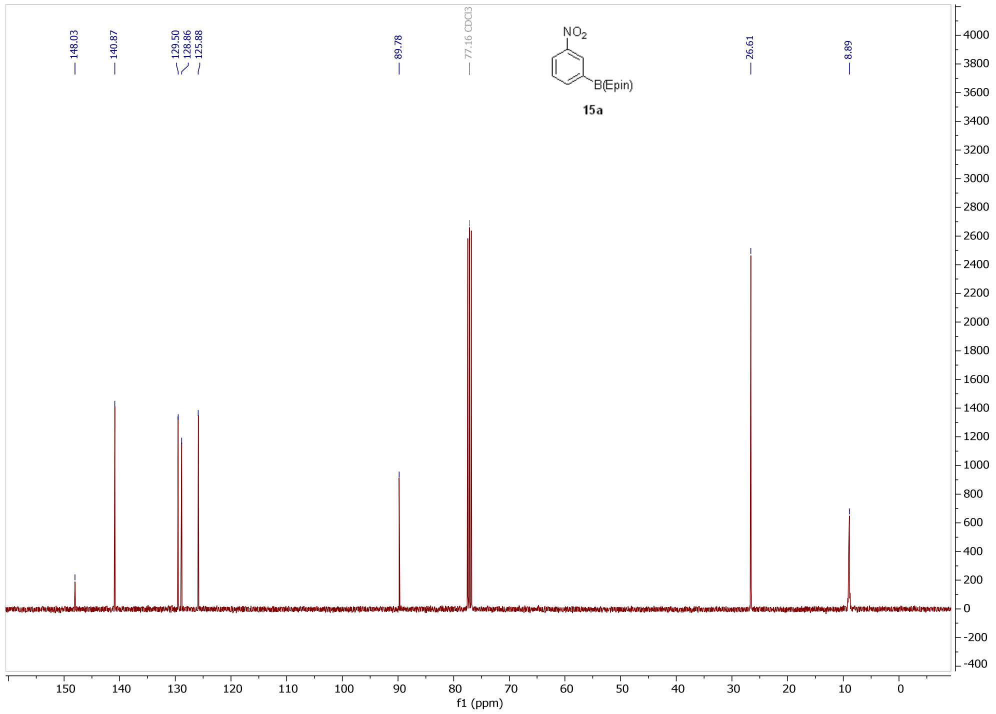

Figure S27: ^{1}H NMR spectrum of compound **15a** in $CDCl_3$.

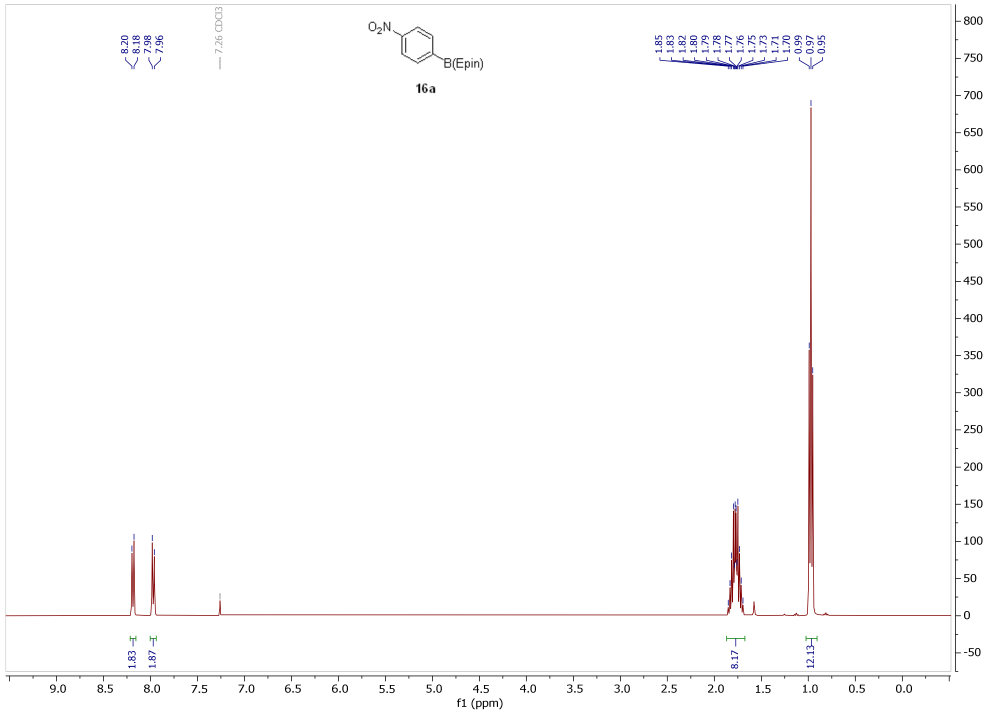

Figure S28: ^{1}H NMR spectrum of compound **16a** in $CDCl_3$.

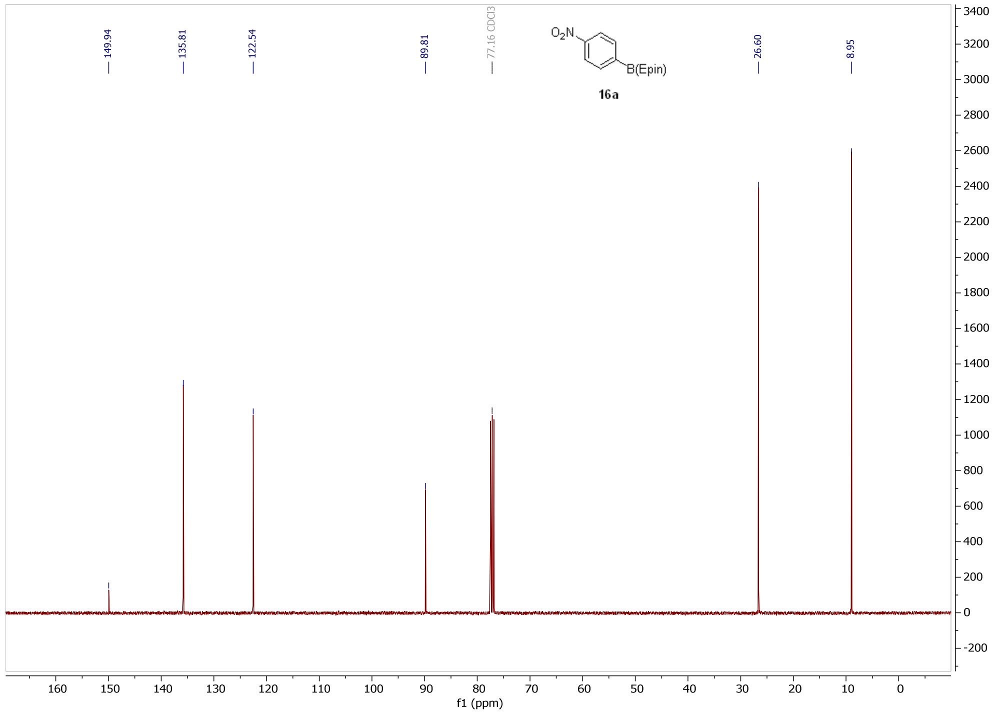

Figure S29: ^{13}C NMR spectrum of compound **16a** in $CDCl_3$.

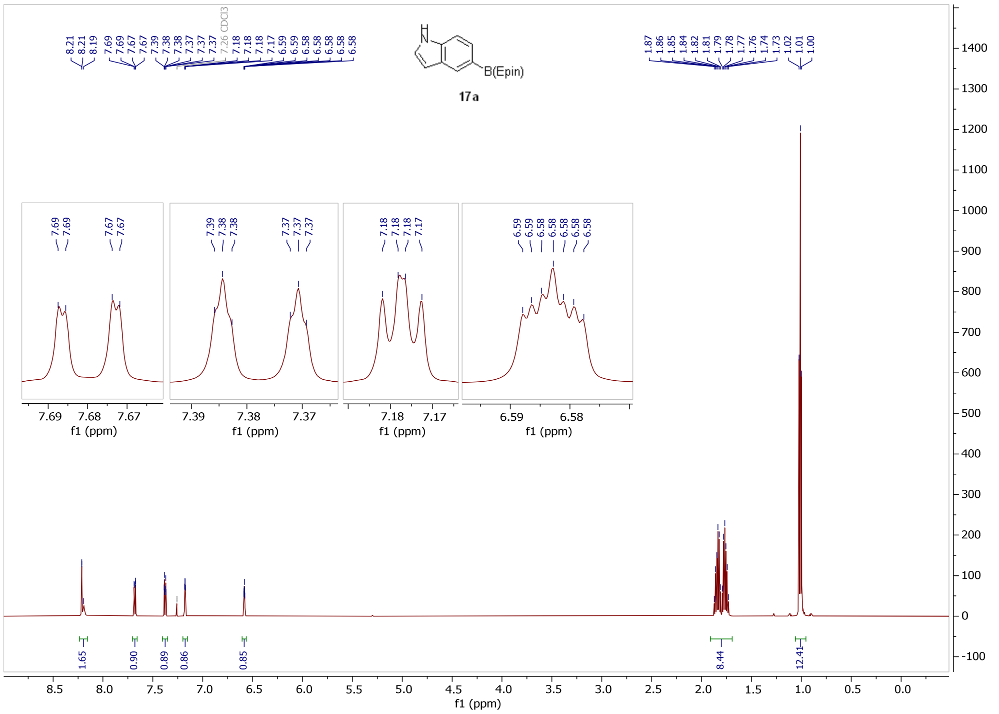

Figure S30: ^{1}H NMR spectrum of compound **17a** in $CDCl_3$.

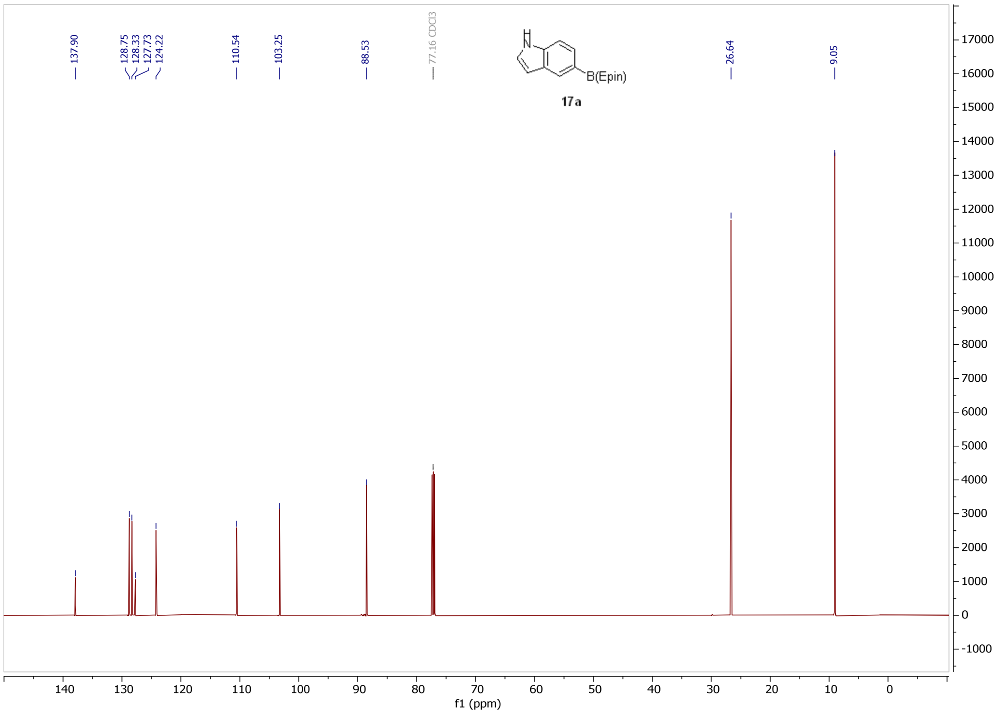

Figure S31: ^{13}C NMR spectrum of compound **17a** in $CDCl_3$.

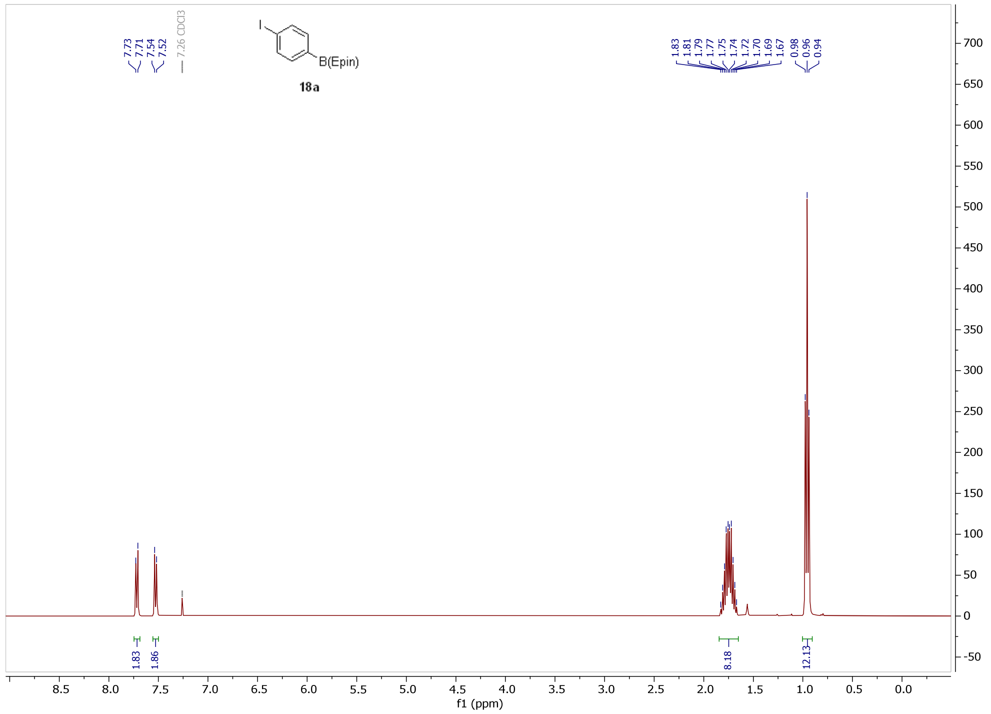

Figure S32: ^{1}H NMR spectrum of compound **18a** in $CDCl_3$.

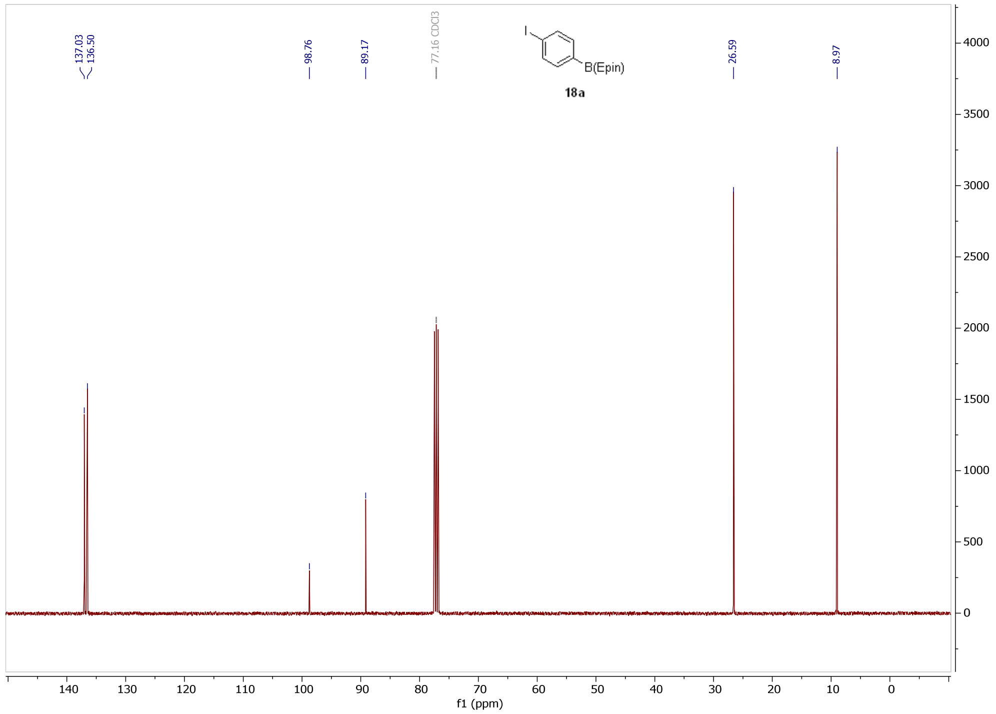

Figure S33: ^{13}C NMR spectrum of compound **18a** in $CDCl_3$.

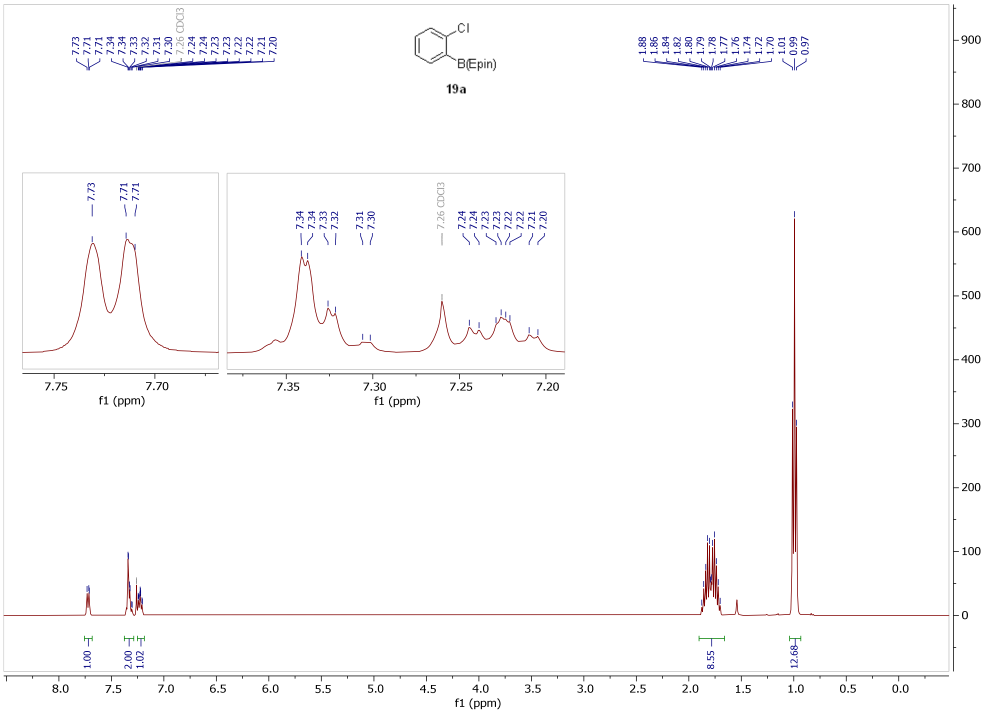

Figure S34: ^{1}H NMR spectrum of compound **19a** in $CDCl_3$.

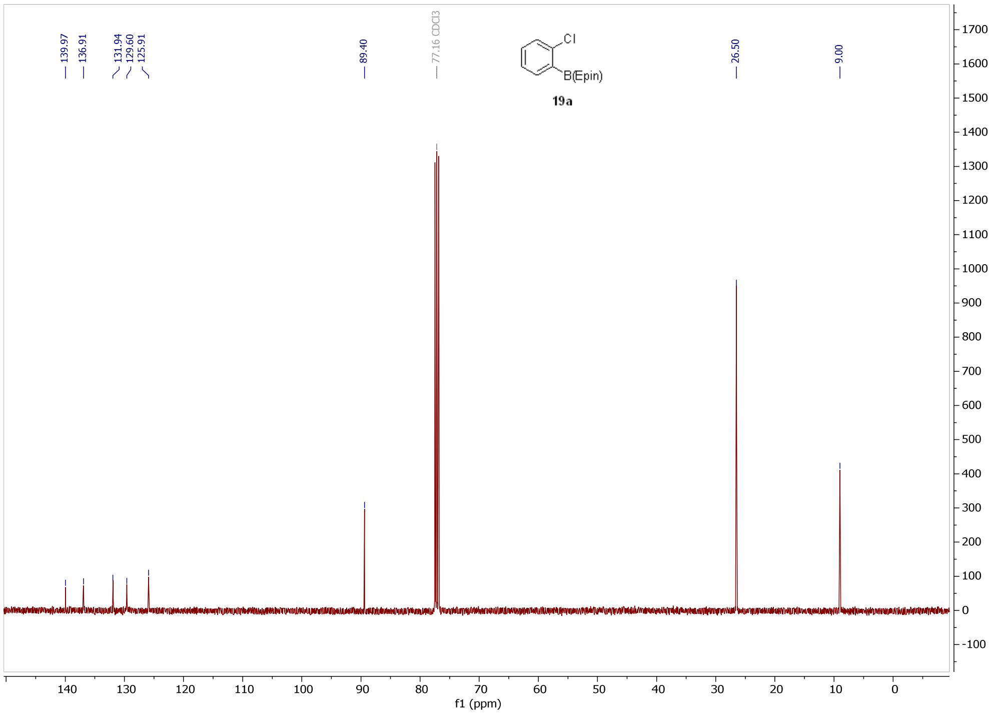

Figure S35: ^{13}C NMR spectrum of compound **19a** in $CDCl_3$.

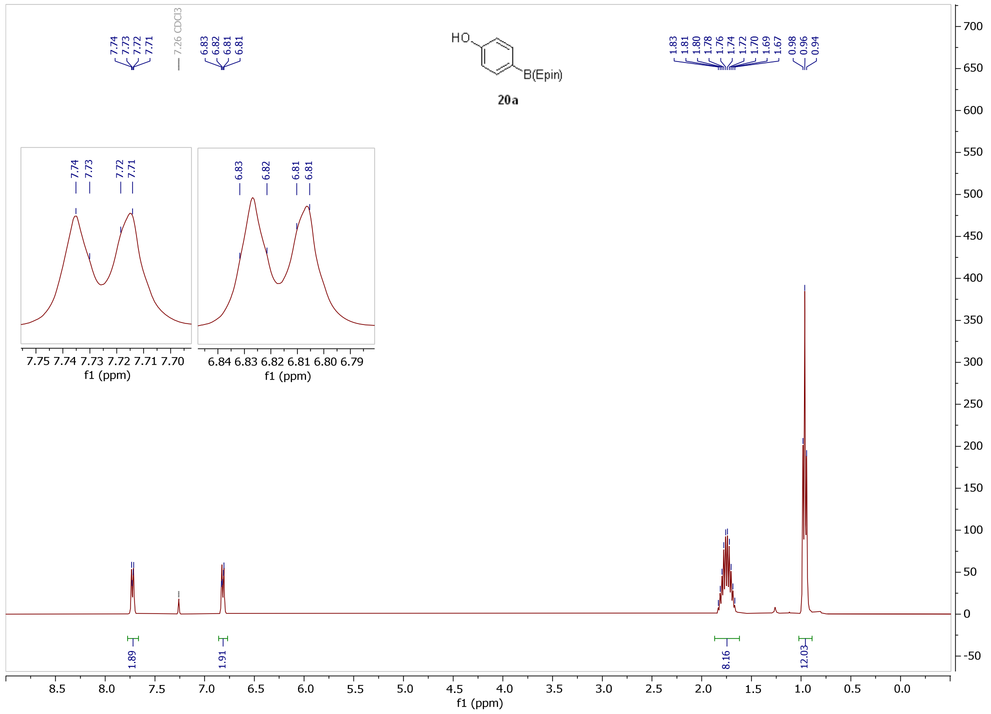

Figure S36: ^{1}H NMR spectrum of compound **20a** in $CDCl_3$.

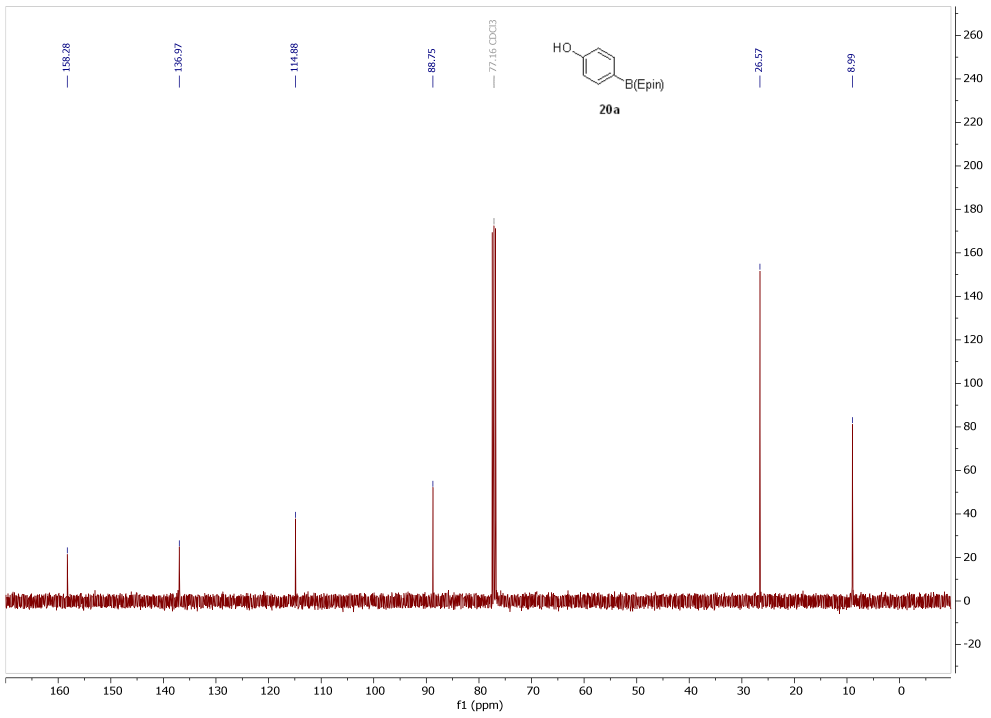

Figure S37: ^{13}C NMR spectrum of compound 2**0a** in $CDCl_3$.

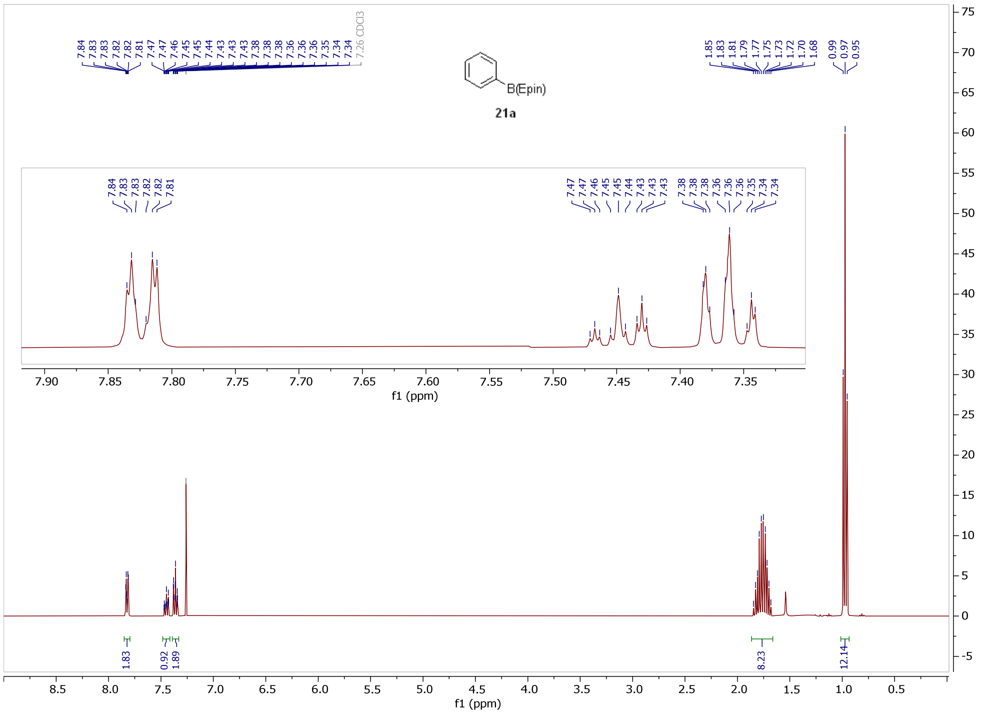

Figure S38: ^{1}H-NMR spectrum of compound **21a** in $CDCl_3$.

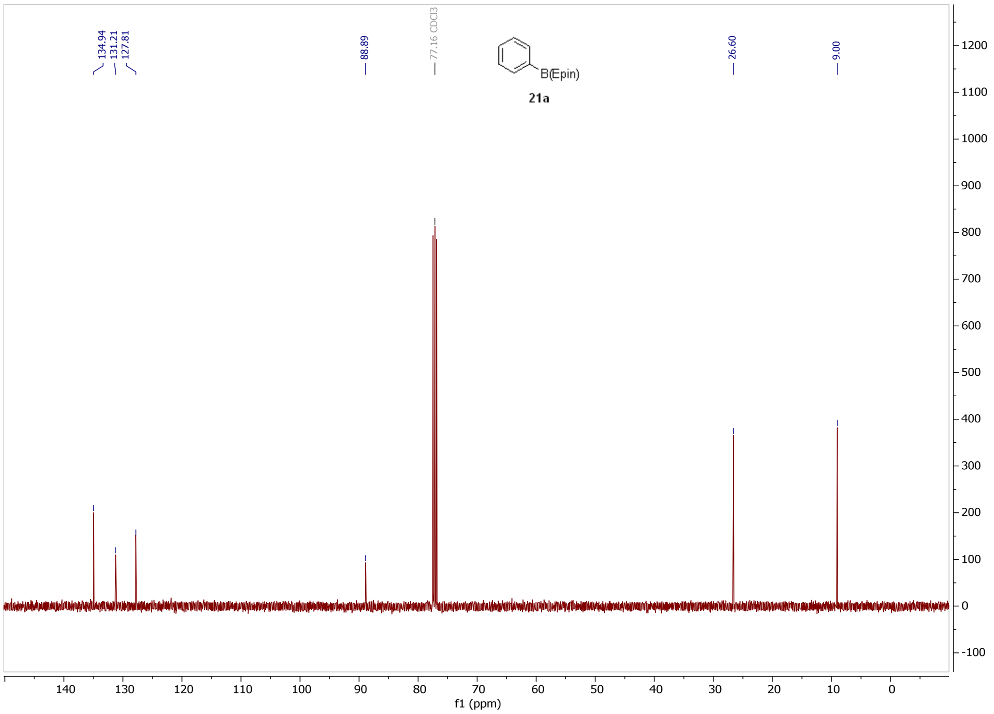

Figure S39: ^{13}C NMR spectrum of compound **21a** in $CDCl_3$.

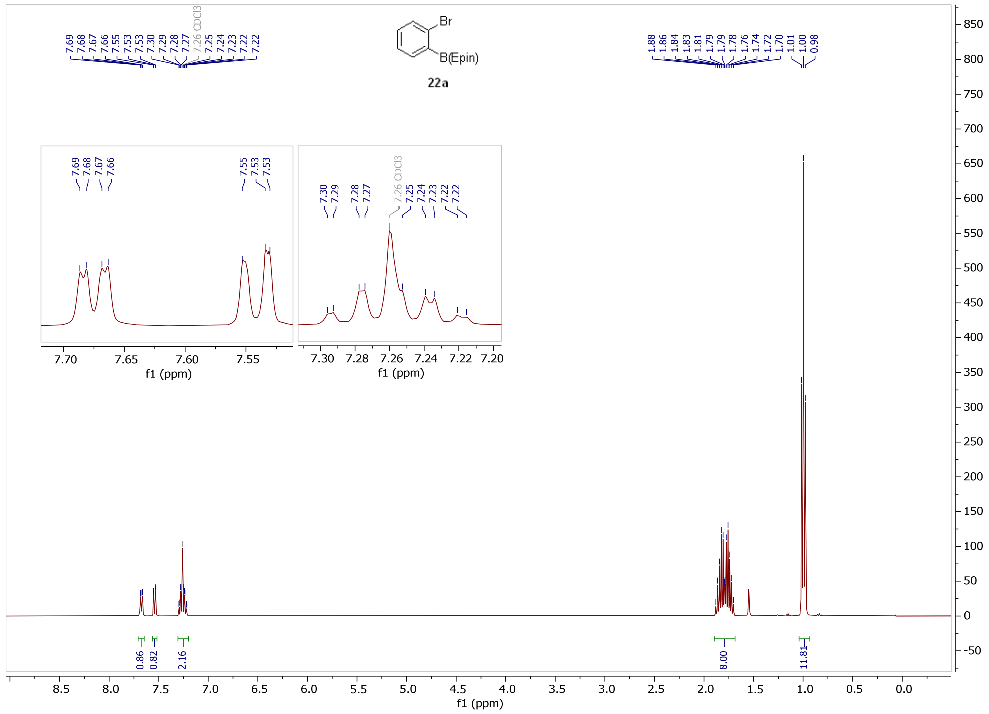

Figure S40: ^{1}H NMR spectrum of compound **22a** in $CDCl_3$.

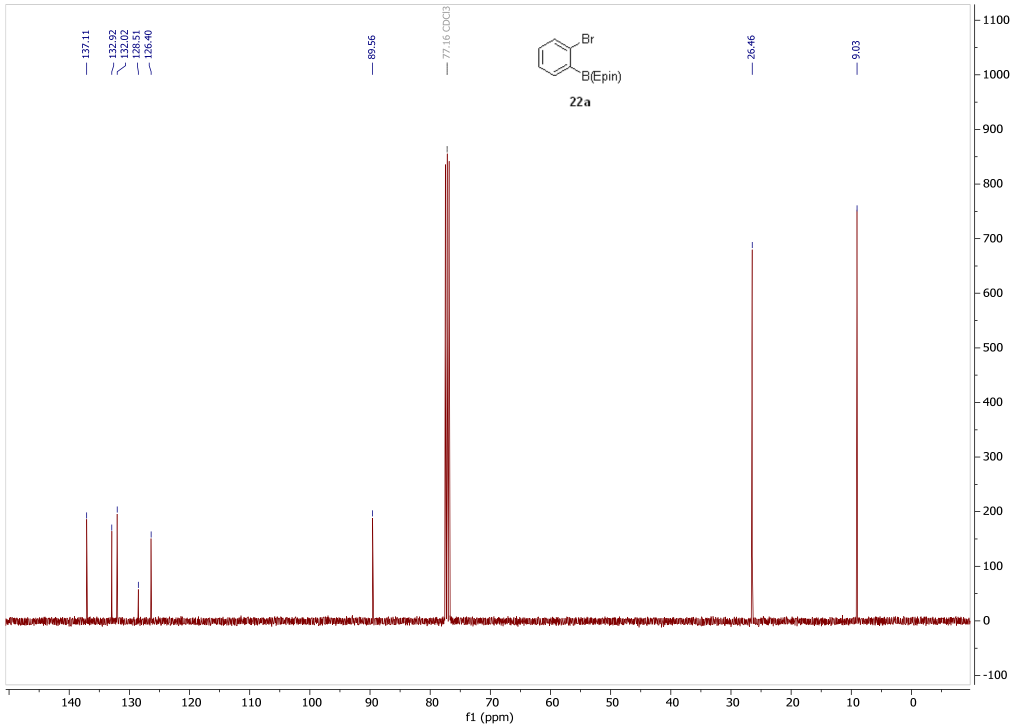

Figure S41: ^{13}C NMR spectrum of compound **22a** in $CDCl_3$.

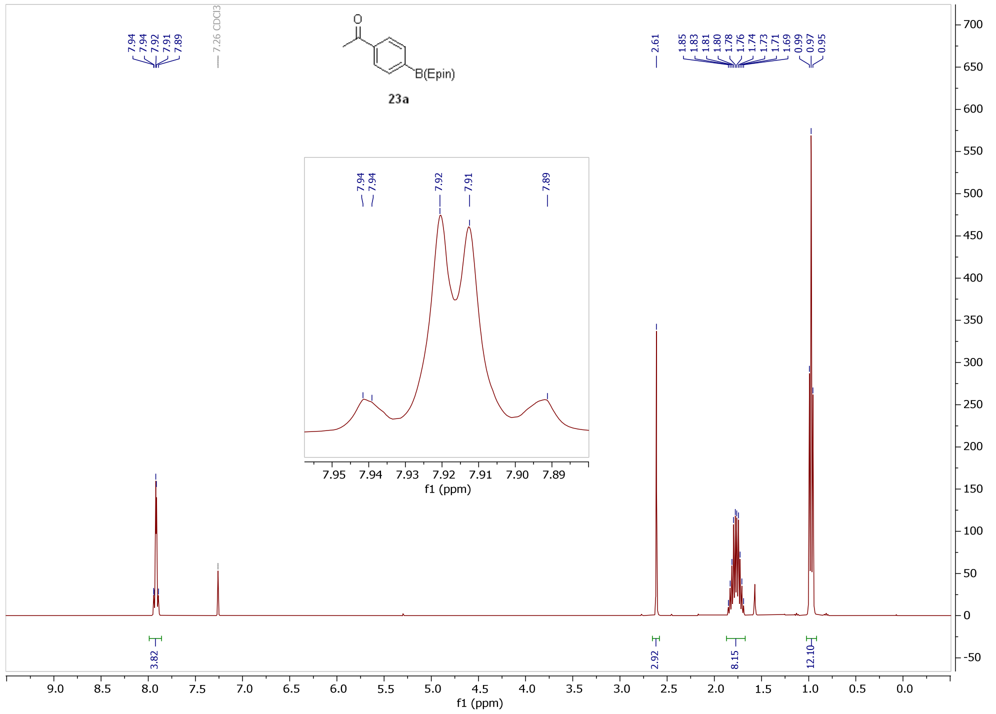

Figure S42: ^{1}H NMR spectrum of compound **23a** in $CDCl_3$.

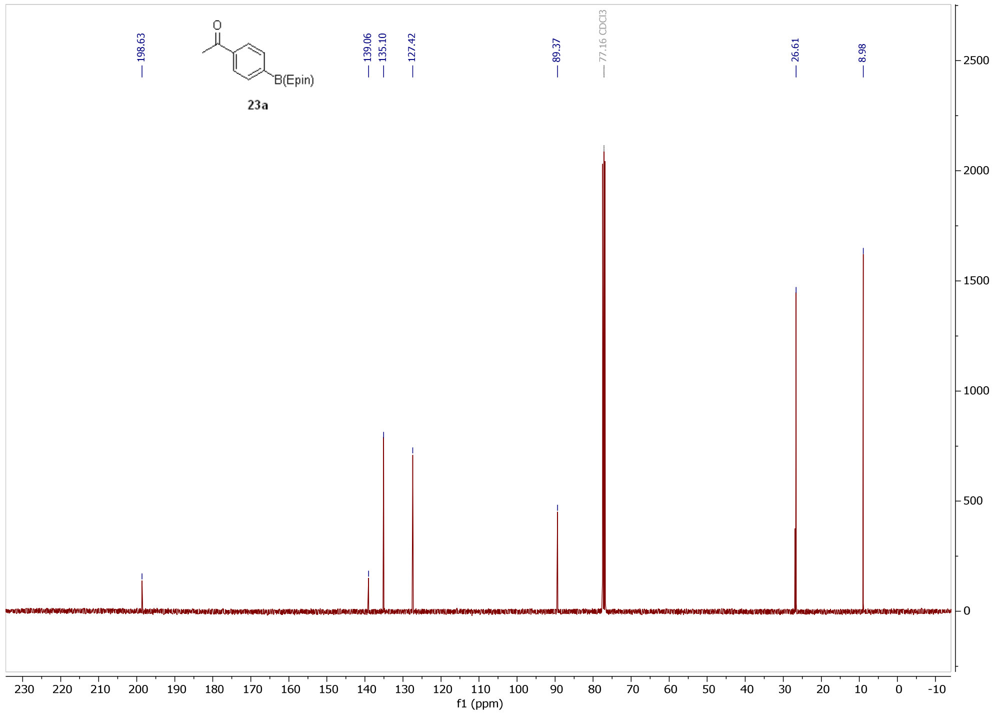

Figure S43: ^{13}C NMR spectrum of compound **23a** in $CDCl_3$.

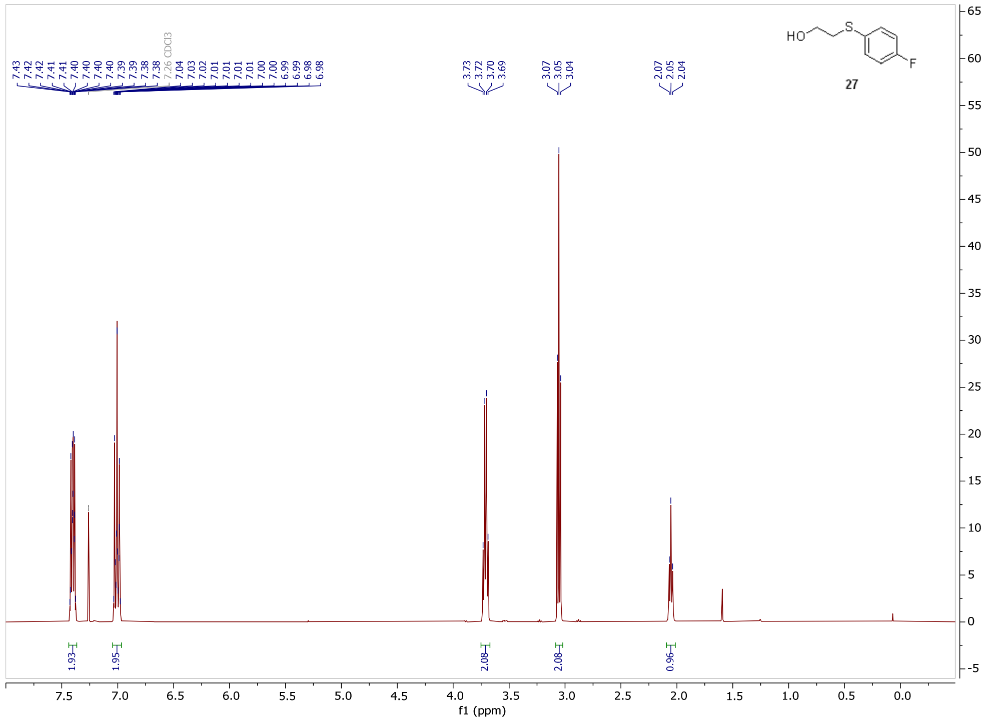

Figure S44: ^{1}H NMR spectrum of compound **27** in $CDCl_3$.

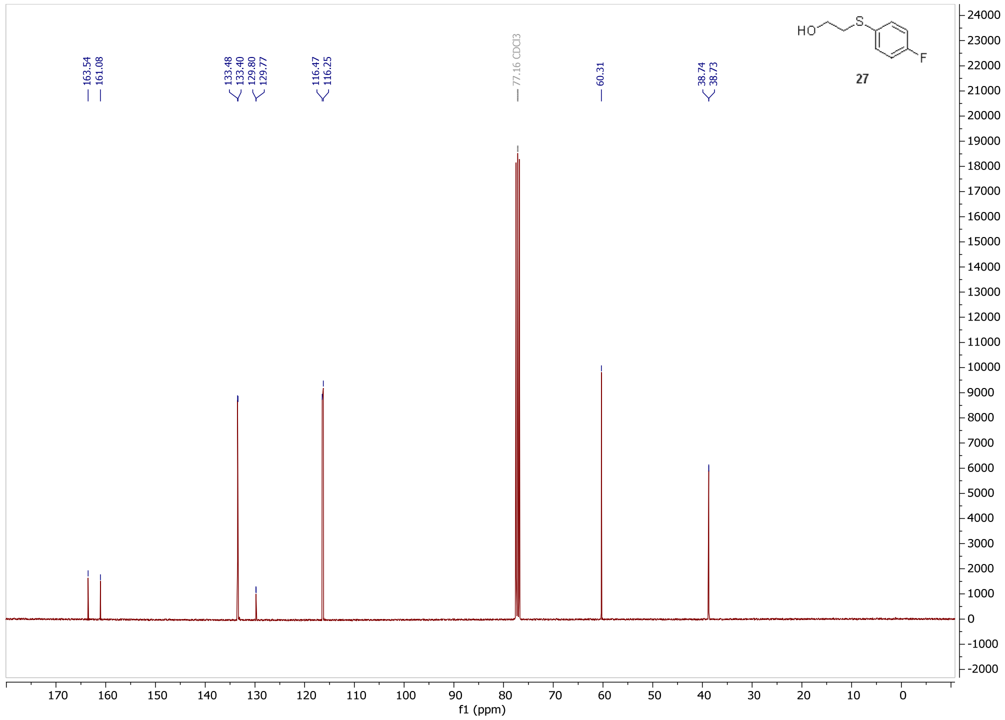

Figure S45: ^{13}C NMR spectrum of compound **27** in $CDCl_3$.

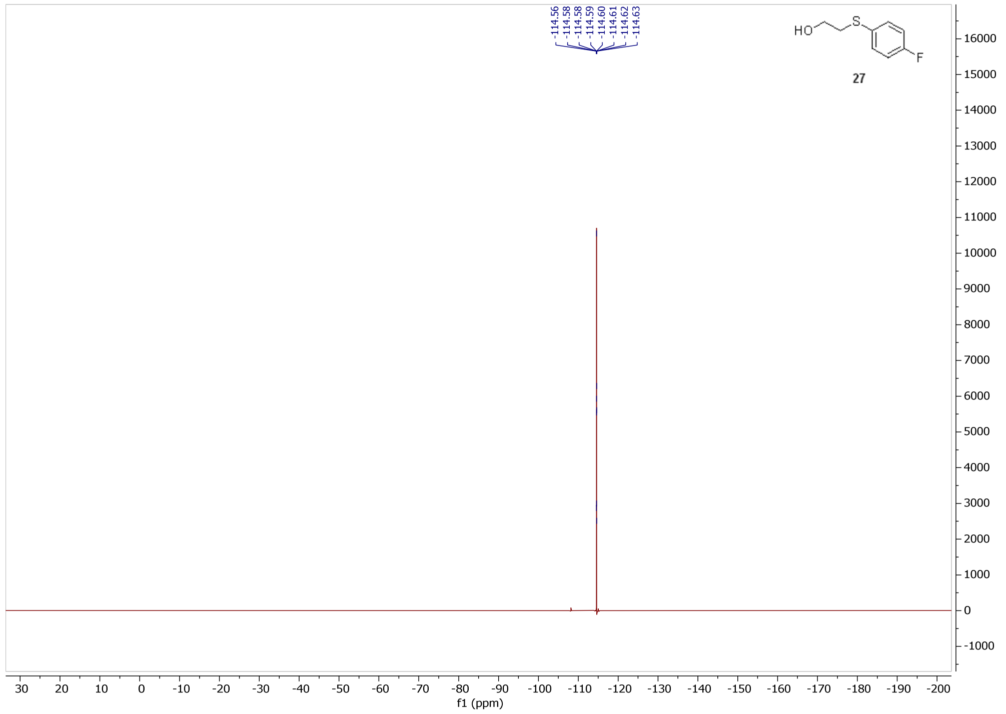

Figure S46: ^{19}F NMR spectrum of compound **27** in $CDCl_3$.

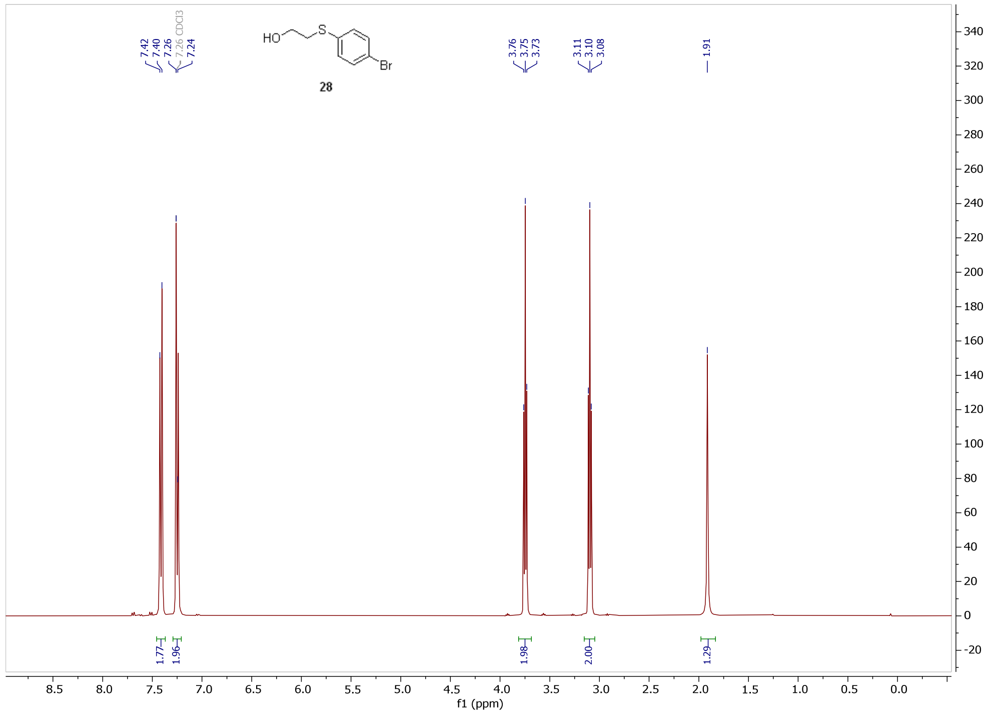

Figure S47: ^{1}H NMR spectrum of compound **28** in $CDCl_3$.

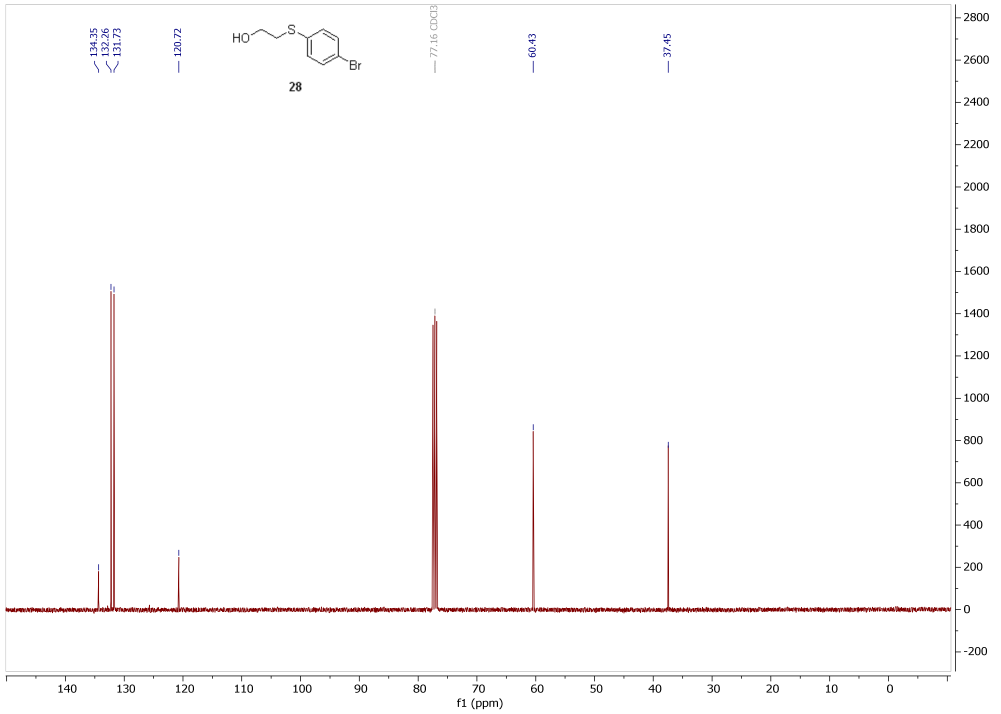

Figure S48: ^{13}C NMR spectrum of compound **28** in $CDCl_3$.

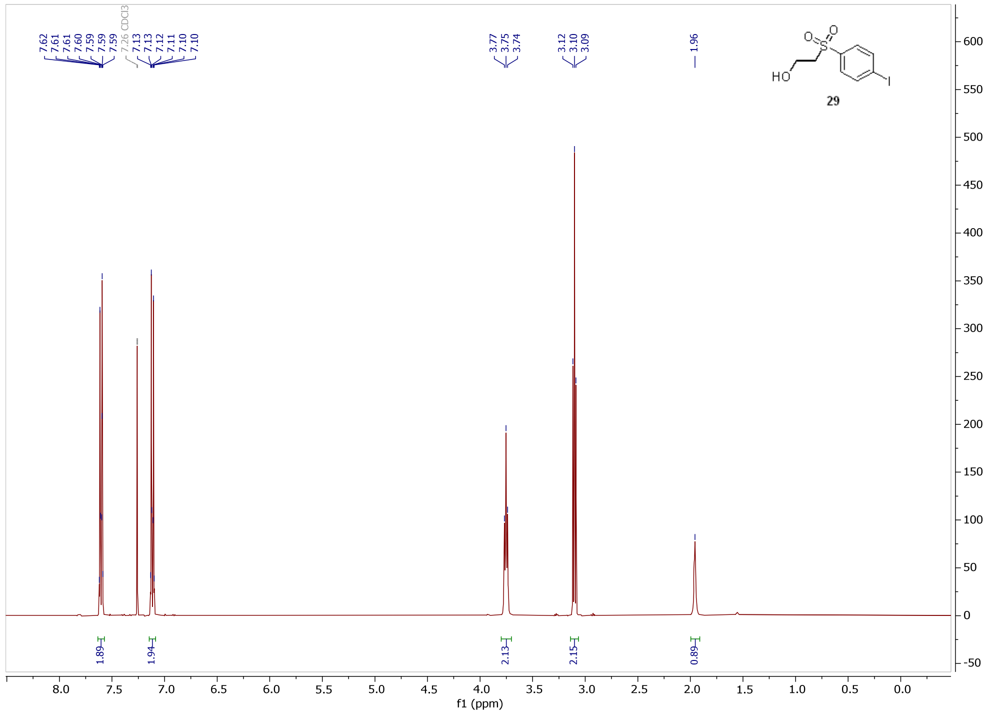

Figure S49: ^{1}H NMR spectrum of compound **29** in $CDCl_3$.

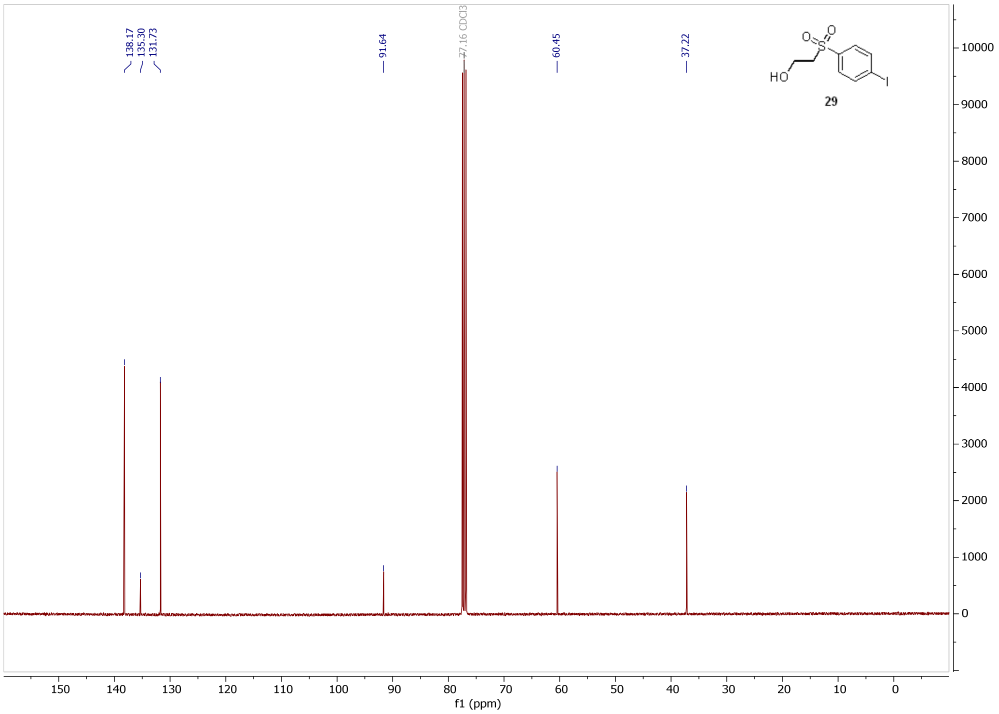

Figure S50: ^{13}C NMR spectrum of compound **29** in $CDCl_3$.

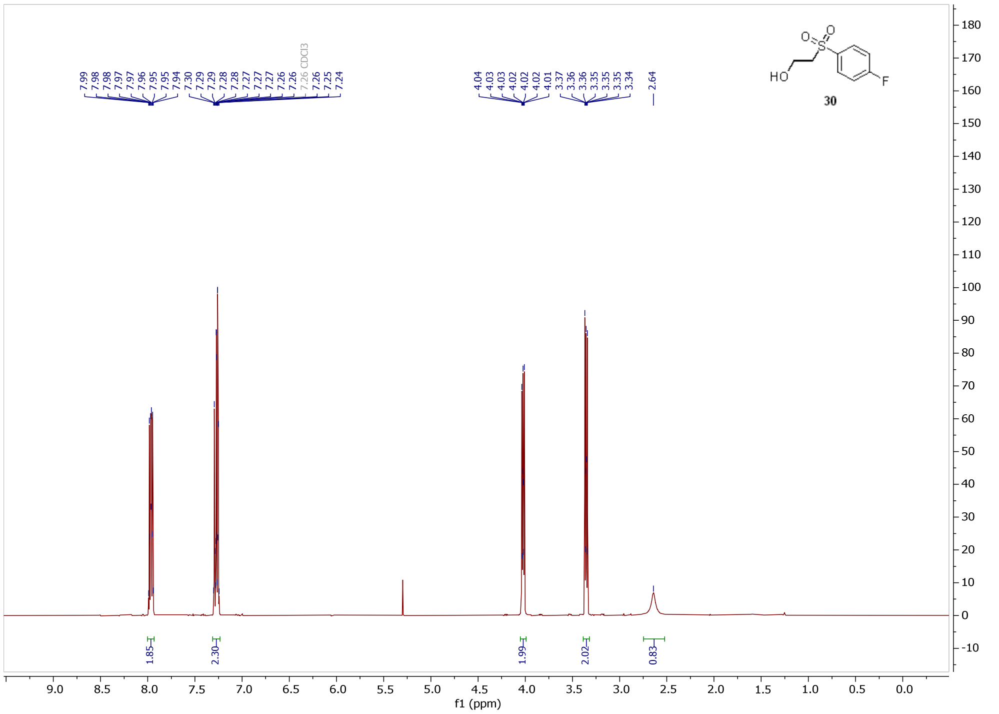

Figure S51: ^{1}H NMR spectrum of compound **30** in $CDCl_3$.

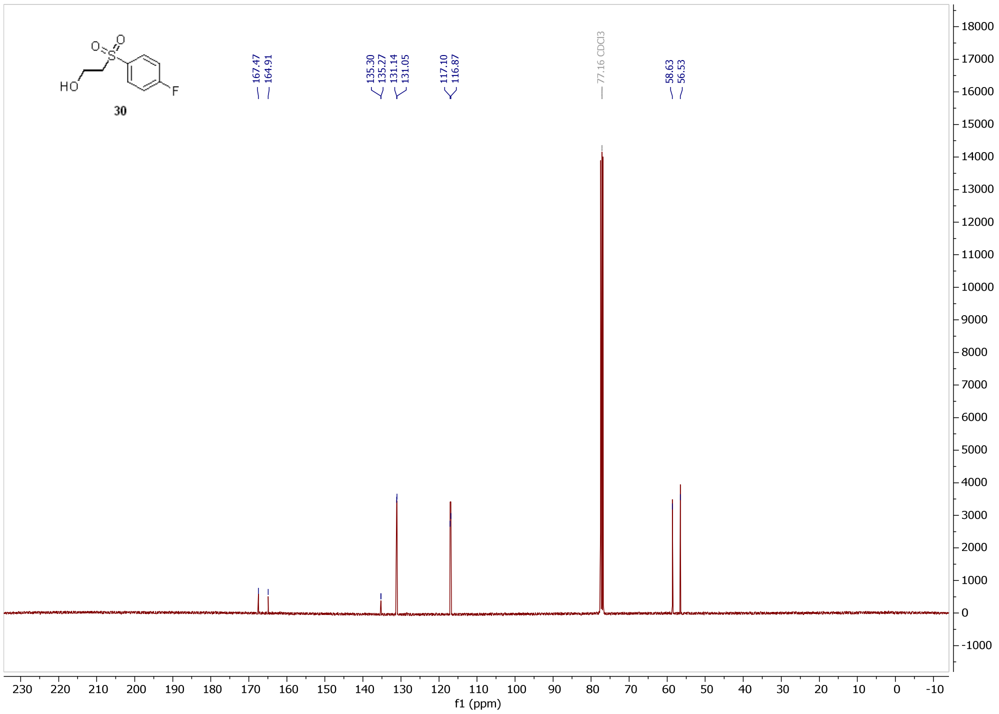

Figure S52: ^{13}C NMR spectrum of compound **30** in $CDCl_3$.

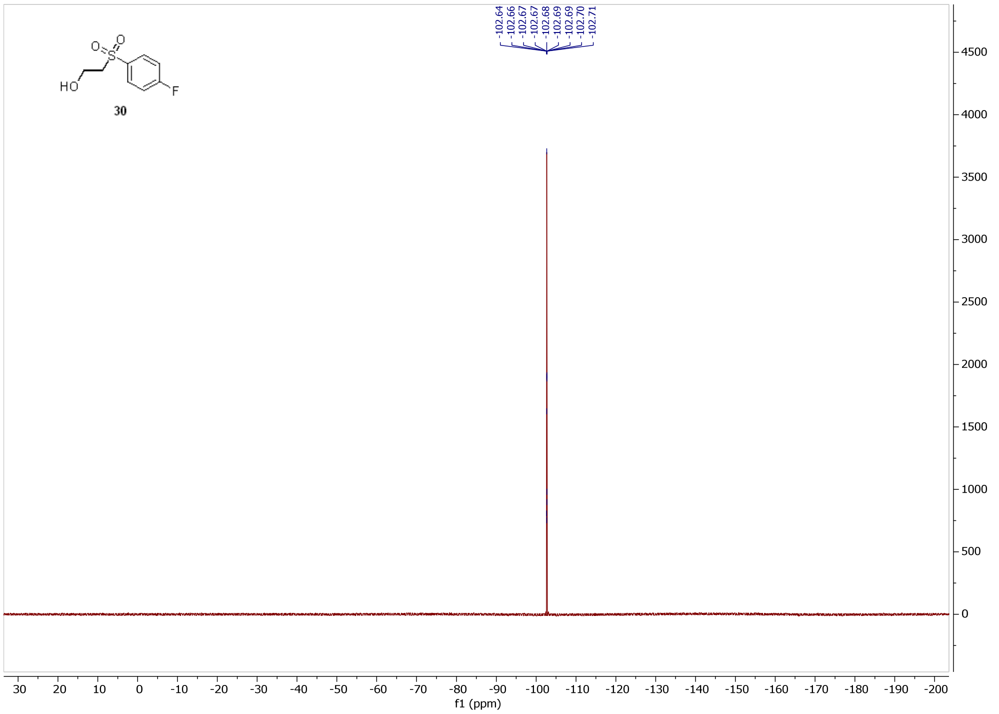

Figure S 53: ^{19}F NMR spectrum of compound **30** in $CDCl_3$.

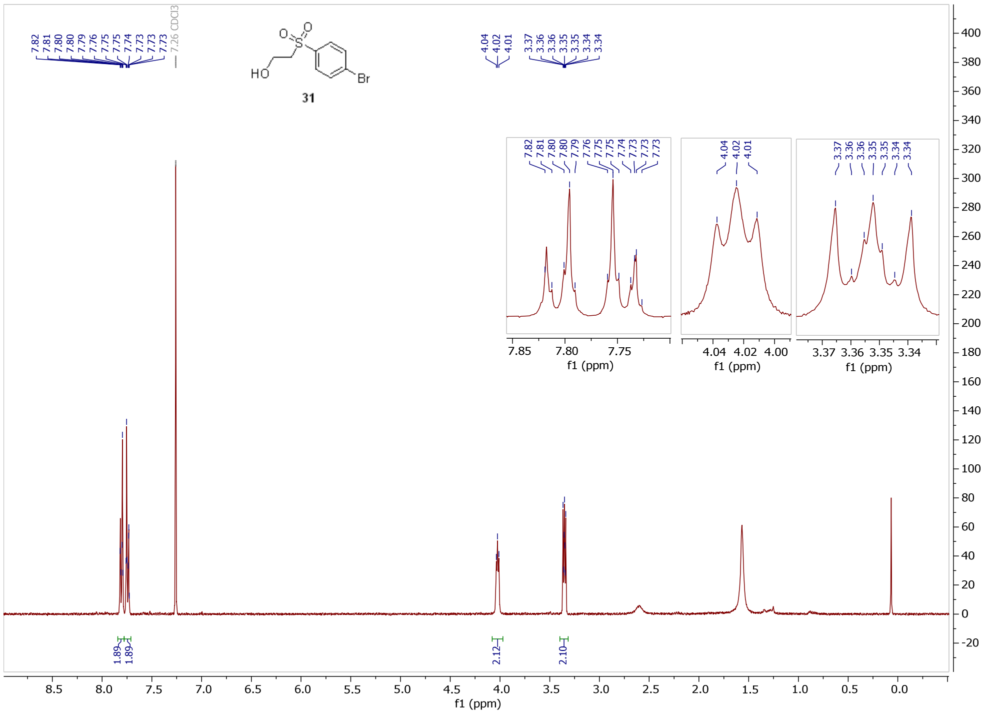

Figure S54: ^{1}H NMR spectrum of compound **31** in $CDCl_3$.

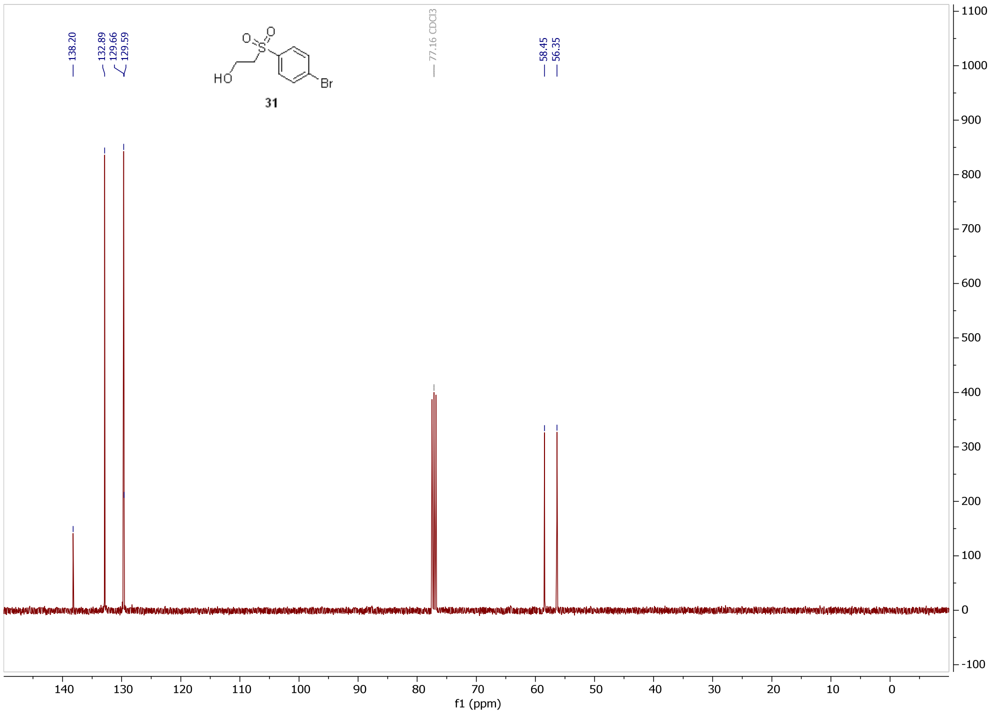

Figure S55: ^{13}C NMR spectrum of compound **31** in $CDCl_3$.

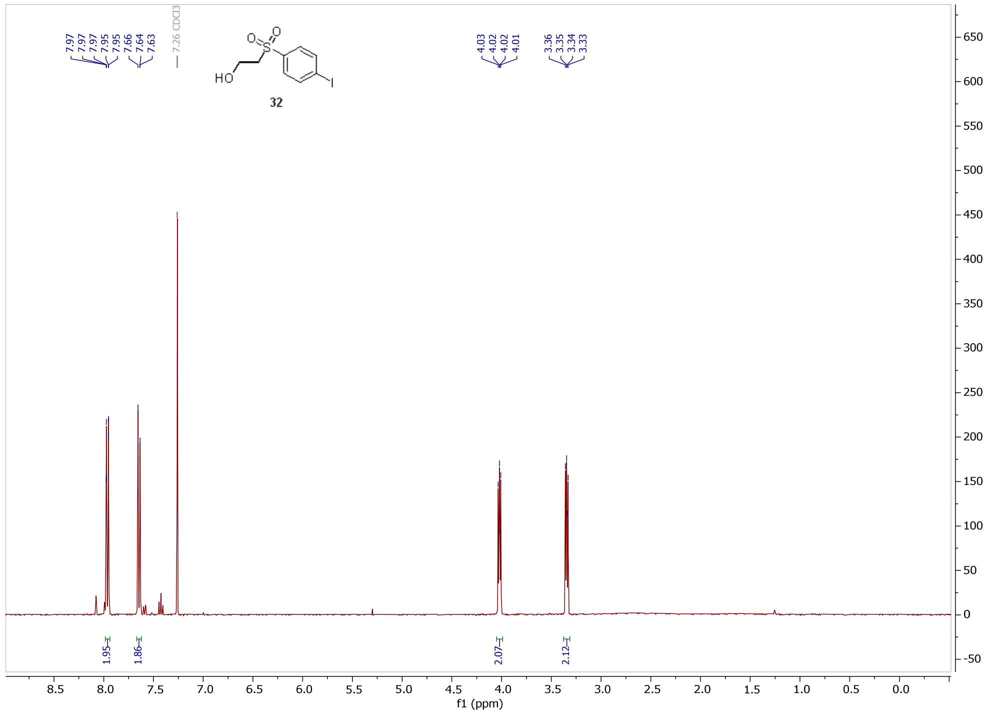

Figure S56: ^{1}H NMR spectrum of compound **32** in $CDCl_3$.

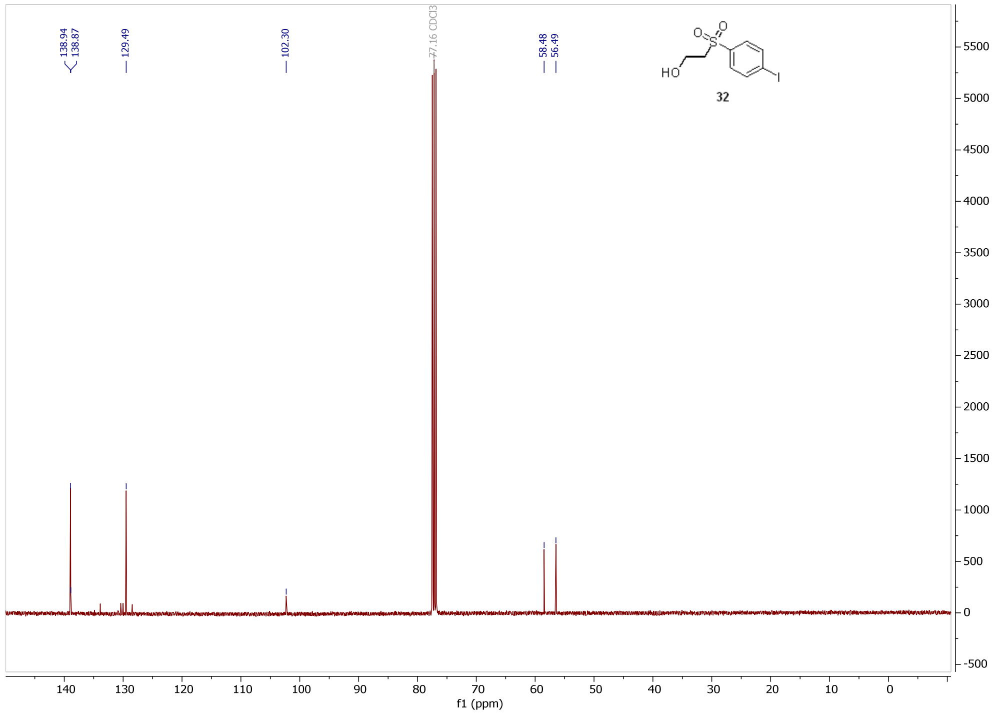

Figure S57: ^{13}C NMR spectrum of compound **32** in $CDCl_3$.

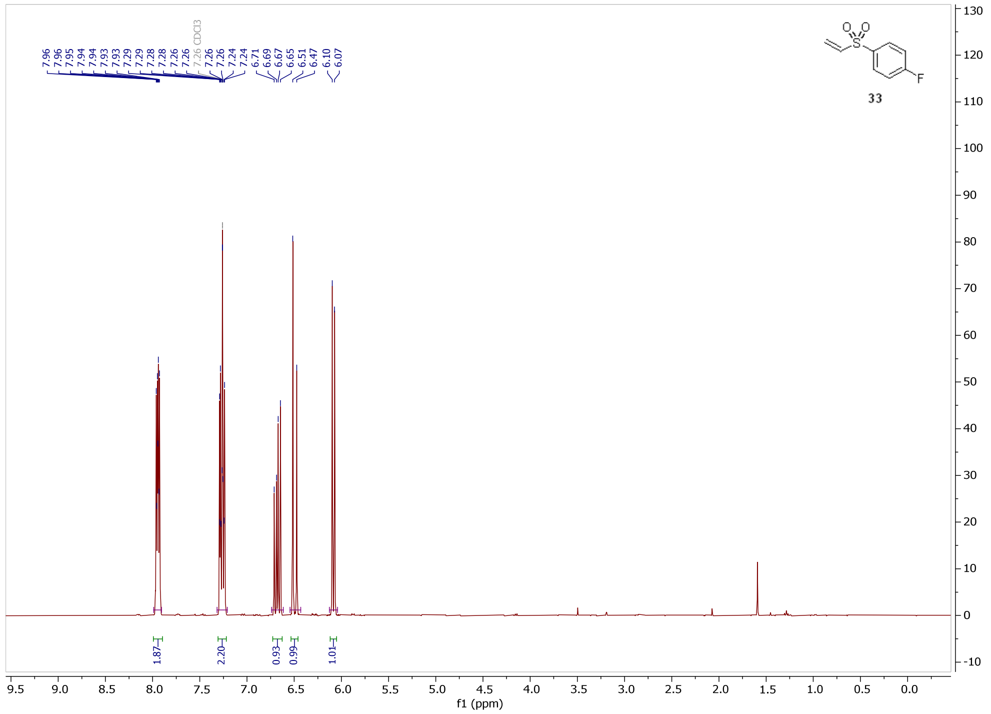

Figure S58: ^{1}H NMR spectrum of compound **33** in $CDCl_3$.

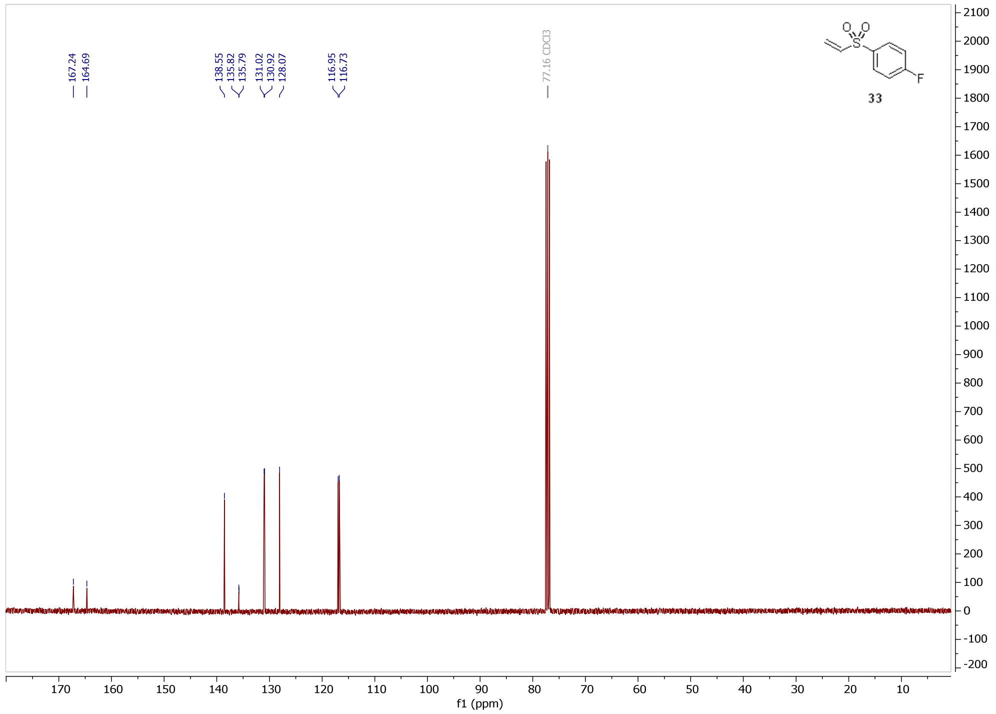

Figure S59: ^{13}C NMR spectrum of compound **33** in $CDCl_3$.

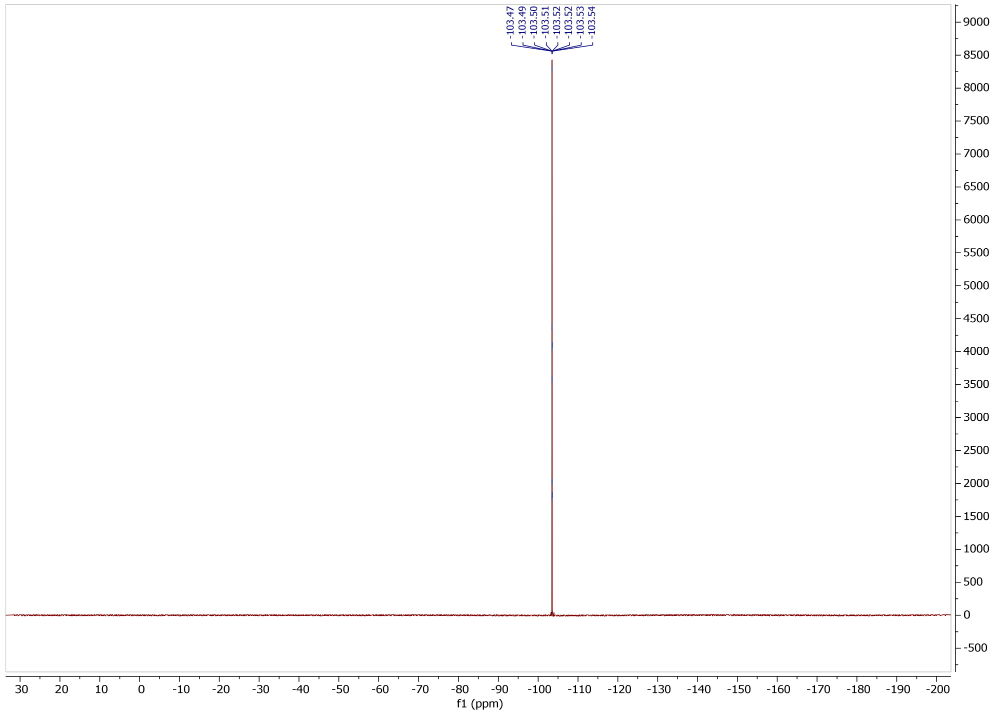

Figure S60: ^{19}F NMR spectrum of compound **33** in $CDCl_3$.

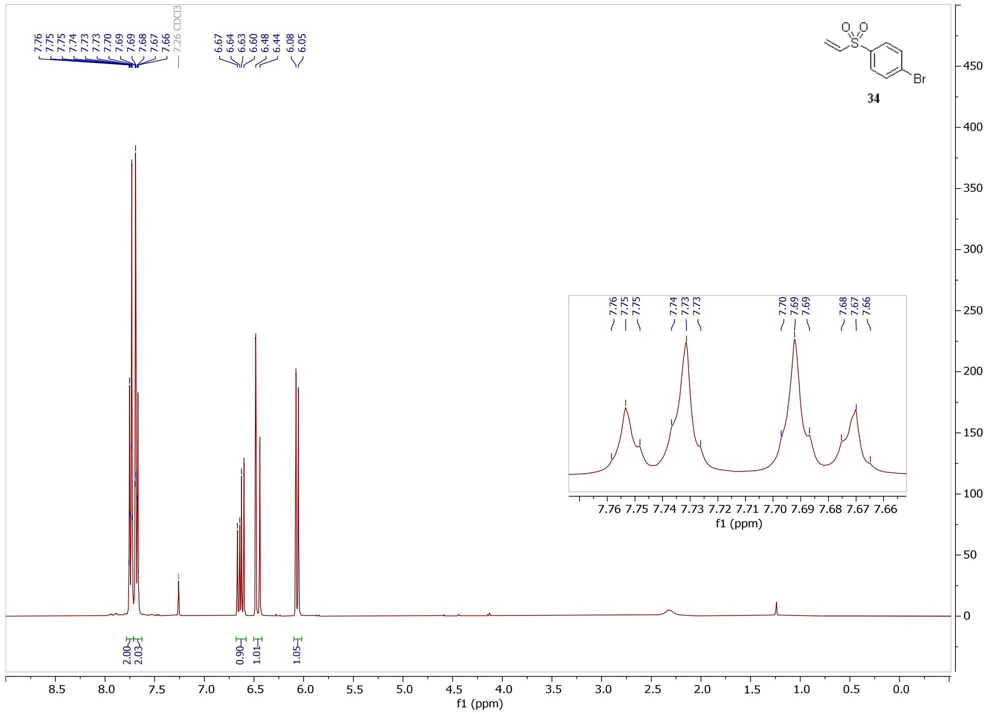

Figure S61: ^{1}H NMR spectrum of compound **34** in $CDCl_3$.

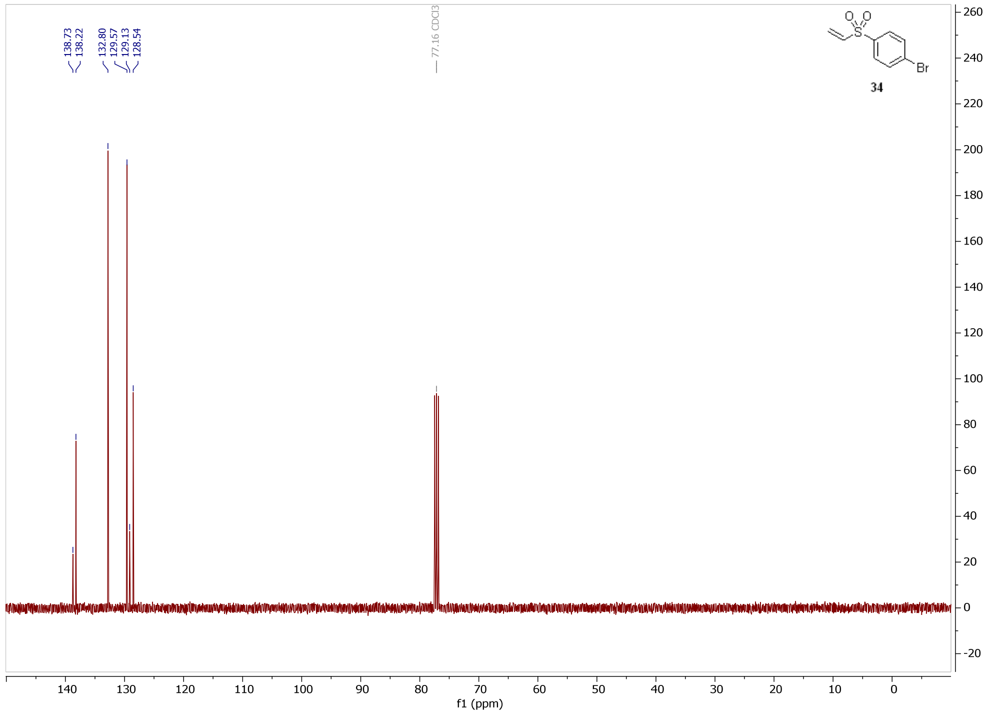

Figure S62: ^{13}C NMR spectrum of compound **34** in $CDCl_3$.

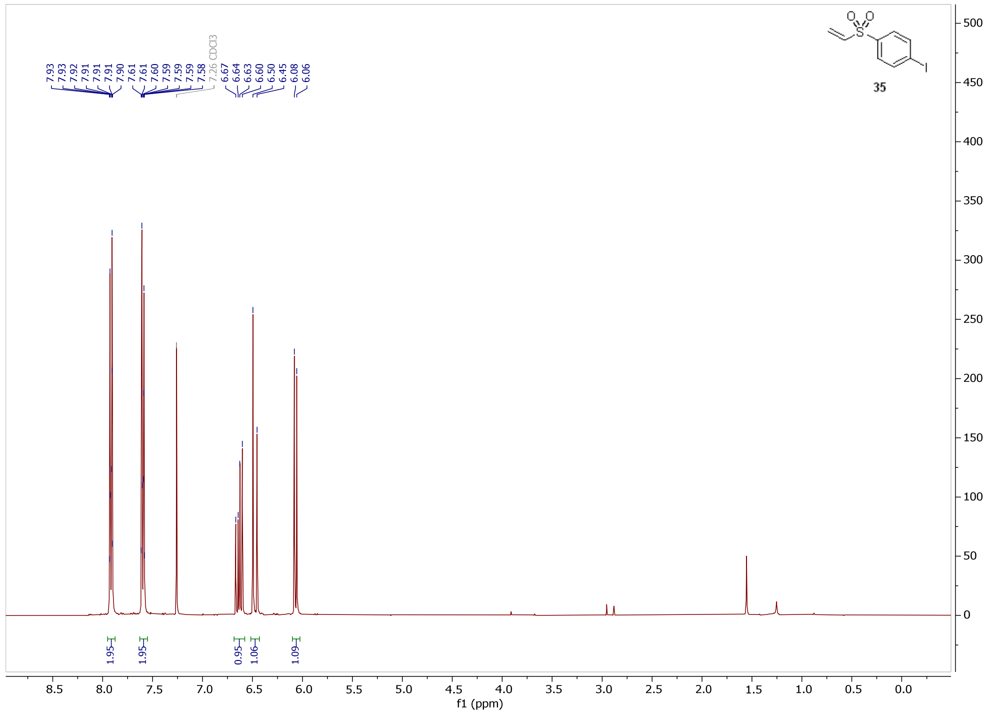

Figure S63: ^{1}H NMR spectrum of compound **35** in $CDCl_3$.

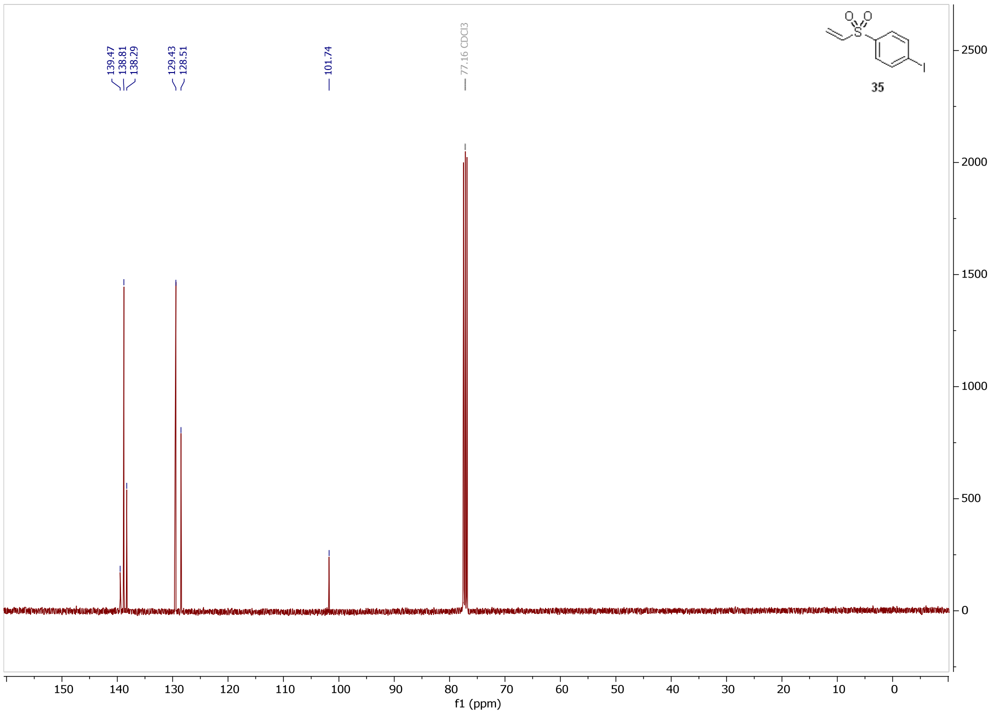

Figure S64: ^{13}C NMR spectrum of compound **35** in $CDCl_3$.

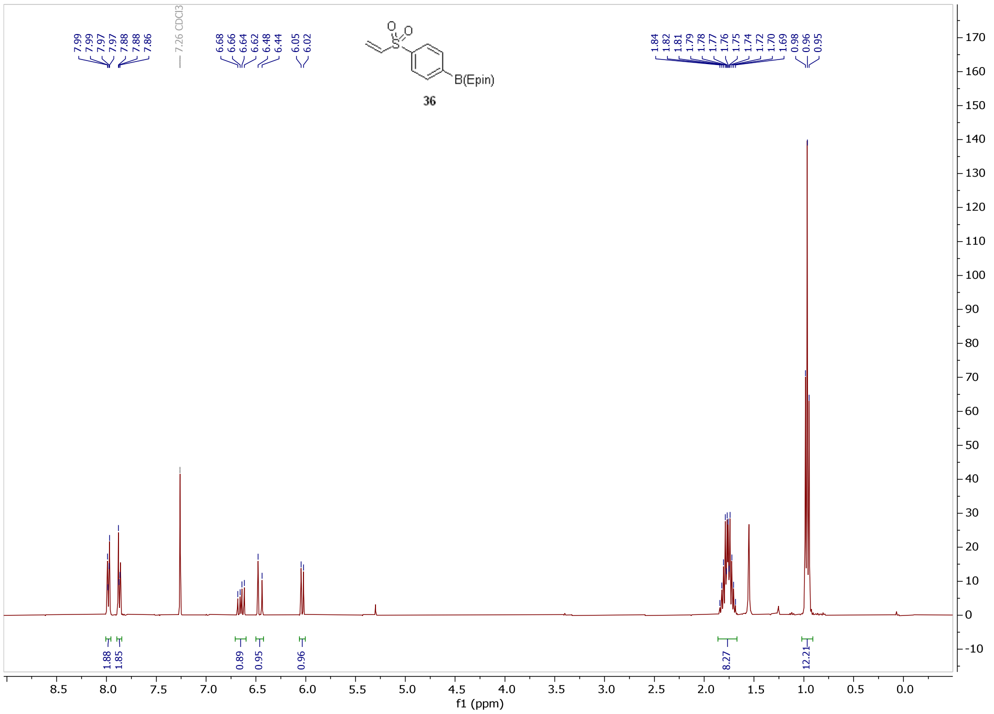

Figure S65: ^{1}H-NMR spectrum of compound **36** in $CDCl_3$.

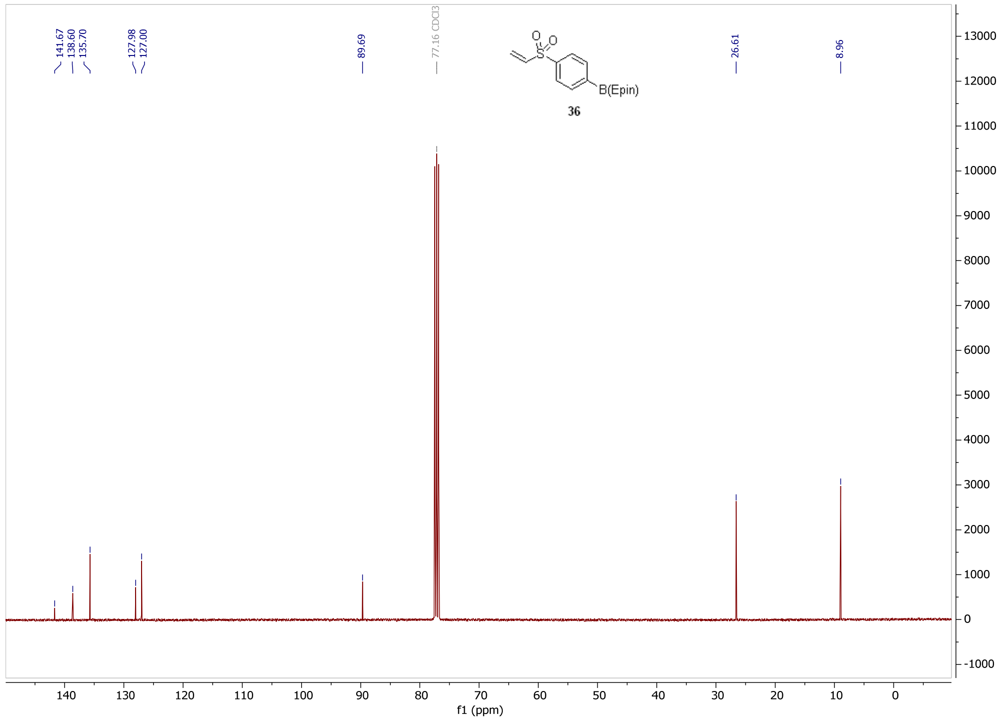

Figure S66: ^{13}C NMR spectrum of compound **36** in $CDCl_3$.

^1H, ^{13}C and ^{19}F NMR Spectra of PPin Compounds

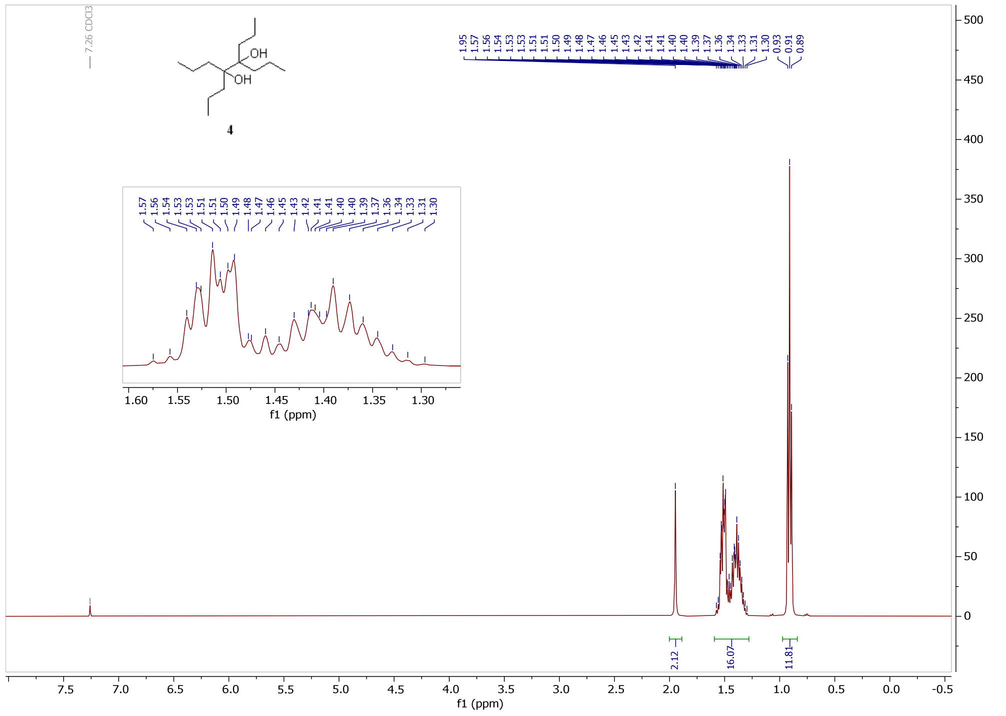

Figure S67: ^1H NMR spectrum of compound **4** in $CDCl_3$.

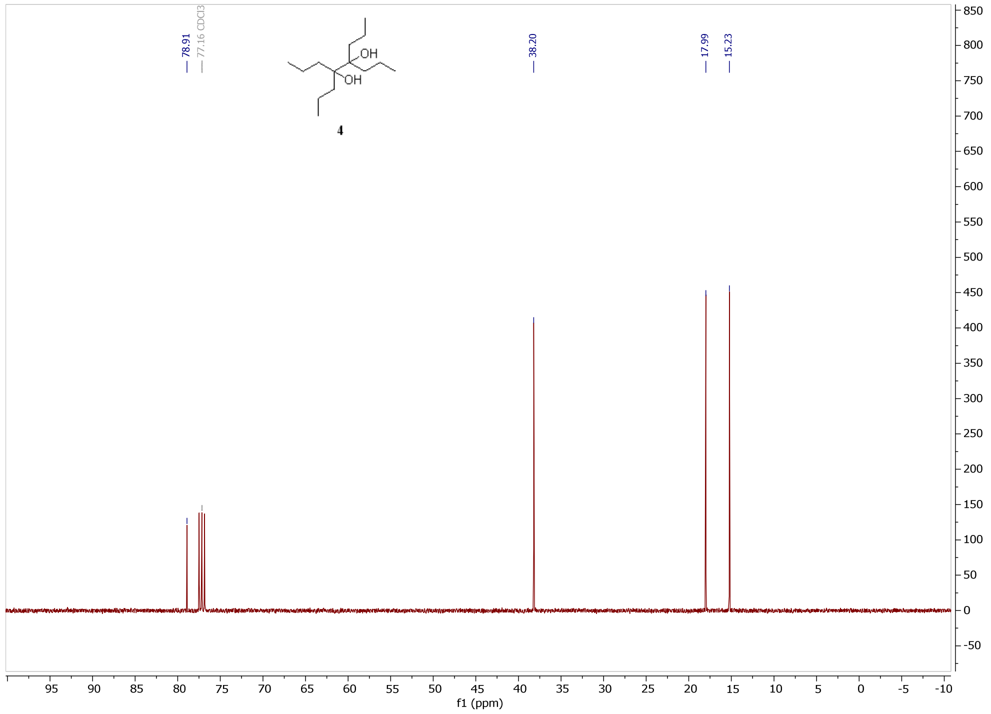

Figure S68: ^{13}C NMR spectrum of compound **4** in $CDCl_3$.

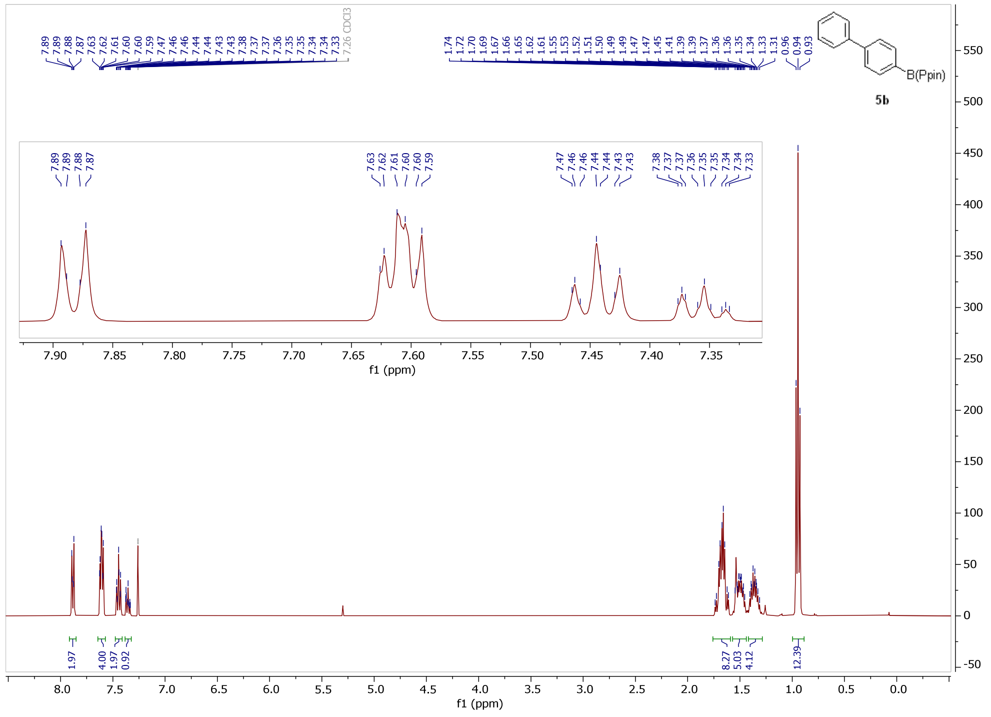

Figure S69: ^{1}H NMR spectrum of compound **5b** in $CDCl_3$.

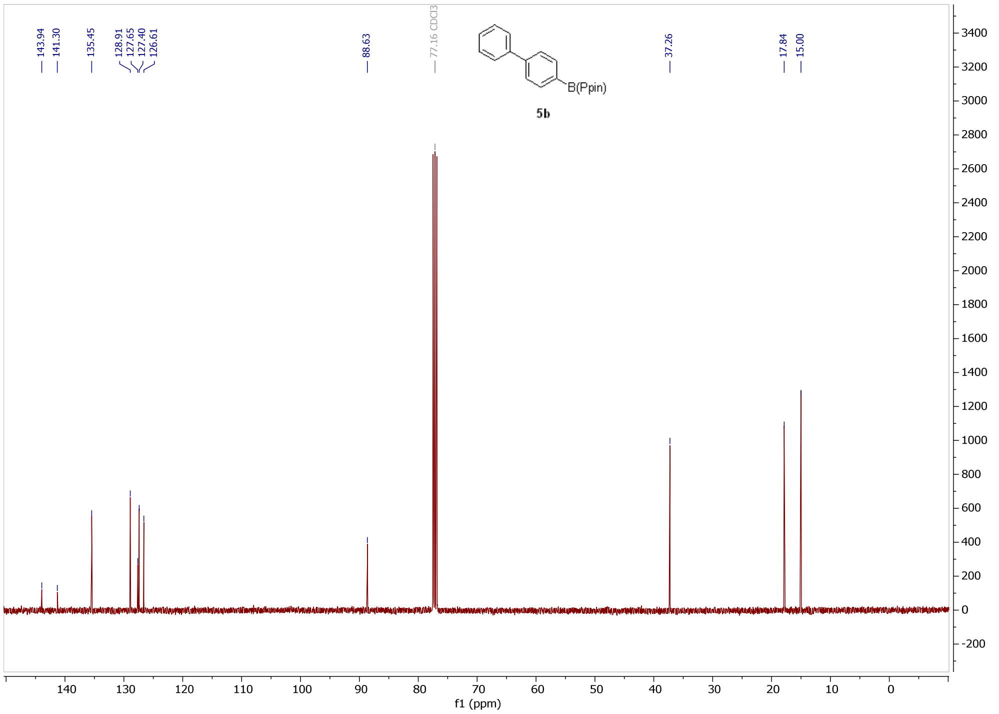

Figure S70: ^{13}C NMR spectrum of compound **5b** in $CDCl_3$.

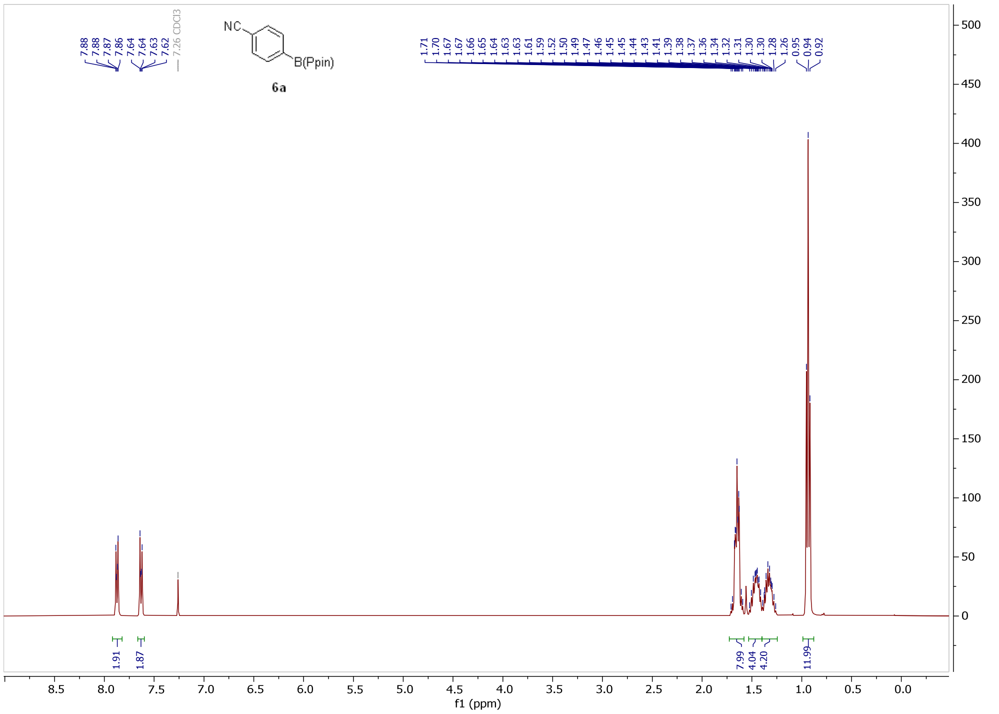

Figure S71: ^{1}H NMR spectrum of compound **6b** in $CDCl_3$.

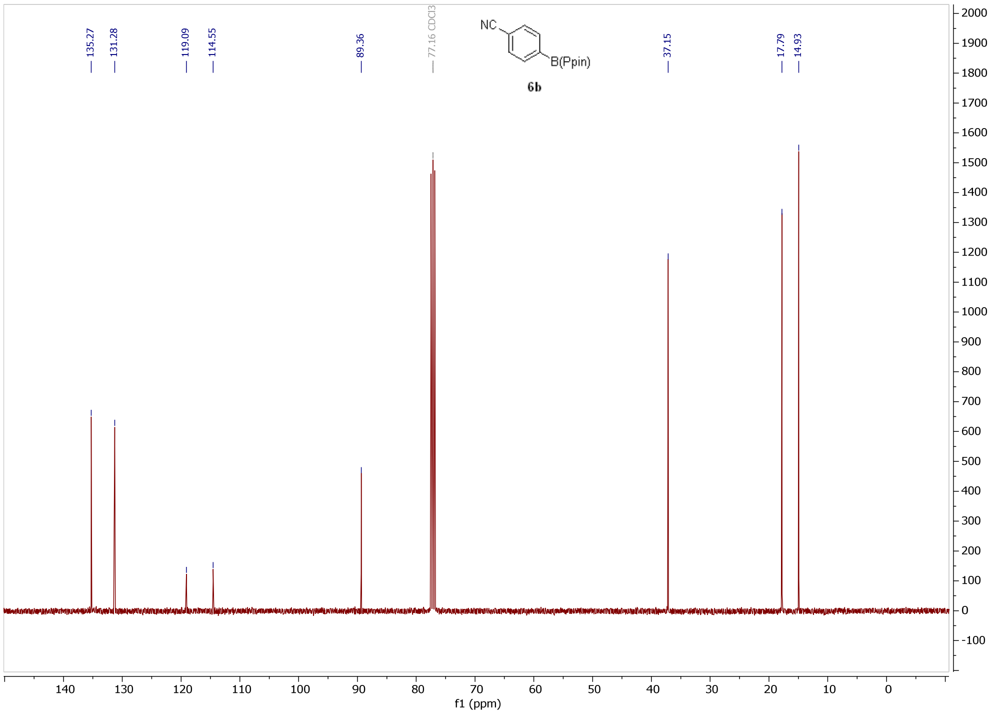

Figure S72: ^{13}C NMR spectrum of compound **6b** in $CDCl_3$.

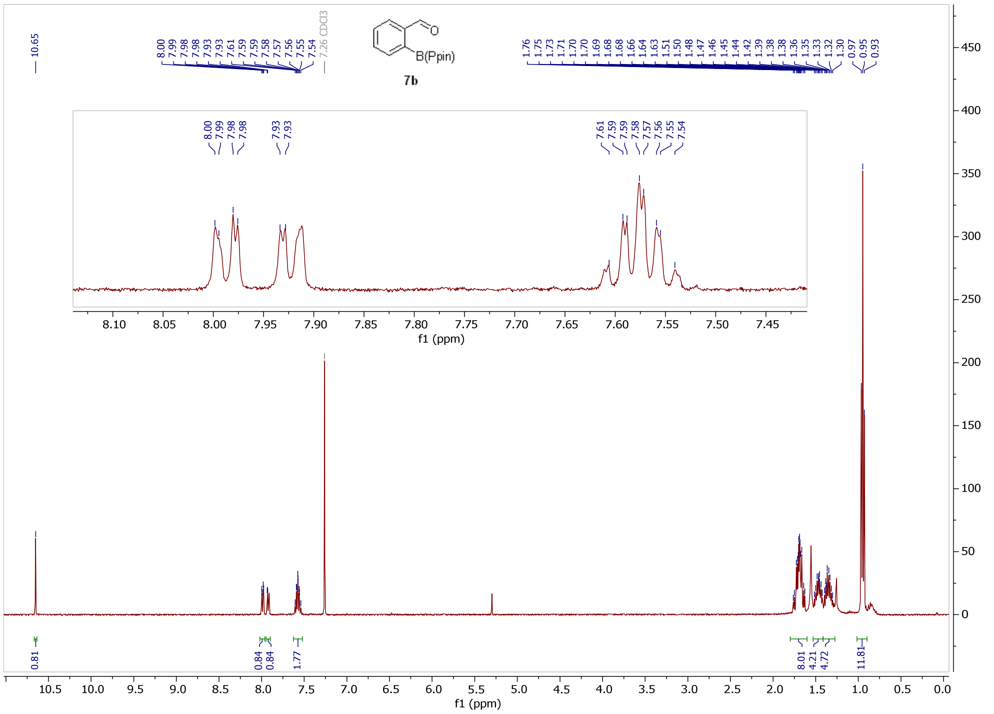

Figure S73: ^{1}H NMR spectrum of compound **7b** in $CDCl_3$.

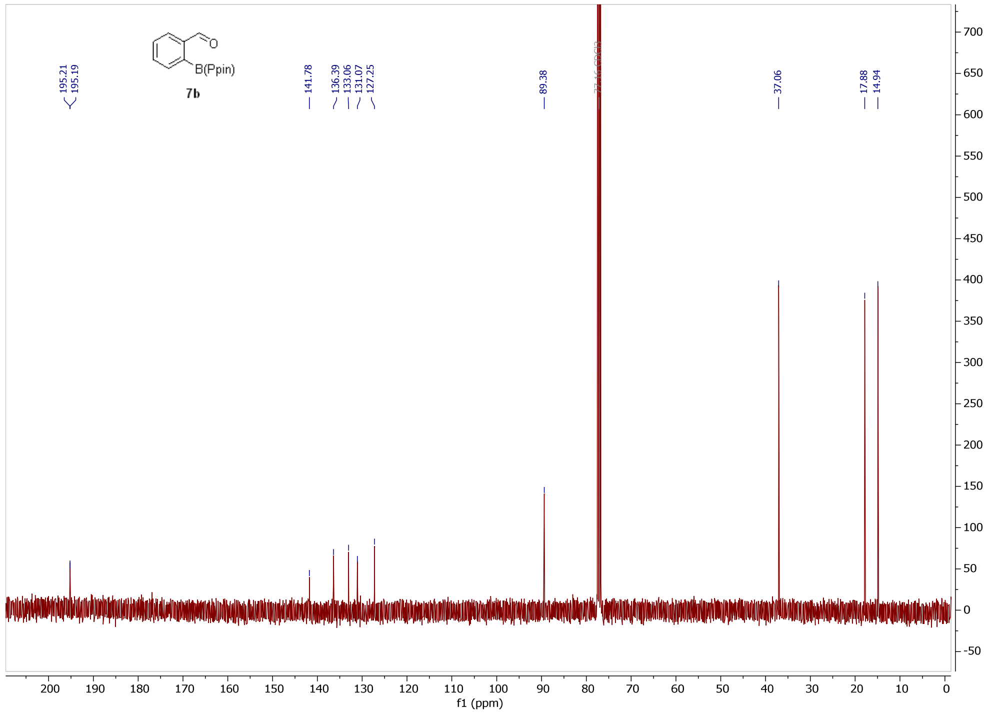

Figure S74: ^{13}C NMR spectrum of compound **7b** in $CDCl_3$.

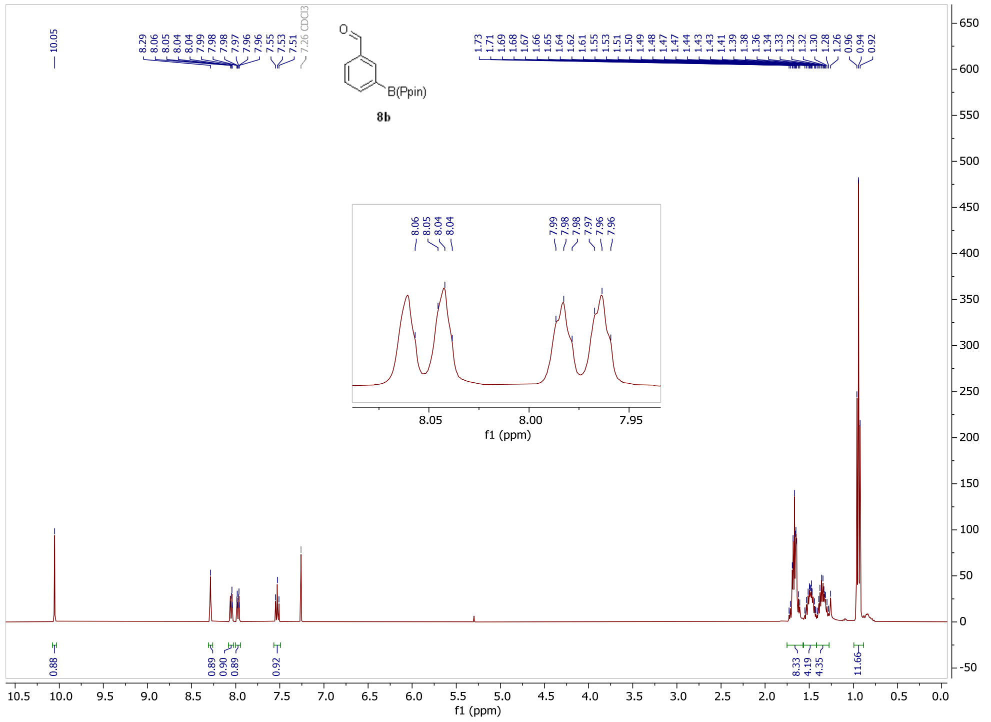

Figure S75: ^{1}H NMR spectrum of compound **8b** in $CDCl_3$.

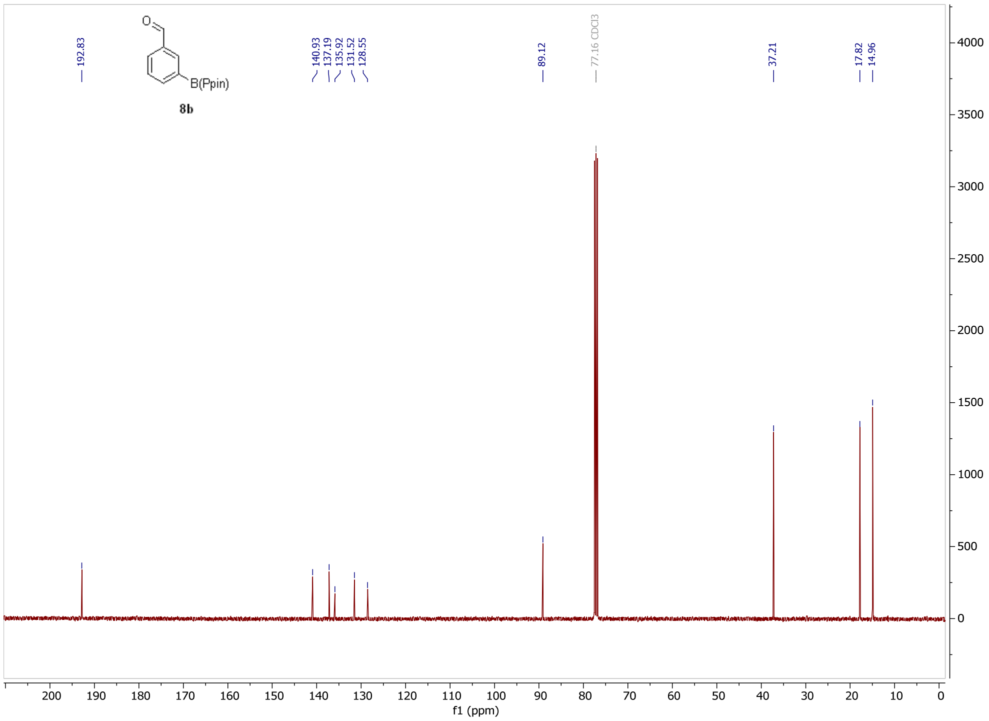

Figure S76: ^{13}C NMR spectrum of compound **8b** in $CDCl_3$.

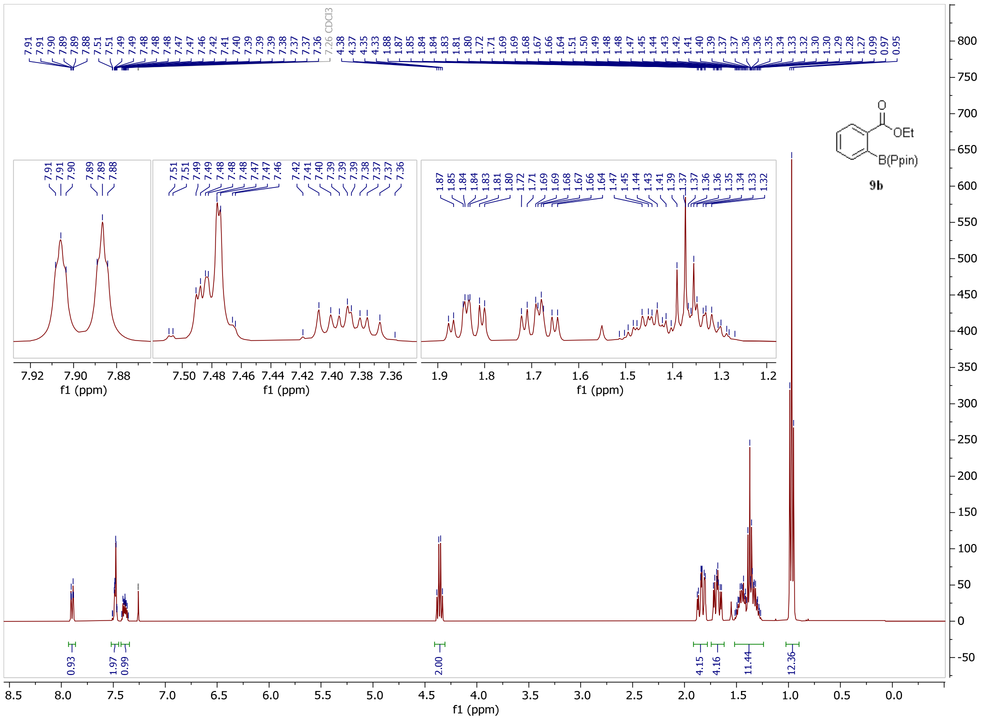

Figure S77: ^{1}H NMR spectrum of compound **9b** in $CDCl_3$.

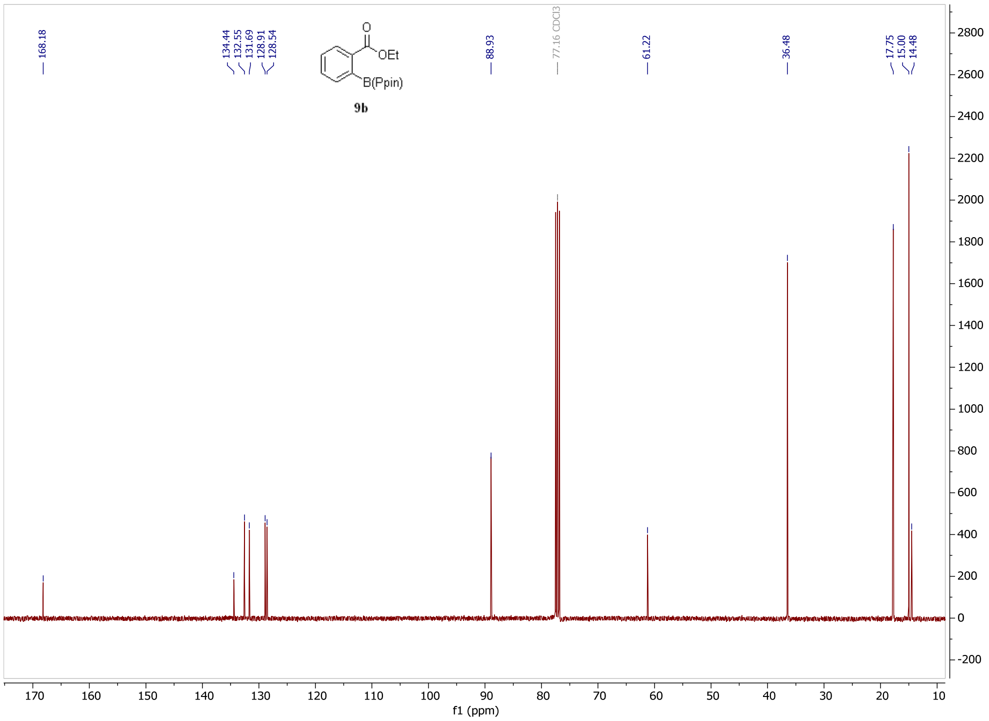

Figure S78: ^{13}C NMR spectrum of compound **9b** in $CDCl_3$.

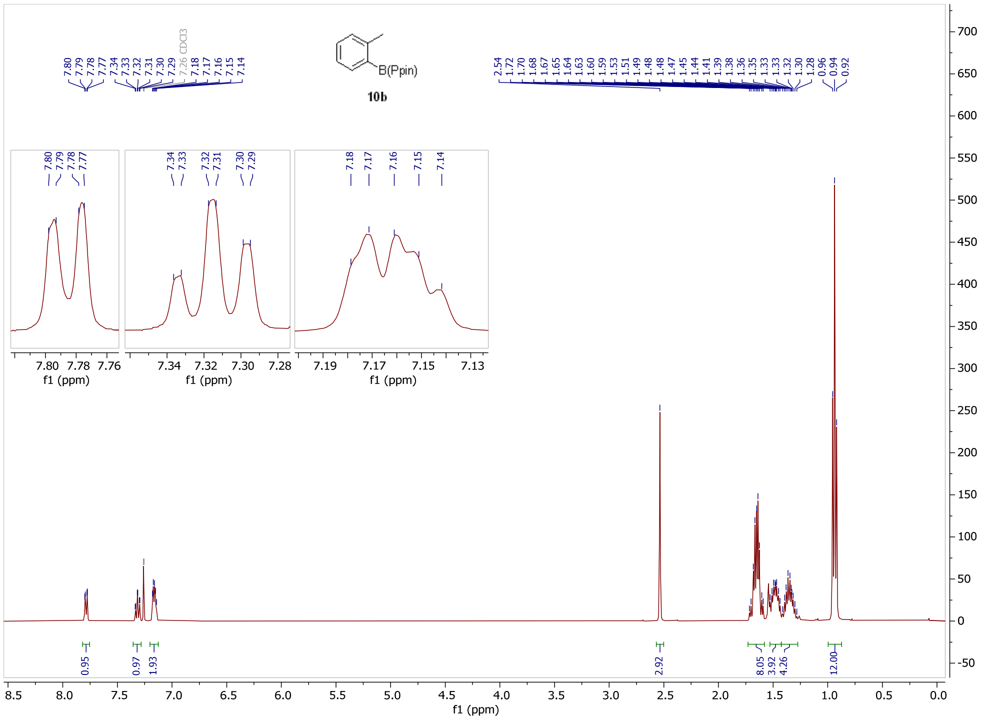

Figure S79: ^{1}H NMR spectrum of compound **10b** in $CDCl_3$.

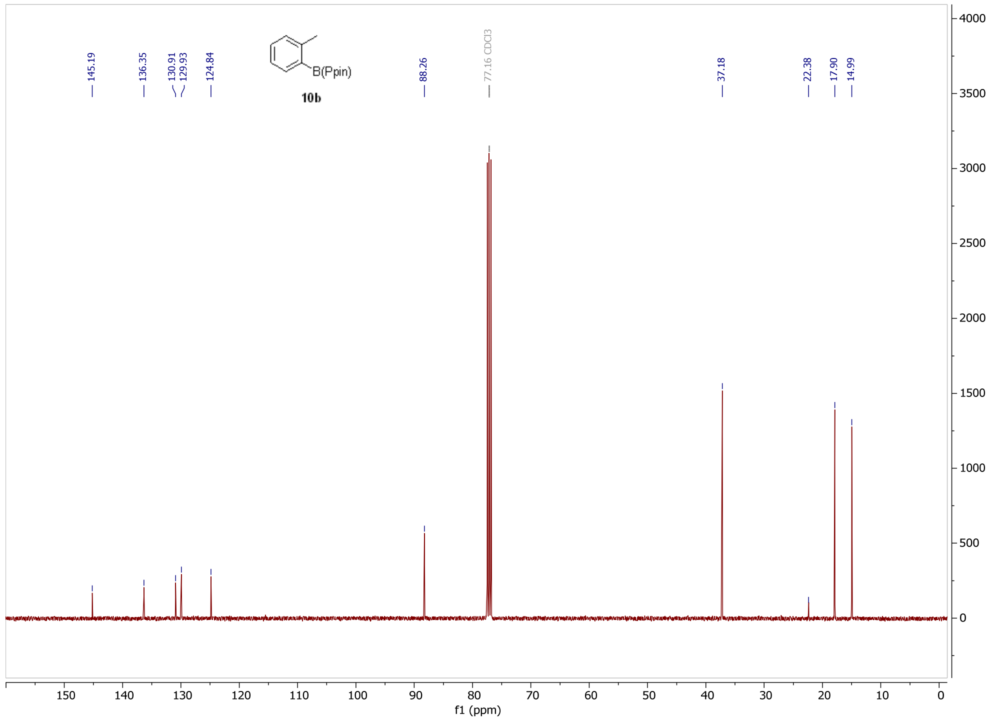

Figure S80: ^{13}C NMR spectrum of compound **10b** in $CDCl_3$.

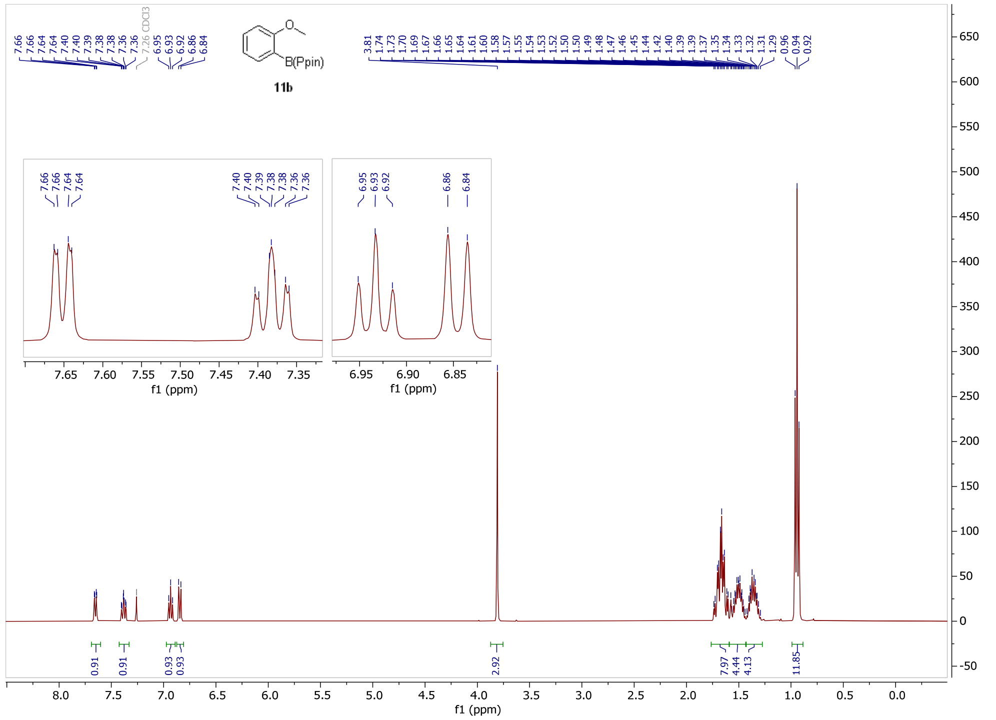

Figure S81: ^{1}H NMR spectrum of compound **11b** in $CDCl_3$.

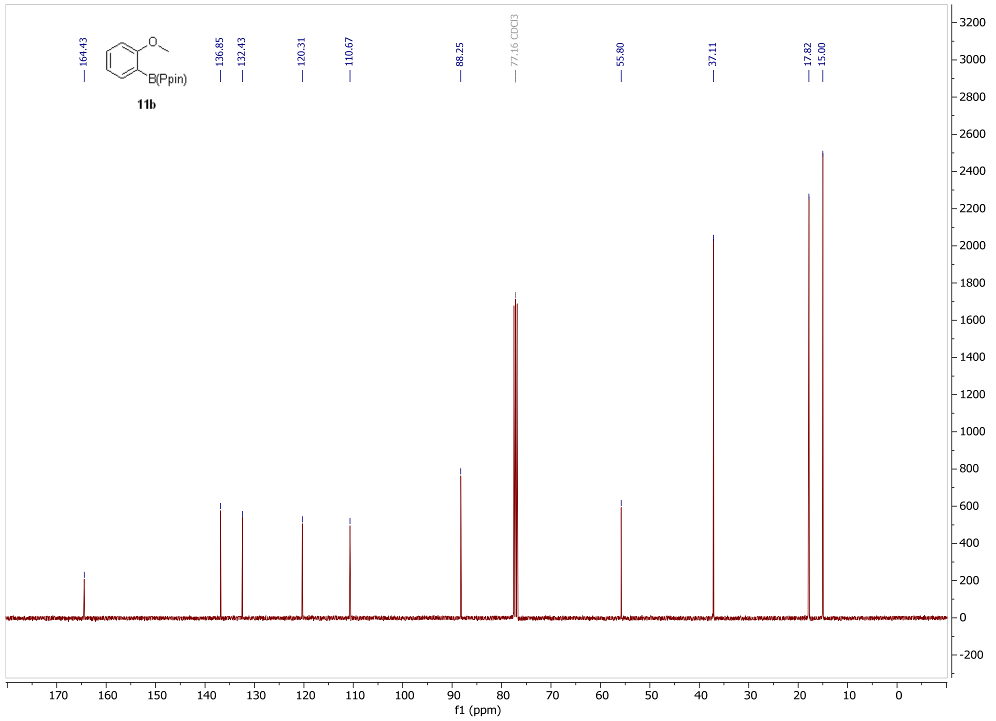

Figure S82: ^{13}C NMR spectrum of compound **11b** in $CDCl_3$.

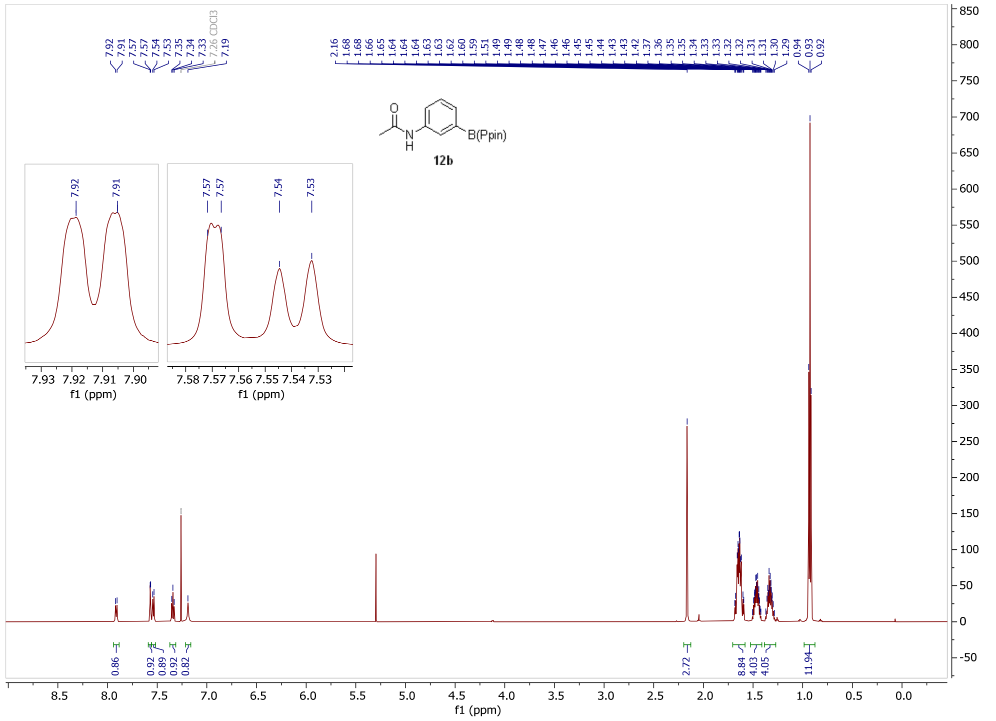

Figure S83: ^{1}H NMR spectrum of compound **12b** in $CDCl_3$.

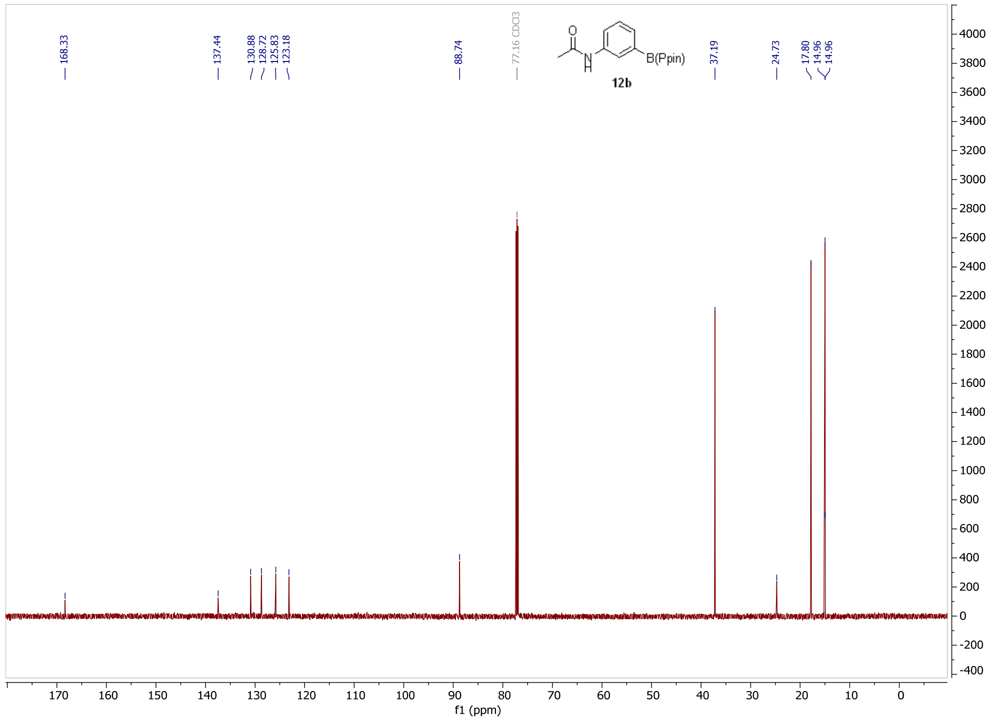

Figure S84: ^{13}C NMR spectrum of compound **12b** in $CDCl_3$.

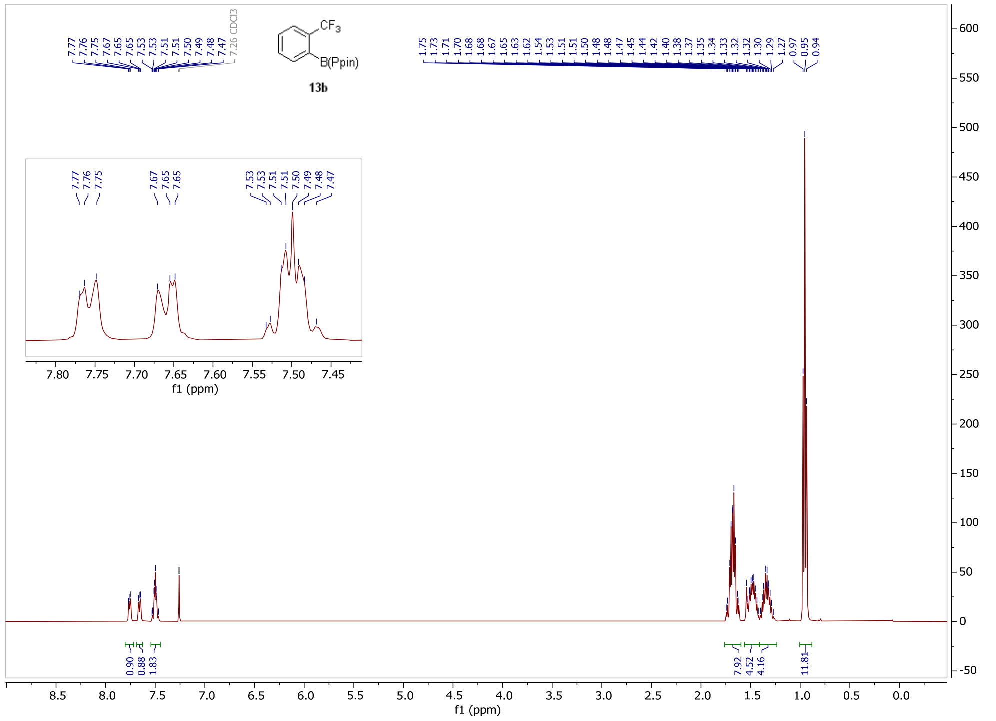

Figure S85: ^{1}H NMR spectrum of compound **13b** in $CDCl_3$.

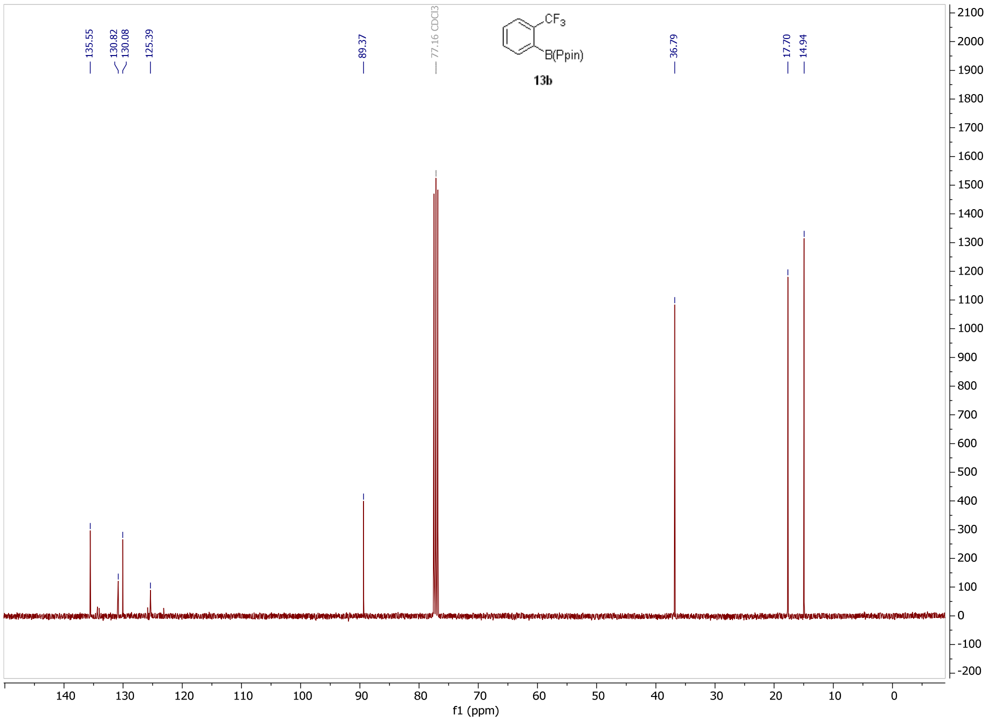

Figure S86: ^{13}C NMR spectrum of compound **13b** in $CDCl_3$.

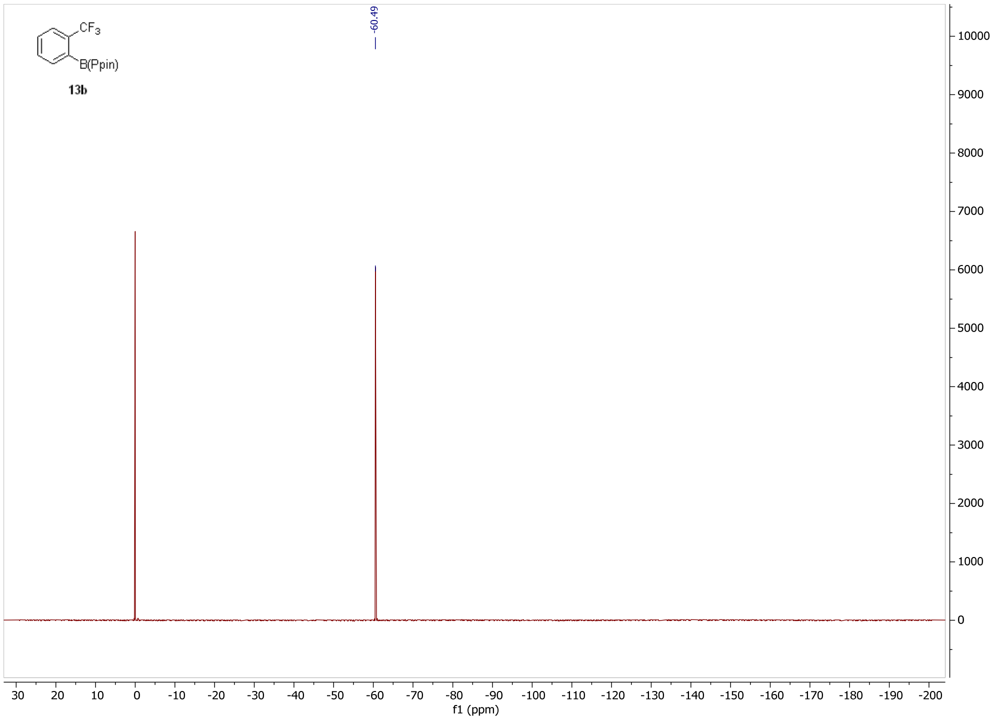

Figure S87: ^{19}F NMR spectrum of compound **13b** in $CDCl_3$.

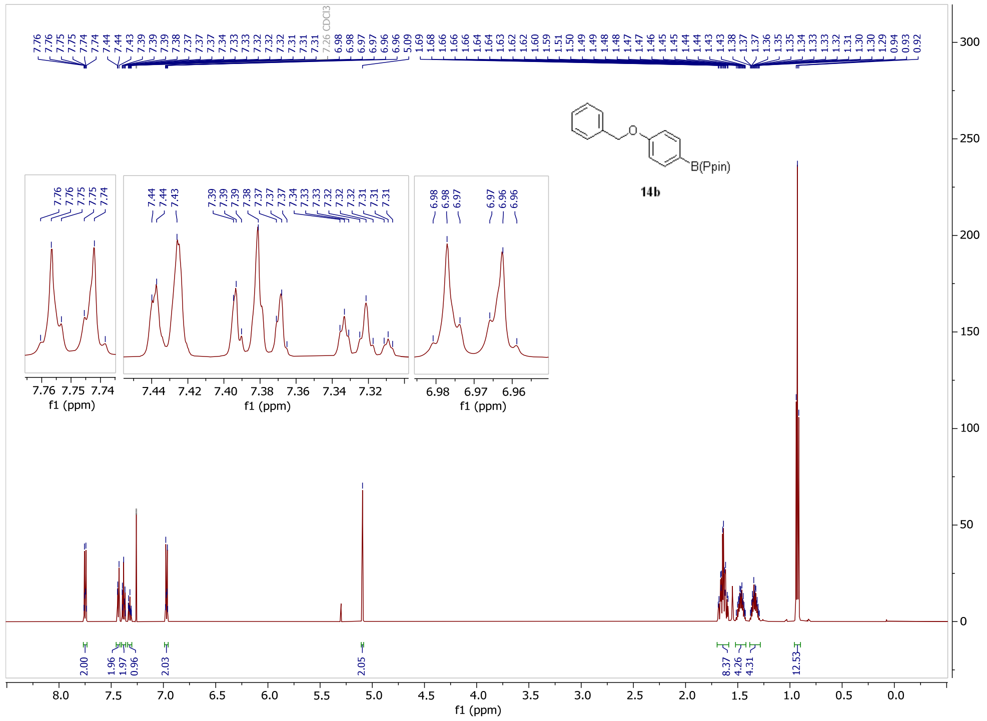

Figure S88: ^{1}H NMR spectrum of compound **14b** in $CDCl_3$.

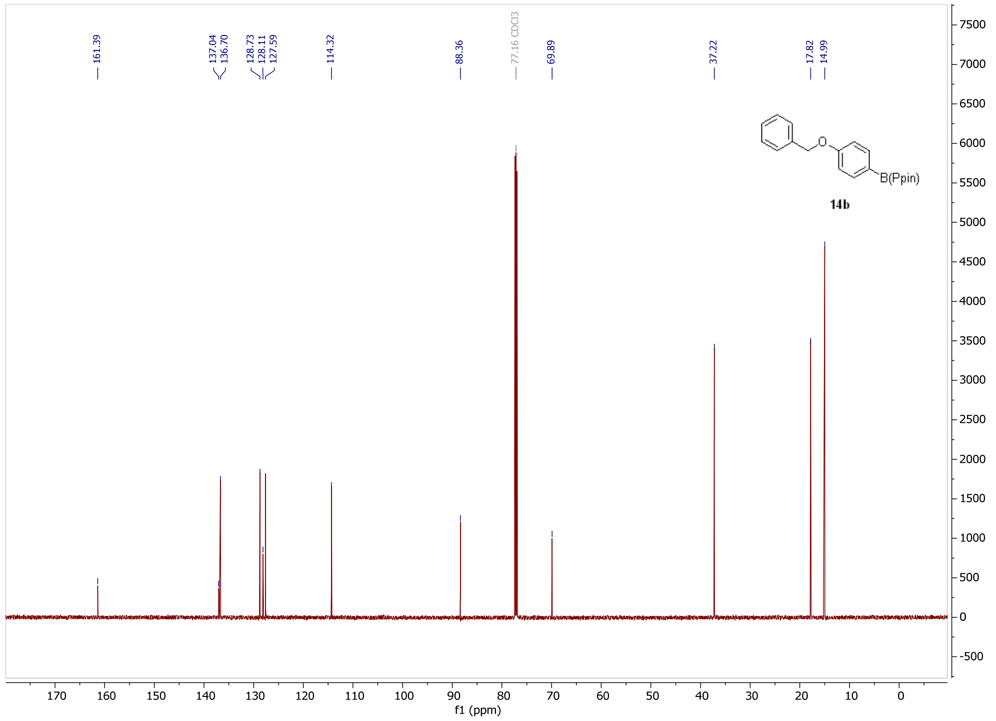

Figure S89: ^{13}C NMR spectrum of compound **14b** in $CDCl_3$.

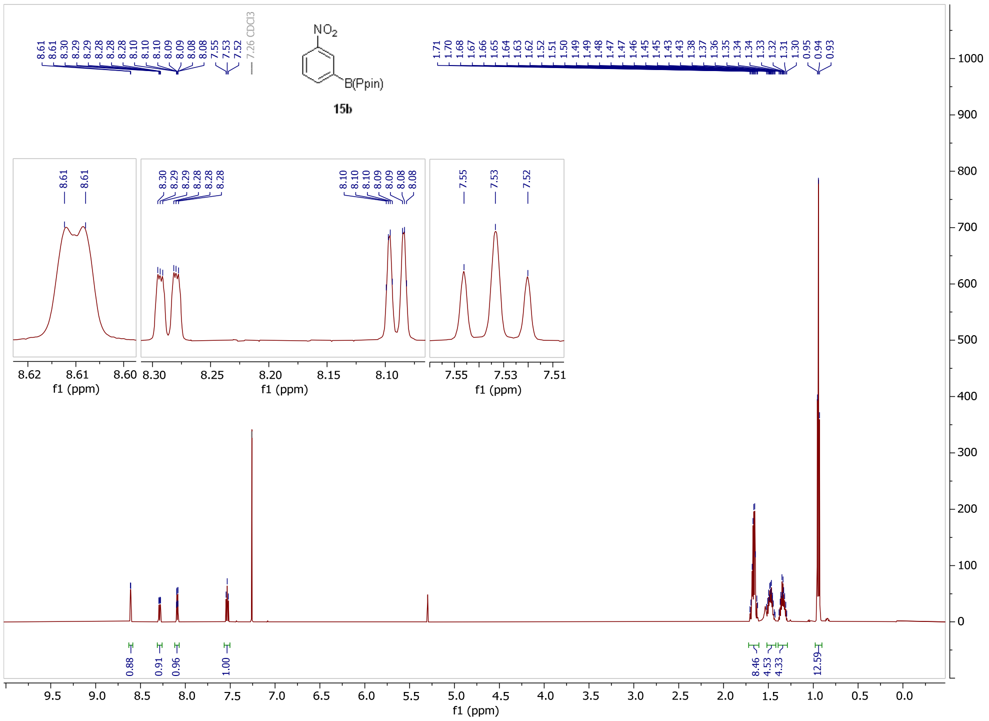

Figure S90: ^{1}H NMR spectrum of compound **15b** in $CDCl_3$.

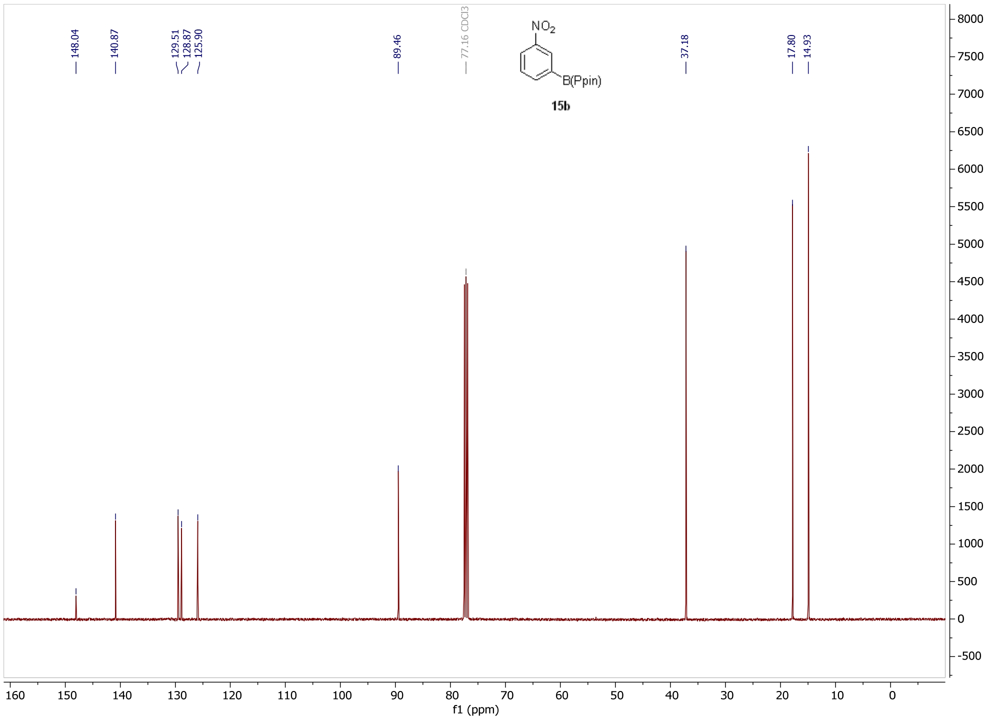

Figure S91: ^{13}C NMR spectrum of compound **15b** in $CDCl_3$.

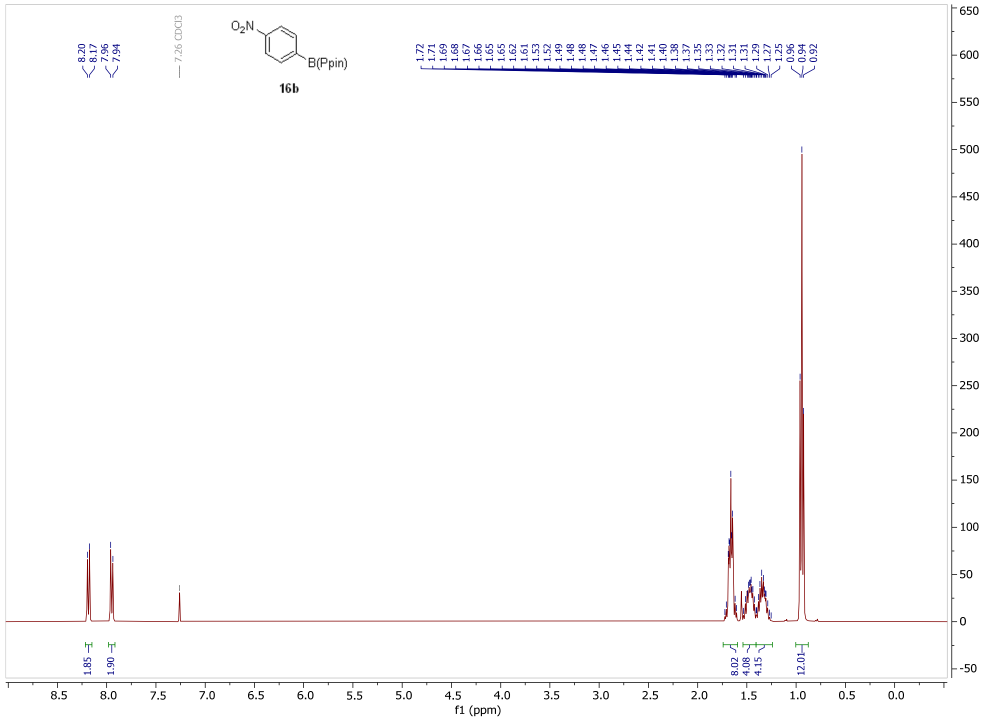

Figure S92: ^{1}H NMR spectrum of compound **16b** in $CDCl_3$.

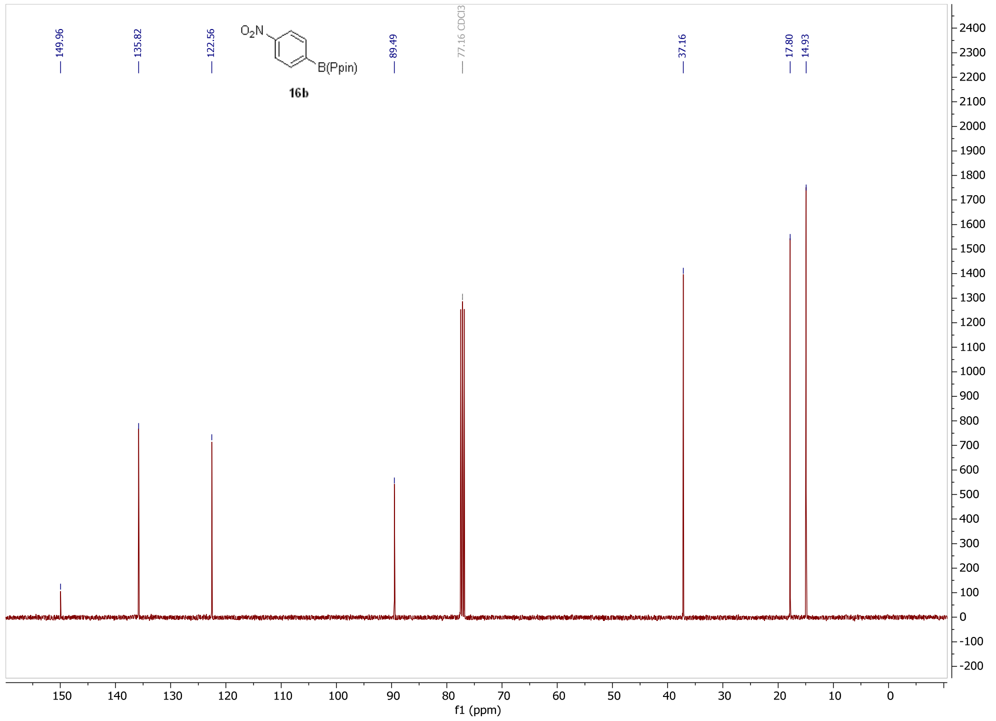

Figure S93: ^{13}C NMR spectrum of compound **16b** in $CDCl_3$.

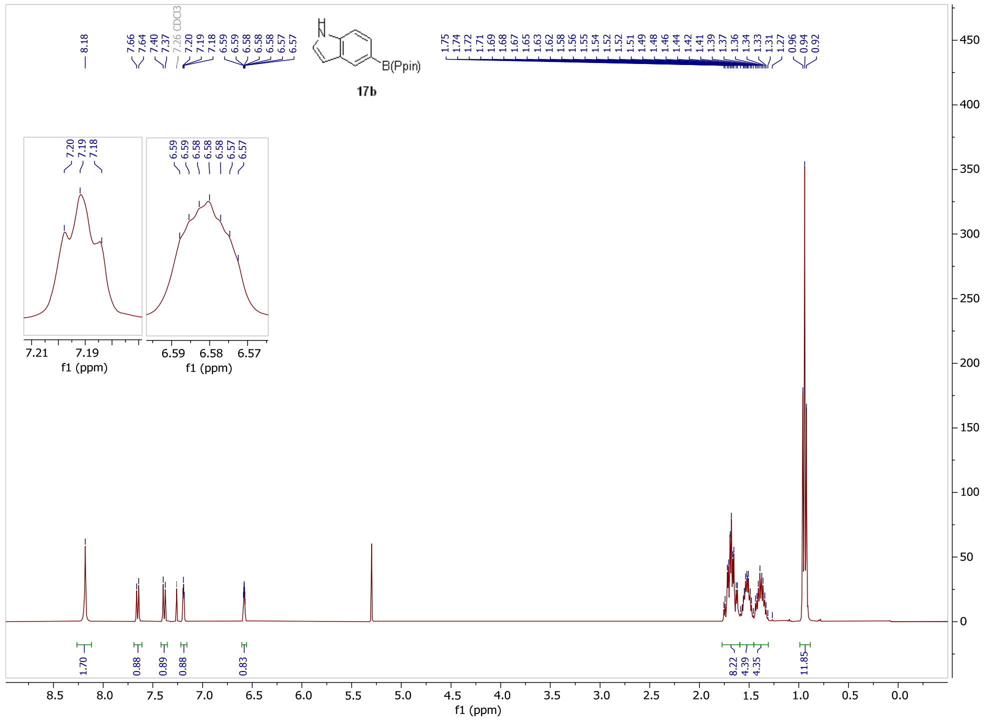

Figure S94: ^{1}H NMR spectrum of compound **17b** in $CDCl_3$.

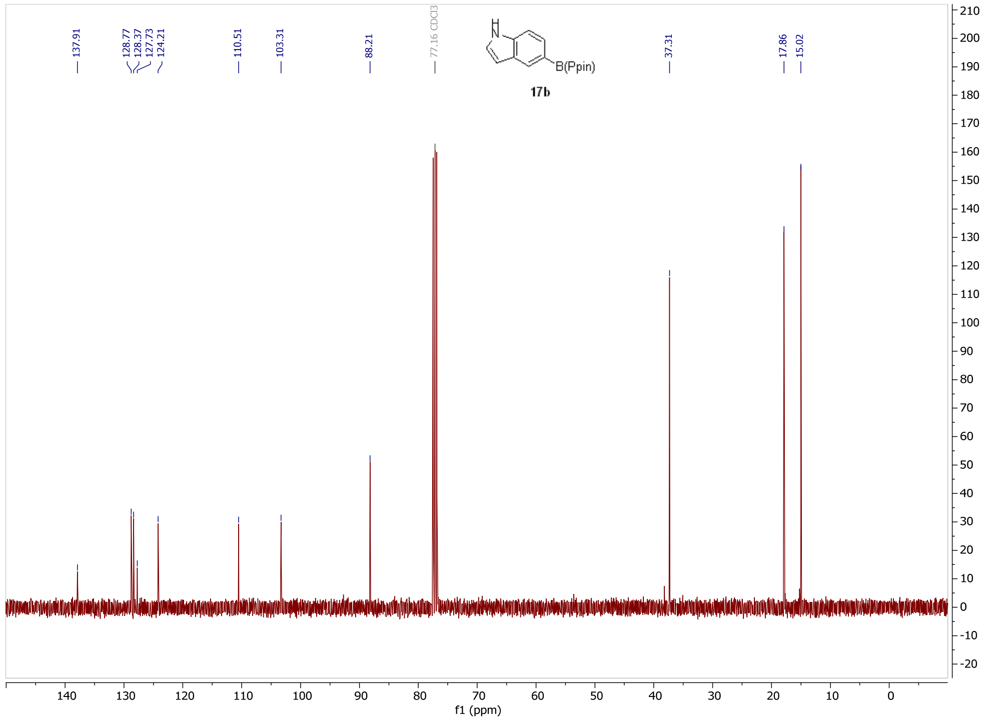

Figure S95: ^{13}C NMR spectrum of compound **17b** in $CDCl_3$.

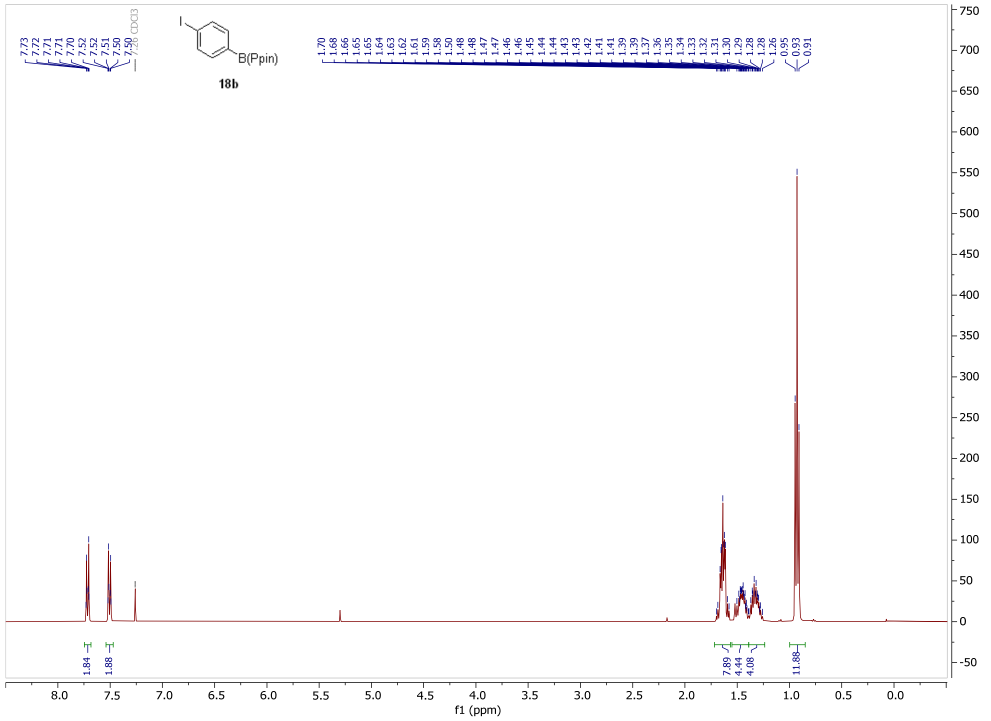

Figure S96: ^{13}C NMR spectrum of compound **18b** in $CDCl_3$.

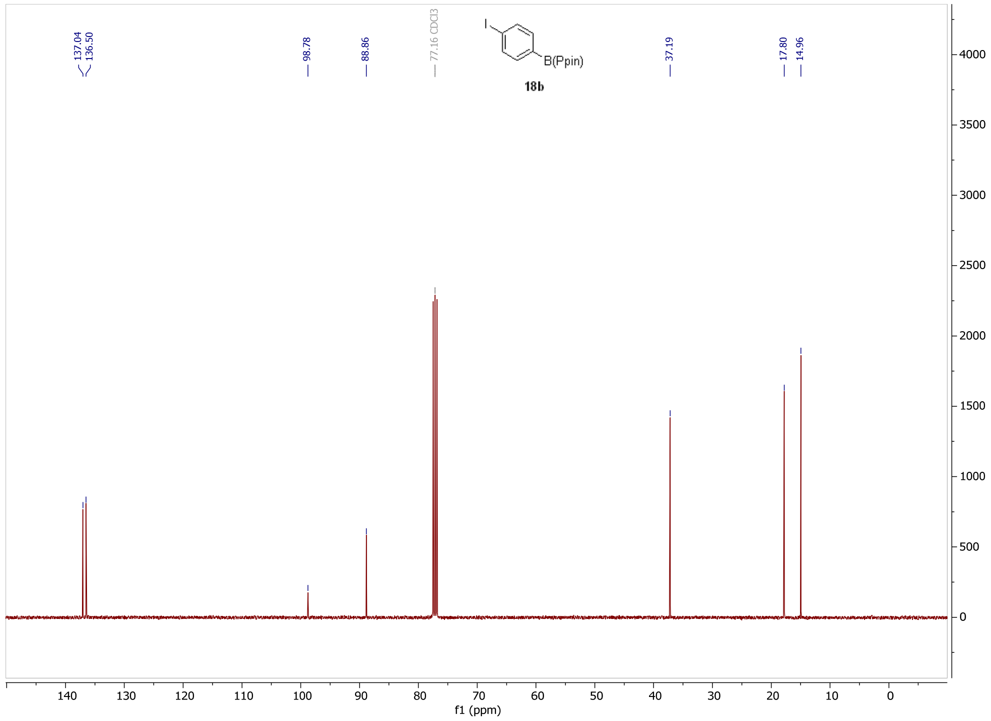

Figure S97: ^{13}C NMR spectrum of compound **18b** in $CDCl_3$.

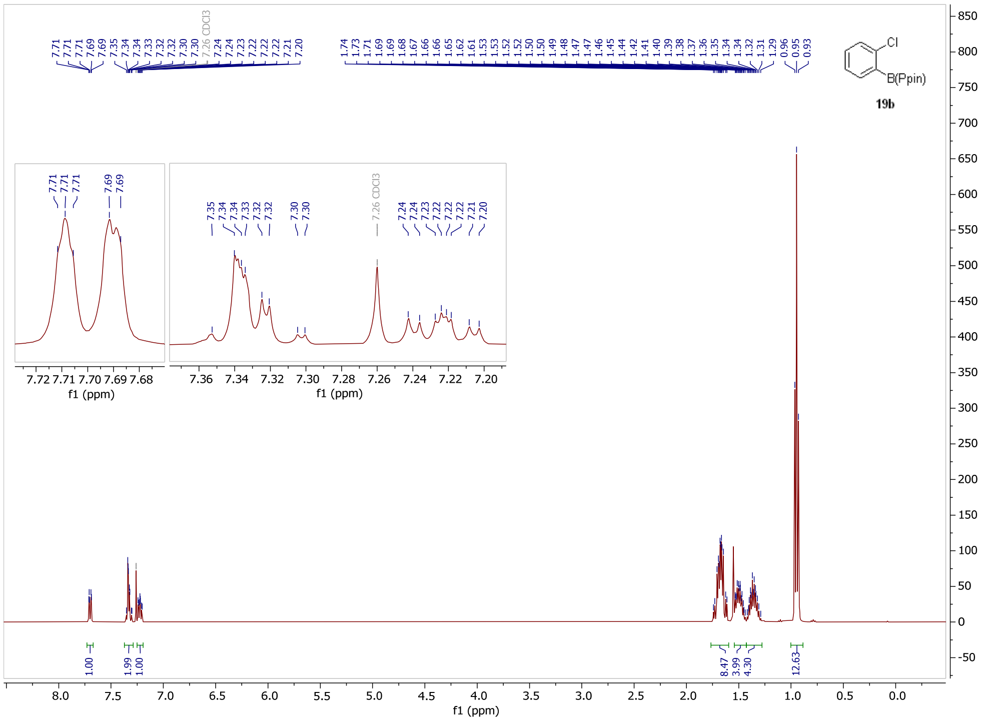

Figure S98: ^{1}H NMR spectrum of compound **19b** in $CDCl_3$.

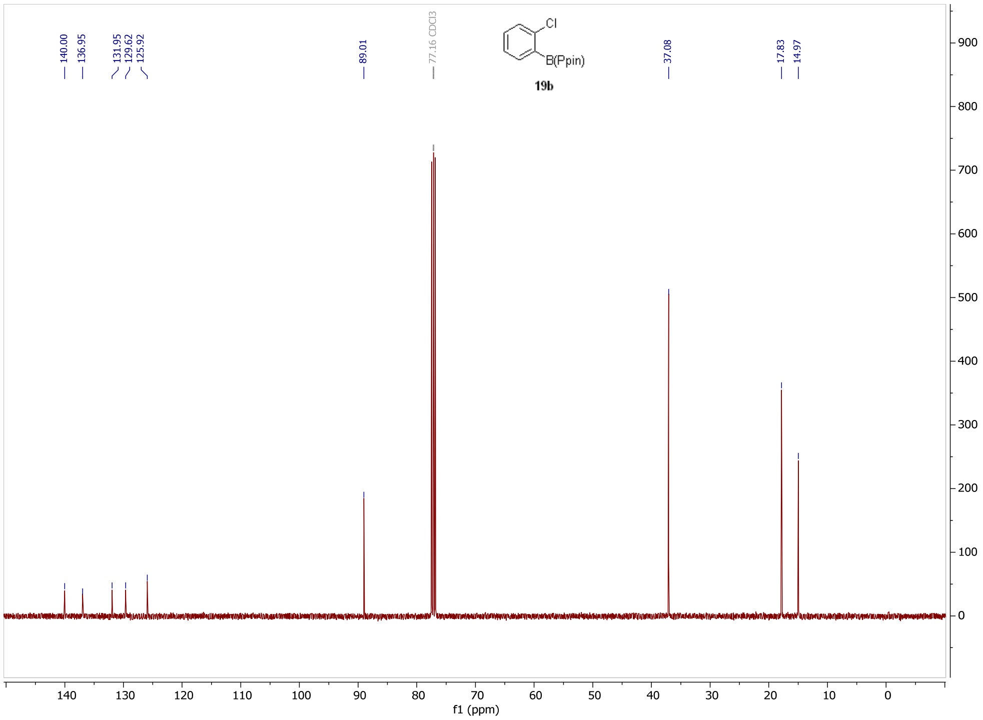

Figure S99: ^{13}C NMR spectrum of compound **19b** in $CDCl_3$.

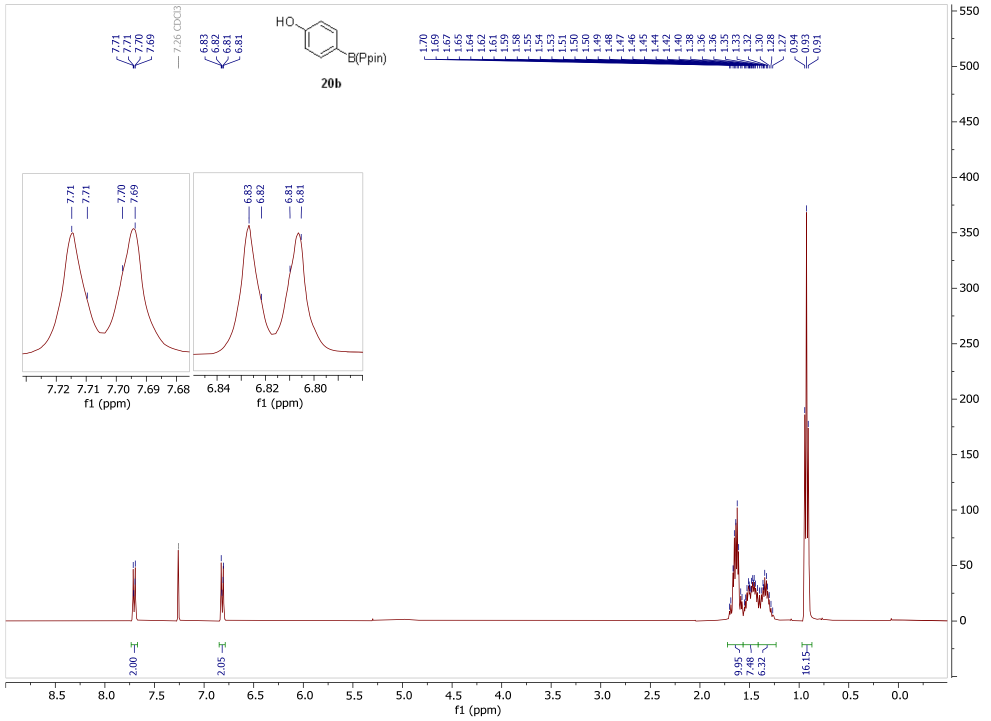

Figure S100: ^{1}H NMR spectrum of compound **20b** in $CDCl_3$.

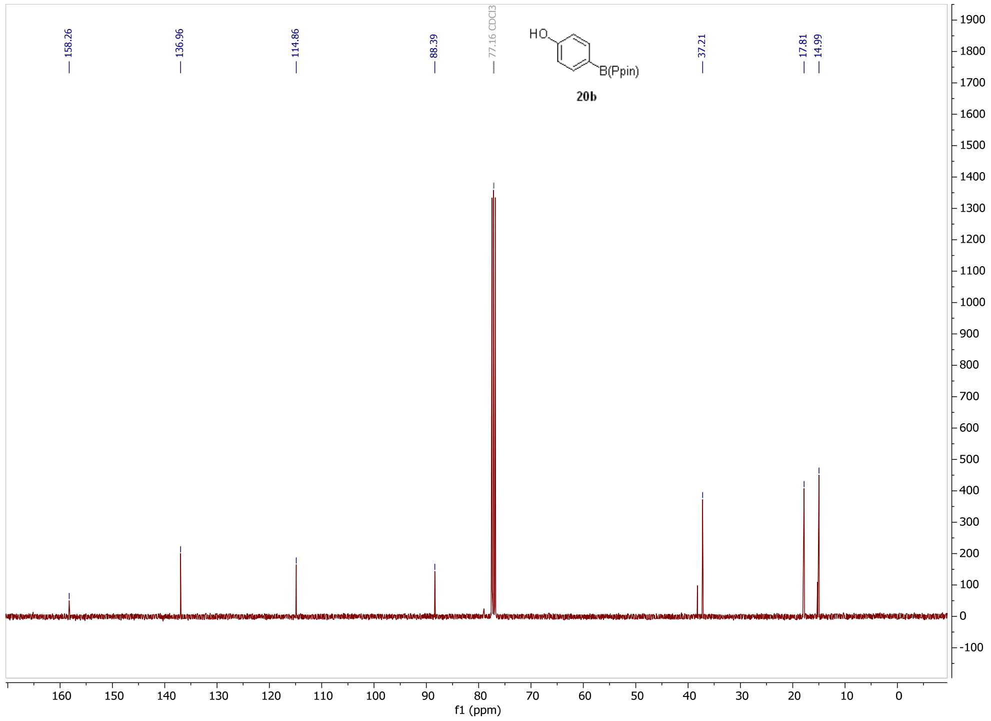

Figure S101: ^{13}C NMR spectrum of compound **20b** in $CDCl_3$.

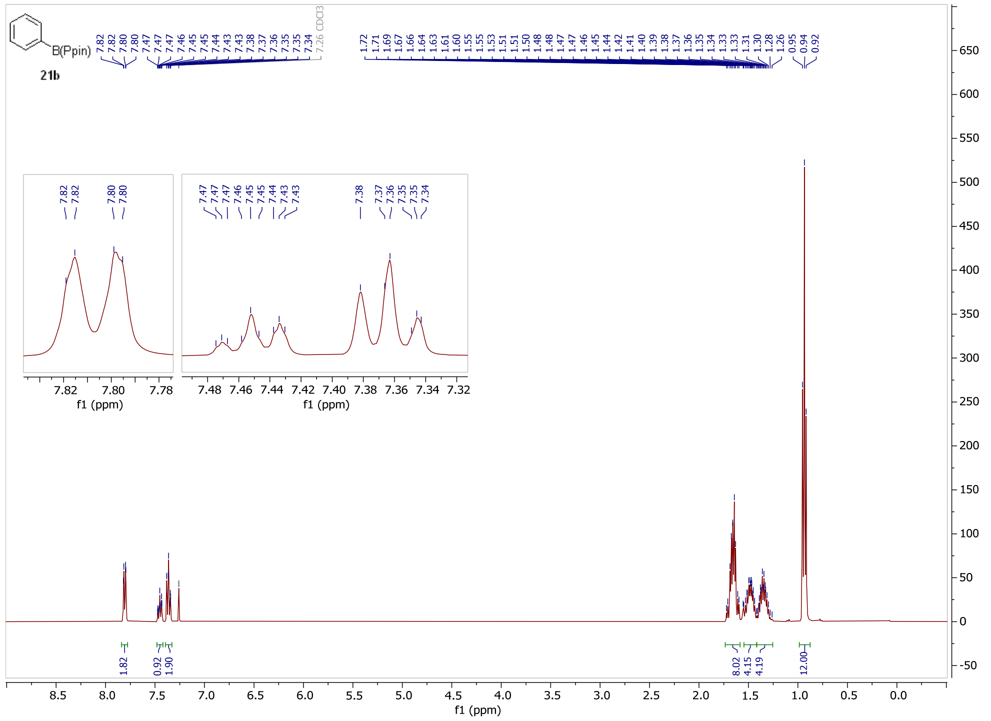

Figure S102: ^{1}H NMR spectrum of compound **21b** in $CDCl_3$.

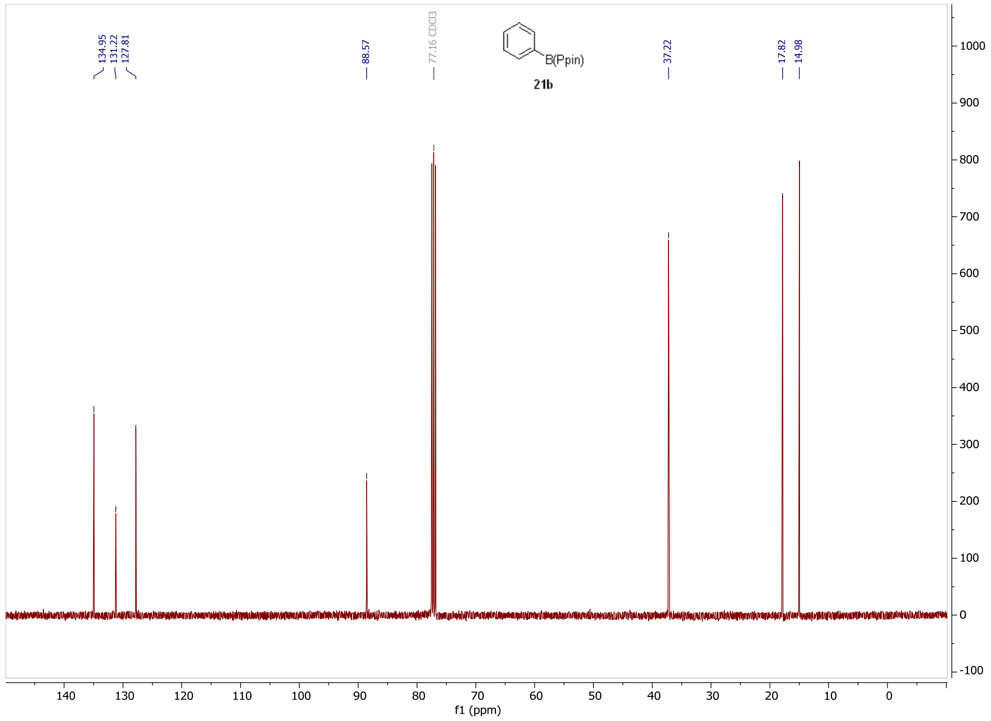

Figure S103: ^{13}C NMR spectrum of compound **21b** in $CDCl_3$.

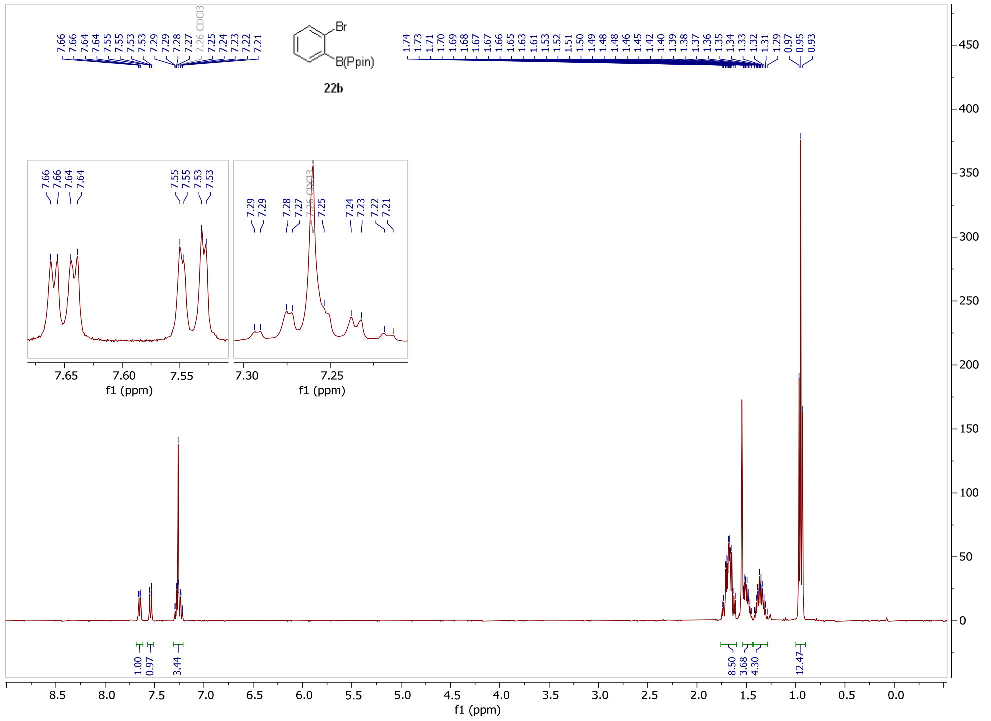

Figure S104: ^{1}H NMR spectrum of compound **22b** in $CDCl_3$.

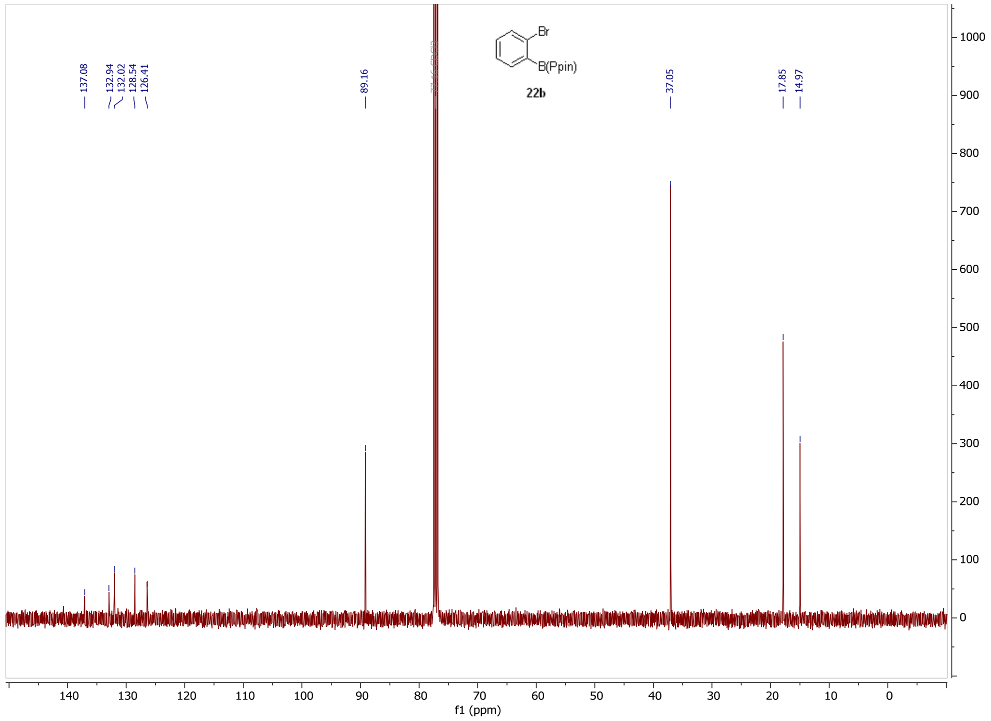

Figure S105: ^{13}C NMR spectrum of compound **22b** in $CDCl_3$.

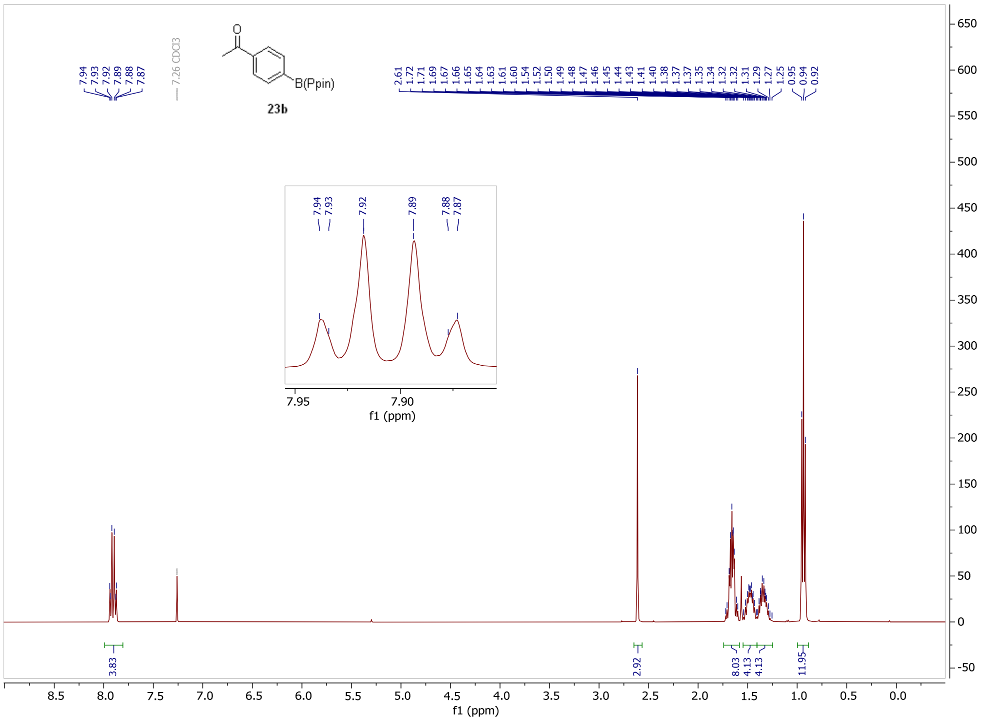

Figure S106: [13]H NMR spectrum of compound **23b** in $CDCl_3$.

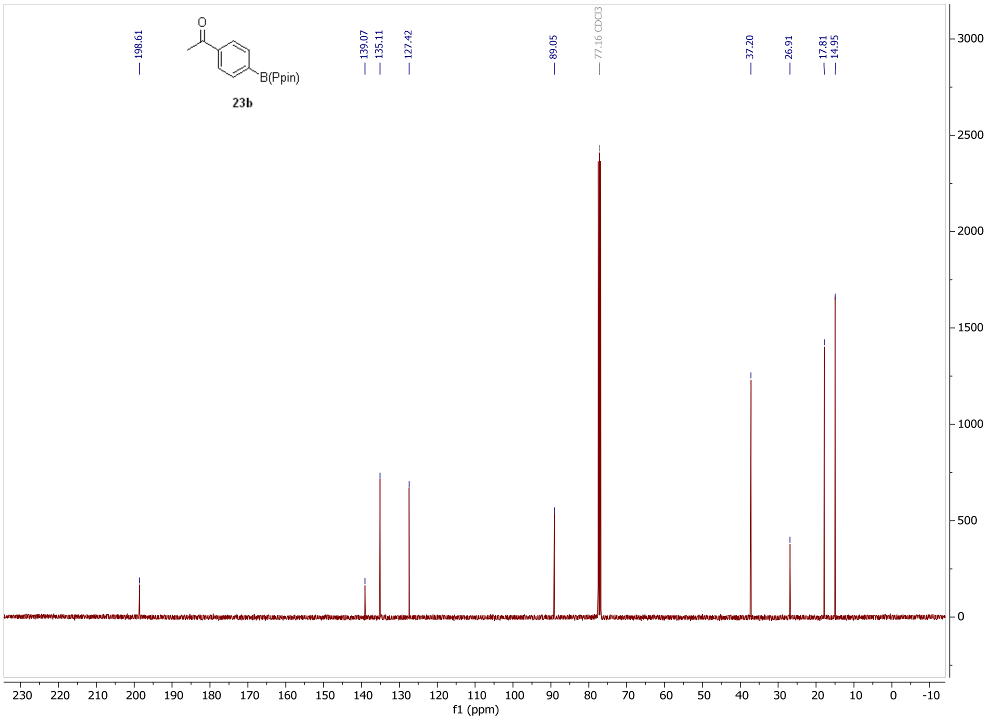

Figure S107: ^{13}C NMR spectrum of compound **23b** in $CDCl_3$.

IR-Spectra of Novel Compounds

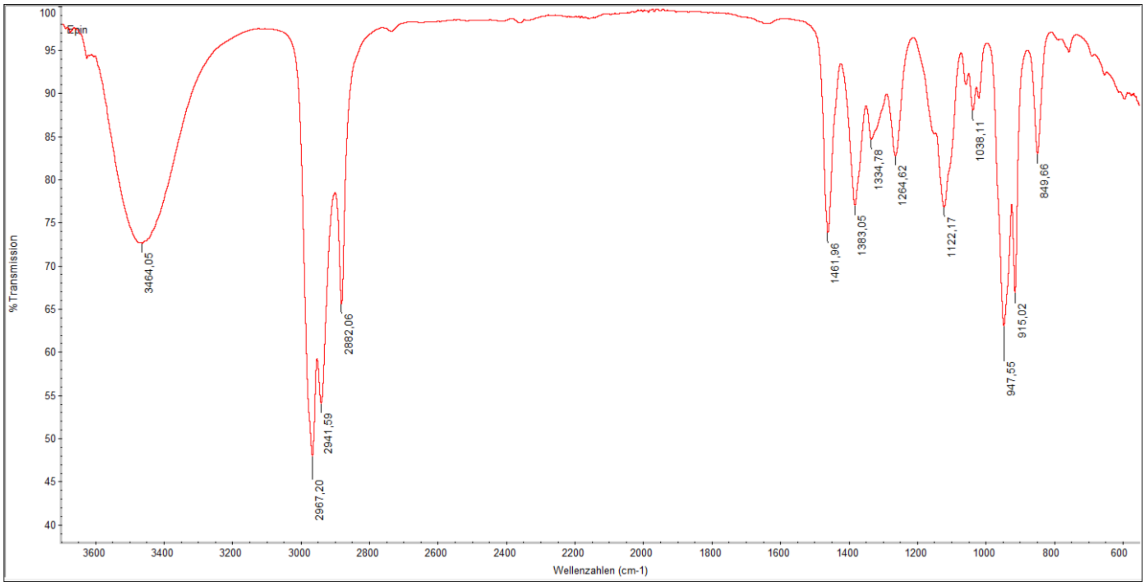

Figure S108: ATR FT-IR spectrum of compound **2**.

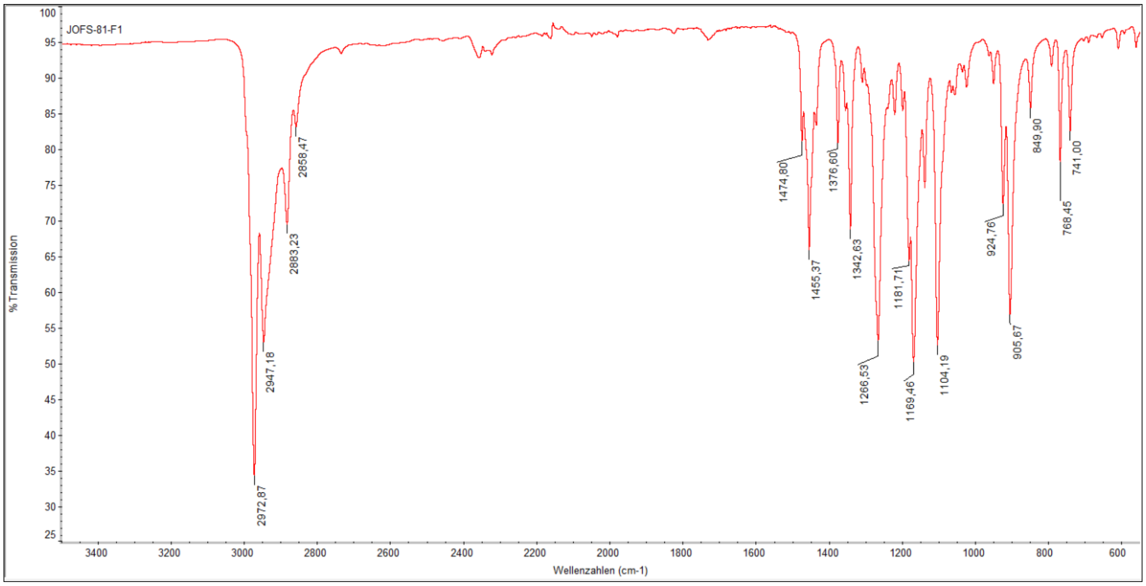

Figure S109: ATR FT-IR spectrum of compound **S1**.

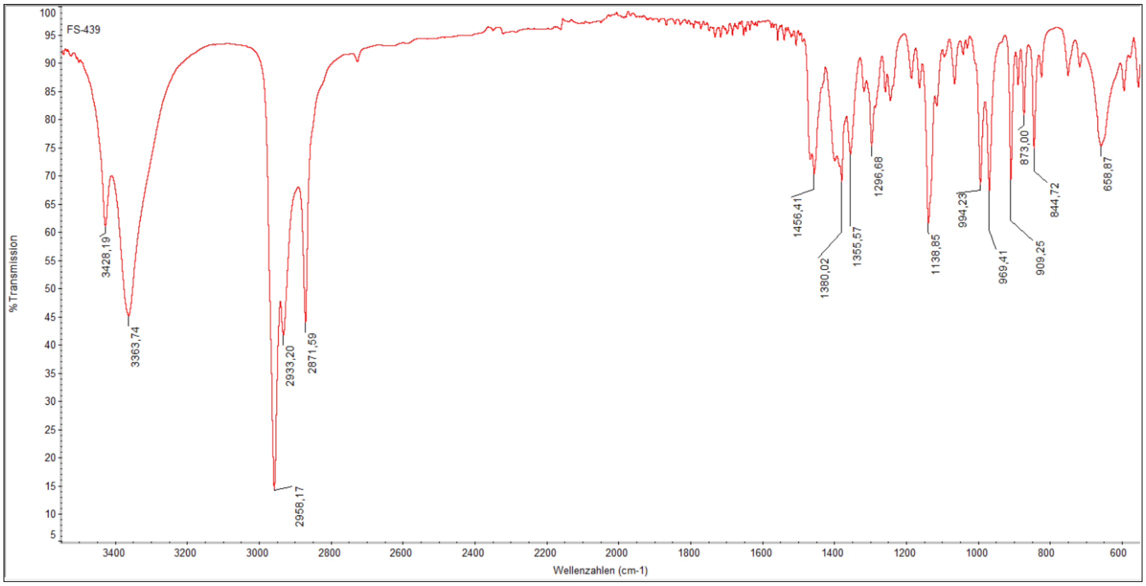

Figure S110: ATR FT-IR spectrum of compound **4**.

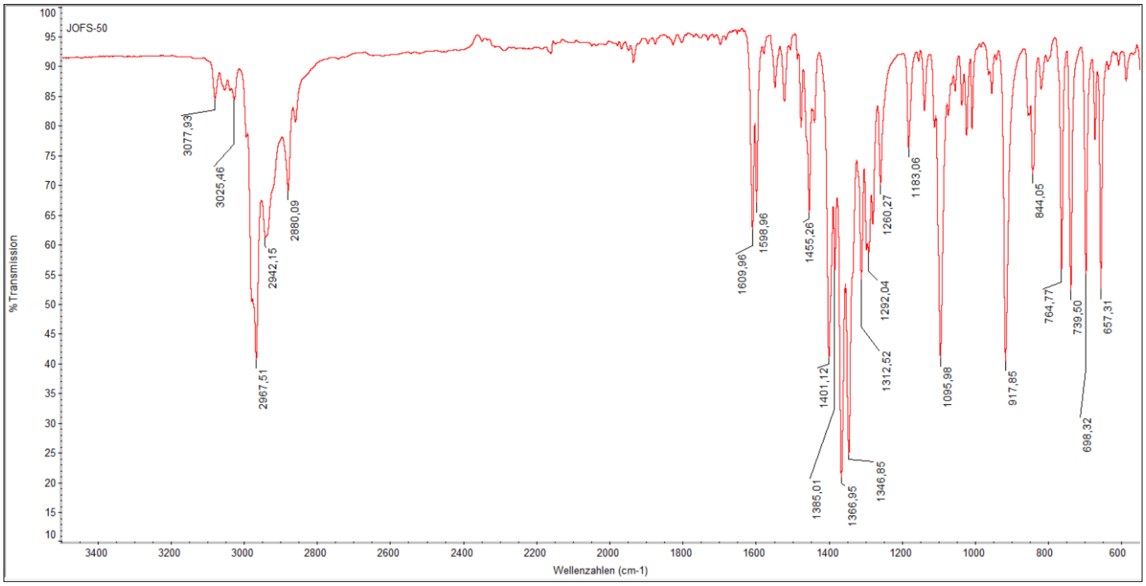

Figure S111: ATR FT-IR spectrum of compound **5a**.

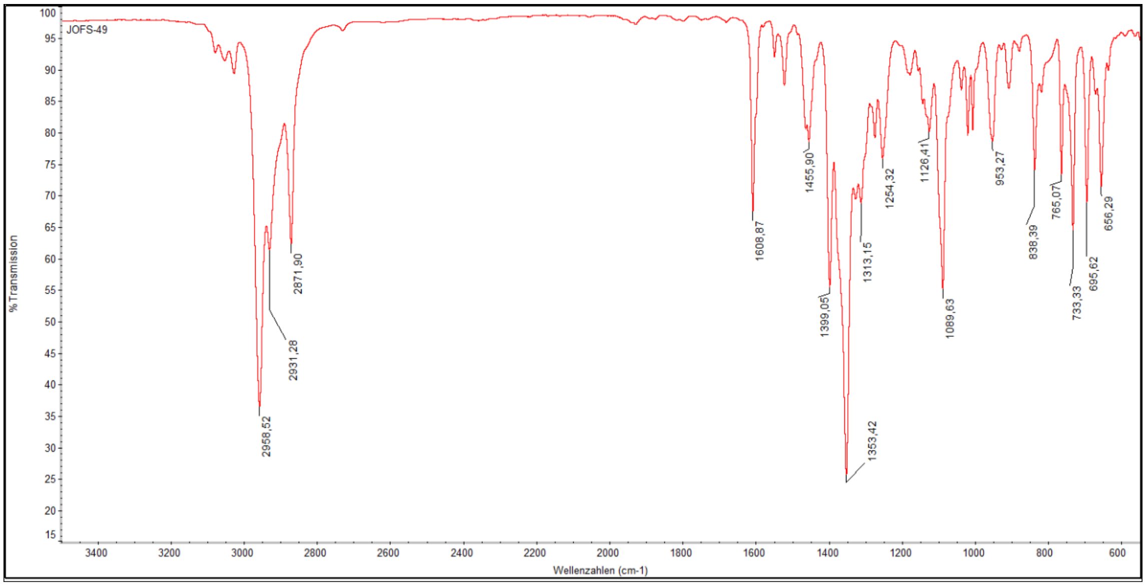

Figure S112: ATR FT-IR spectrum of compound **5b**.

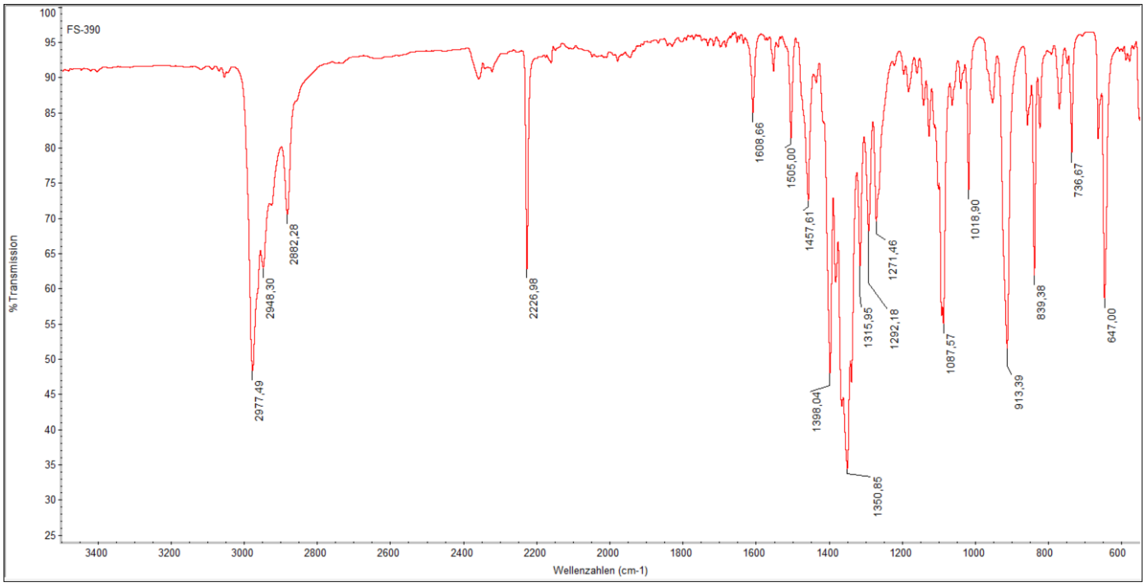

Figure S113: ATR FT-IR spectrum of compound **6a**.

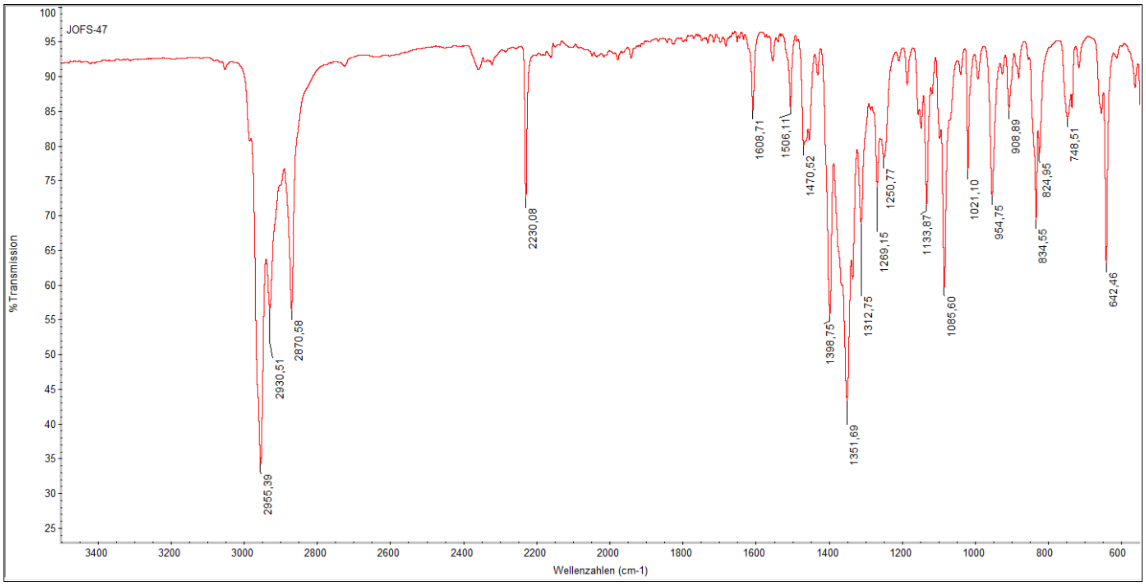

Figure S114: ATR FT-IR spectrum of compound **6b**.

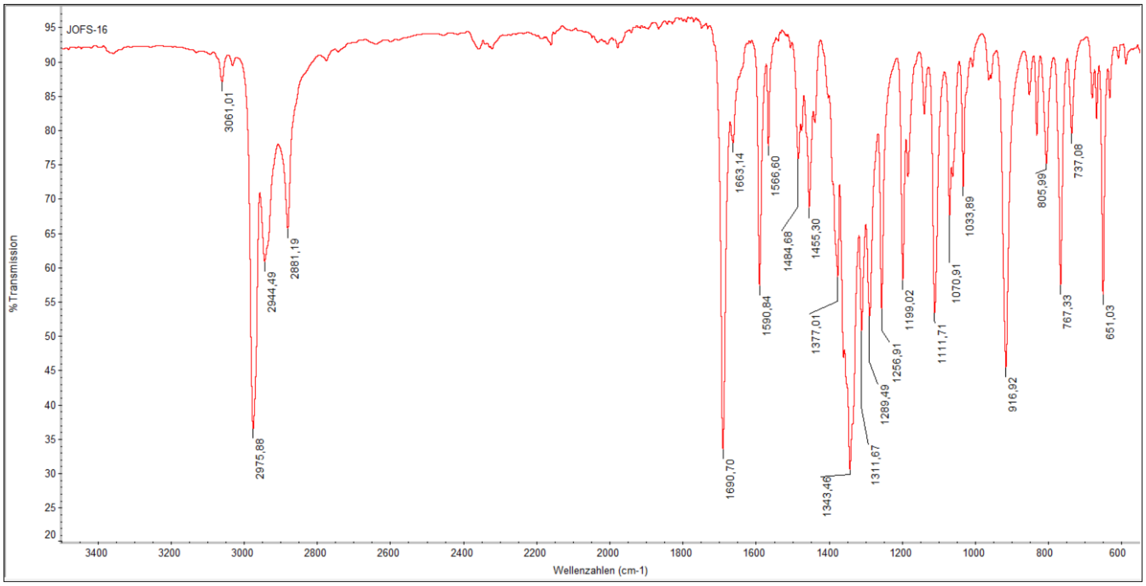

Figure S115: ATR FT-IR spectrum of compound **7a**.

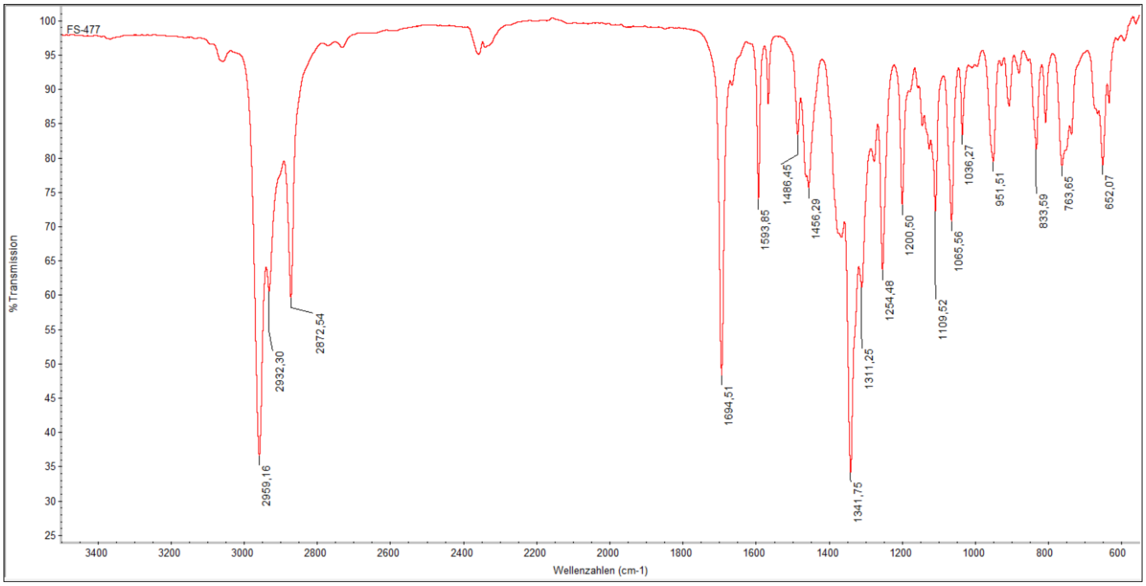

Figure S116: ATR FT-IR spectrum of compound **7b**.

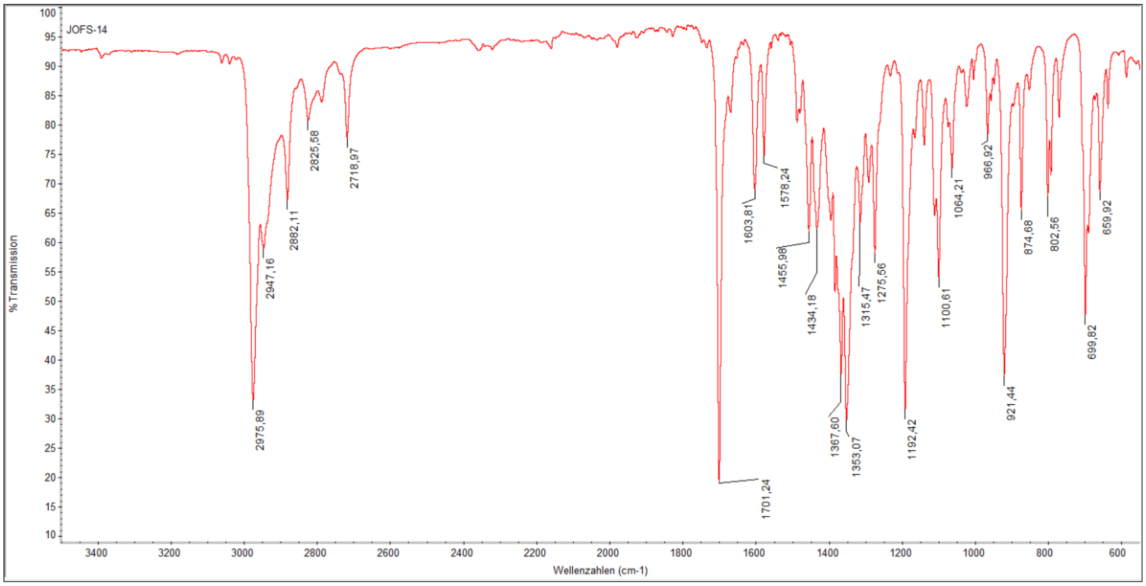

Figure S117: ATR FT-IR spectrum of compound **8a**.

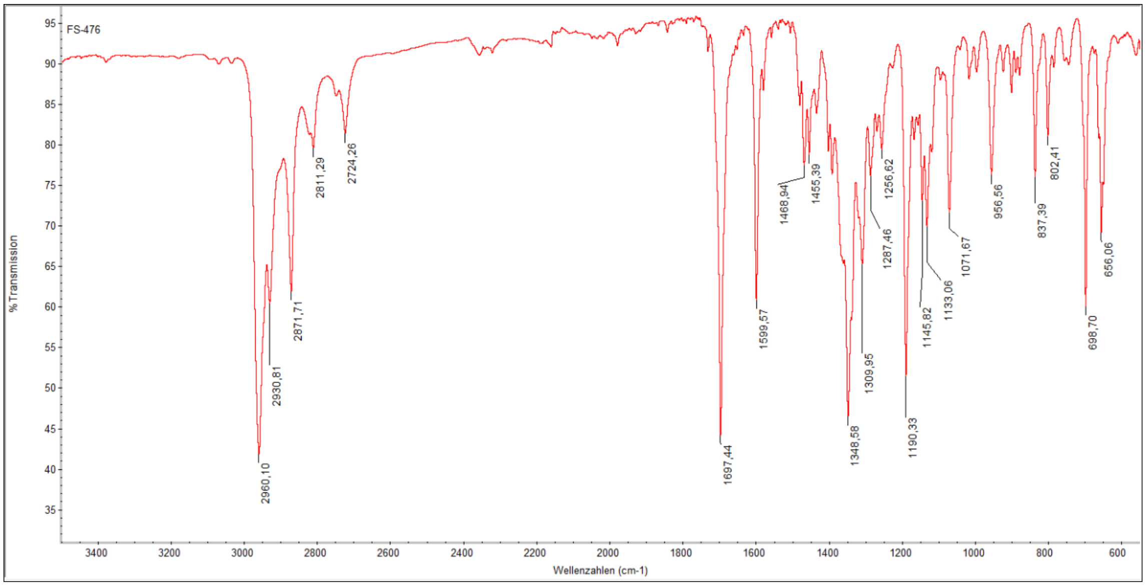

Figure S118: ATR FT-IR spectrum of compound **8b**.

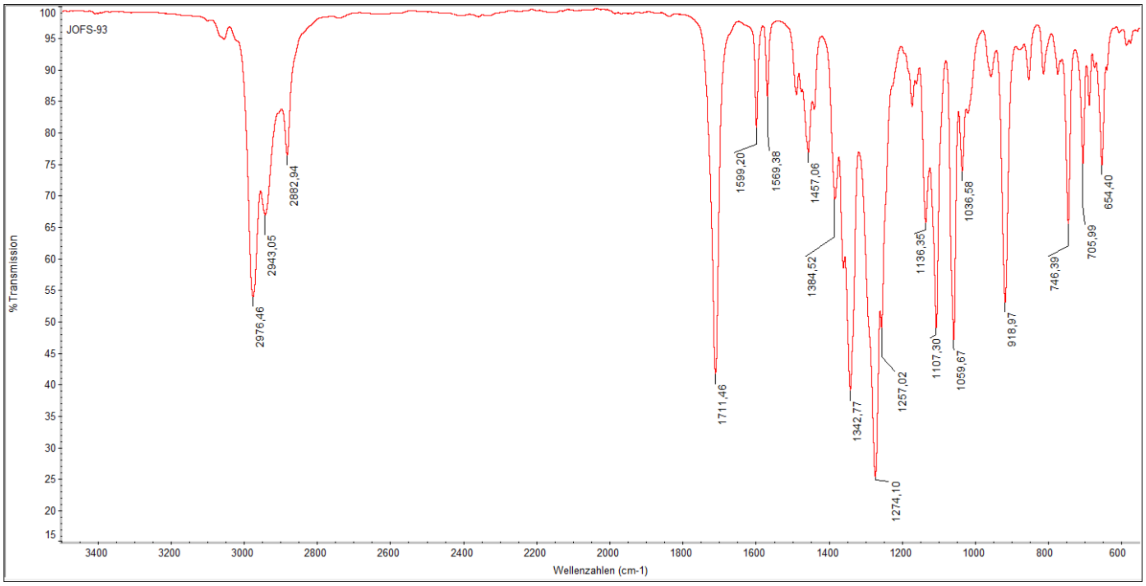

Figure S119: ATR FT-IR spectrum of compound **9a**.

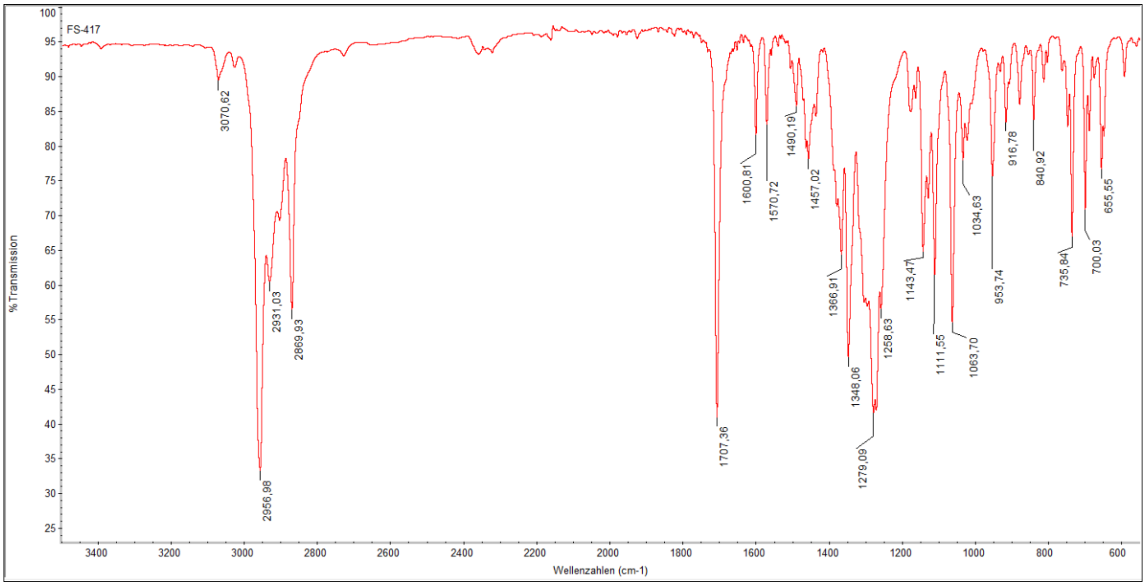

Figure S120: ATR FT-IR spectrum of compound **9b**.

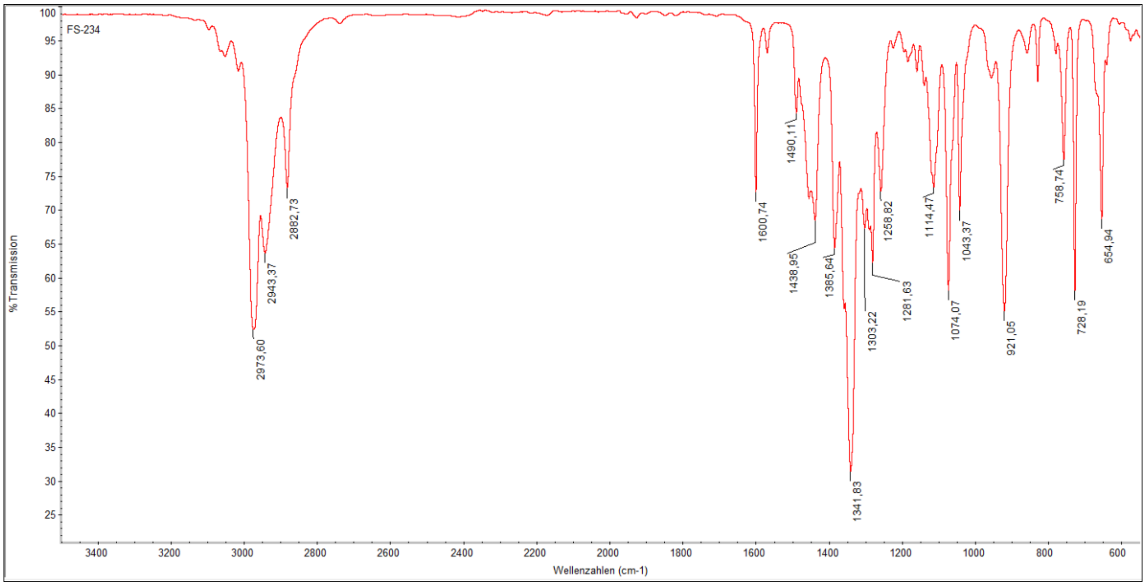

Figure S121: ATR FT-IR spectrum of compound **10a**.

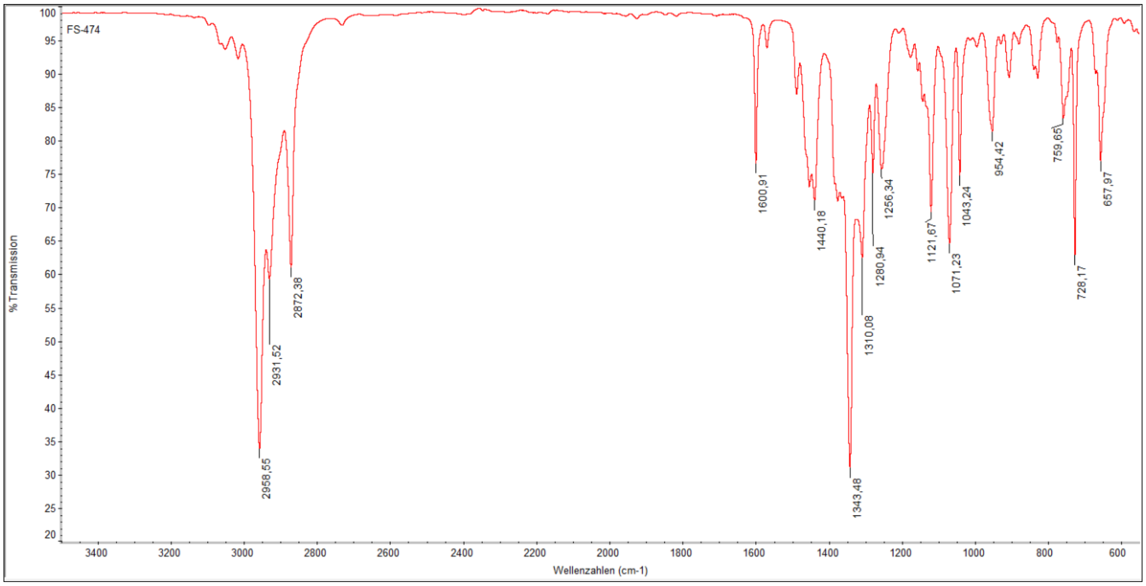

Figure S122: ATR FT-IR spectrum of compound **10b**.

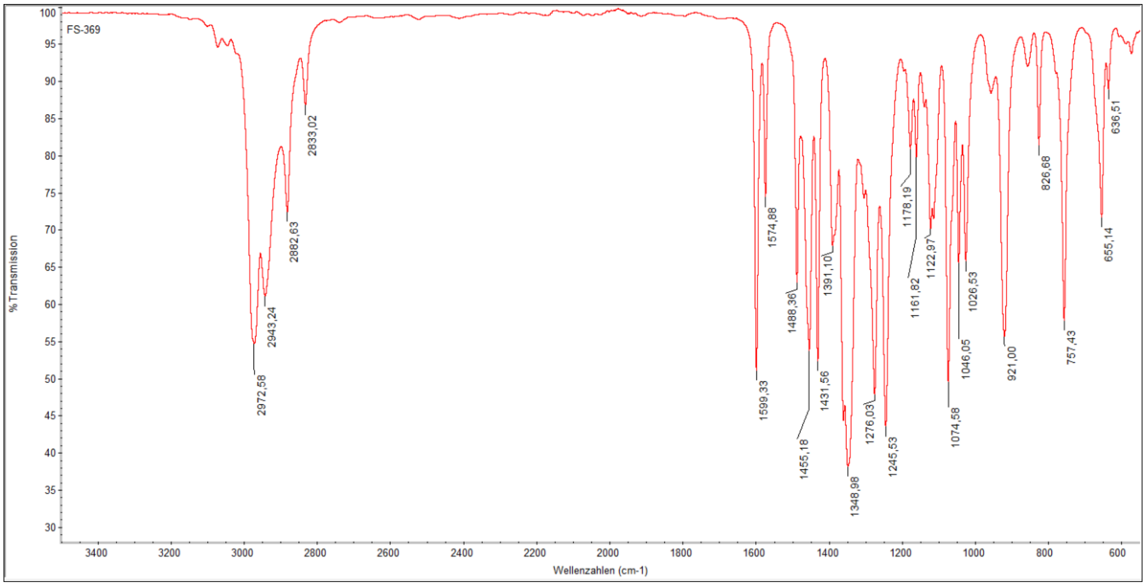

Figure S123: ATR FT-IR spectrum of compound **11a**.

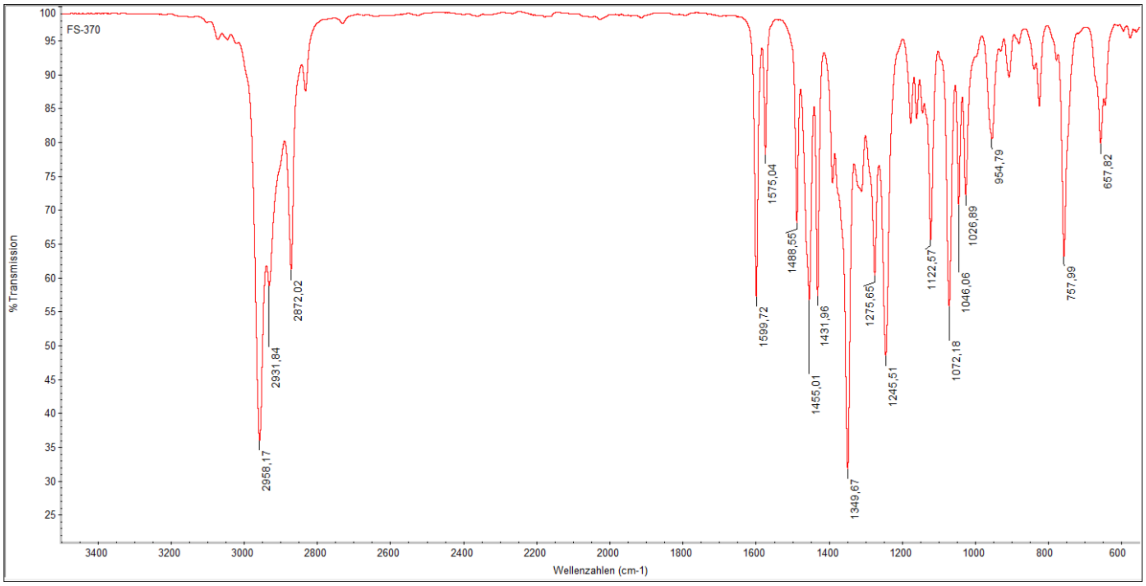

Figure S124: ATR FT-IR spectrum of compound **11b**.

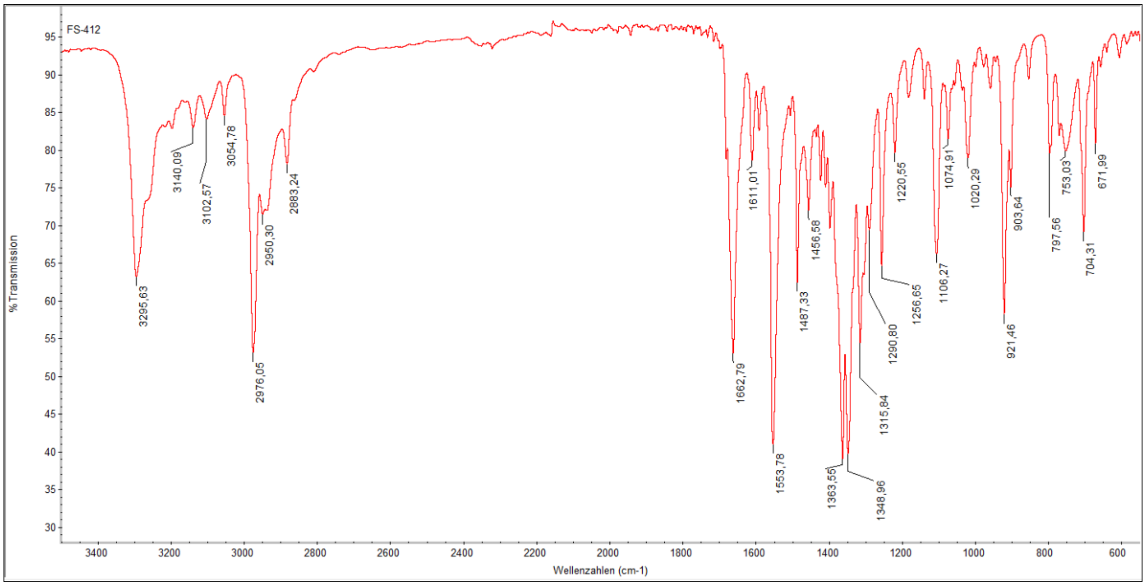

Figure S125: ATR FT-IR spectrum of compound **12a**.

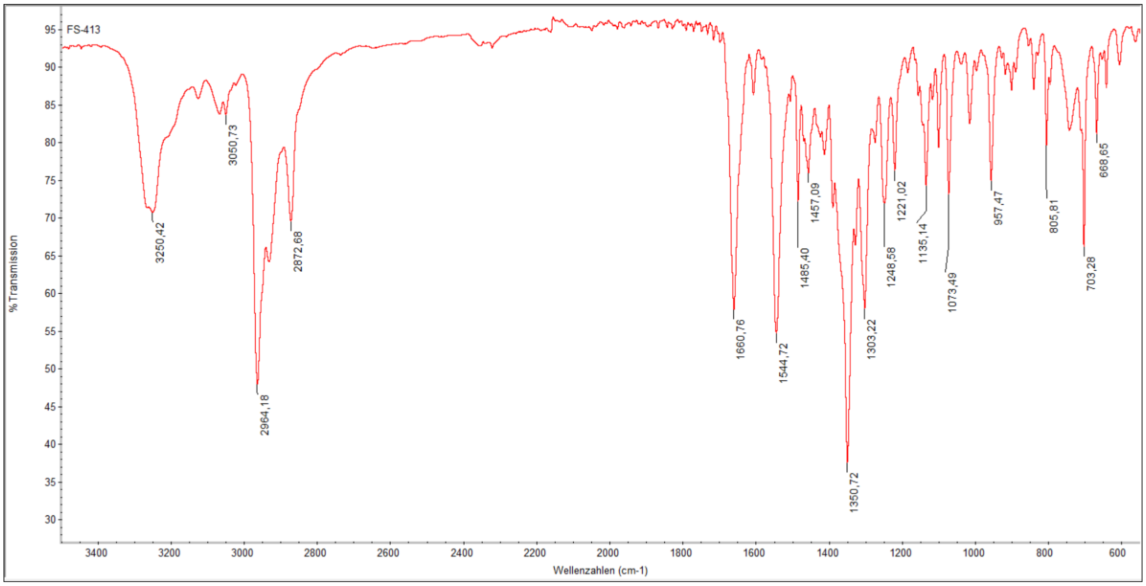

Figure S126: ATR FT-IR spectrum of compound **12b**.

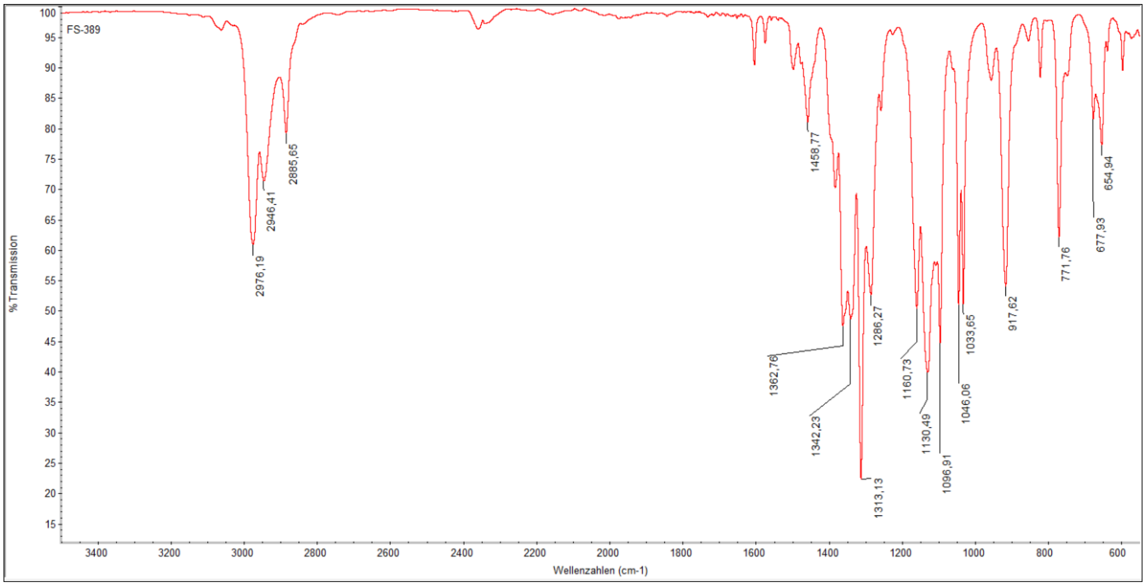

Figure S127: ATR FT-IR spectrum of compound **13a**.

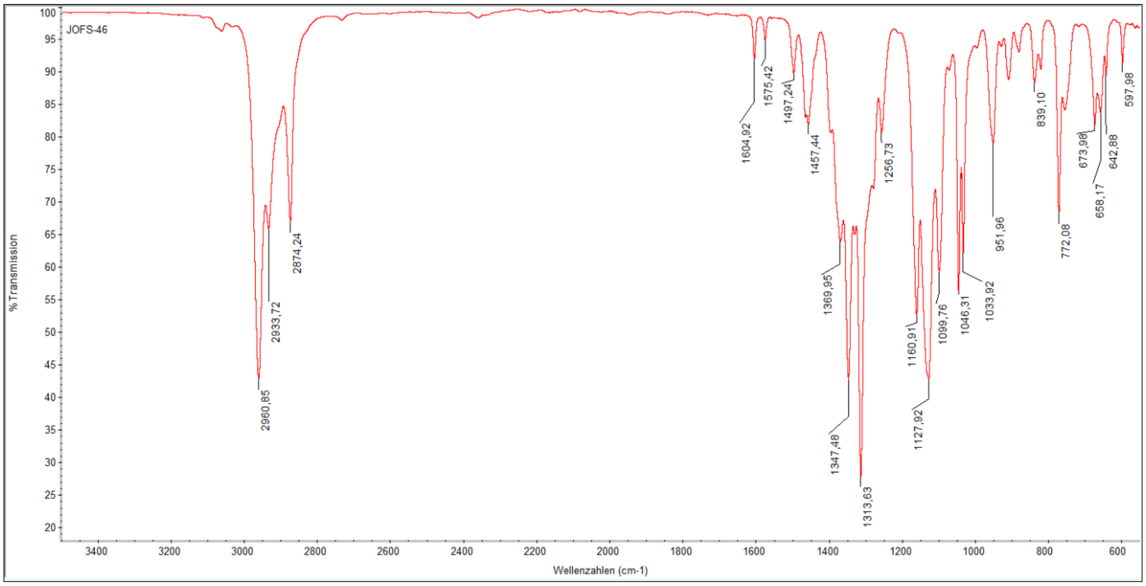

Figure S128: ATR FT-IR spectrum of compound **13b**.

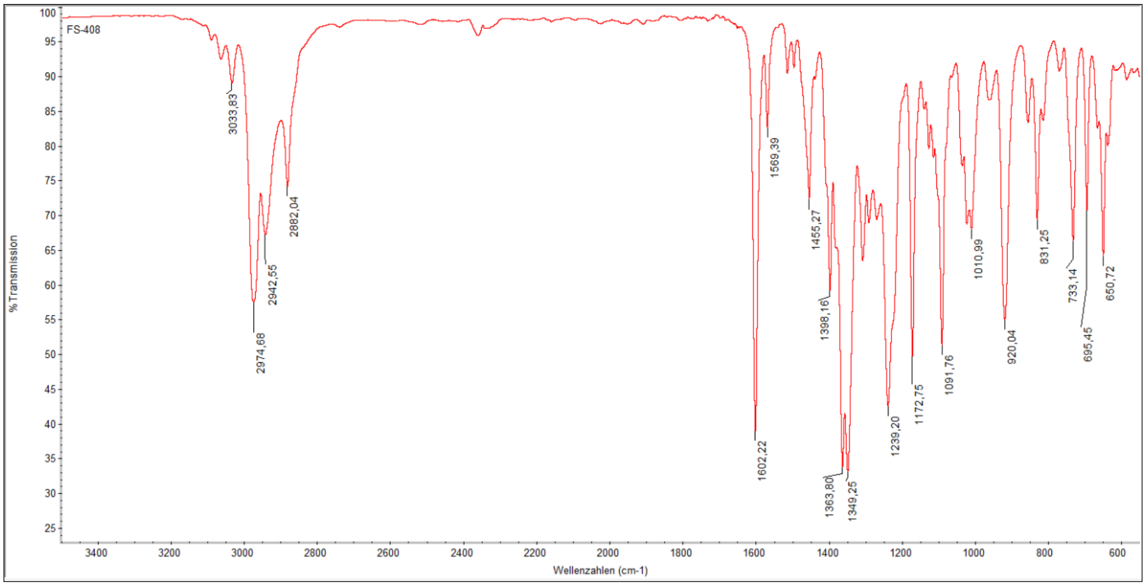

Figure S129: ATR FT-IR spectrum of compound **14a**.

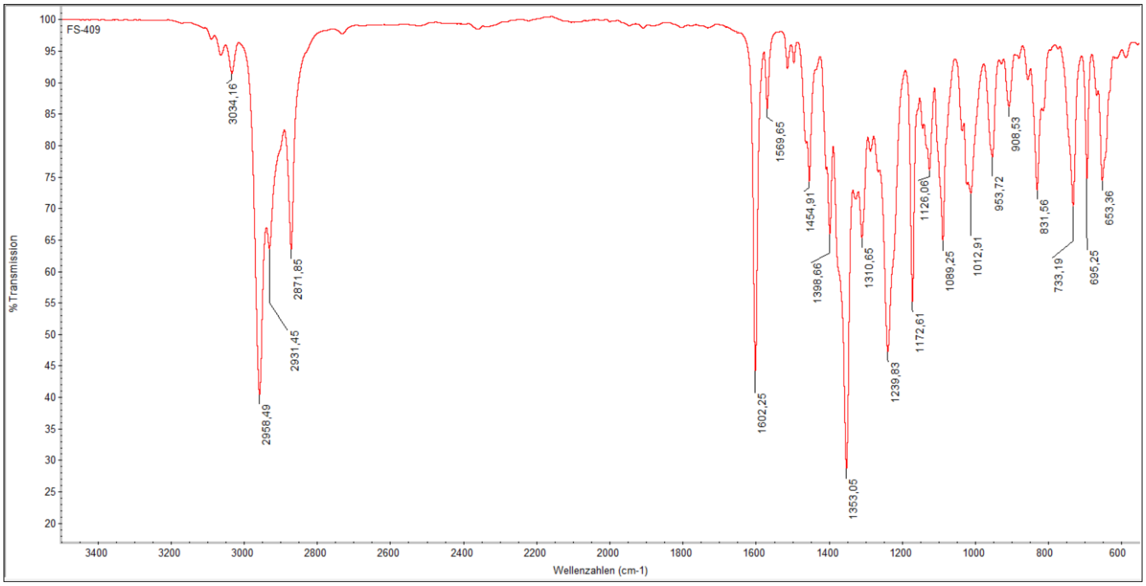

Figure S130: ATR FT-IR spectrum of compound **14b**.

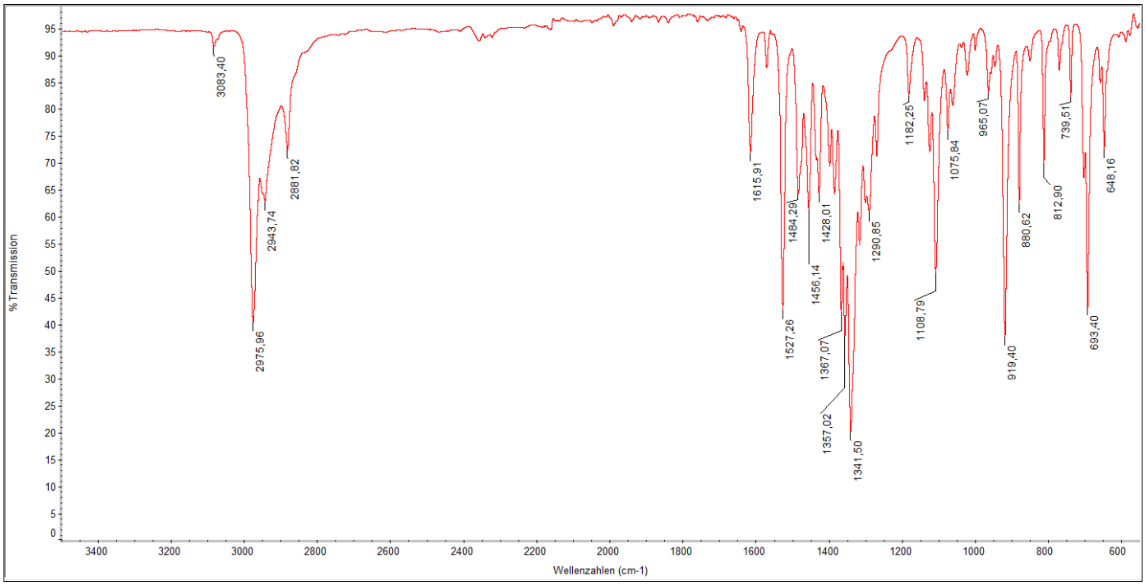

Figure S131: ATR FT-IR spectrum of compound **15a**.

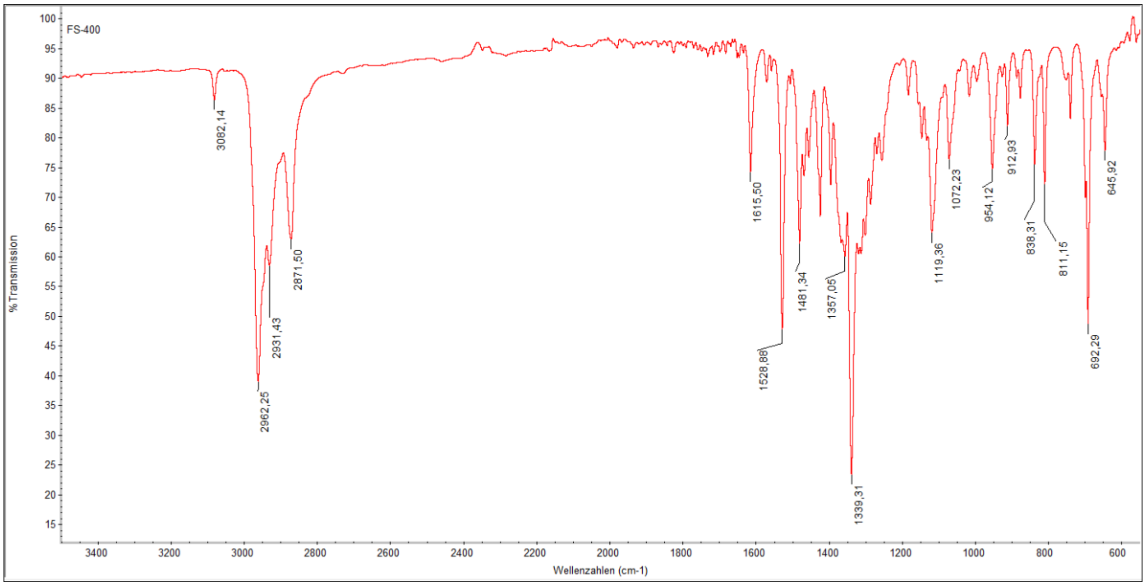

Figure S132: ATR FT-IR spectrum of compound **15b**.

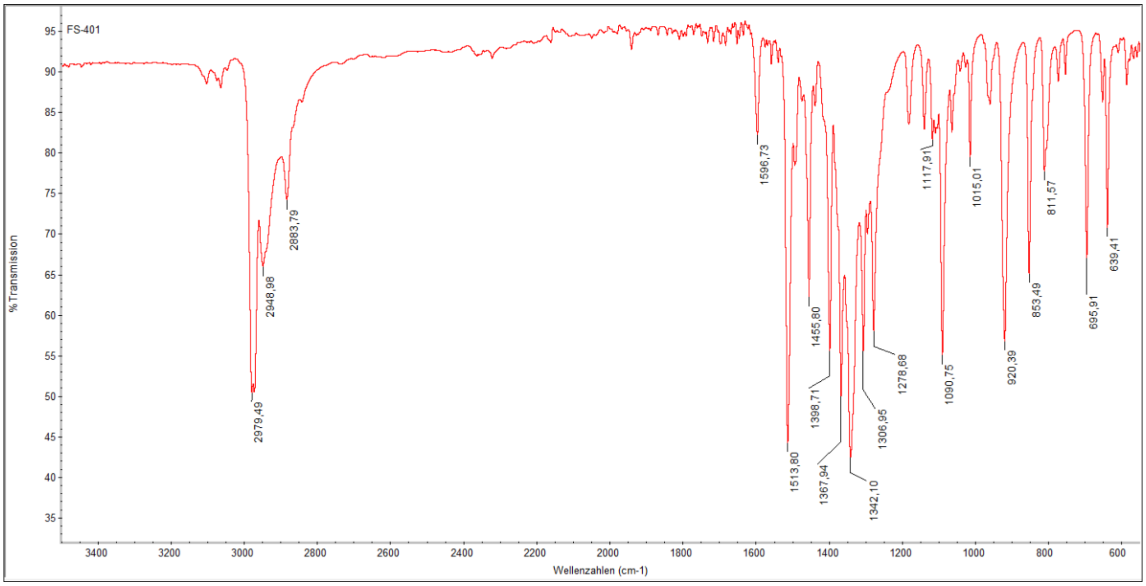

Figure S133: ATR FT-IR spectrum of compound **16a**.

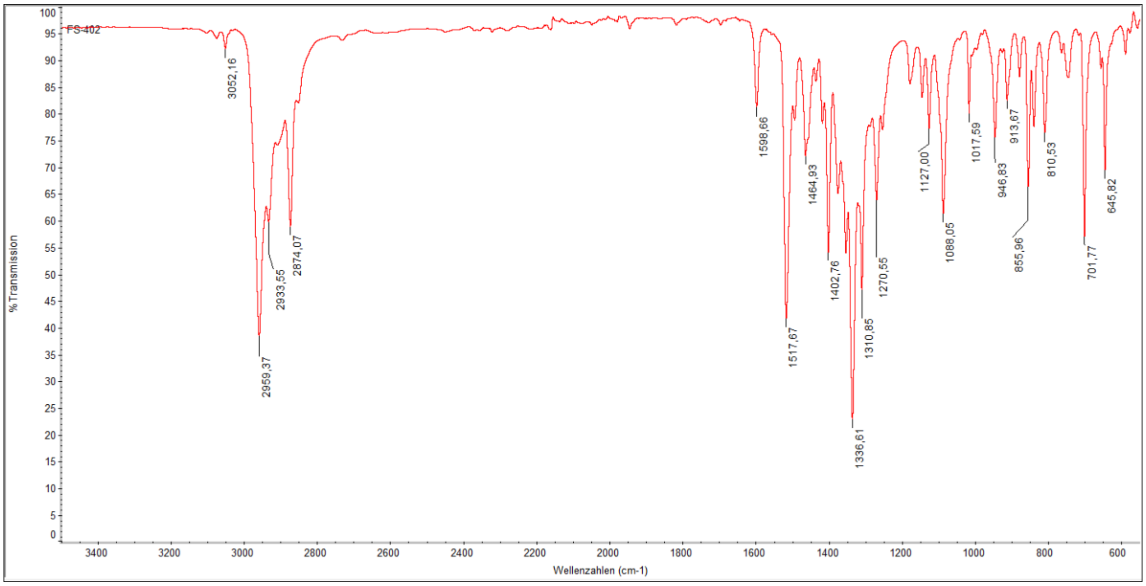

Figure S134: ATR FT-IR spectrum of compound **16b**.

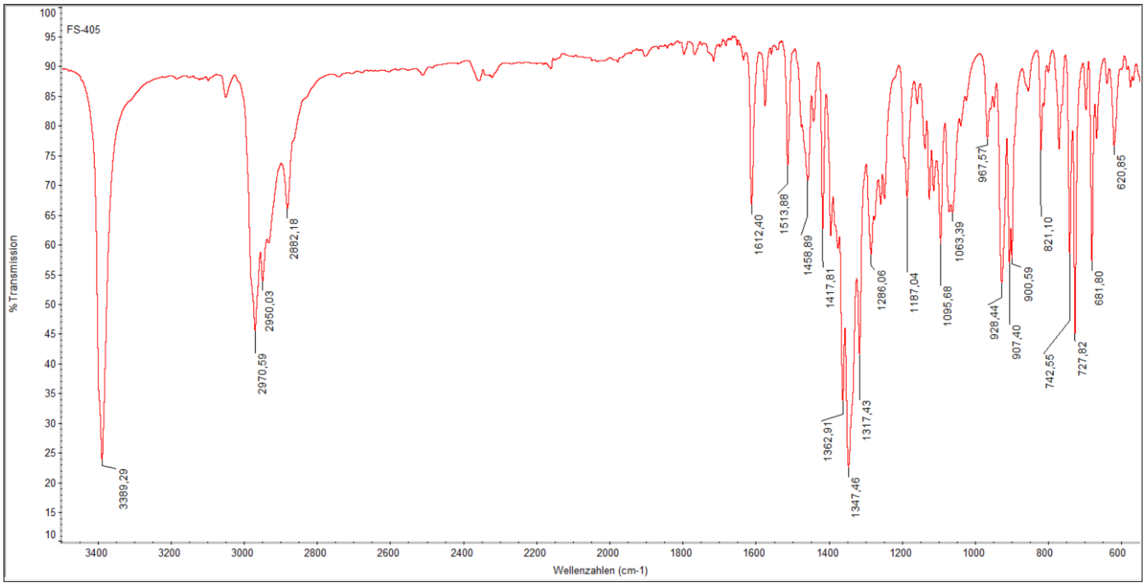

Figure S135: ATR FT-IR spectrum of compound **17a**.

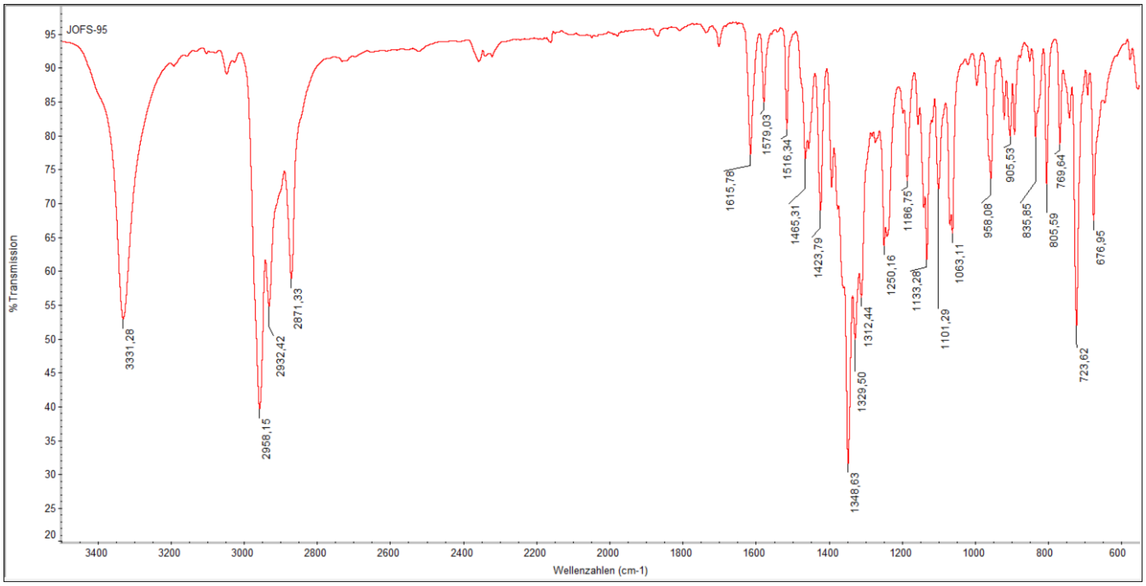

Figure S136: ATR FT-IR spectrum of compound **17b**.

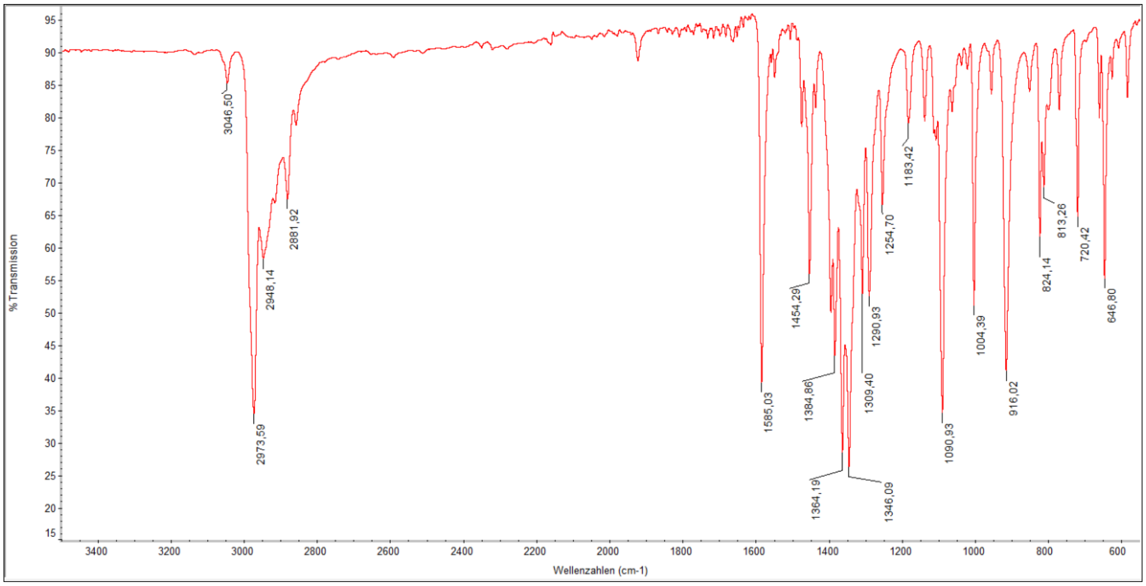

Figure S137: ATR FT-IR spectrum of compound **18a**.

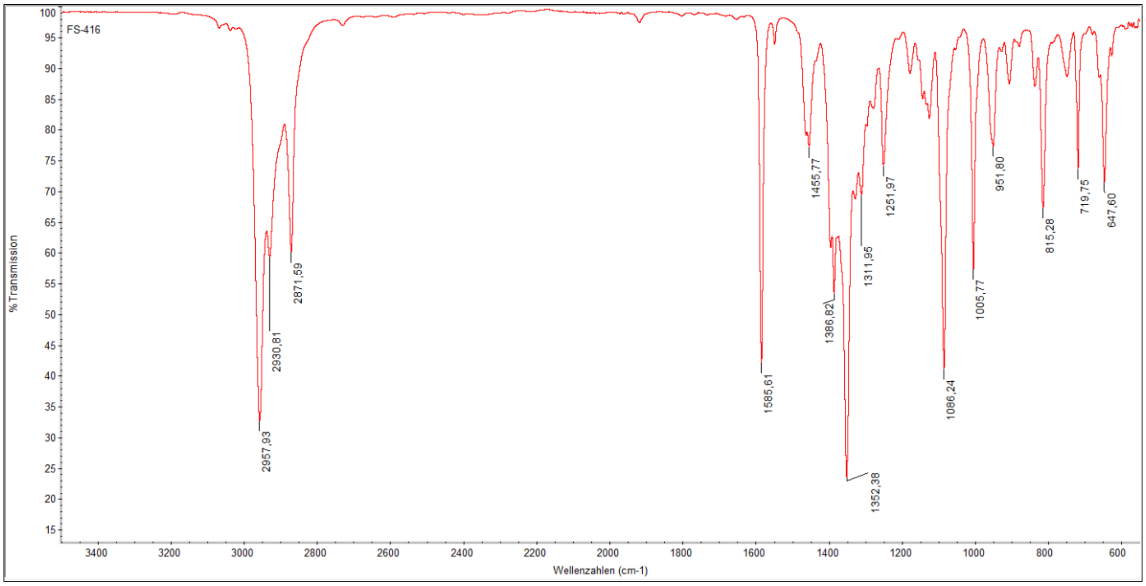

Figure S138: ATR FT-IR spectrum of compound **18b**.

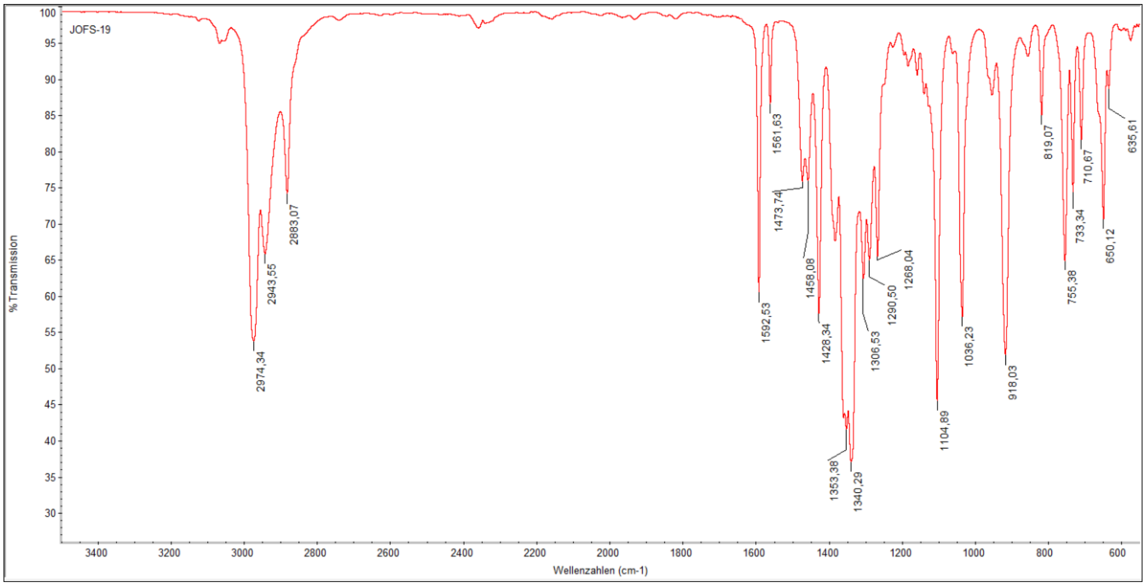

Figure S139: ATR FT-IR spectrum of compound **19a**.

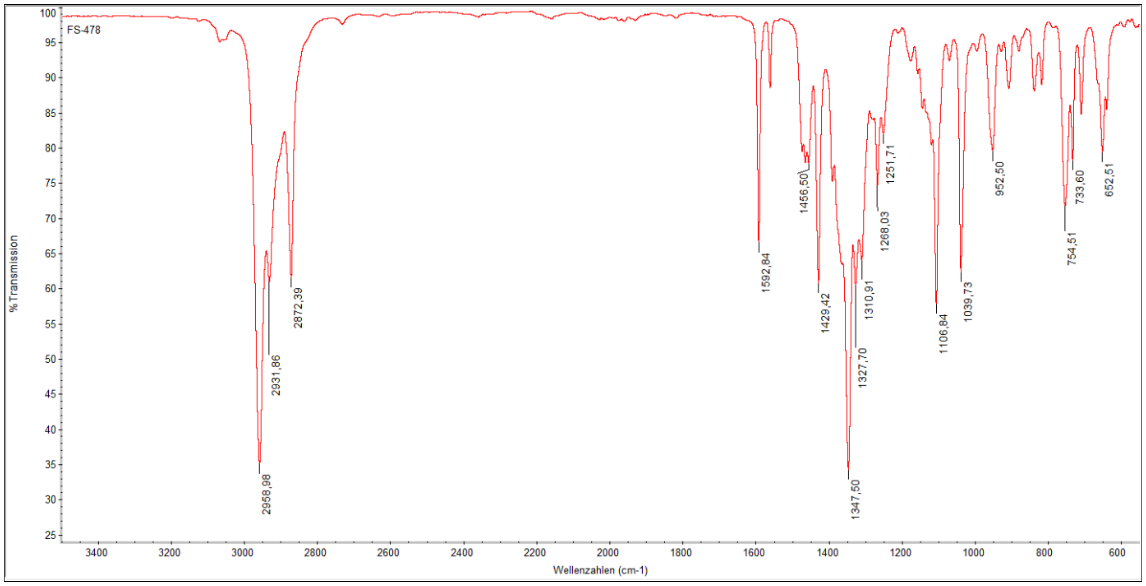

Figure S140: ATR FT-IR spectrum of compound **19b**.

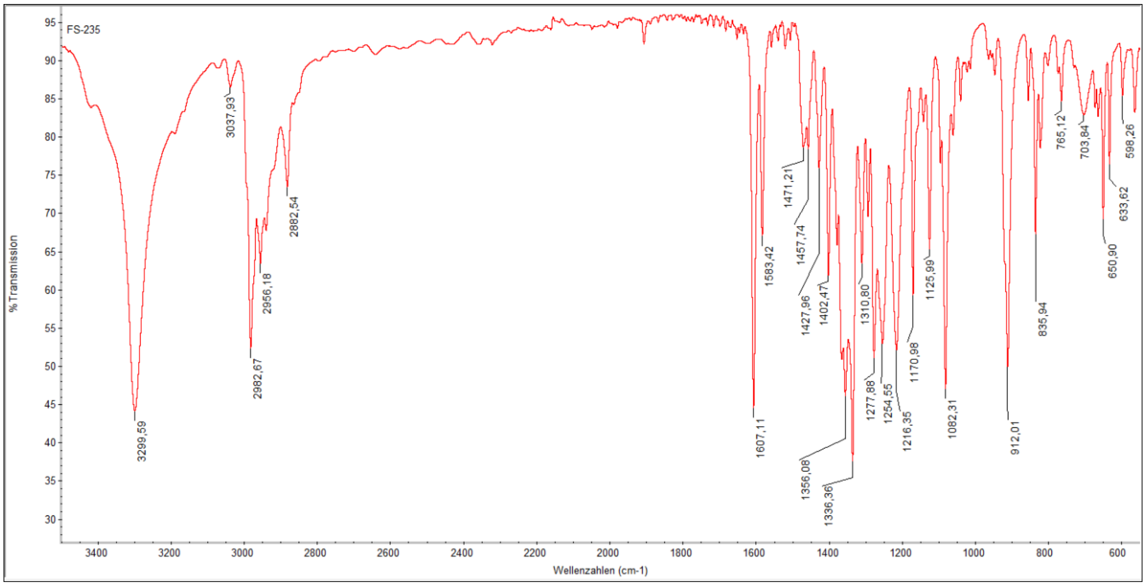

Figure S141: ATR FT-IR spectrum of compound **20a**.

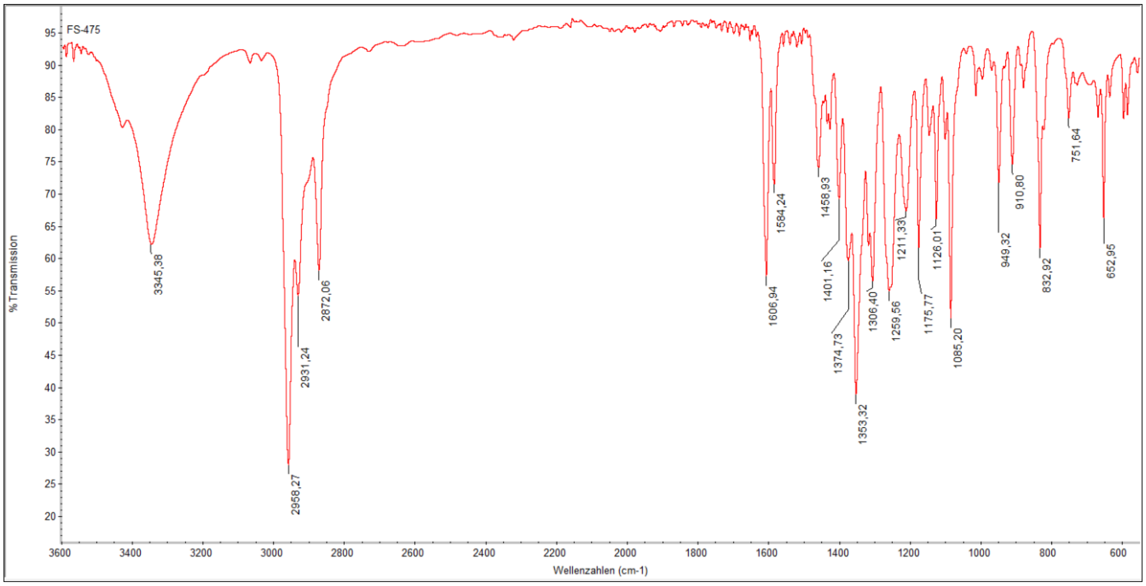

Figure S142: ATR FT-IR spectrum of compound **20b**.

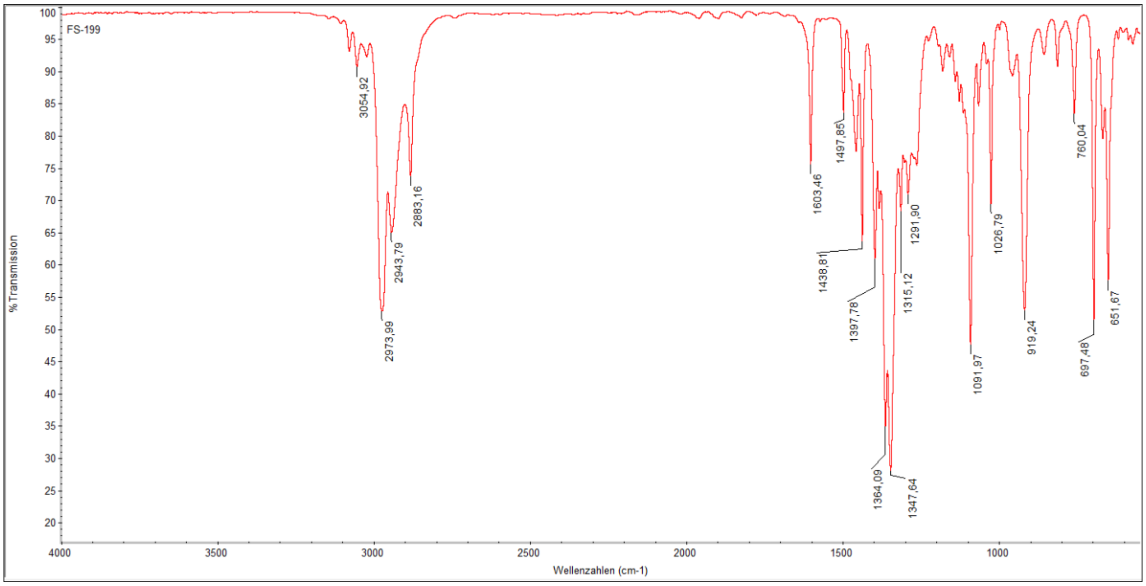

Figure S143: ATR FT-IR spectrum of compound **21a**.

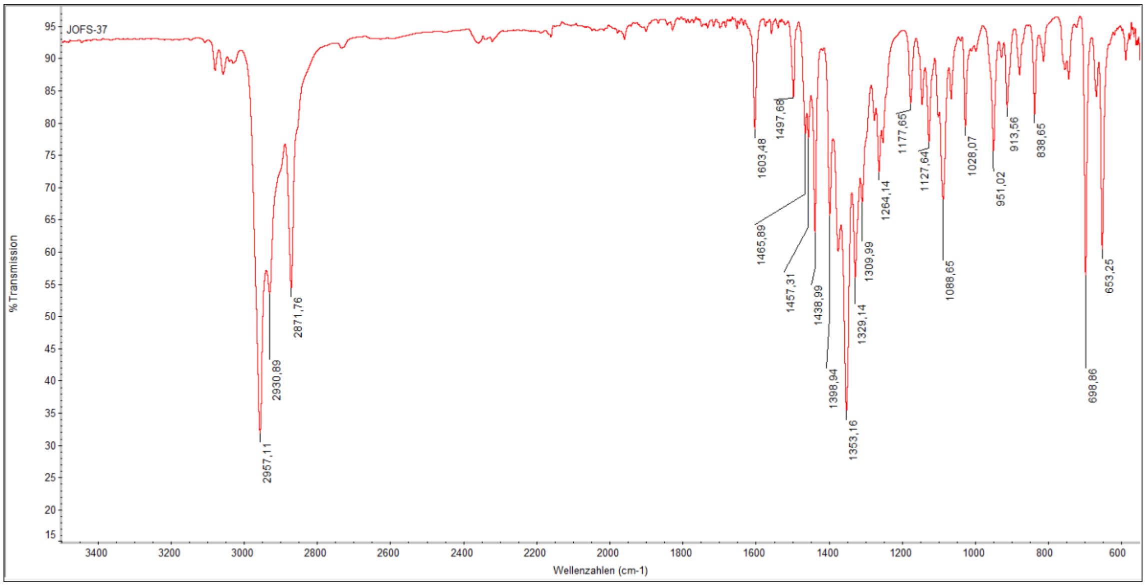

Figure S144: ATR FT-IR spectrum of compound **21b**.

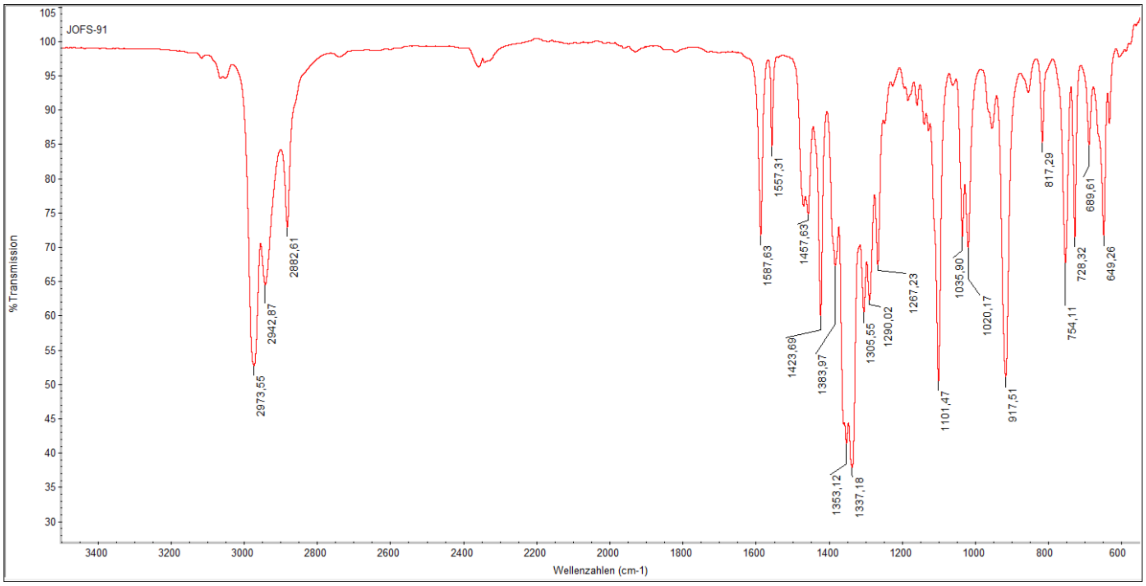

Figure S145: ATR FT-IR spectrum of compound **22a**.

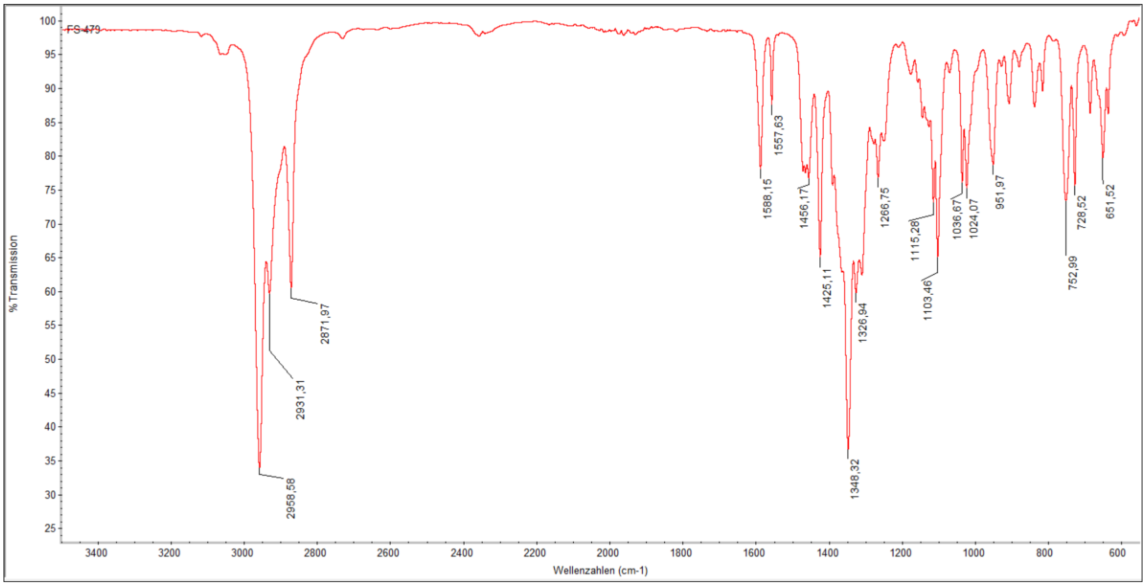

Figure S146: ATR FT-IR spectrum of compound **22b**

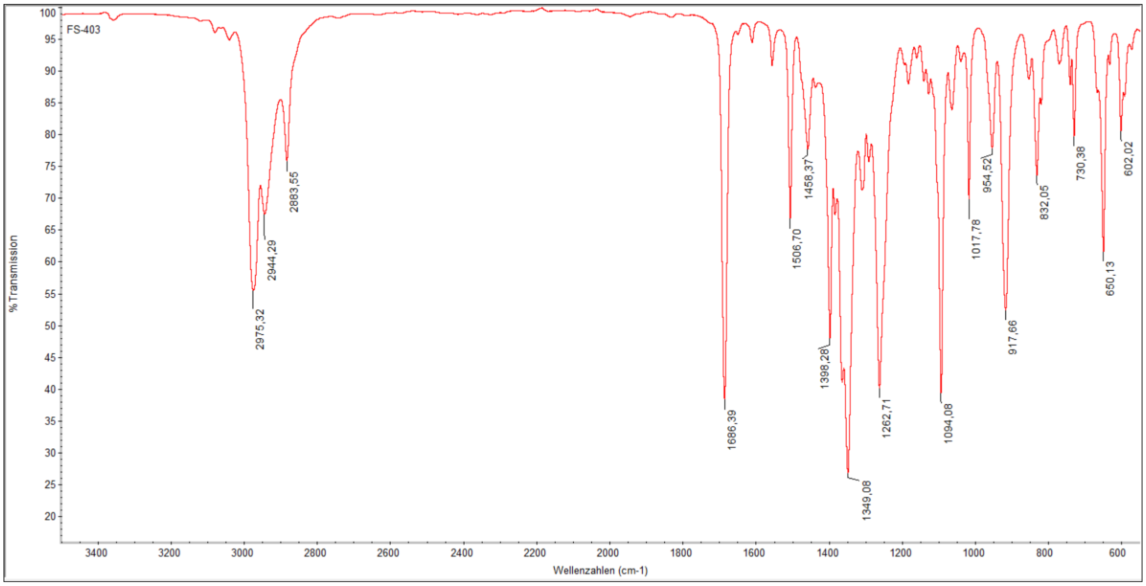

Figure S147: ATR FT-IR spectrum of compound **23a**.

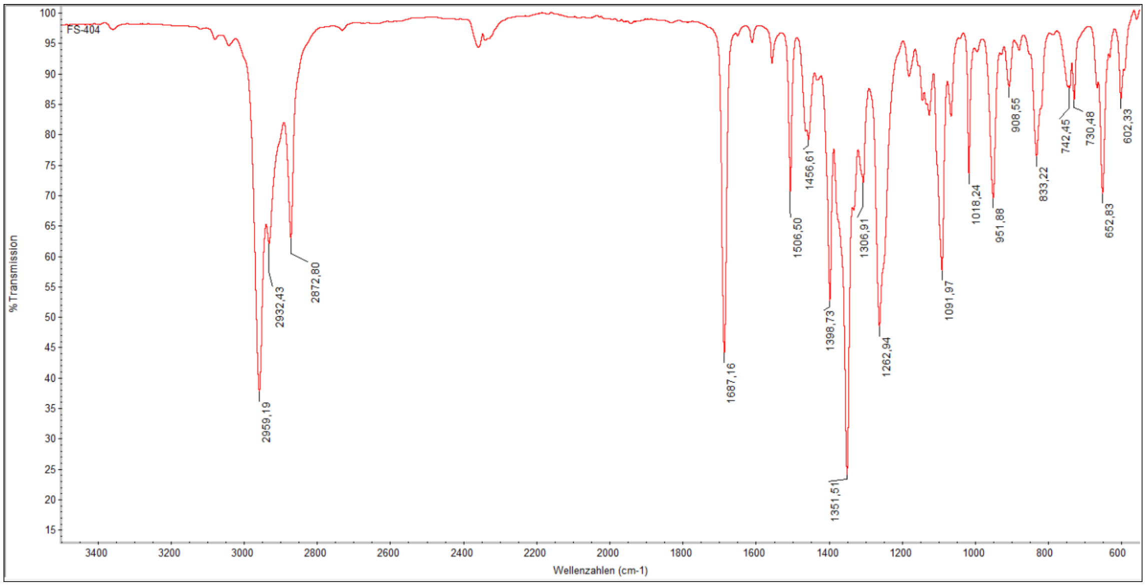

Figure S148: ATR FT-IR spectrum of compound **23b**.

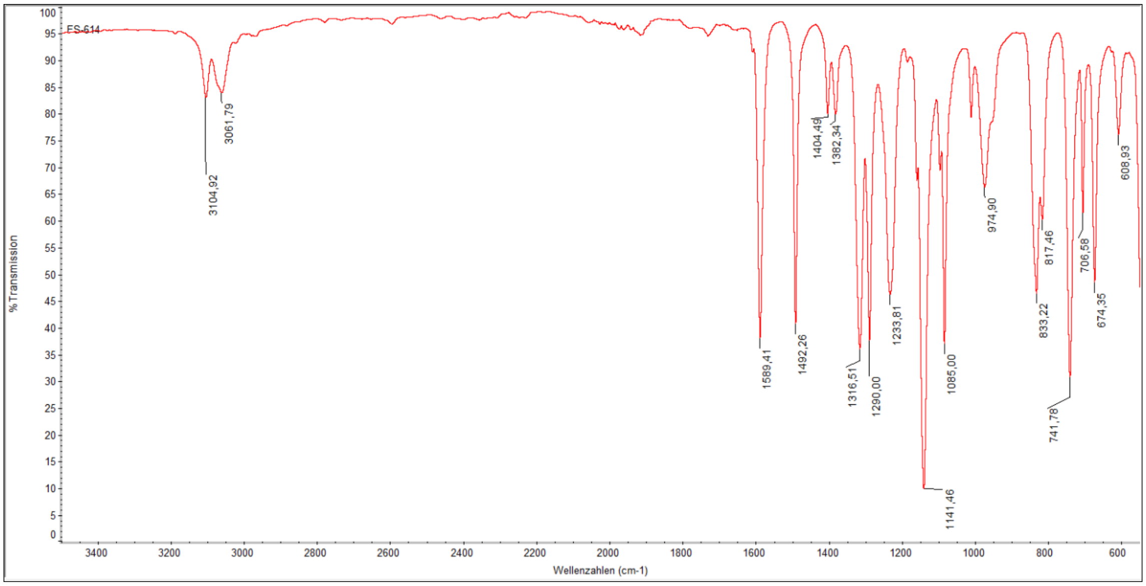

Figure S149: ATR FT-IR spectrum of compound **33**.

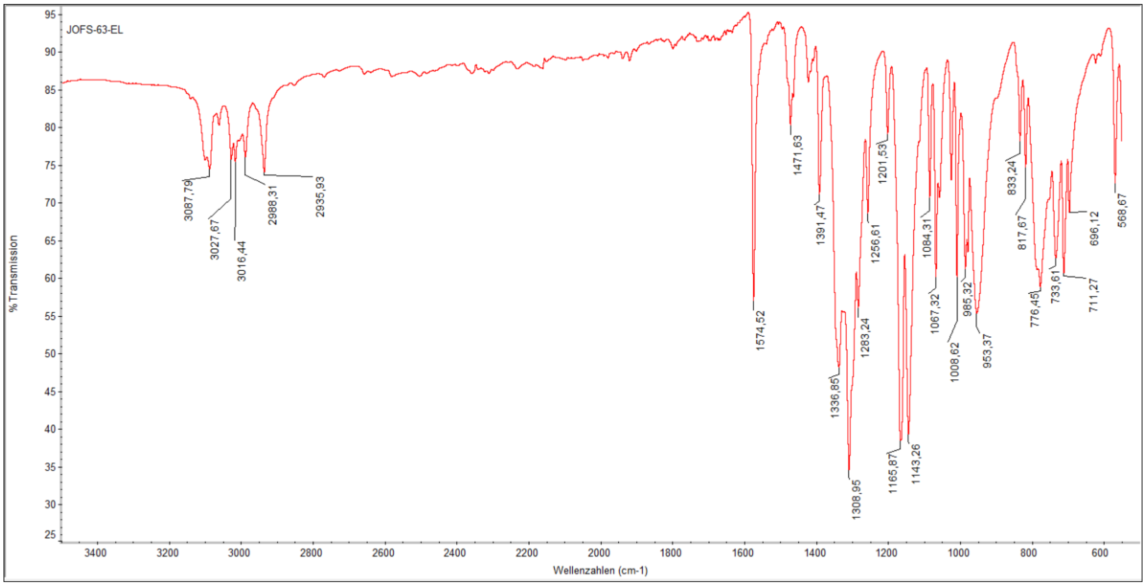

Figure S150: ATR FT-IR spectrum of compound **34**.

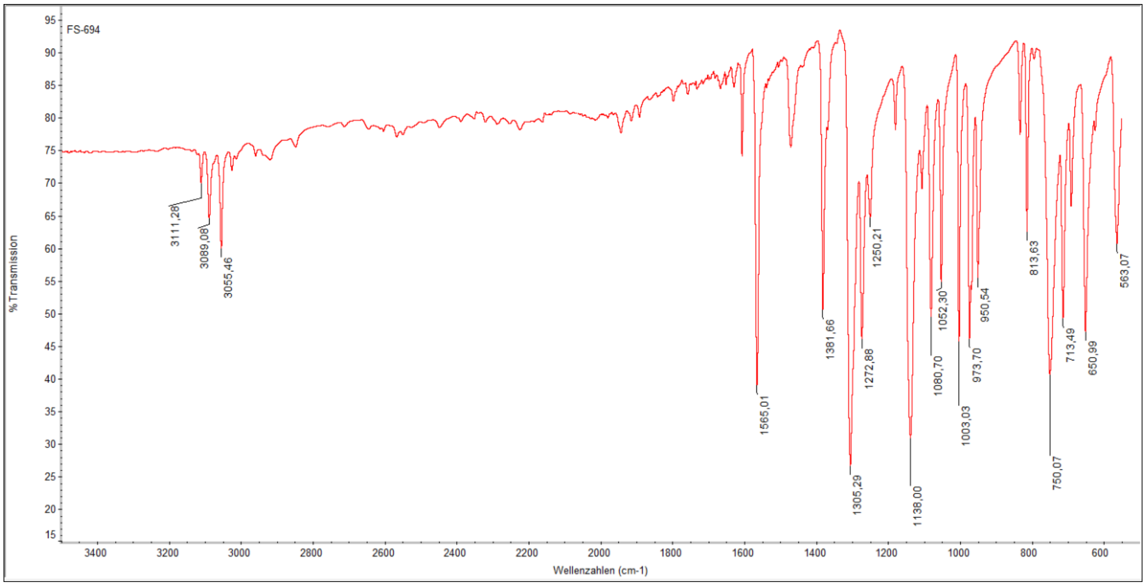

Figure S151: ATR FT-IR spectrum of compound **35**.

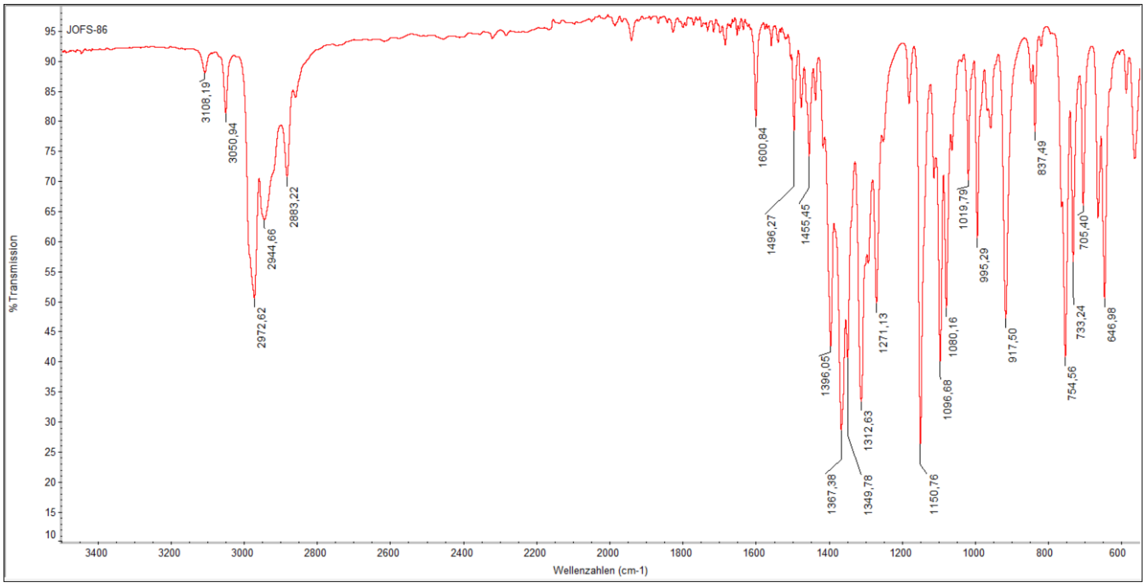

Figure S152: ATR FT-IR spectrum of compound **36**.

HR-MS of Novel Compounds

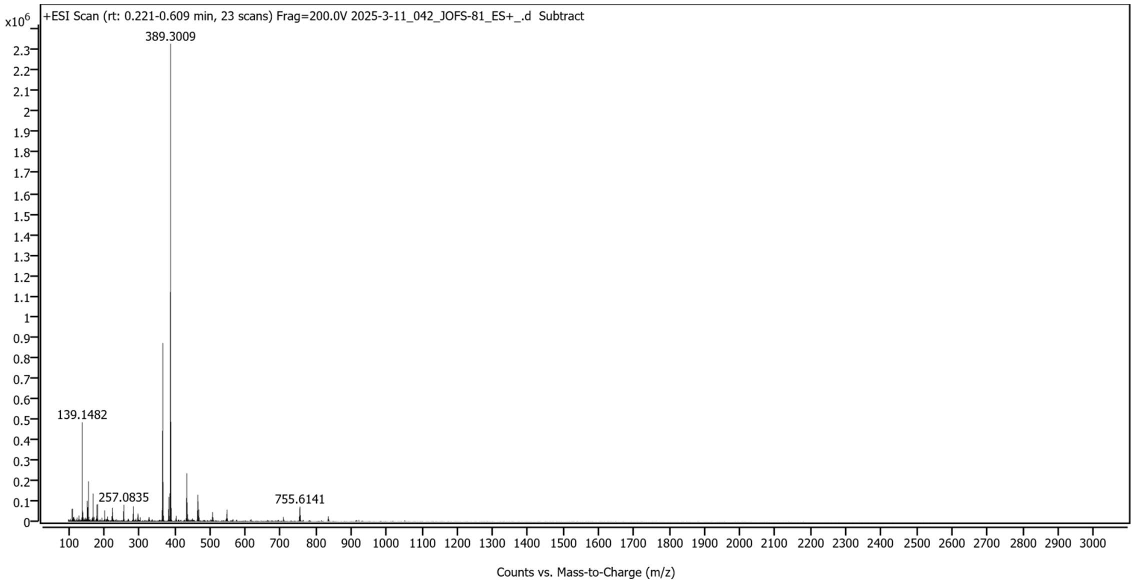

Figure S153: HR-MS spectrum of compound **S1**.

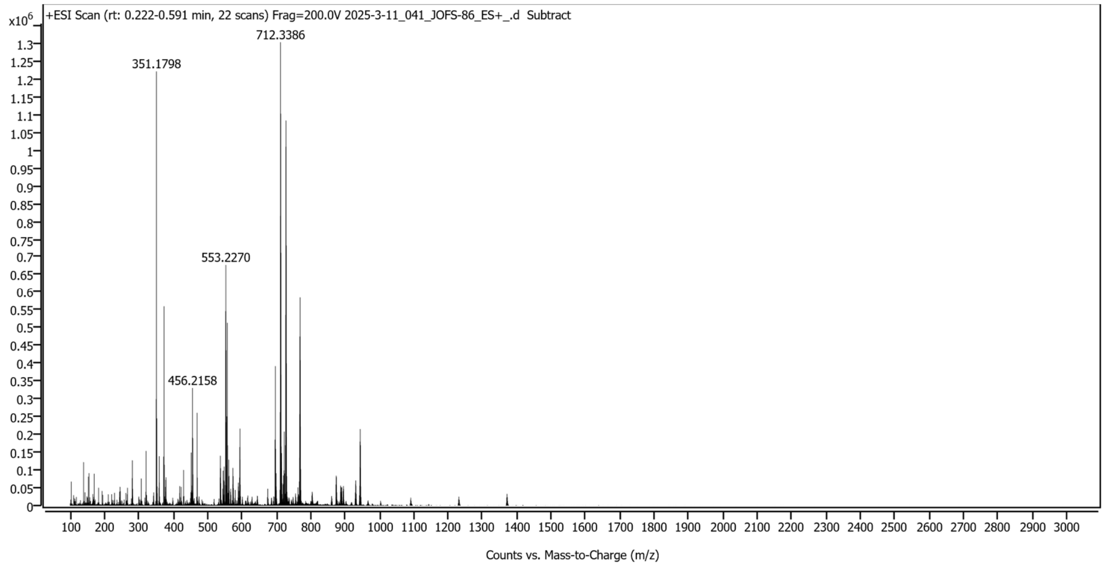

Figure S154: HR-MS spectrum of compound **36**.

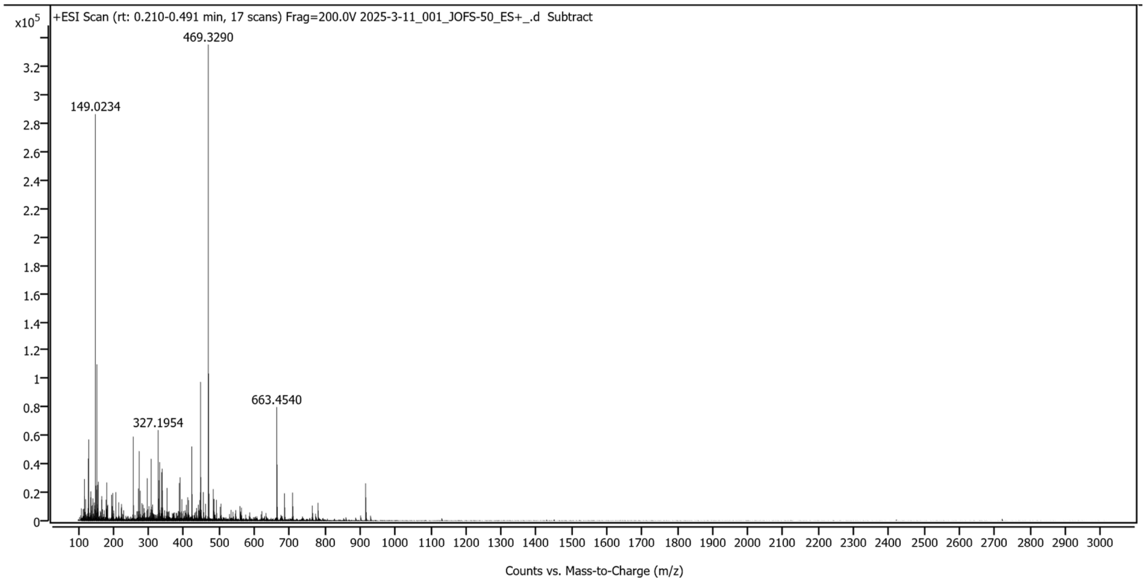

Spectrum Peaks

m/z	Z	Abund	Abund %	m/z (Calc)	Diff (ppm)	Ion Species	Formula	Ion Type
118.1227		29343	8.74					
128.9981		43747	13.03					
129.9950		57204	17.04					
149.0234		286587	85.37					
153.1387		110053	32.78					
157.1587		27470	8.18					
181.0823		26952	8.03					
256.2636		59215	17.64					
273.1673		48997	14.60					
296.2374		29898	8.91					
307.1880		43573	12.98					
327.1954		63824	19.01					
329.1936		28507	8.49					
331.2092		41307	12.31					
337.2337		34139	10.17					
338.3418		36677	10.93					
387.2363		26576	7.92					
389.2510		30558	9.10					
422.3782		52237	15.56					
447.3469	1	97637	29.09					
448.3502	1	30689	9.14					
469.3290	1	335683	100.00					
470.3323	1	103582	30.86					
663.4540	1	79989	23.83					
664.4573	1	39529	11.78					

+ Scan (rt: 0.491-0.597 min) Sub Peak 3 from + TIC Scan

Figure S155: HR-MS spectrum of compound **5a**.

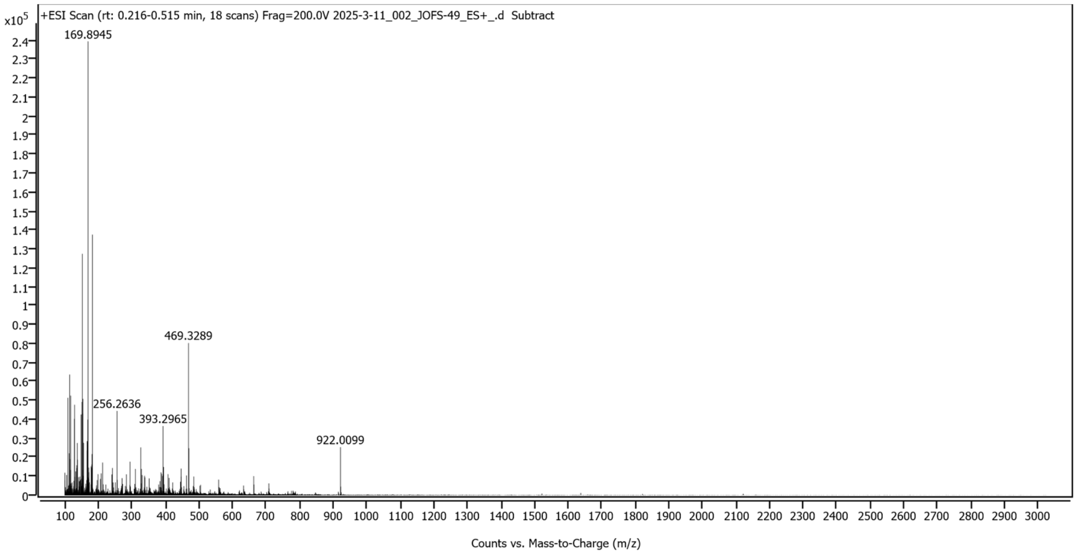

Figure S156: HR-MS spectrum of compound **5b**.

+ Scan (rt: 0.224-0.611 min) Sub Peak 1 from + TIC Scan

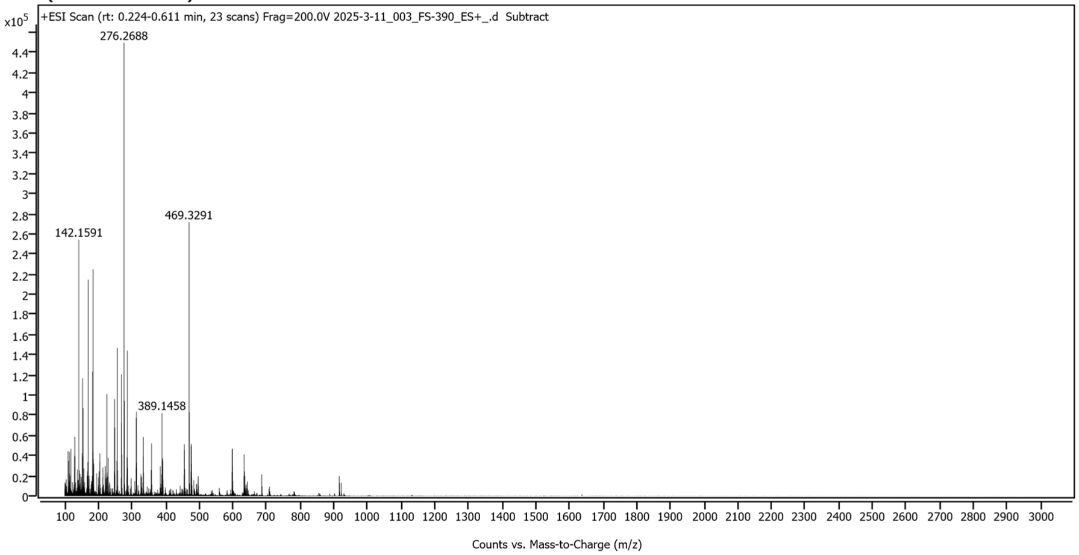

Spectrum Peaks

m/z	Z	Abund	Abund %	m/z (Calc)	Diff (ppm)	Ion Species	Formula	Ion Type
130.0459		58577	13.03					
142.1591		254234	56.55					
153.1387		116633	25.94					
154.9074		87067	19.37					
169.8945		214399	47.69					
182.9023		123283	27.42					
184.2061		224652	49.97					
225.1036		101091	22.48					
248.1394		95857	21.32					
248.6387	2	67619	15.04					
256.1505		146793	32.65					
268.6528	2	72227	16.06					
269.1514		120578	26.82					
276.2688	1	449611	100.00					
277.2721	1	94214	20.95					
286.1975		144042	32.04					
312.6590	2	77240	17.18					
313.1580		83644	18.60					
333.6710		58233	12.95					
358.1274		51978	11.56					
389.1458		82030	18.24					
455.7536		51142	11.37					
469.3291	1	271426	60.37					
470.3324	1	82834	18.42					
476.2671		51645	11.49					

+ Scan (rt: 0.506-0.558 min) Sub Peak 2 from + TIC Scan

Figure S157: HR-MS spectrum of compound **6a**.

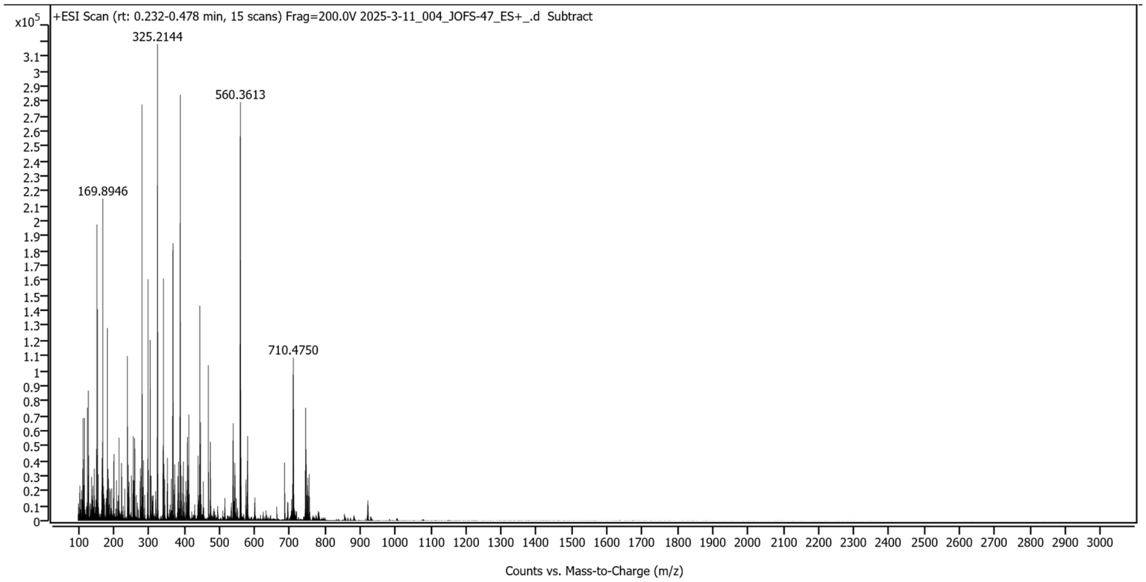

Spectrum Peaks

m/z	Z	Abund	Abund %	m/z (Calc)	Diff (ppm)	Ion Species	Formula	Ion Type
153.1388		197597	62.16					
154.9075		140801	44.29					
169.8946		214960	67.62					
182.9024		128241	40.34					
239.6826	2	109594	34.48					
281.1663		277597	87.33					
298.1975		160757	50.57					
304.2021		120375	37.87					
324.7154		223830	70.41					
325.2144	2	317884	100.00					
325.7157	2	125590	39.51					
342.2602		161221	50.72					
368.7217		180510	56.78					
369.2210		185037	58.21					
389.2350		198409	62.42					
389.7339	2	284028	89.35					
390.2350	2	132526	41.69					
445.2085		143138	45.03					
469.3289		103529	32.57					
559.3630		117265	36.89					
559.8619	2	256260	80.61					
560.3613	2	279057	87.79					
560.8620	2	173956	54.72					
709.9754	2	97312	30.61					
710.4750	2	108512	34.14					

Figure S158: HR-MS spectrum of compound **6b**.

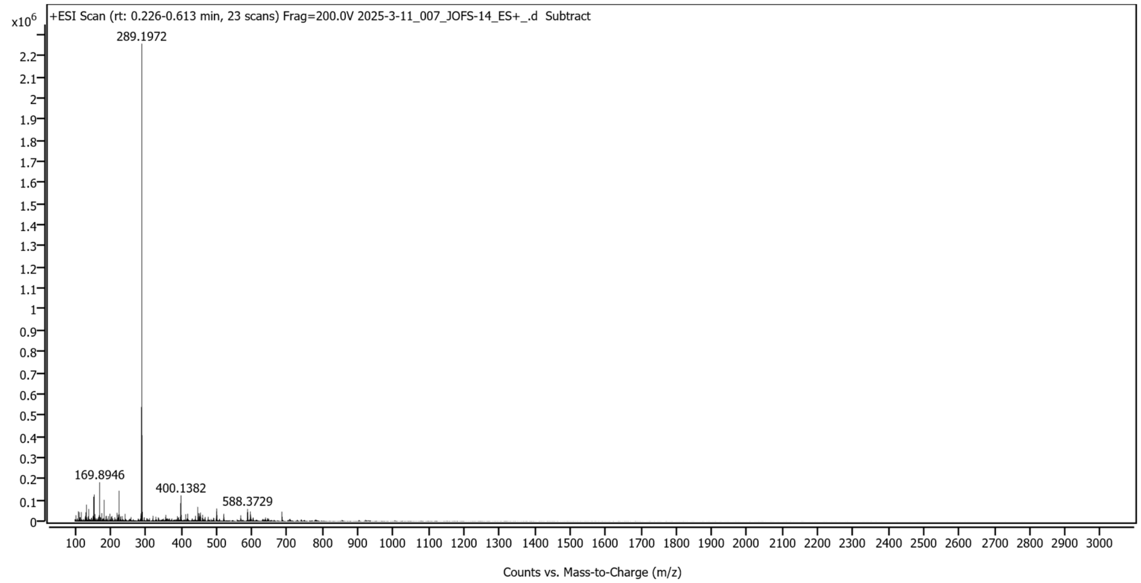

Figure S159: HR-MS spectrum of compound **8a**.

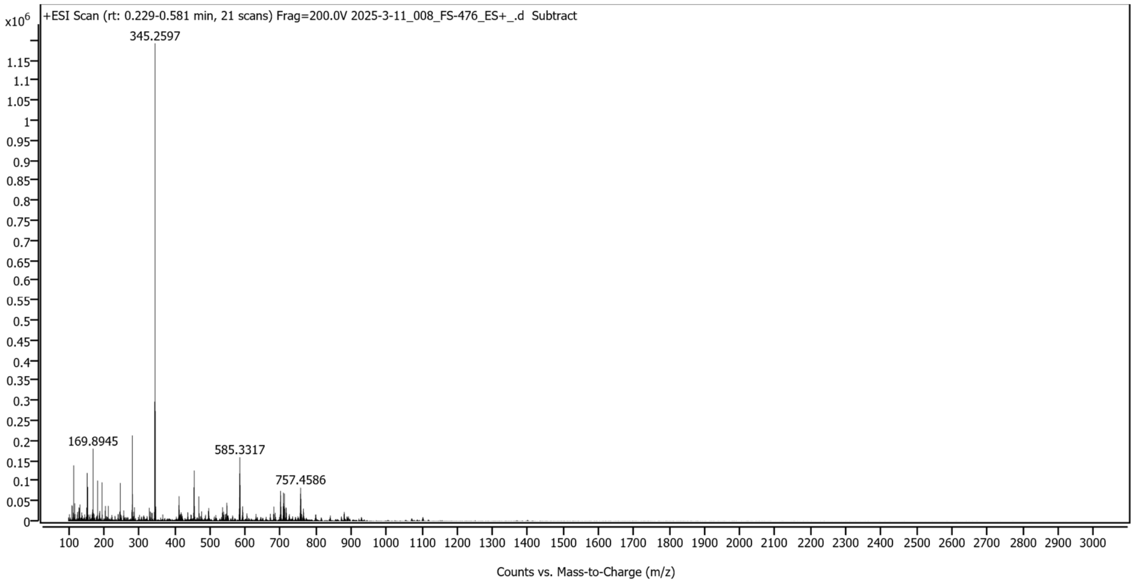

Figure S160: HR-MS spectrum of compound **8b**.

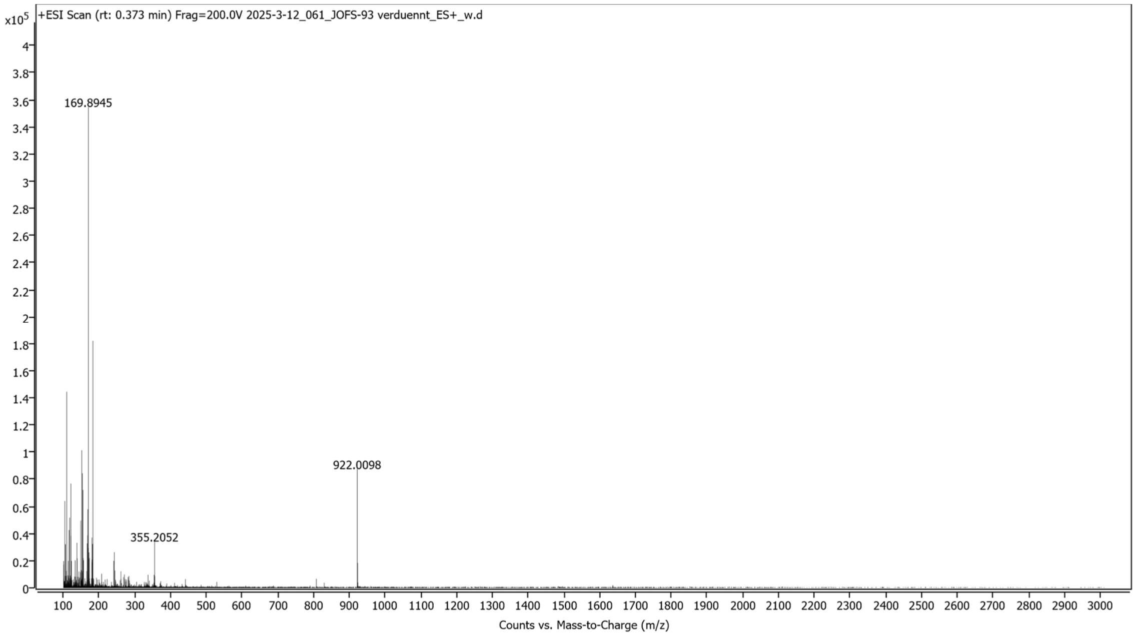

Figure S161: HR-MS spectrum of compound **9a**.

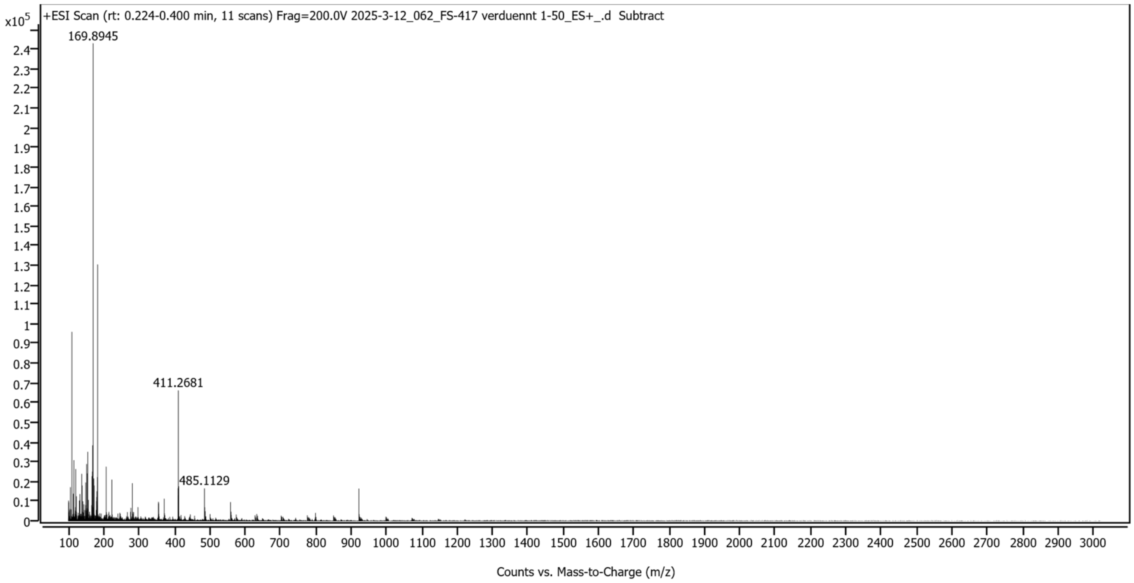

Figure S162: HR-MS spectrum of compound **9b**.

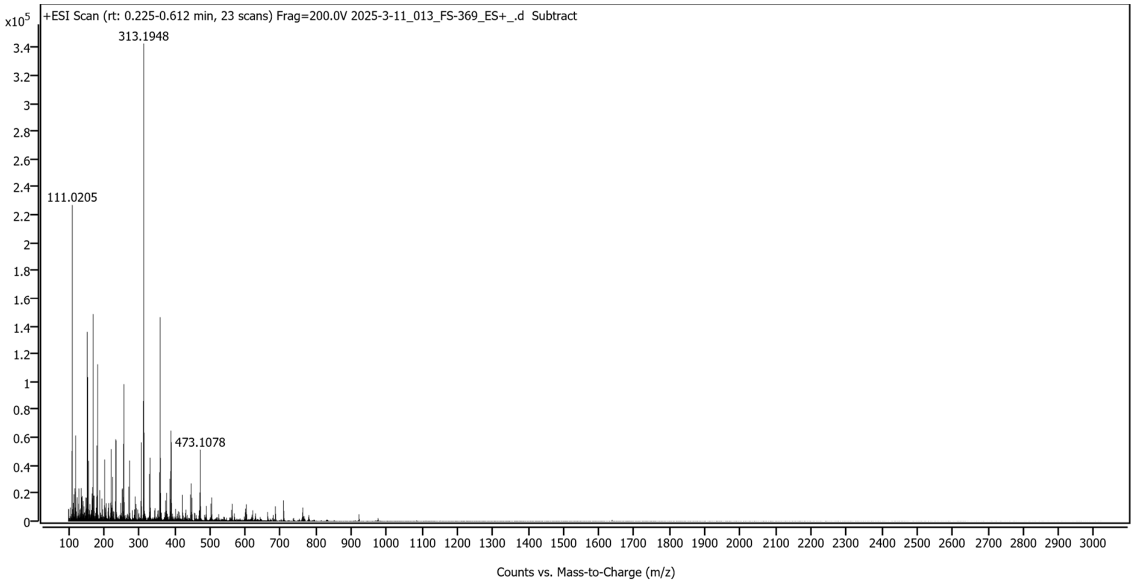

Figure S163: HR-MS spectrum of compound **11a**.

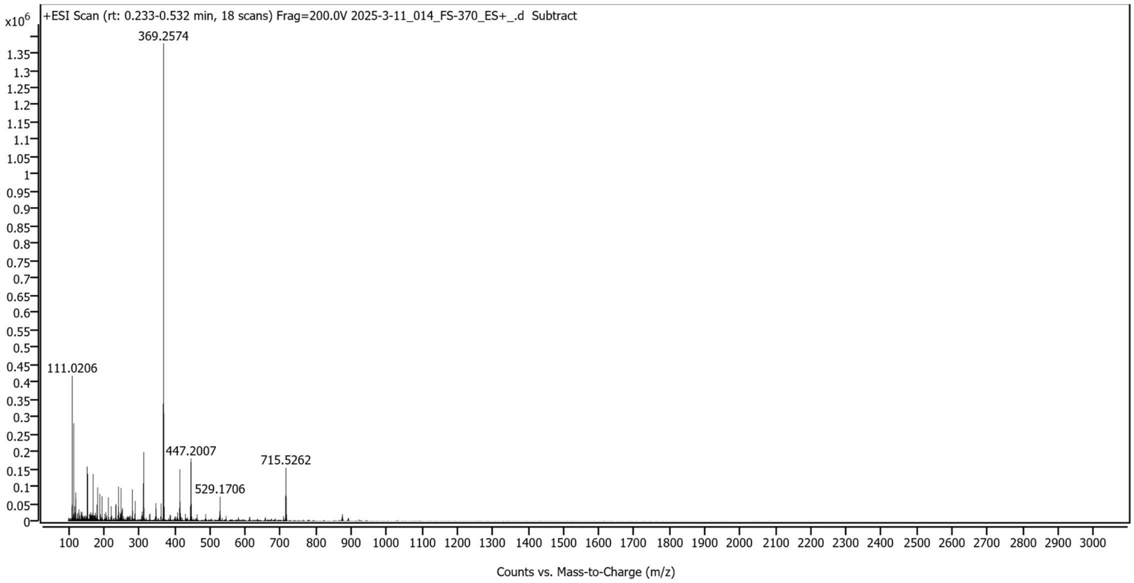

Figure S164: HR-MS spectrum of compound **11b**.

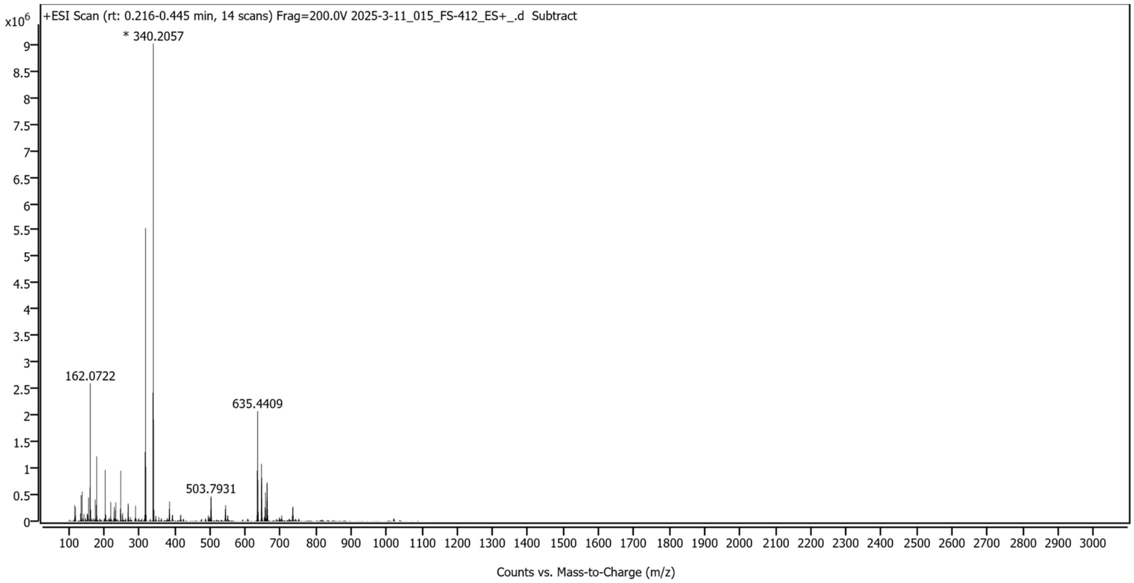

Figure S165: HR-MS spectrum of compound **12a**.

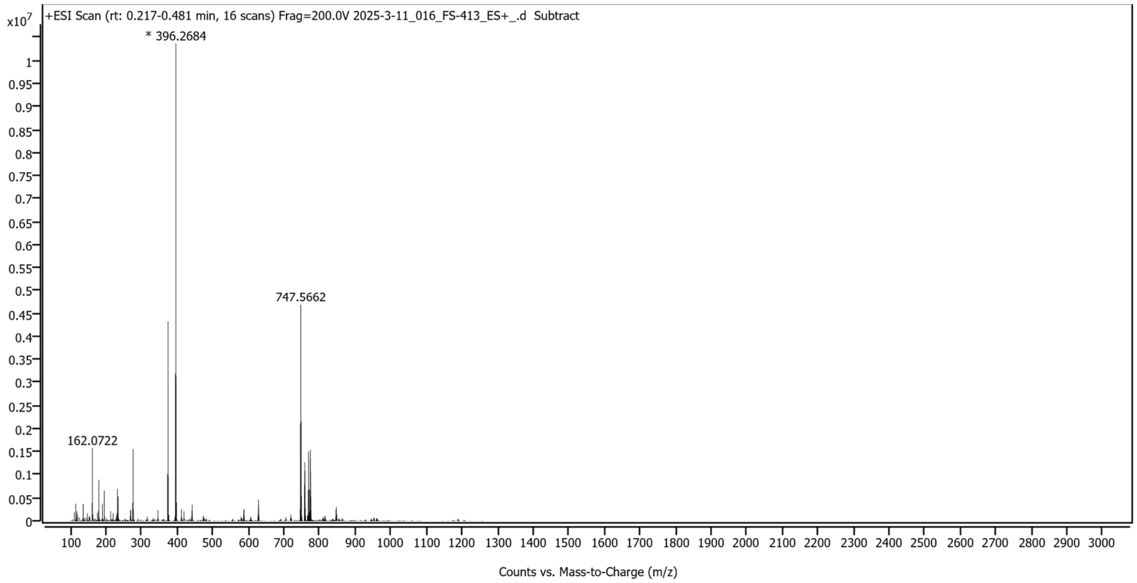

Figure S166: HR-MS spectrum of compound **12b**.

+ Scan (rt: 0.223-0.522 min) Sub **Peak 1 from + TIC Scan**

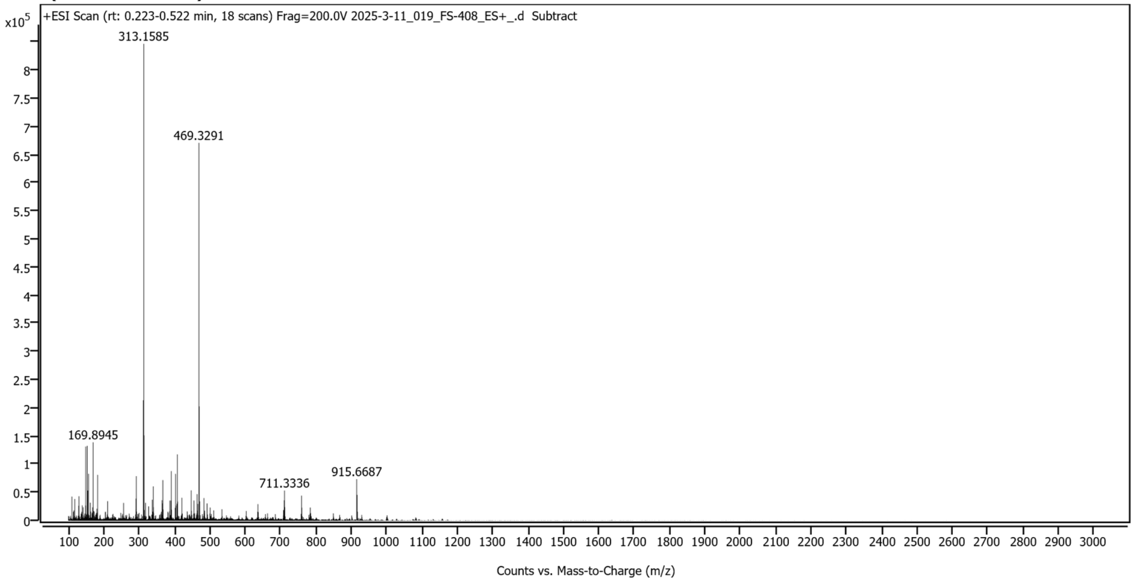

Spectrum Peaks

m/z	Z	Abund	Abund %	m/z (Calc)	Diff (ppm)	Ion Species	Formula	Ion Type
109.9436		42054	4.97					
129.9951		42647	5.04					
149.0234		130722	15.45					
153.1387		131945	15.59					
154.9075		52759	6.23					
157.1587		82497	9.75					
169.8945		138286	16.34					
182.9024		80575	9.52					
292.2080		78267	9.25					
312.1620		212861	25.15					
313.1585	1	846260	100.00					
314.1618	1	150714	17.81					
340.2058		60101	7.10					
367.2441		71286	8.42					
391.1012		87334	10.32					
403.2054		82315	9.73					
408.2708		116889	13.81					
447.3469		53136	6.28					
464.1541		46407	5.48					
469.3291	1	670296	79.21					
470.3324	1	201869	23.85					
711.3336		52786	6.24					
759.4985		43696	5.16					
915.6687	1	73077	8.64					
916.6722	1	45070	5.33					

+ Scan (rt: 0.522-0.592 min) Sub **Peak 2 from + TIC Scan**

Figure S167: HR-MS spectrum of compound **14a**.

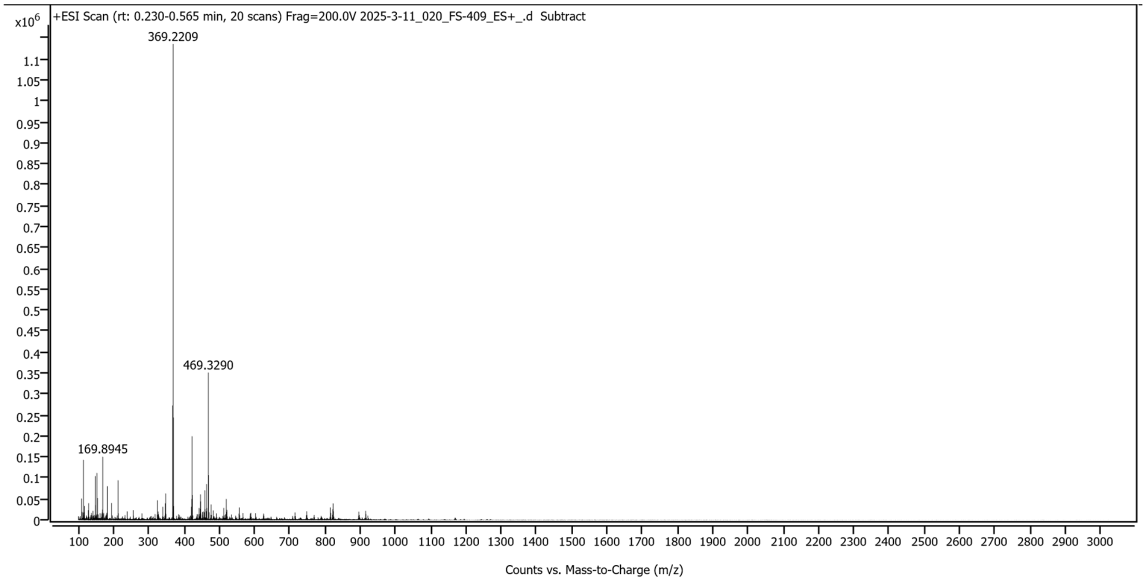

Spectrum Peaks

m/z	Z	Abund	Abund %	m/z (Calc)	Diff (ppm)	Ion Species	Formula	Ion Type
109.9436		50619	4.46					
115.1117		142613	12.56					
129.9949		39470	3.48					
149.0233		104124	9.17					
153.1386		111555	9.82					
154.9074		51803	4.56					
169.8945		150091	13.22					
182.9023		79852	7.03					
195.2107		39953	3.52					
213.2213		94063	8.28					
325.1970		46504	4.09					
348.2705		62636	5.52					
368.2245		272306	23.98					
369.2209	1	1135666	100.00					
370.2243	1	243805	21.47					
422.3102		49585	4.37					
423.3067	1	199001	17.52					
424.3103	1	59020	5.20					
447.1638		60552	5.33					
447.3468		43731	3.85					
459.2681		70208	6.18					
464.3334		85045	7.49					
469.3290	1	350734	30.88					
470.3323	1	106294	9.36					
520.2167		49570	4.36					

+ Scan (rt: 0.565-0.617 min) Sub **Peak 4 from + TIC Scan**

Figure S168: HR-MS spectrum of compound **14b**.

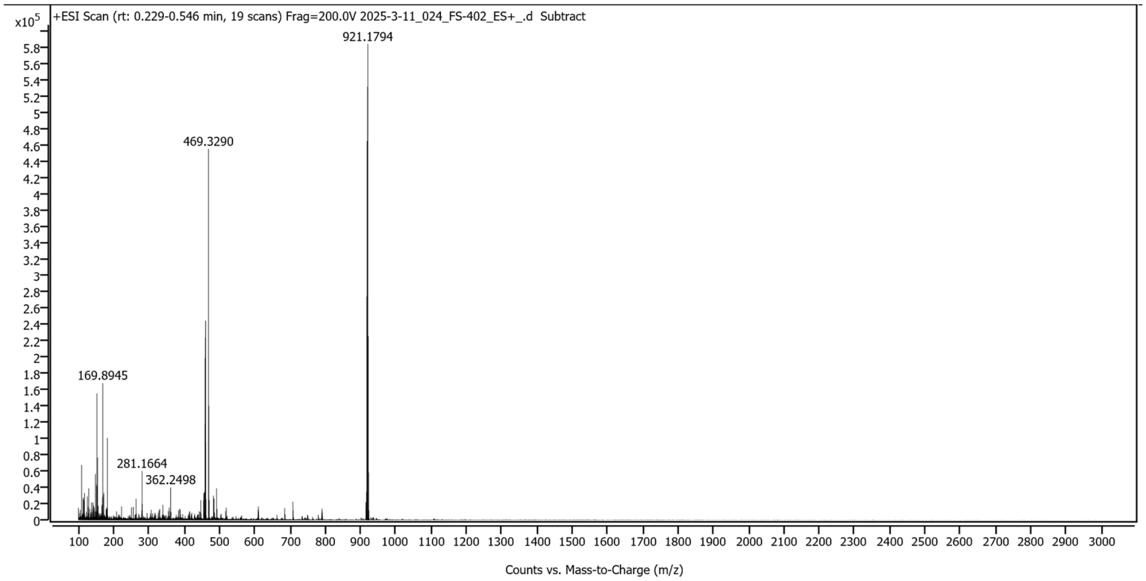

Spectrum Peaks

m/z	Z	Abund	Abund %	m/z (Calc)	Diff (ppm)	Ion Species	Formula	Ion Type
109.9436		67319	11.50					
129.9950		38499	6.58					
149.0234		56238	9.61					
151.8618		43168	7.38					
153.1387		155222	26.52					
153.8600		40698	6.95					
154.9074		76386	13.05					
169.8945		167780	28.67					
182.9024		100366	17.15					
281.1664		59906	10.24					
362.2498		39709	6.78					
459.5914		117148	20.02					
460.0917		198496	33.92					
460.5926		223554	38.20					
461.0930	2	244418	41.76					
461.5943	2	94522	16.15					
469.3290	1	456207	77.95					
470.3324	1	140093	23.94					
492.4048		38602	6.60					
918.1761		274242	46.86					
919.1768		466021	79.62					
920.1784		532680	91.01					
921.1794	1	585276	100.00					
922.1820	1	225467	38.52					
923.1848	1	58281	9.96					

+ Scan (rt: 0.493-0.546 min) Sub **Peak 3 from + TIC Scan**

Figure S169: HR-MS spectrum of compound **16b**.

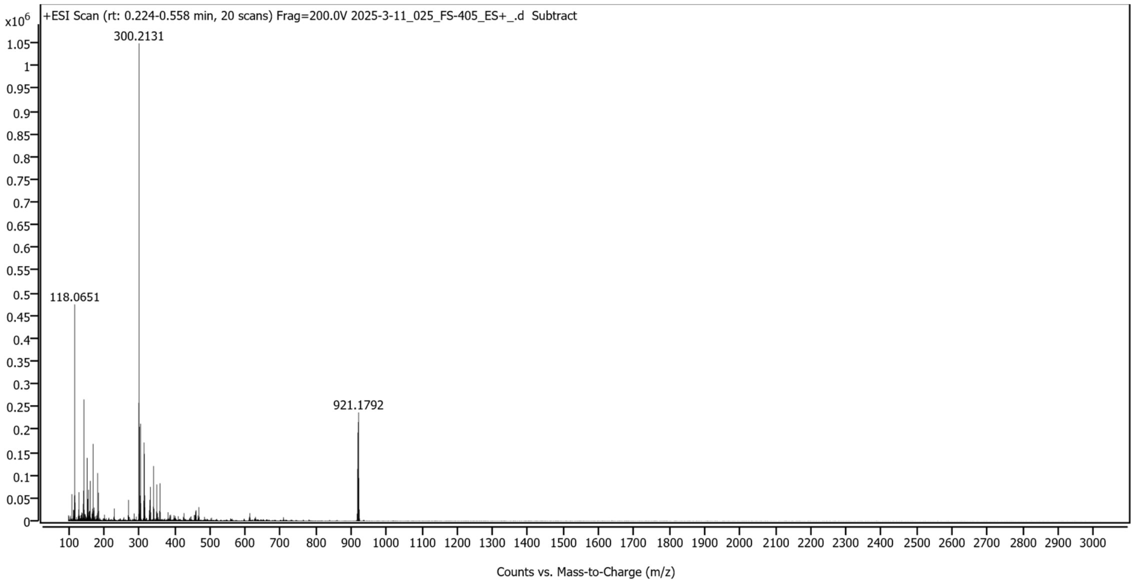

Figure S170: HR-MS spectrum of compound **17a**.

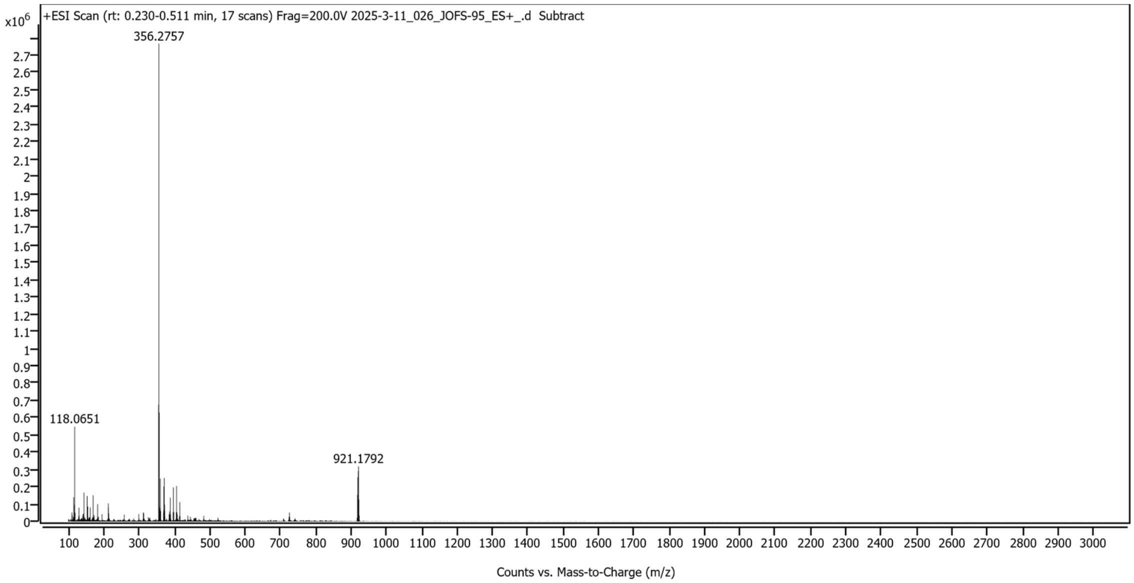

Figure S171: HR-MS spectrum of compound **17b**.

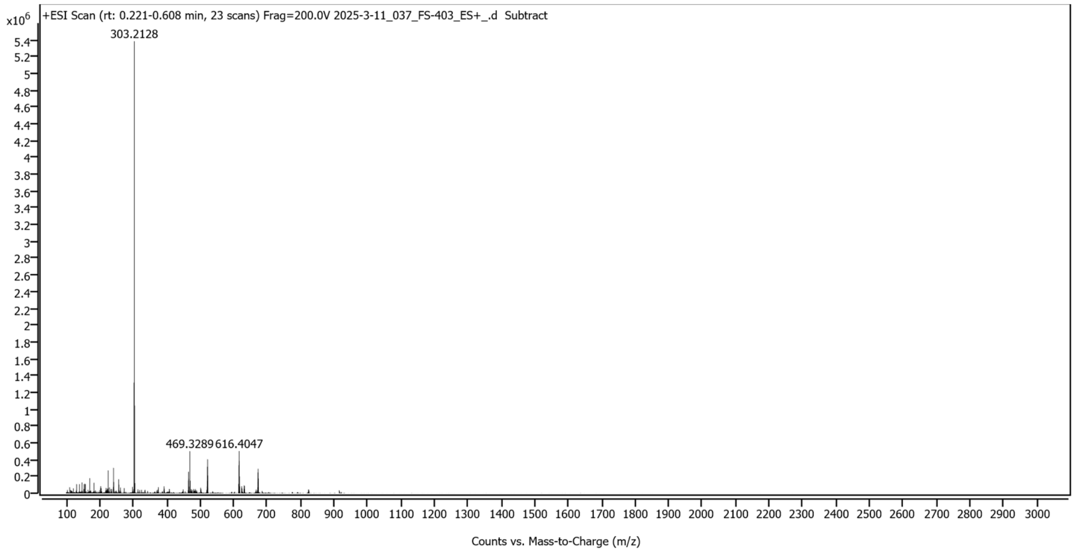

Figure S172: HR-MS spectrum of compound **23a**.

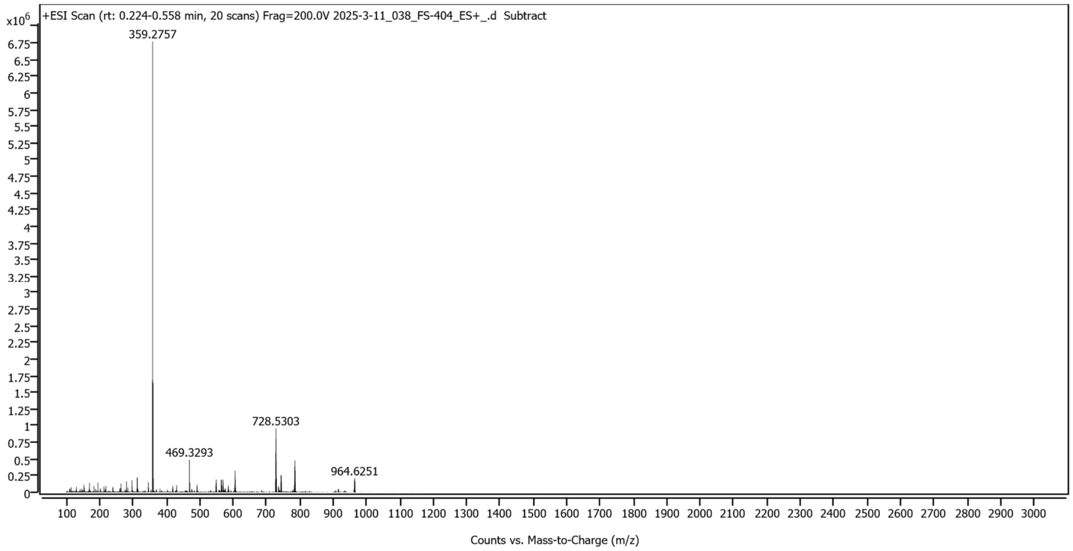

Figure S173: HR-MS spectrum of compound **23b**.

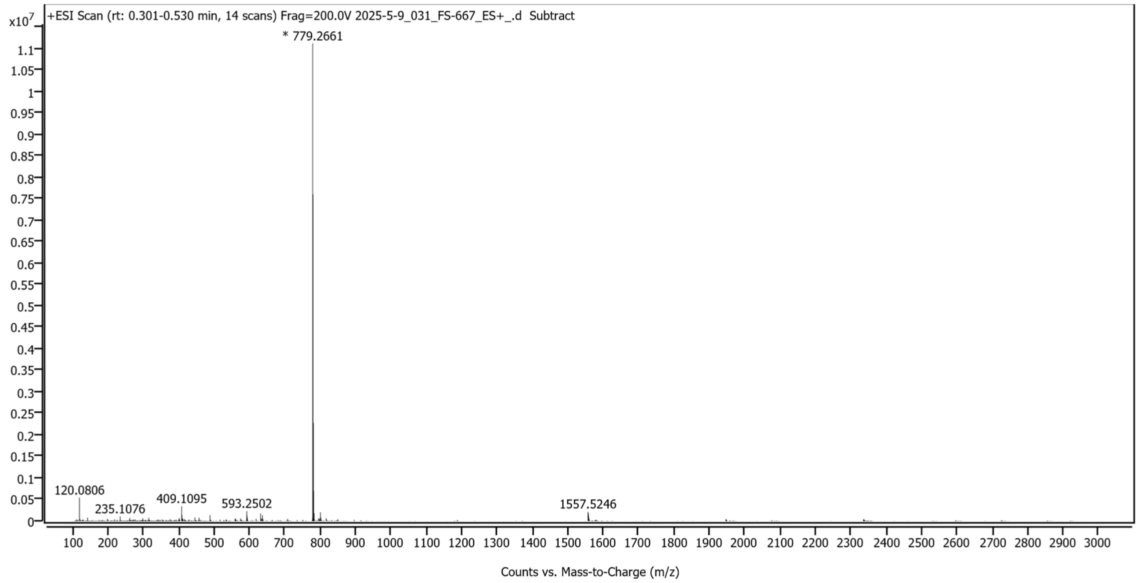

Figure S174: HR-MS spectrum of compound **80**.

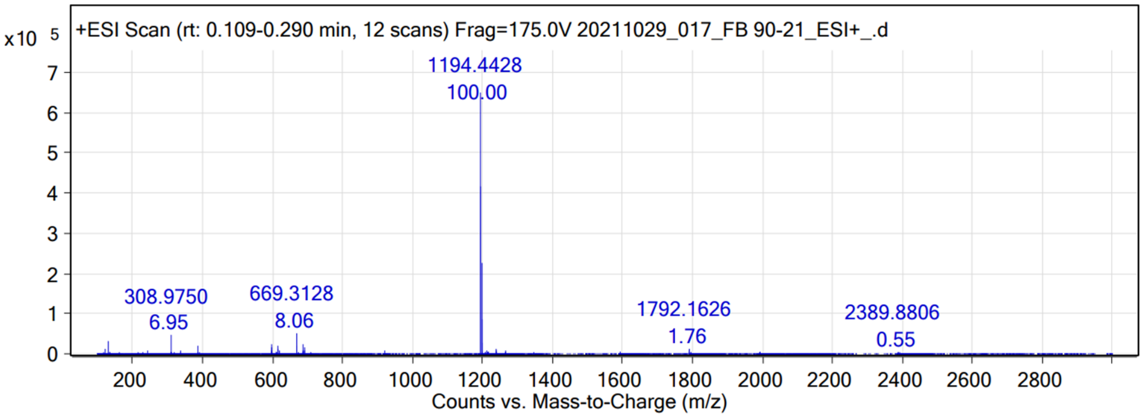

Figure S175: HR-MS spectrum of compound **81**.

HPLC runs of the labeling precursors and determination of their purity

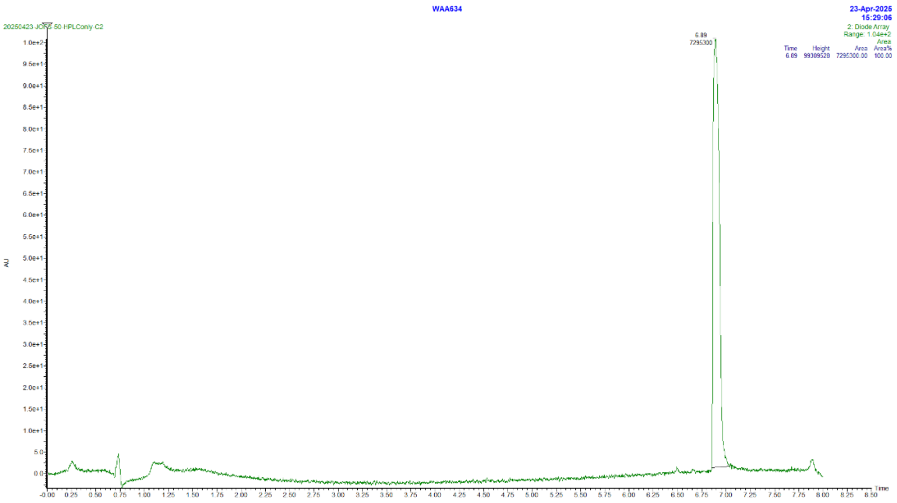

Figure S176: HPLC Chromatogram of compound **5a**.

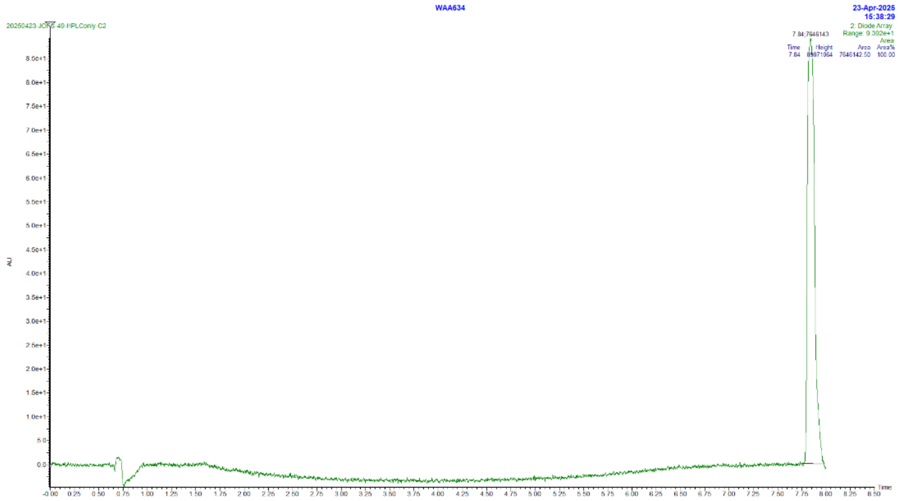

Figure S177. HPLC Chromatogram of compound **5b**.

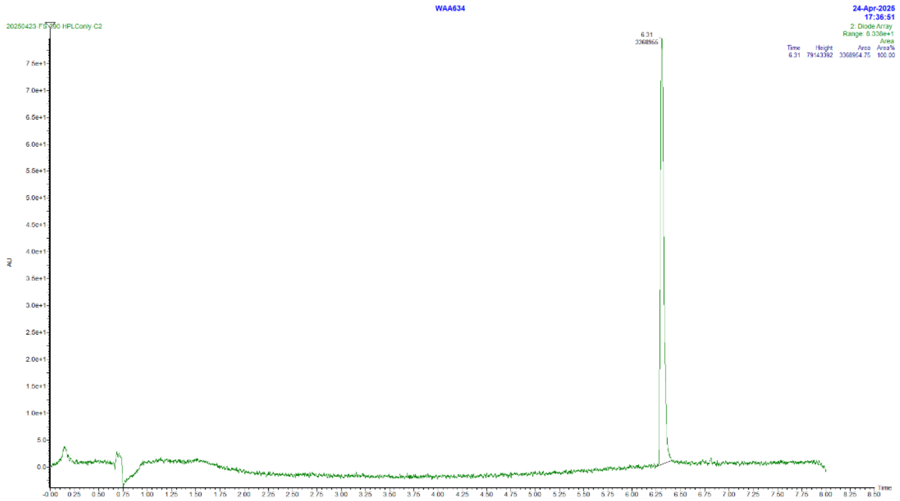

Figure S178: HPLC Chromatogram of compound **6a**.

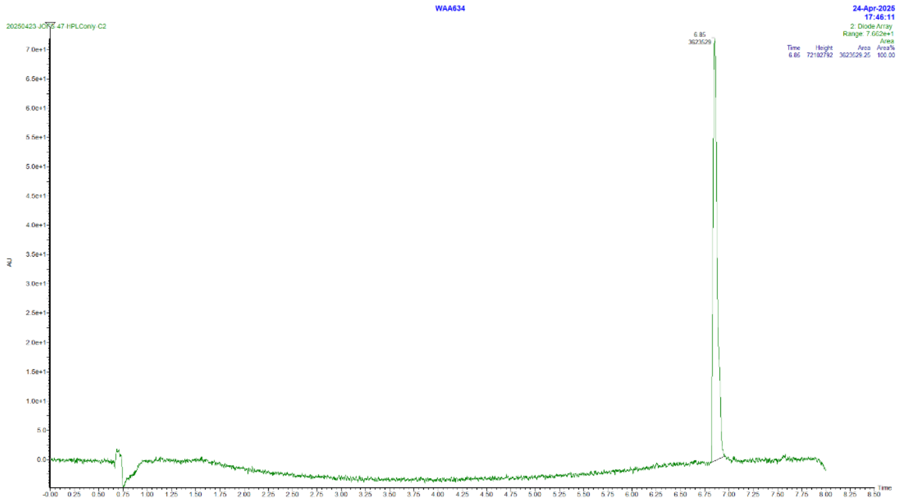

Figure S179: HPLC Chromatogram of compound **6b**.

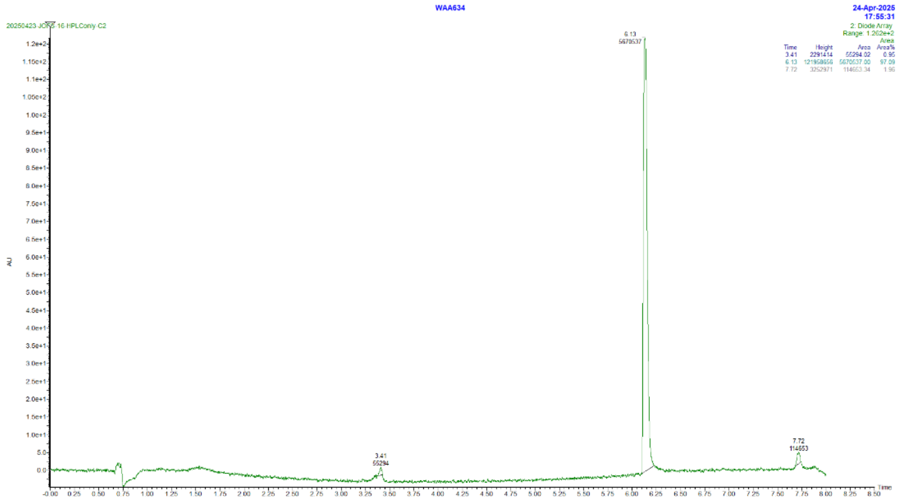

Figure S180: HPLC Chromatogram of compound **7a**.

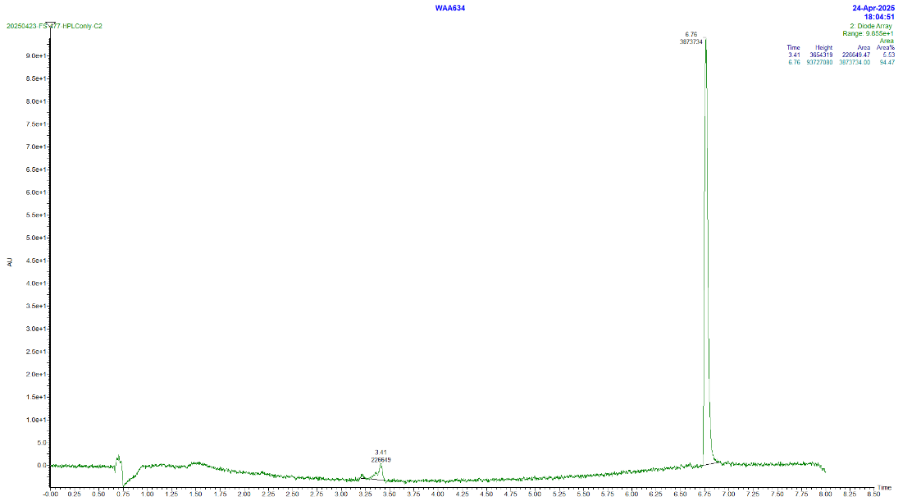

Figure S181: HPLC Chromatogram of compound **7b**.

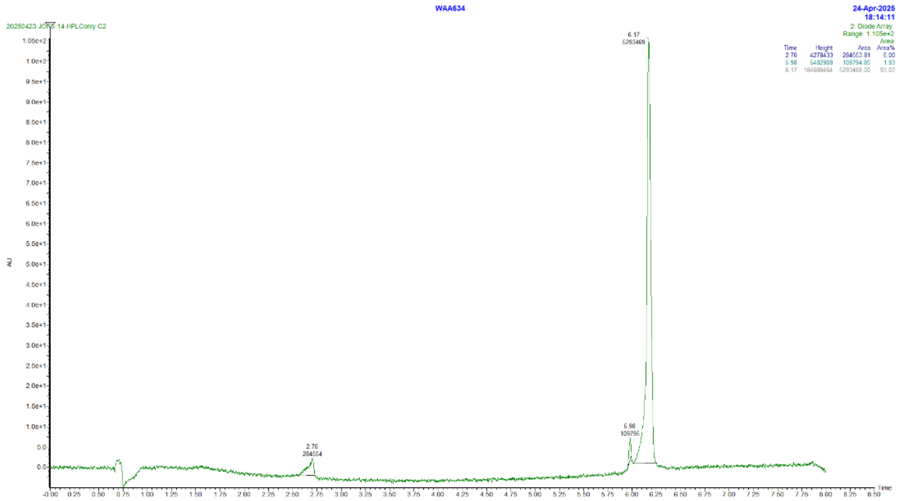

Figure S182: HPLC Chromatogram of compound **8a**.

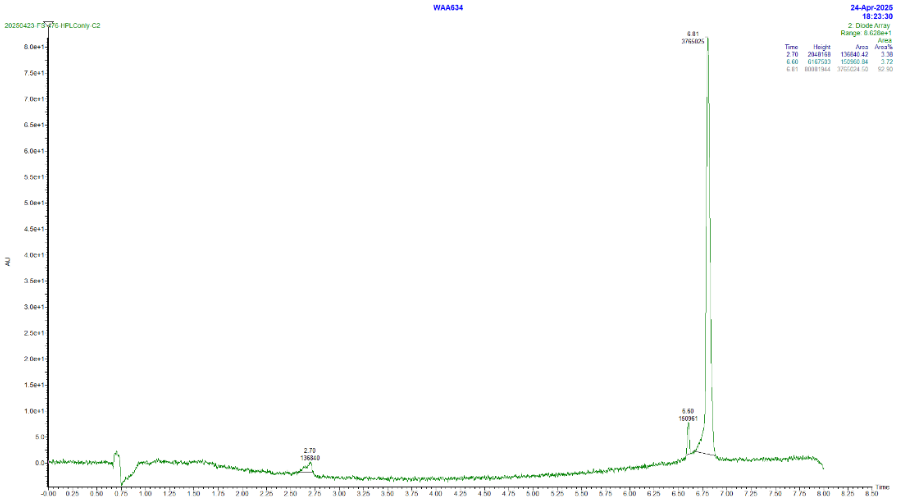

Figure S183: HPLC Chromatogram of compound **8b**.

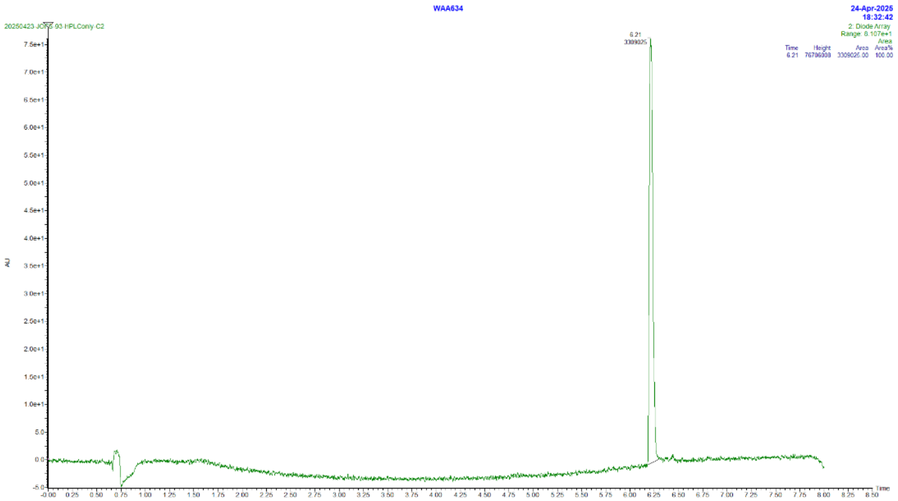

Figure S184: HPLC Chromatogram of compound **9a**.

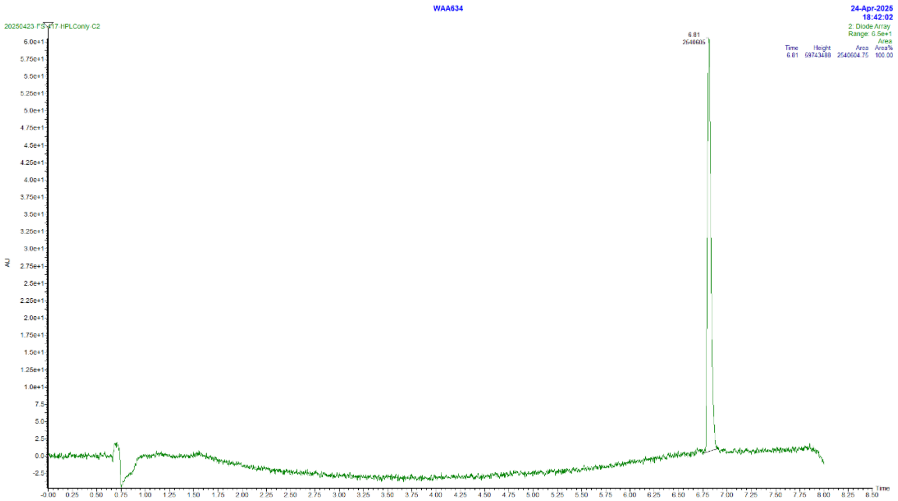

Figure S 185: HPLC Chromatogram of compound **9b**.

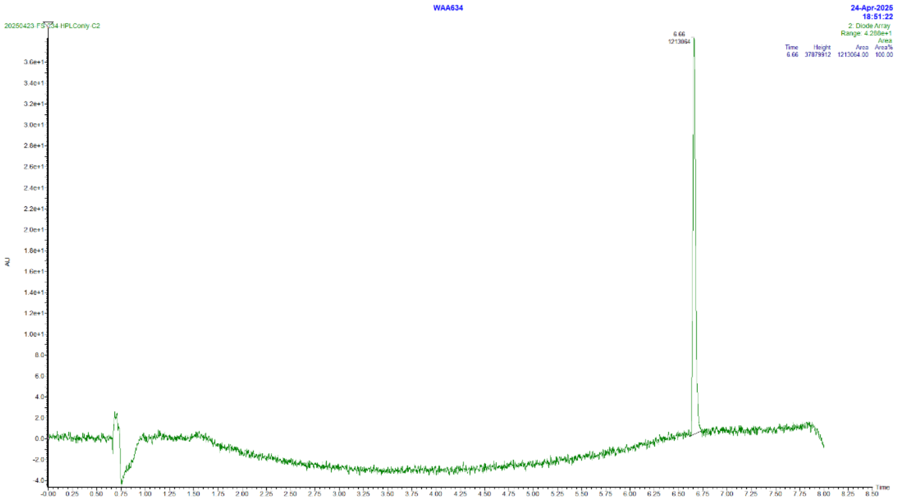

Figure S186: HPLC Chromatogram of compound **10a**.

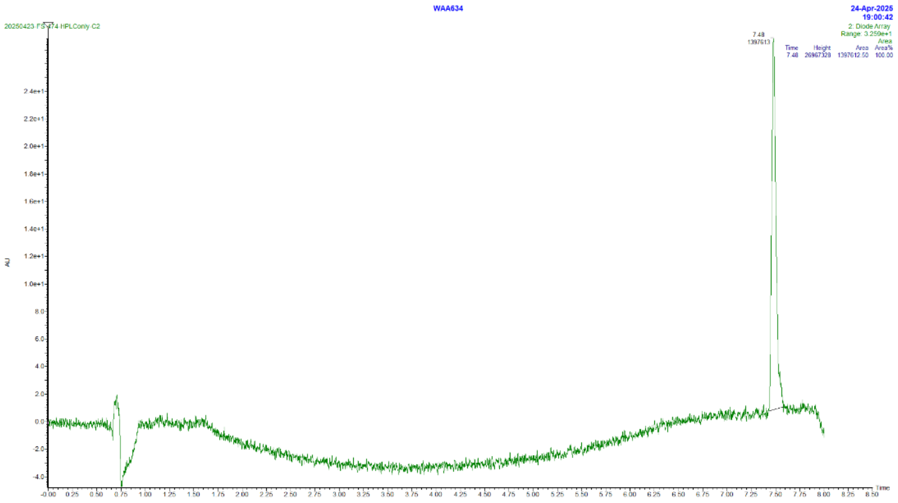

Figure S187: HPLC Chromatogram of compound **10b**.

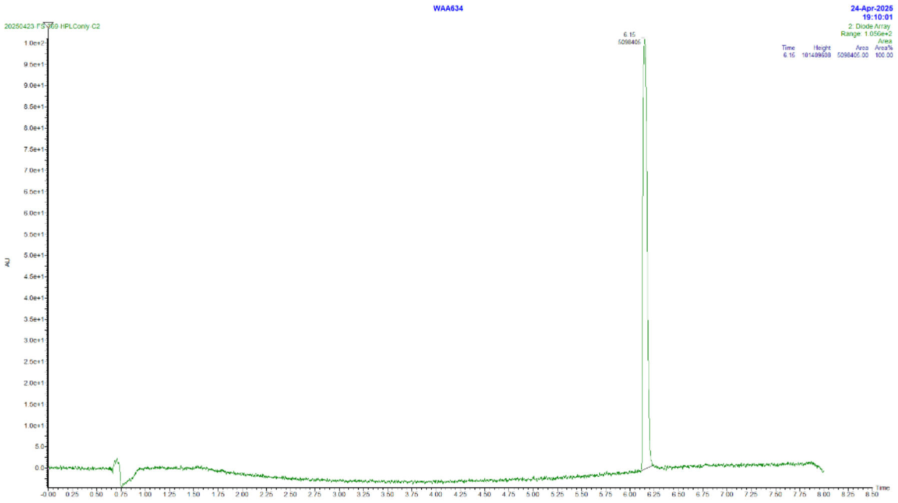

Figure S188: HPLC Chromatogram of compound **11a**.

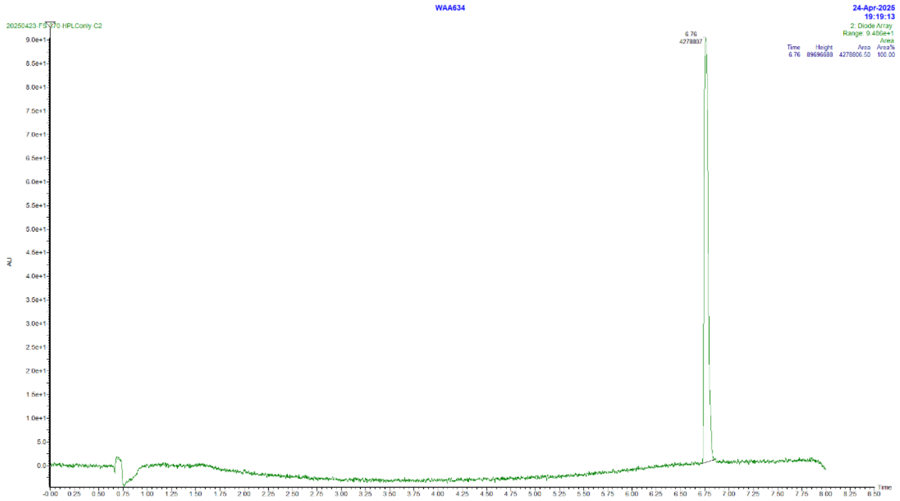

Figure S189: HPLC Chromatogram of compound **11b**.

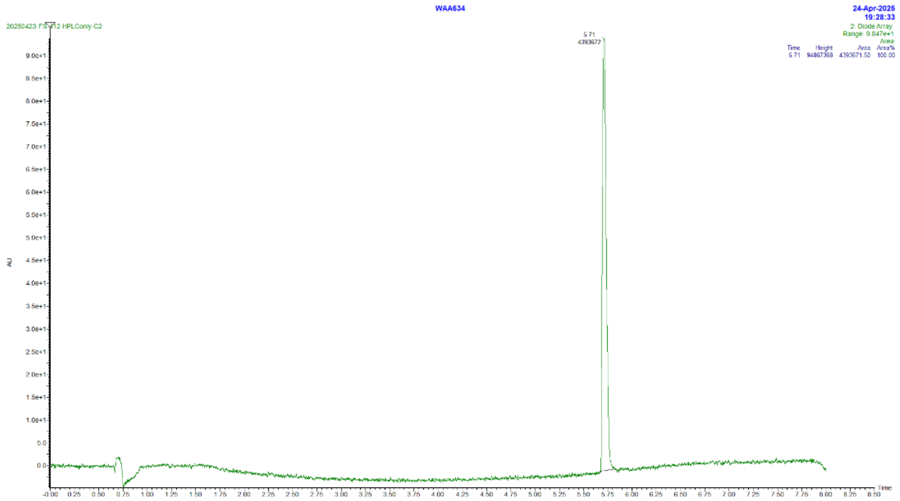

Figure S190: HPLC Chromatogram of compound **12a**.´

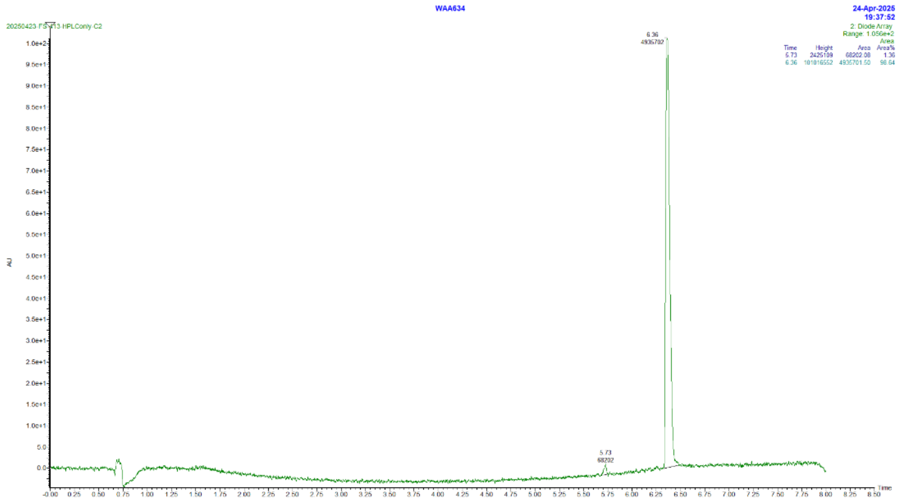

Figure S191: HPLC Chromatogram of compound **12b**.

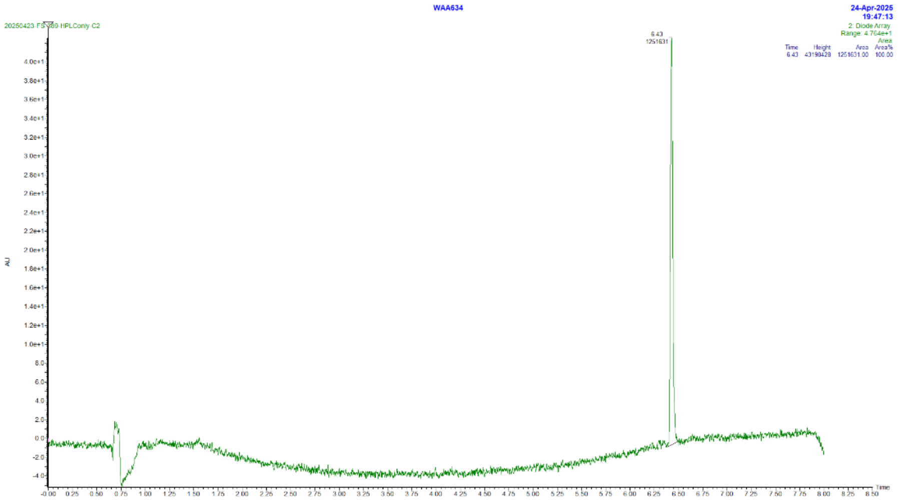

Figure S192: HPLC Chromatogram of compound **13a**.

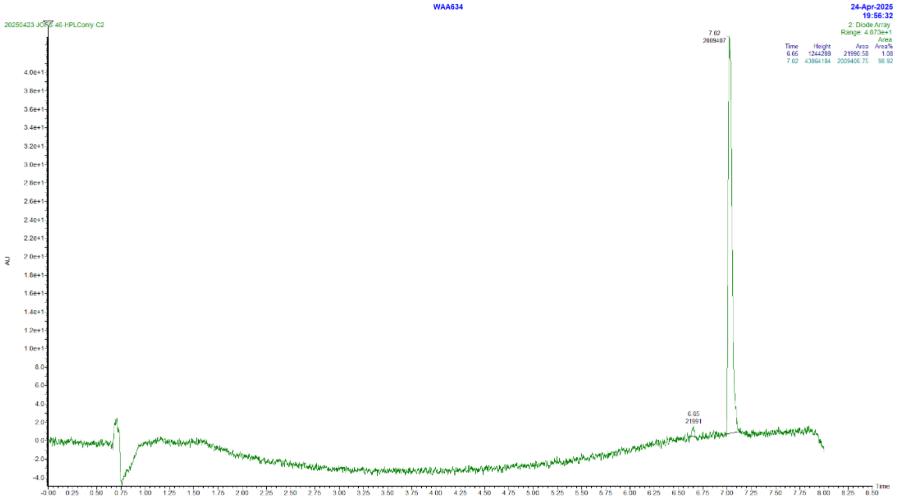

Figure S193: HPLC Chromatogram of compound **13b**.

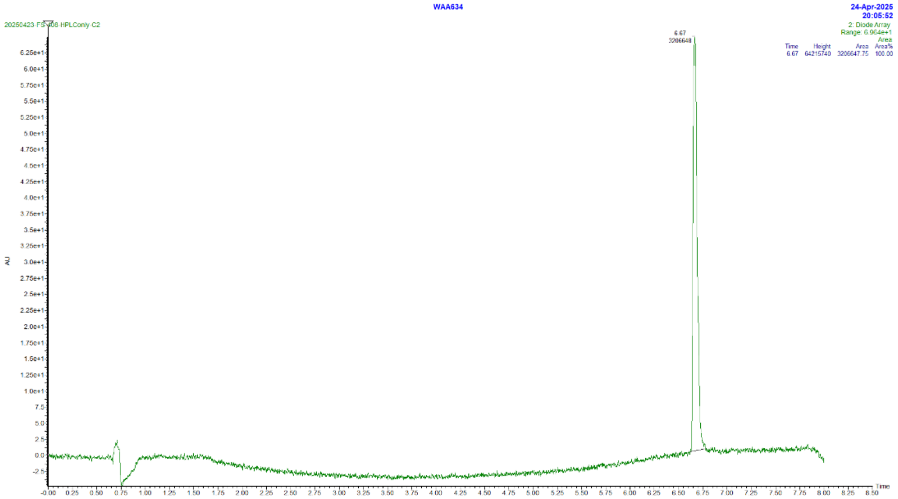

Figure S194: HPLC Chromatogram of compound **14a**.

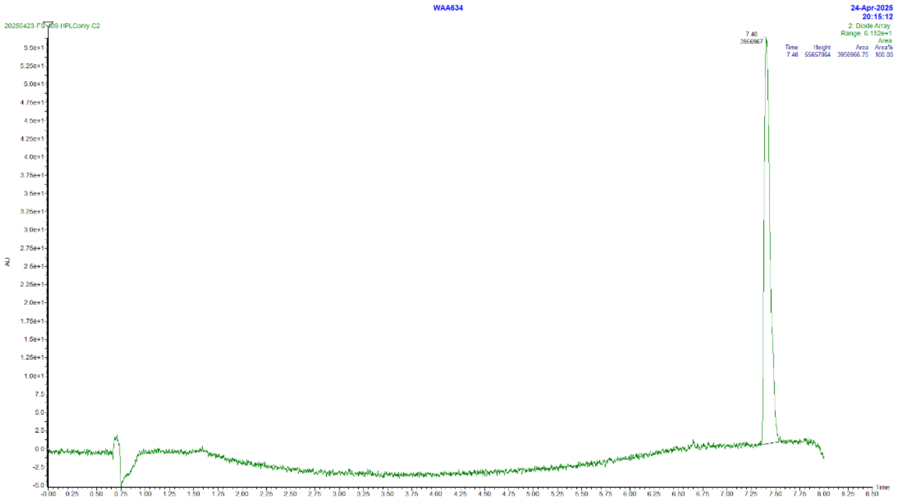

Figure S195: HPLC Chromatogram of compound **14b**.

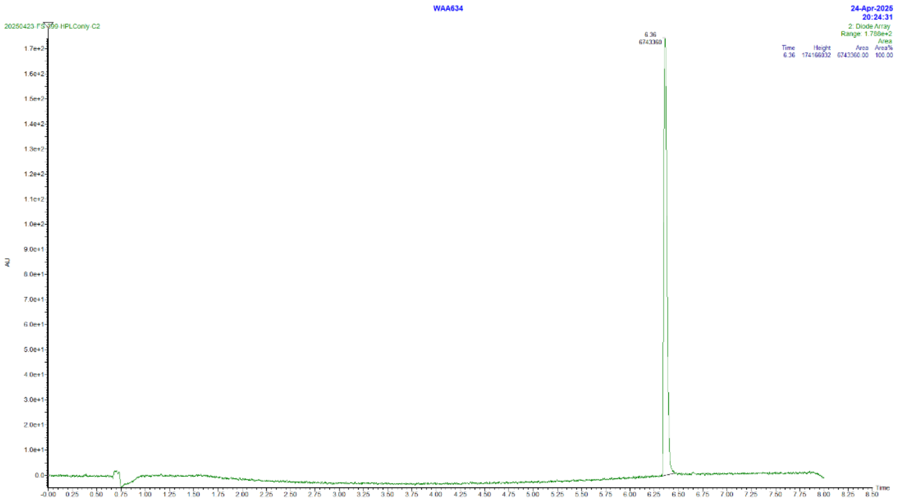

Figure S196: HPLC Chromatogram of compound **15a**.

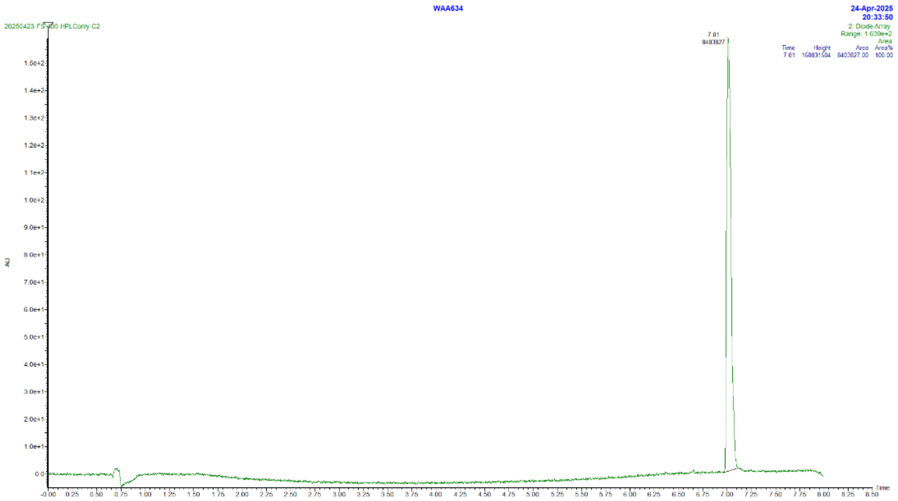

Figure S197: HPLC Chromatogram of compound **15b**.

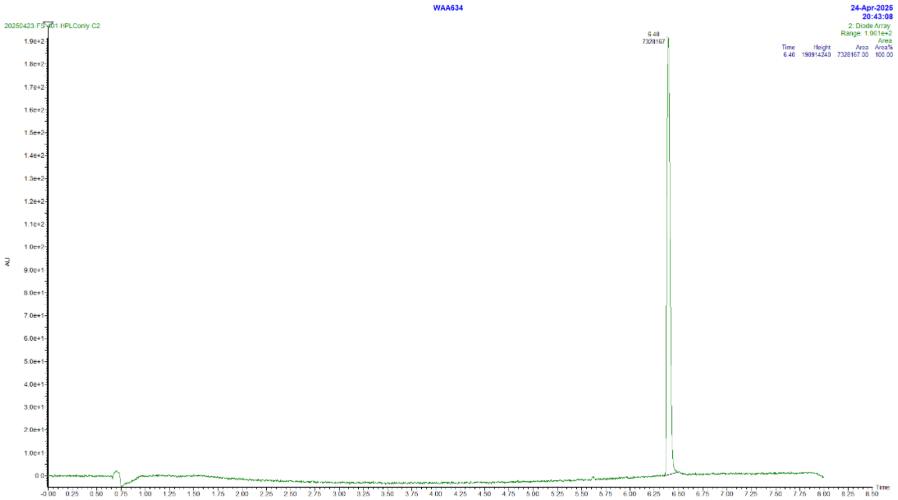

Figure S198: HPLC Chromatogram of compound **16a**.

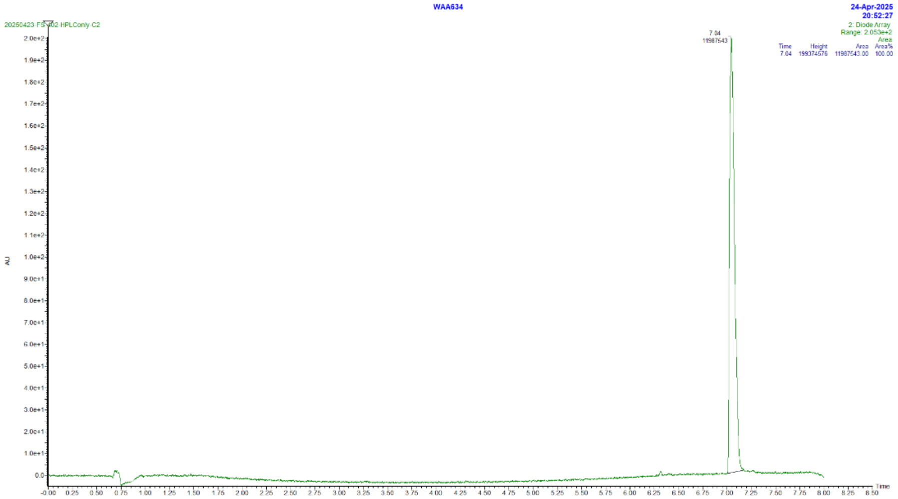

Figure S199: HPLC Chromatogram of compound **16b**.

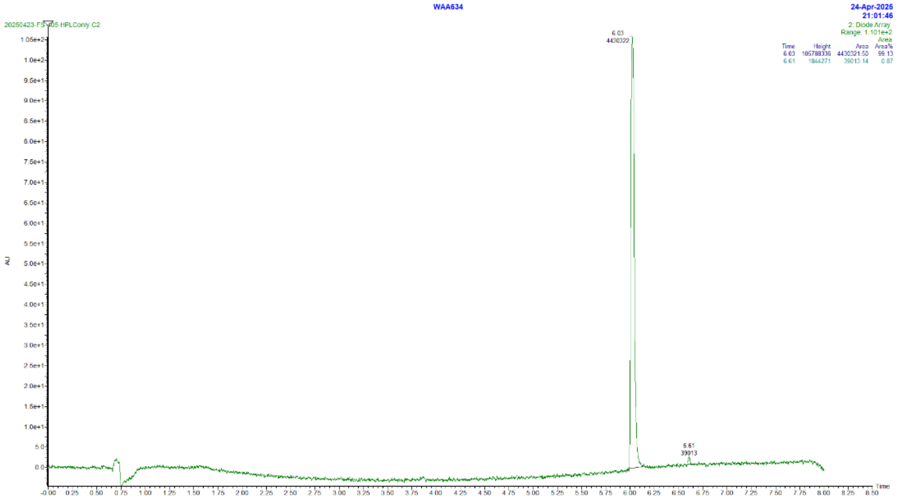

Figure S200: HPLC Chromatogram of compound **17a**.

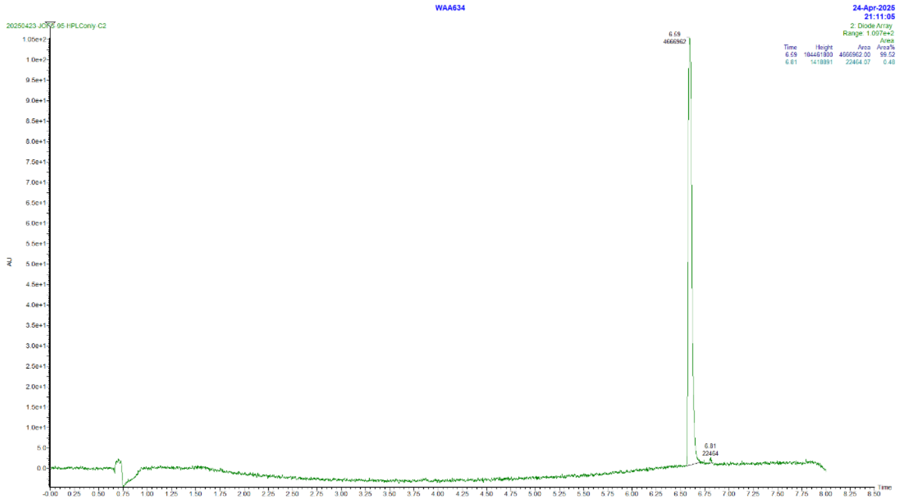

Figure S201: HPLC Chromatogram of compound **17b**.

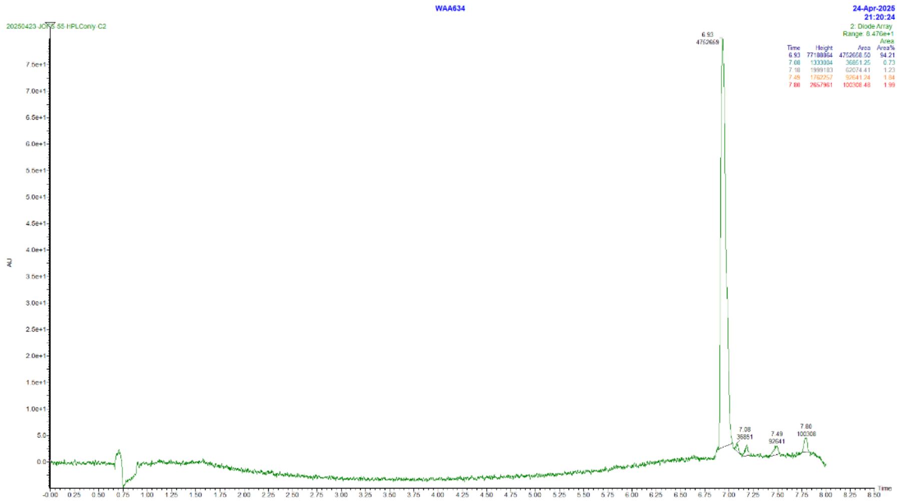

Figure S202: HPLC Chromatogram of compound **18a**.

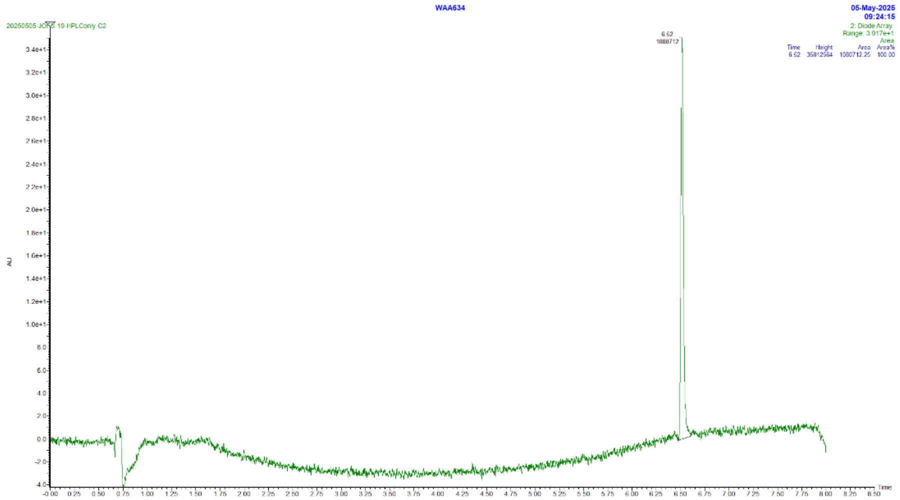

Figure S203:HPLC Chromatogram of compound **19a**.

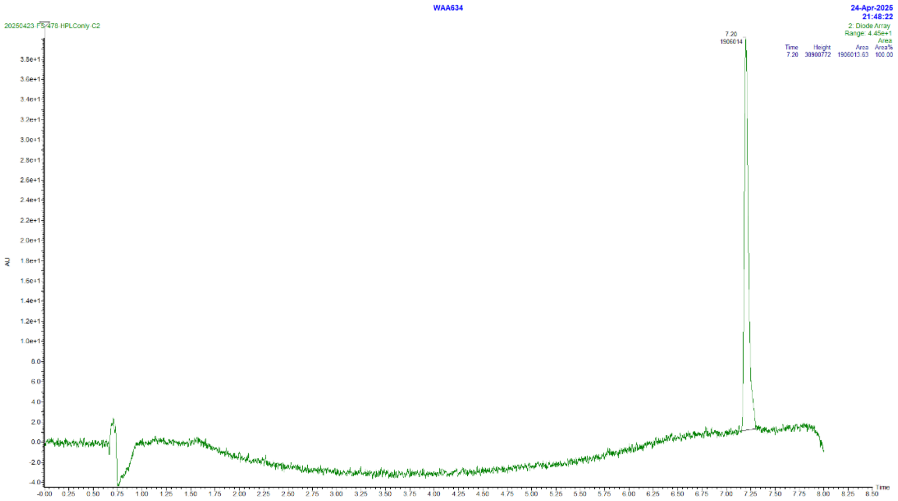

Figure S204: HPLC Chromatogram of compound **19b**.

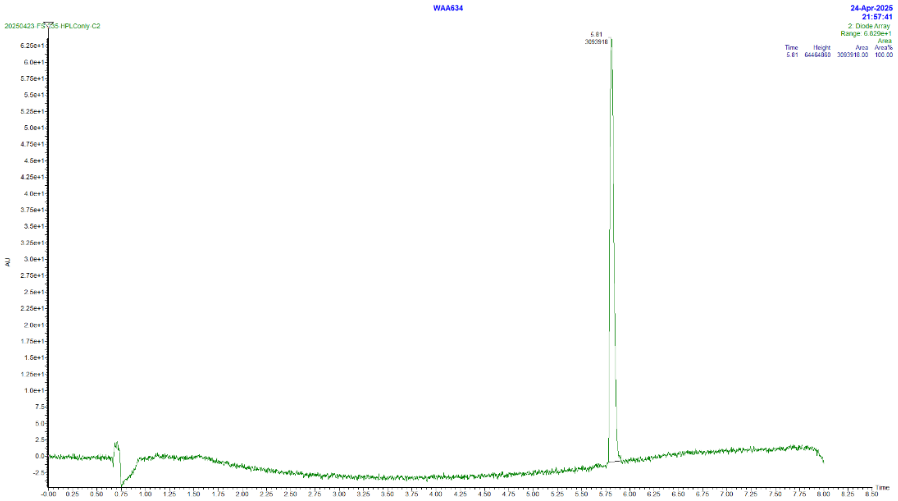

Figure S205: HPLC Chromatogram of compound **20a**.

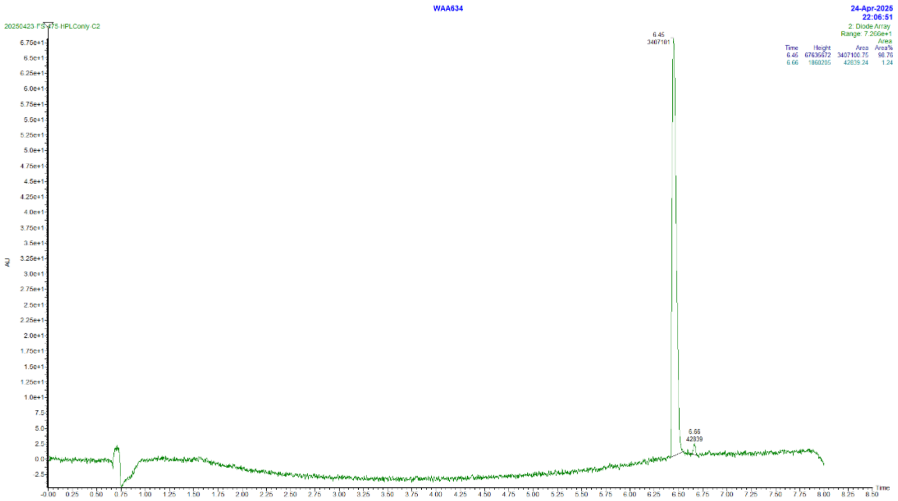

Figure S206: HPLC Chromatogram of compound **20b**.

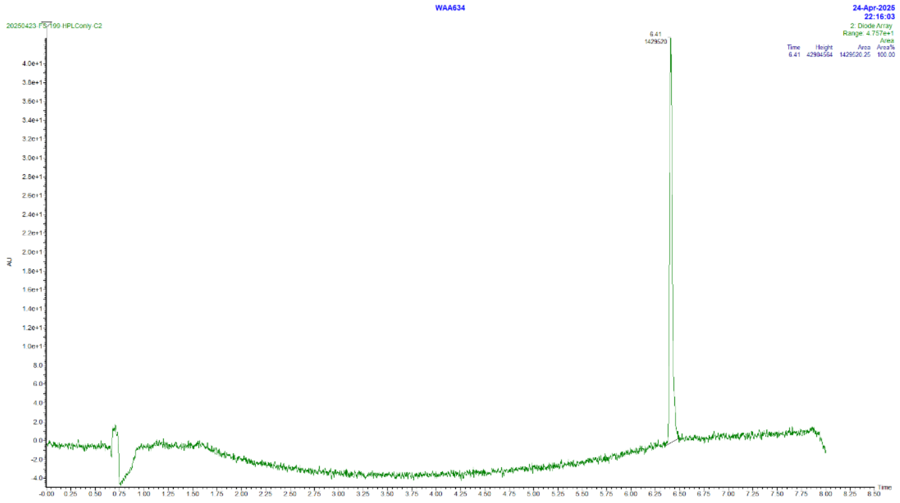

Figure S207: HPLC Chromatogram of compound **21a**.

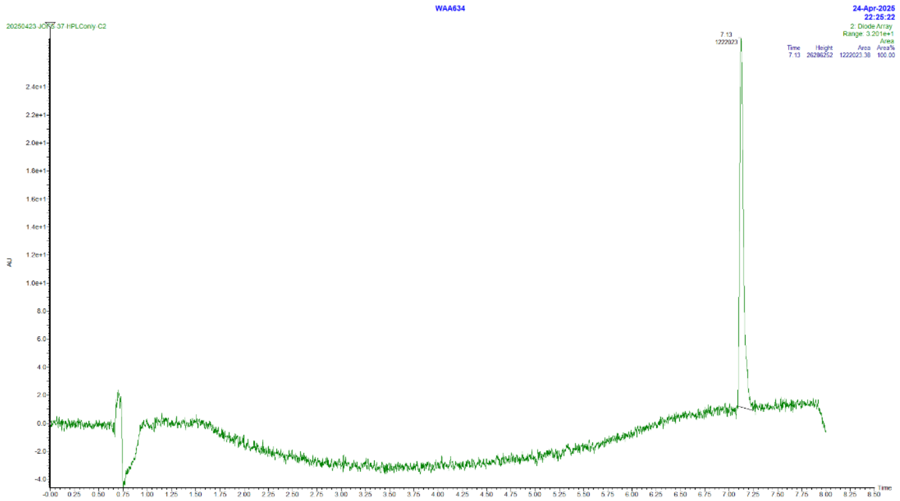

Figure S208: HPLC Chromatogram of compound **21b**.

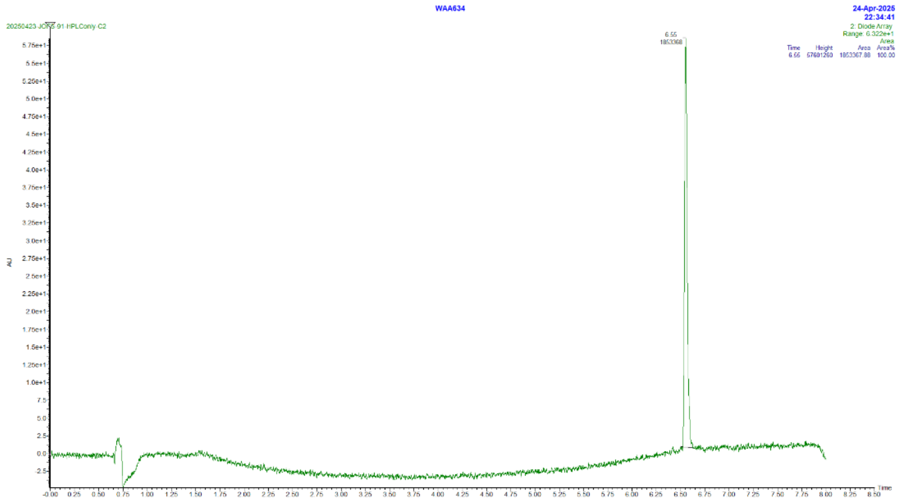

Figure S209: HPLC Chromatogram of compound **22a**.

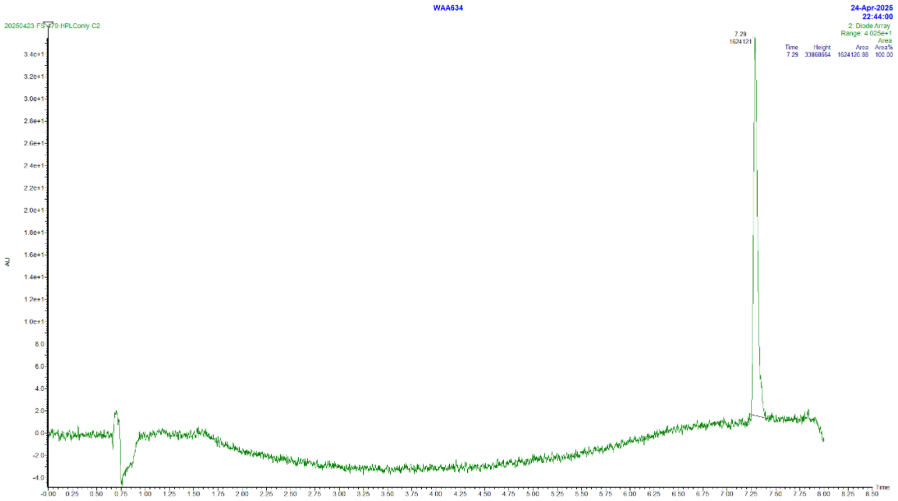

Figure S210: HPLC Chromatogram of compound **22b**.

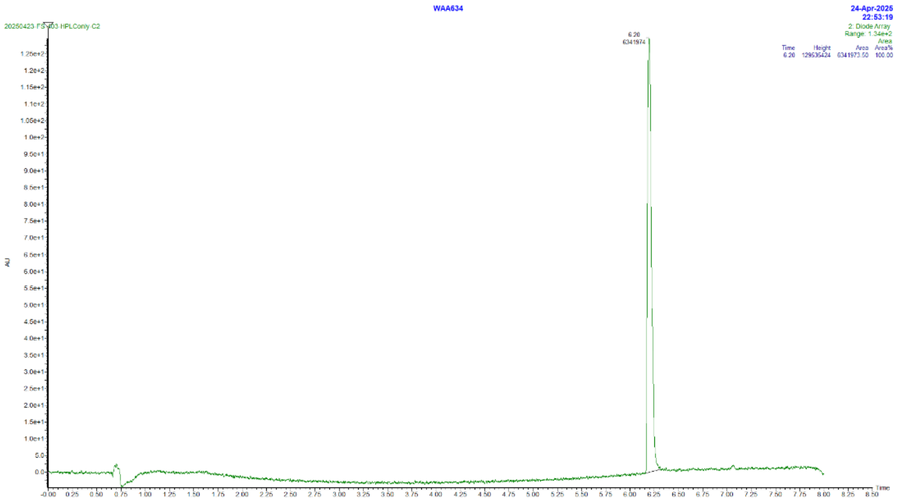

Figure S211: HPLC Chromatogram of compound **23a**.

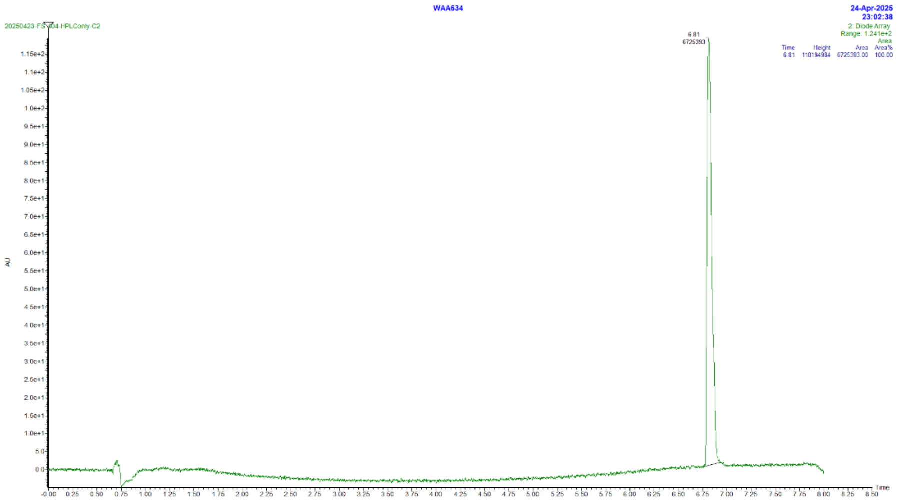

Figure S212: HPLC Chromatogram of compound **23b**.

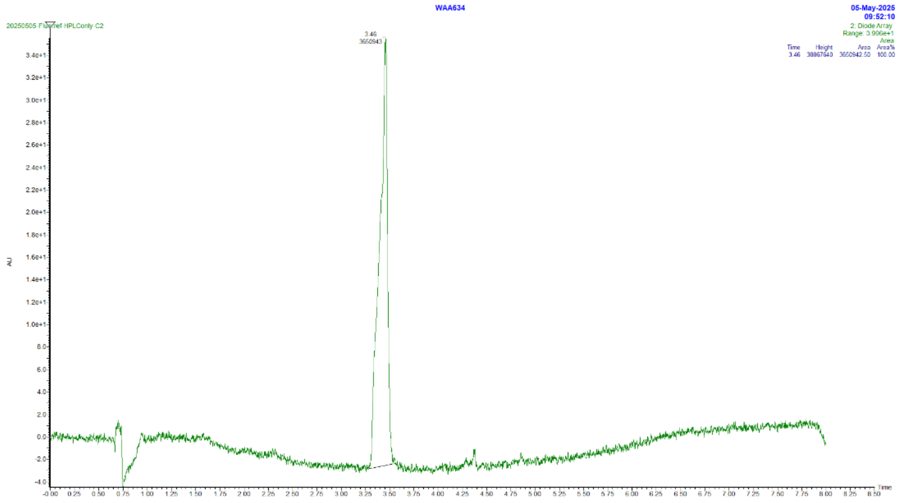

Figure S213: HPLC Chromatogram of compound **33**.

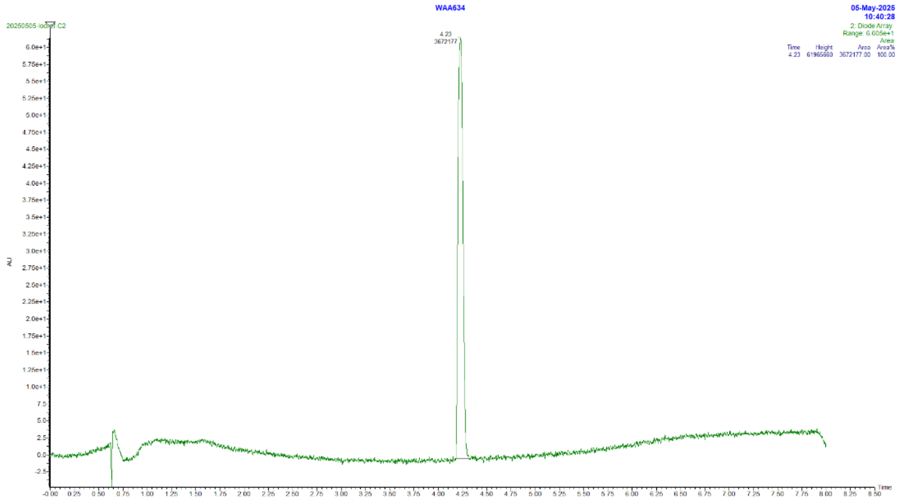

Figure S214: HPLC Chromatogram of compound **35**.

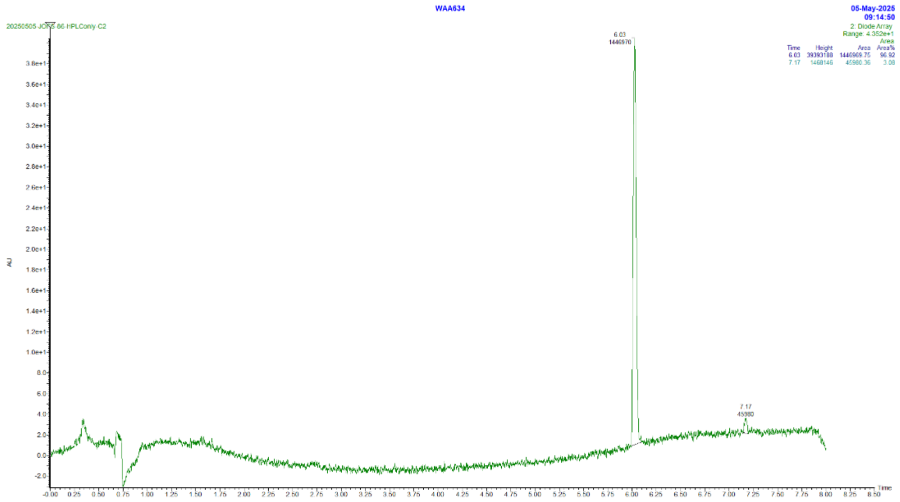

Figure S215: HPLC Chromatogram of compound **36**.

Stability testing on TLC

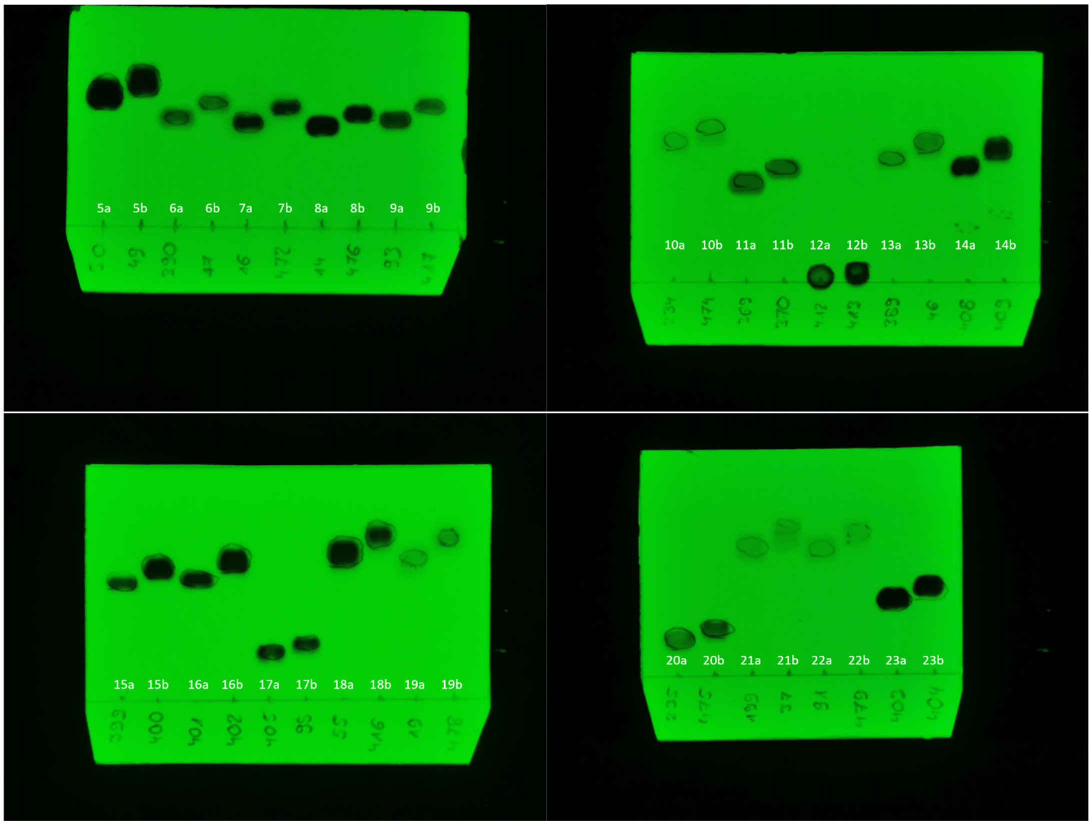

Figure S216: Normal phase thin layer chromatography of the labeling precursors on TLC plates ROTI®ChromaPlate Alu 60 UV with eluent cyclohexane:EtOAc 4:1.

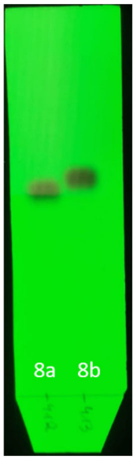

Figure S217: Normal phase thin layer chromatography of the labeling precursors on TLC plates ROTI®ChromaPlate Alu 60 UV with eluent EtOAc.

Radiochemistry

Optimization of CMRF Conditions: Phase Transfer Agents

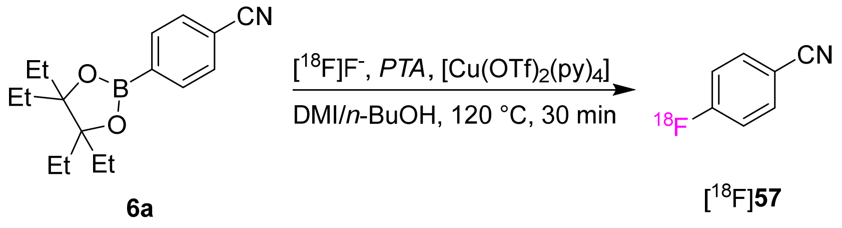

For this set of optimization experiments, the general optimized CMRF conditions described in the Materials and Methods section were applied, only the phase transfer agents (PTA) were varied.

Table S1. ^{18}F-Elution, radiochemical conversions (RCC) and radiochemical conversions (RCC) using different salts.

Salt	Run	^{18}F-Elution (%)	RCC (%) 30 min
Et_4NOTf	**1**	98%	70%
	**2**	97%	83%
	**3**	98%	82%
Et_4NHCO_3	**4**	98%	73%
	**5**	98%	75%
	**6**	97%	82%
$BnBu_3NCl$	**7**	99%	84%
	**8**	99%	80%
	**9**	99%	83%

Table S2. Summarized table of ^{18}F-Elution, and mean standard deviations of RCC values using different salts.

Salt	^{18}F-Elution (%)	RCC (%) 30 min
Et_4NOTf	98 ± 1% (n = 3)	78 ± 7% (n = 3)
Et_4NHCO_3	98 ± 1% (n = 3)	77 ± 7% (n = 3)
$BnBu_3NCl$	99% (n = 3)	82 ± 2% (n = 3)

Optimization of CMRF Conditions: Equivalents of Copper Mediator Compound

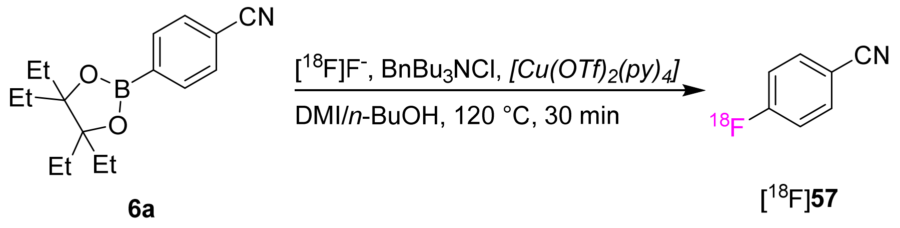

Table S3. RCC values using different Cu mediator equivalents.

Radiolabeling Precursor/Cu-Mediator Equivalents	Run	Cu-Mediator Concentration	RCC (%) 30 min
1:1	**1**	10 µmol	49%
	**2**	10 µmol	50%
	**3**	10 µmol	35%

1:1.5	**4**	15 µmol	68%
	**5**	15 µmol	69%
	**6**	15 µmol	64%
1:2	**7**	20 µmol	66%
	**8**	20 µmol	76%
	**9**	20 µmol	71%
1:2.5	**10**	25 µmol	84%
	**11**	25 µmol	80%
	**12**	25 µmol	83%
1:3	**13**	30 µmol	85%
	**14**	30 µmol	91%
	**15**	30 µmol	73%
	**16**	30 µmol	72%
1:3.5	**17**	35 µmol	59%
	**18**	35 µmol	81%
	**19**	35 µmol	84%

Table S4. Summarized table of mean standard deviations of RCC values using different Cu mediator equivalents.

Precursor/Cu-Mediator Equivalents	RCC (%) 30 min
1:1	45 ± 8% (n = 3)
1:1.5	67 ± 3% (n = 3)
1:2	71 ± 5% (n = 3)
1:2.5	82 ± 2% (n = 3)
1:3	80 ± 9% (n = 4)
1:3.5	75 ± 14% (n = 3)

Optimization of CMRF Conditions: Reaction Solvent

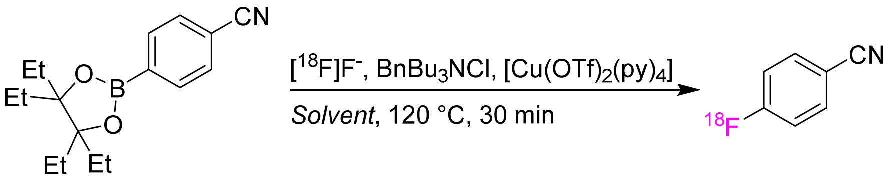

6a [^{18}F]**57**

For this set of optimization experiments, the general optimized CMRF conditions described in the Materials and Methods section were applied. The only deviations from the optimized CMRF conditions were that 20 µmol copper mediator compound concentrations (instead of 25 µmol) were used, and the reaction solvent systems were varied.

Table S5. RCC values using various reaction media solvents (1200 µL).

Solvent	Run	RCC (%) 30 min
DMI	**1**	59%
	**2**	40%
	**3**	38%
	**4**	27%

DMA	**5**	43%
	**6**	45%
DMA/*n*-BuOH (2:1)	**7**	35%
	**8**	34%
	**9**	26%
DMI/*n*-BuOH (2:1)	**10**	66%
	**11**	76%
	**12**	71%

Table S6. Summarized table of mean standard deviations of RCC values using various reaction media solvents.

Solvent	RCC (%) 30 min
DMI	46 ± 12%(n = 3)
DMA	38 ± 10% (n = 3)
DMA/*n*-BuOH (2:1)	32 ± 5% (n = 3)
DMI/*n*-BuOH (2:1)	71 ± 5% (n = 3)

Optimization of CMRF Conditions: Reaction Temperature

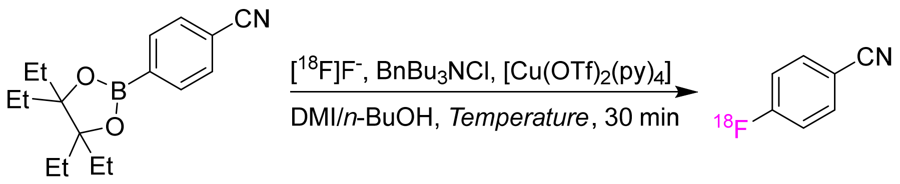

6a [18F]**57**

For this set of optimization experiments, the general optimized CMRF conditions described in the Materials and Methods section were applied. The only deviations from the optimized CMRF conditions were that 20 μmol copper mediator compound concentrations (instead of 25 μmol) were used, and the reaction temperatures were varied.

Table S7. RCC values at 30 min with various reaction temperatures.

Temperature	Run	RCC (%) 30 min
100 °C	**1**	62%
	**2**	52%
110 °C	**3**	65%
	**4**	67%
120 °C	**5**	78%
	**6**	84%
130 °C	**7**	55%
	**8**	67%

Table S8. Summarized table of mean standard deviations of RCC values at various reaction temperatures.

Temperature	RCC (%) 30 min
100 °C	57 ± 7% (n = 2)
110 °C	66 ± 1% (n = 2)
120 °C	81 ± 4% (n = 2)
130 °C	61 ± 8% (n = 2)

CMRH of established radiolabeling precursors

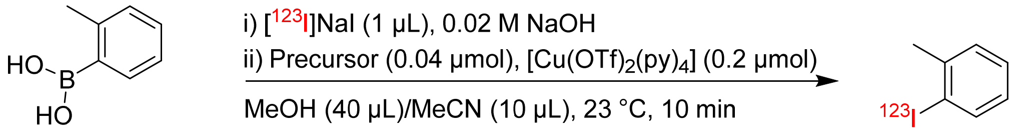

S2 [123I]**44**

For this set of experiments, the general optimized CMRI conditions described in the Materials and Methods section were applied.

Table S9. RCC values mean standard deviation (SD) at 10 min.

Run	RCC (%) 10 min
**1**	100%
**2**	100%
**3**	95%
**SD**	98 ± 3% (n = 3)

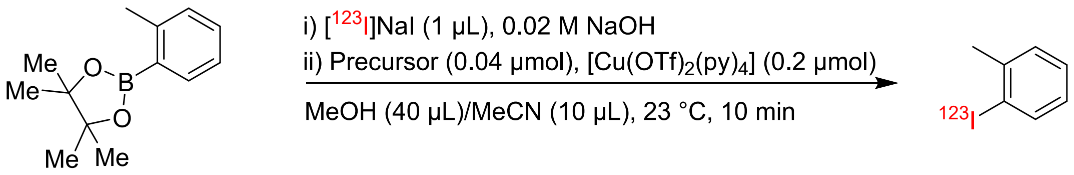

S3 [123I]**44**

For this set of experiments, the general optimized CMRI conditions described in the Materials and Methods section were applied.

Table S10. RCC values at 10 min.

Run	RCC (%) 10 min
**1**	87%
**2**	92%
**3**	92%
**SD**	90 ± 3% (n = 3)

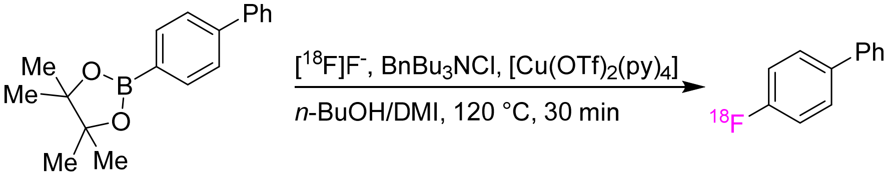

38 [18F]**58**

For this experiment, the general optimized CMRF conditions described in the Materials and Methods section were applied.

Table S11. RCC value at 30 min.

Run	RCC (%) 30 min
1	91%

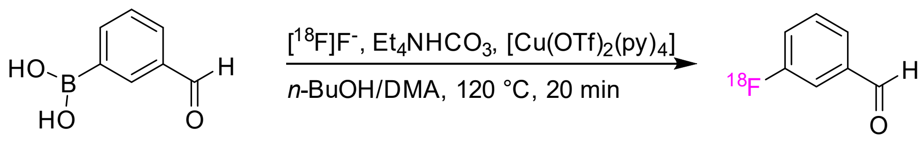

S4 $[^{18}F]$**61**

For this set of experiments, the general optimized CMRF conditions were not used. The deviation from the optimized reaction conditions include: Et_4NHCO_3 (1 mg) as a PTA, 1.0 mg (6.67 µmol) of radiolabeling precursor (**86**), 18.0 mg (26.54 µmol) of $[Cu(OTf)_2(py)_4]$, 1200 µL DMA/*n*-BuOH (2:1) reaction solvent and 25 min reaction time.

Table S12. RCC values at 20 min.

Run	RCC (%) 20 min
1	88%
2	88%
SD	88% (n = 2)

Chromatograms showing radioiodinated byproduct

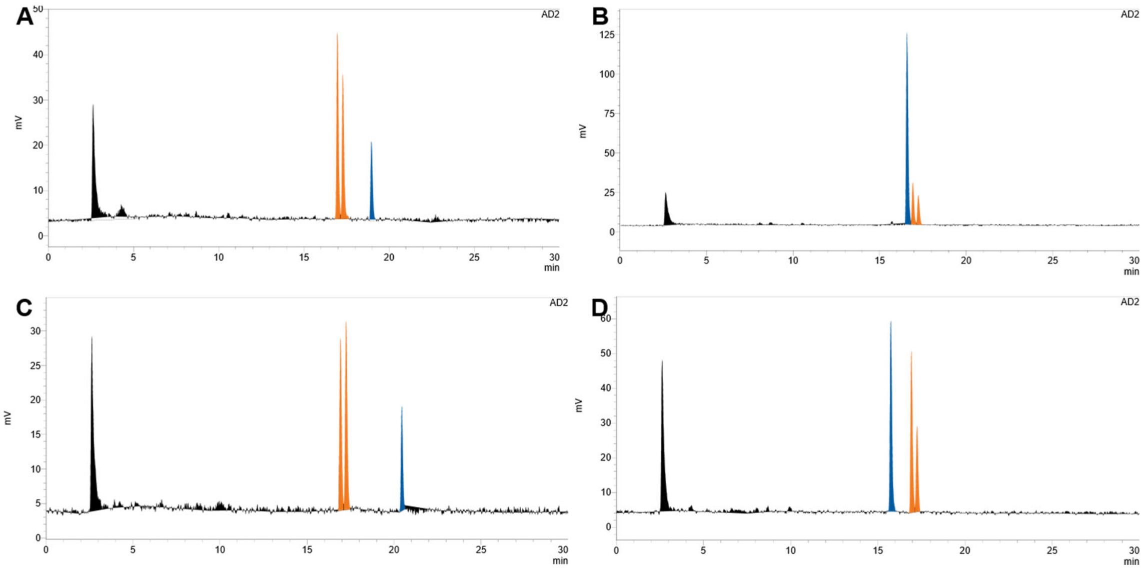

Figure S218: Radio-HPLC chromatograms of compounds (**A**) $[^{123}I]$**44**; (**B**) $[^{123}I]$**49**; (**C**) $[^{123}I]$**48**; and (**D**) $[^{123}I]$**57**, with the respective desired product in blue and the observed common side products in orange.

Radio-HPLC and Radio-UHPLC Chromatograms

HPLC & UHPLC Data

HPLC & UHPLC experiments were performed according to the specified gradients outlined in the materials and methods section in the manuscript.

HPLC & UHPLC Chromatograms of ^{123}I-Labeled Compounds

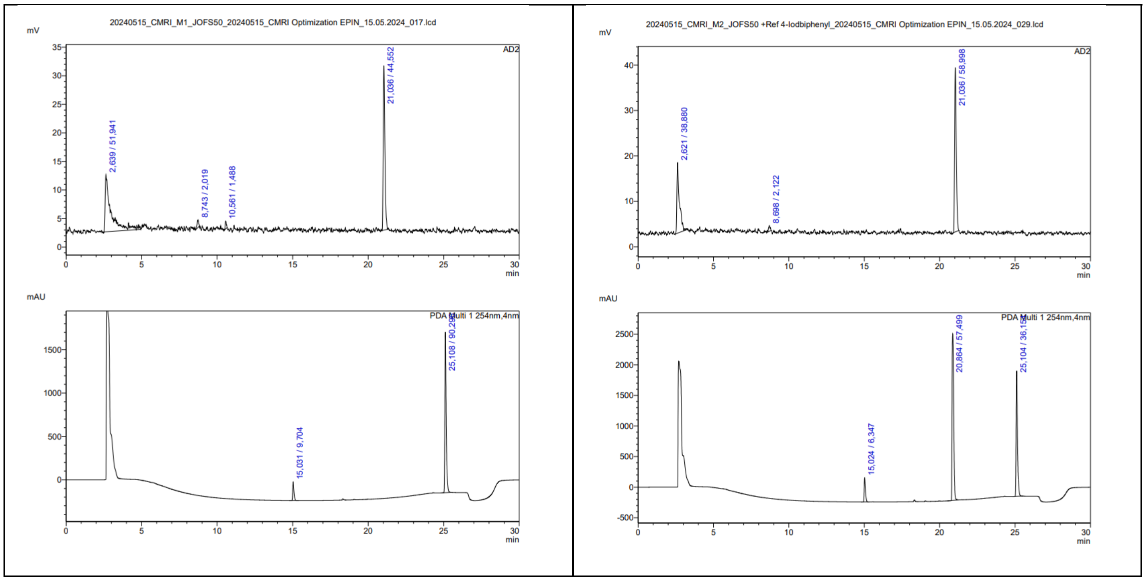

Figure S219: Copy of analytical HPLC chromatograms (Gradient C) of the crude reaction mixture (**upper panel**: signal of gamma-detector; **lower panel**: UV-signal) obtained for [^{123}I]**39** prepared from **5a** without (**left**) and with (**right**) addition of the authentic non-radioactive reference. In the HPLC setup, the UV detector is in row before the γ-detector with Δt_R of 0.17-0.18 min between both detectors.

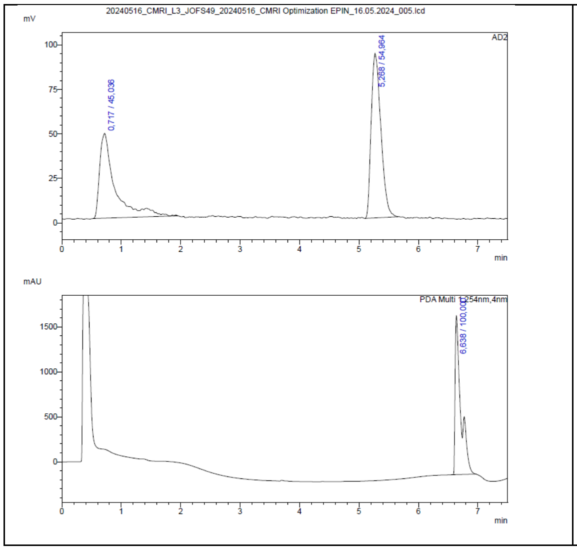

Not determined

Figure S220: Copy of analytical (U)HPLC chromatograms (Gradient A) of the crude reaction mixture (**upper panel**: signal of gamma-detector; **lower panel**: UV-signal) obtained for [^{123}I]**39** prepared from **5b** without (**left**) and with (**right**) addition of the authentic non-radioactive reference. In the HPLC setup, the UV detector is in row before the γ-detector with Δt_R of 0.3 min between both detectors.

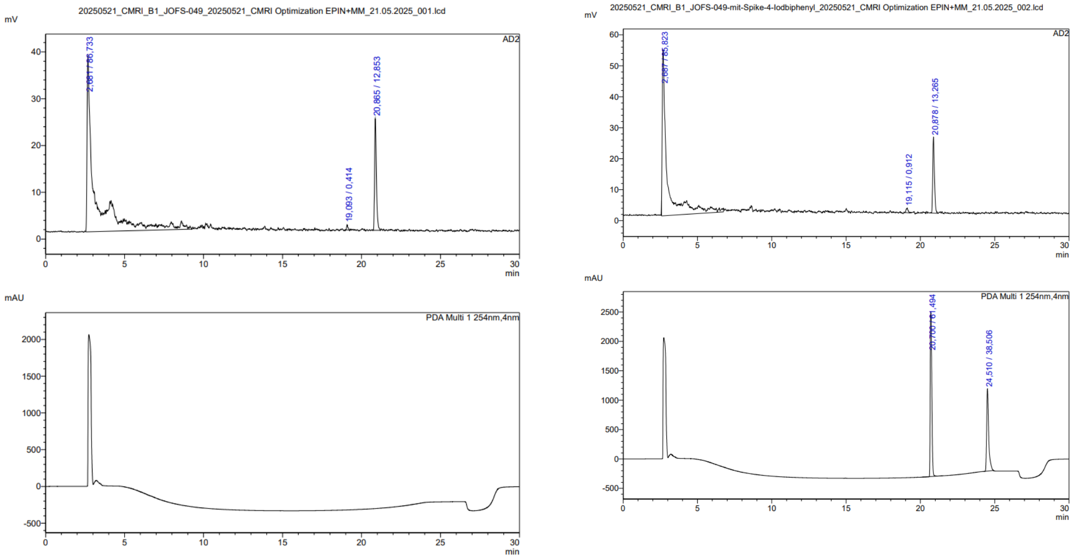

Figure S221: Copy of analytical HPLC chromatograms (Gradient C) of the crude reaction mixture (**upper panel**: signal of gamma-detector; **lower panel**: UV-signal) obtained for [^{123}I]**39** prepared from **5b** without (**left**) and with (**right**) addition of the authentic non-radioactive reference. In the HPLC setup, the UV detector is in row before the γ-detector with Δt_R of 0.17-0.18 min between both detectors.

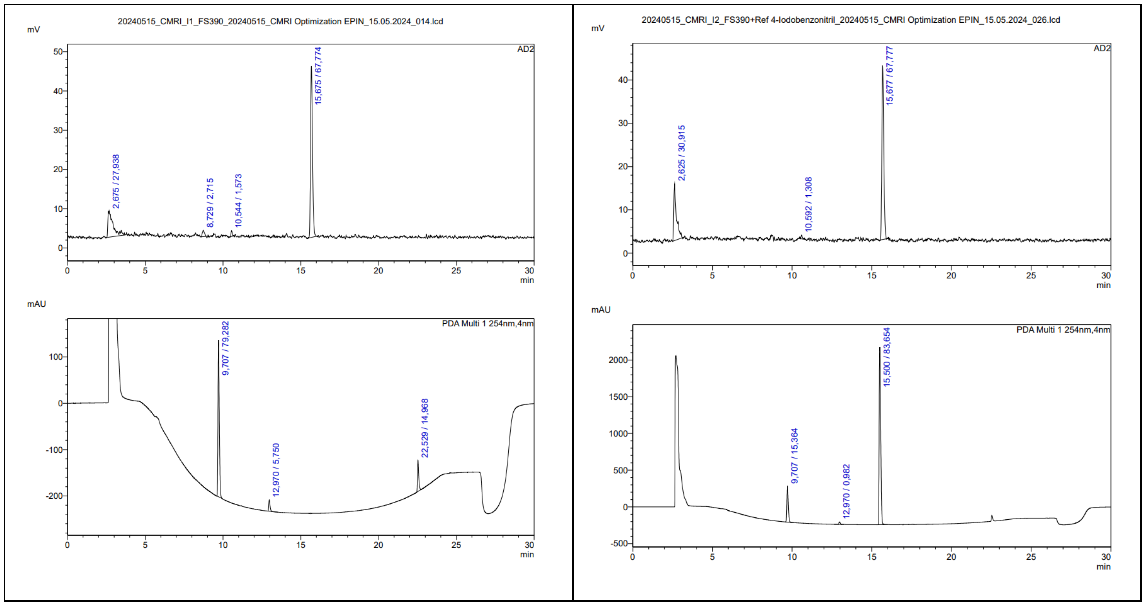

Figure S222: Copy of analytical HPLC chromatograms (Gradient C) of the crude reaction mixture (**upper panel**: signal of gamma-detector; **lower panel**: UV-signal) obtained for [^{123}I]**40** prepared from **6a** without (**left**) and with (**right**) addition of the authentic non-radioactive reference. In the HPLC setup, the UV detector is in row before the γ-detector with Δt_R of 0.17-0.18 min between both detectors.

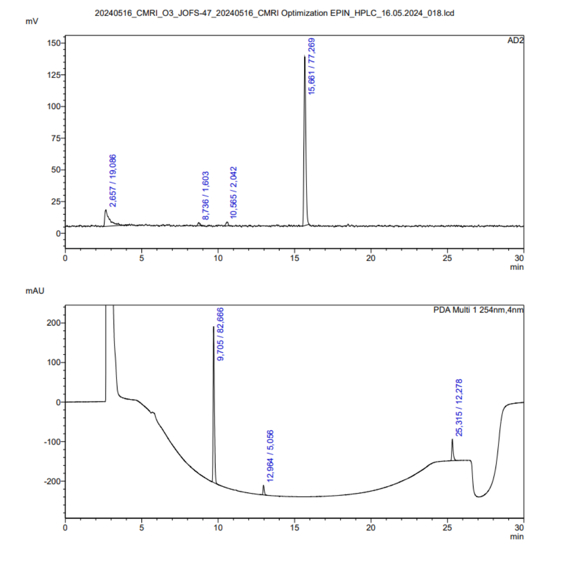

Not determined

Figure S223: Copy of analytical HPLC chromatograms (Gradient C) of the crude reaction mixture (**upper panel**: signal of gamma-detector; **lower panel**: UV-signal) obtained for [^{123}I]**40** prepared from **6b** without addition of the authentic non-radioactive reference. In the HPLC setup, the UV detector is in row before the γ-detector with Δt_R of 0.17-0.18 min between both detectors.

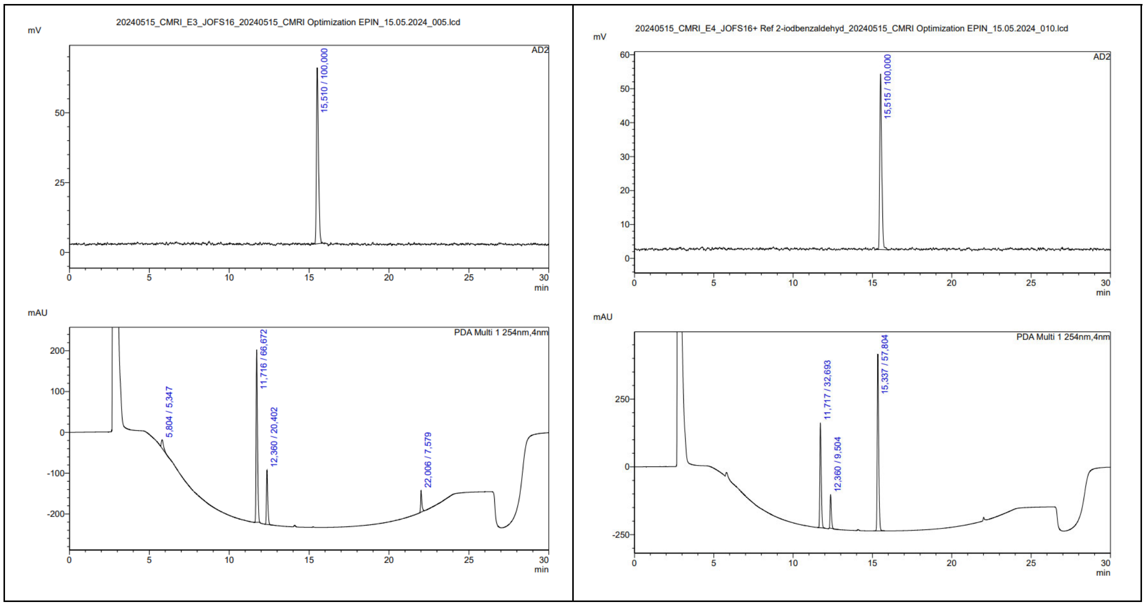

Figure S224: Copy of analytical HPLC chromatograms (Gradient C) of the crude reaction mixture (**upper panel**: signal of gamma-detector; **lower panel**: UV-signal) obtained for [^{123}I]**41** prepared from **7a** without (**left**) and with (**right**) addition of the authentic non-radioactive reference. In the HPLC setup, the UV detector is in row before the γ-detector with Δt_R of 0.17-0.18 min between both detectors.

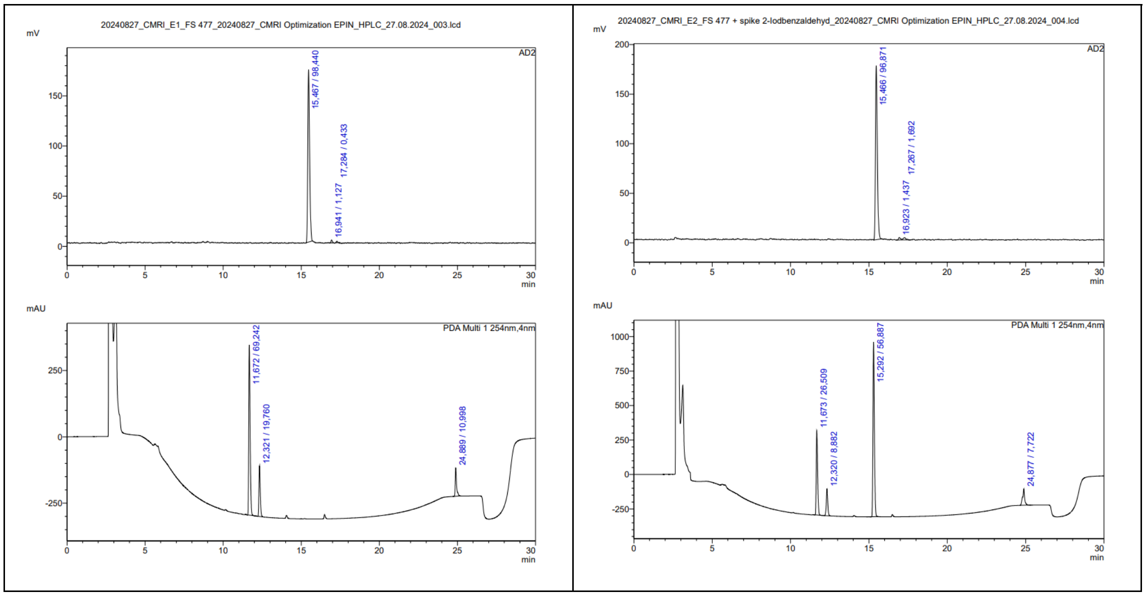

Figure S225: Copy of analytical HPLC chromatograms (Gradient C) of the crude reaction mixture (**upper panel**: signal of gamma-detector; **lower panel**: UV-signal) obtained for [^{123}I]**41** prepared from **7b** without (**left**) and with (**right**) addition of the authentic non-radioactive reference. In the HPLC setup, the UV detector is in row before the γ-detector with Δt$_R$ of 0.17-0.18 min between both detectors.

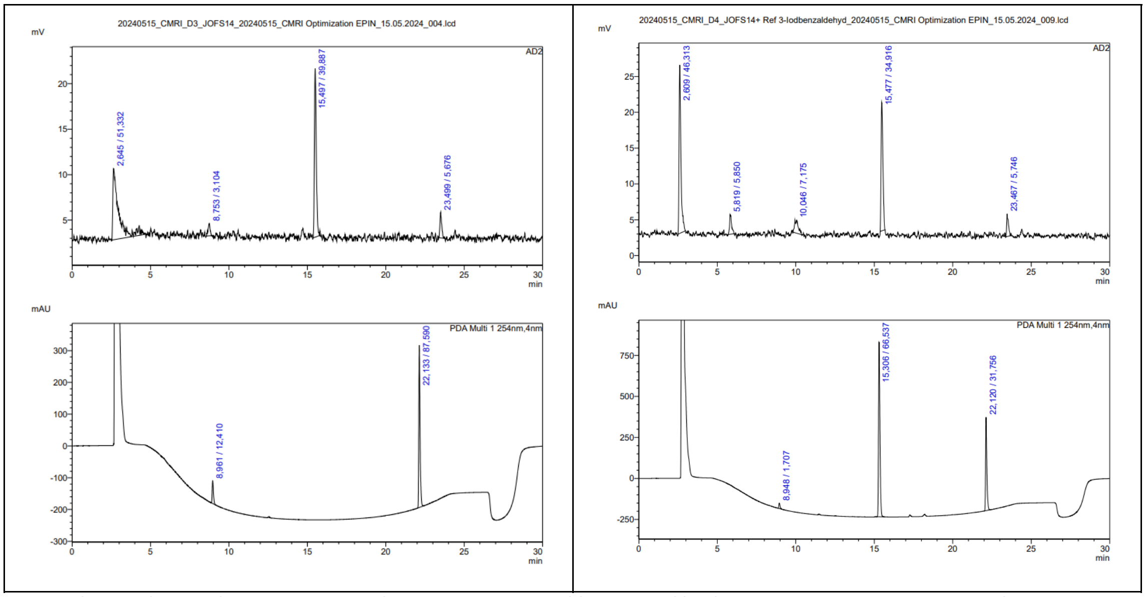

Figure S226: Copy of analytical HPLC chromatograms (Gradient C) of the crude reaction mixture (**upper panel**: signal of gamma-detector; **lower panel**: UV-signal) obtained for [^{123}I]**42** prepared from **8a** without (**left**) and with (**right**) addition of the authentic non-radioactive reference. In the HPLC setup, the UV detector is in row before the γ-detector with Δt$_R$ of 0.17-0.18 min between both detectors.

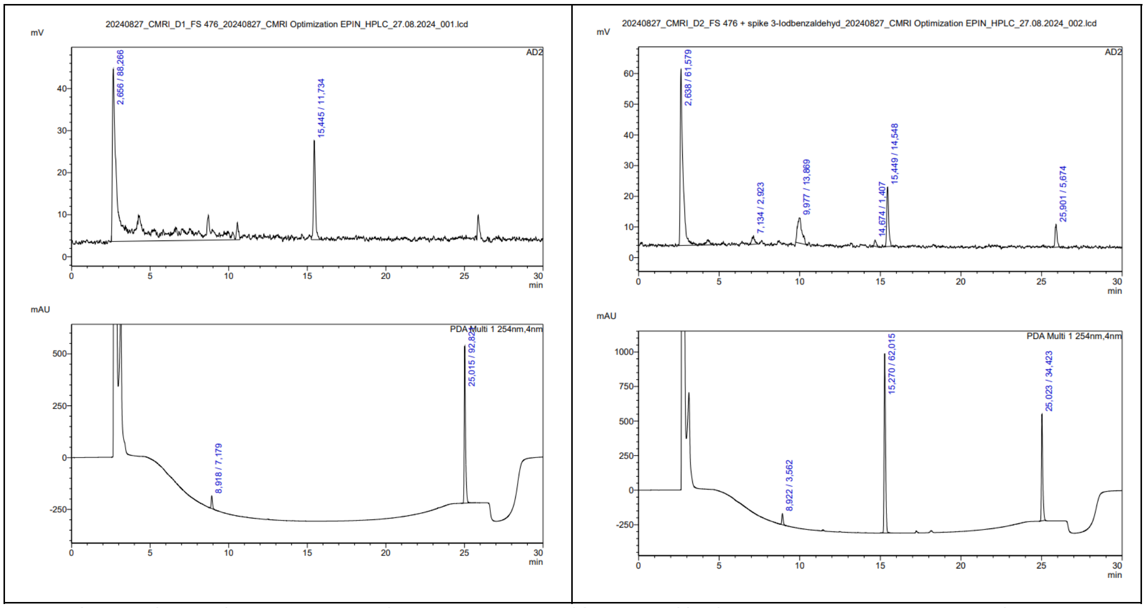

Figure S 227: Copy of analytical HPLC chromatograms (Gradient C) of the crude reaction mixture (**upper panel**: signal of gamma-detector; **lower panel**: UV-signal) obtained for [^{123}I]**42** prepared from **8b** without (**left**) and with (**right**) addition of the authentic non-radioactive reference. In the HPLC setup, the UV detector is in row before the γ-detector with Δt_R of 0.17-0.18 min between both detectors.

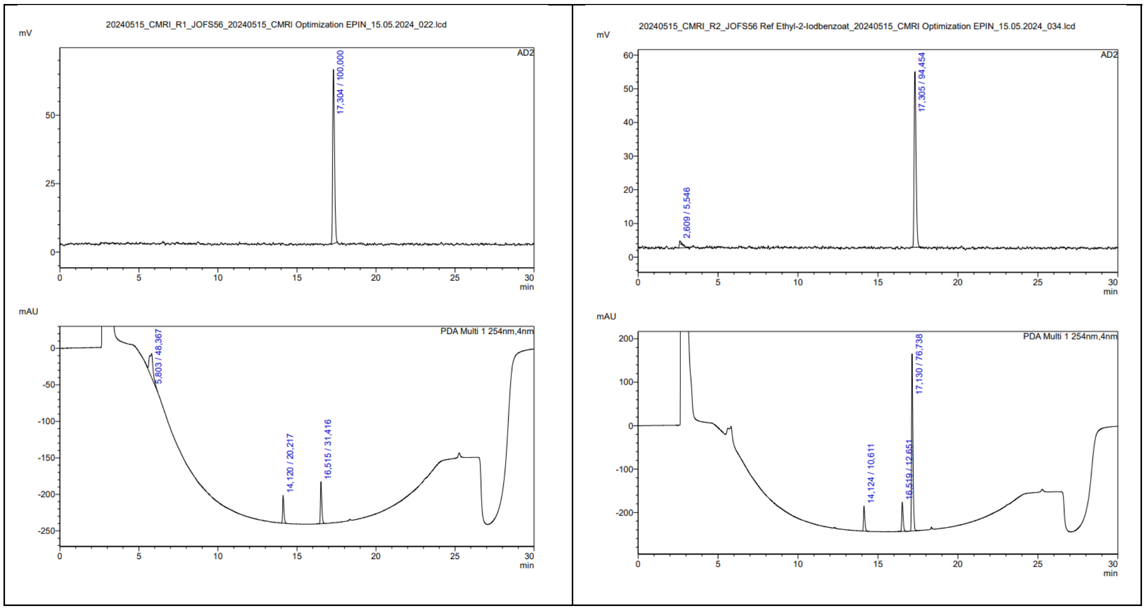

Figure S228: Copy of analytical HPLC chromatograms (Gradient C) of the crude reaction mixture (**upper panel**: signal of gamma-detector; **lower panel**: UV-signal) obtained for [^{123}I]**43** prepared from **9a** without (**left**) and with (**right**) addition of the authentic non-radioactive reference. In the HPLC setup, the UV detector is in row before the γ-detector with Δt_R of 0.17-0.18 min between both detectors.

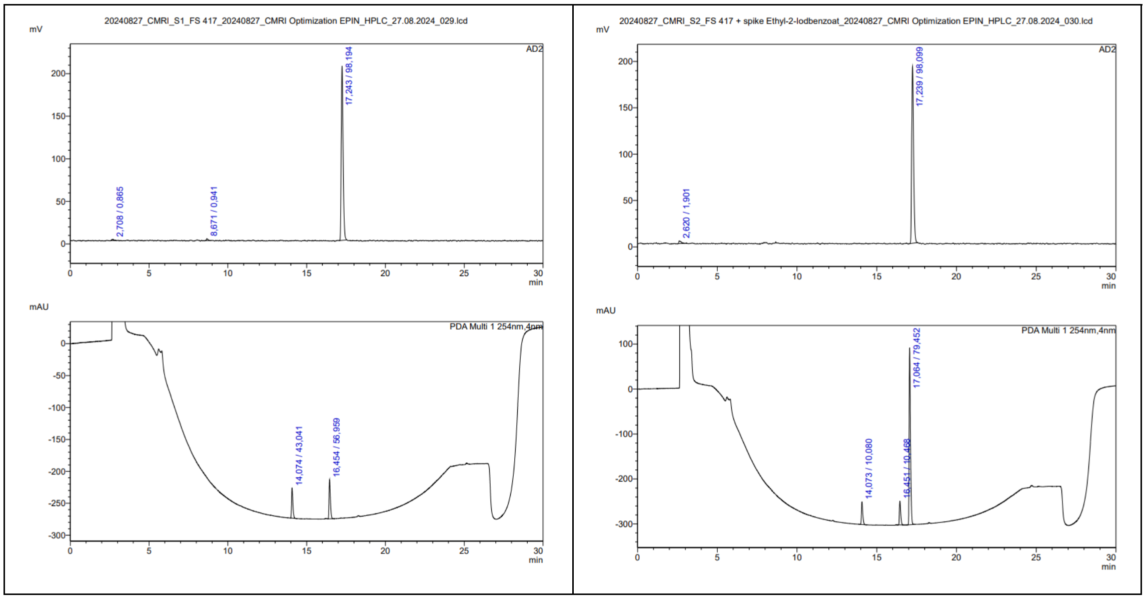

Figure S229: Copy of analytical HPLC chromatograms (Gradient C) of the crude reaction mixture (**upper panel**: signal of gamma-detector; **lower panel**: UV-signal) obtained for [123I]**43** prepared from **9b** without (**left**) and with (**right**) addition of the authentic non-radioactive reference. In the HPLC setup, the UV detector is in row before the γ-detector with Δt_R of 0.17-0.18 min between both detectors.

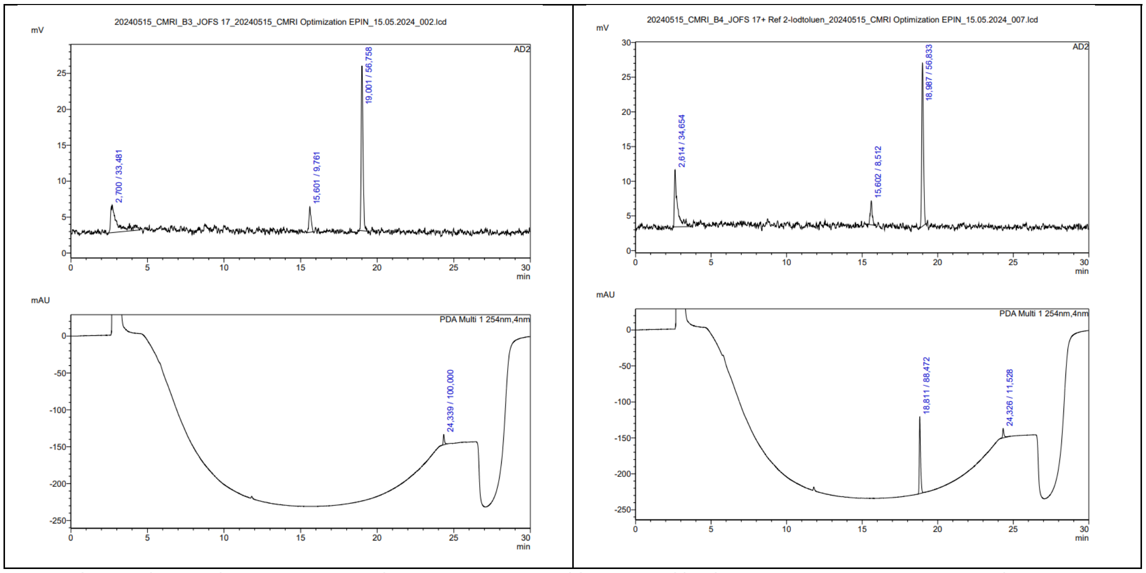

Figure S230: Copy of analytical HPLC chromatograms (Gradient C) of the crude reaction mixture (**upper panel**: signal of gamma-detector; **lower panel**: UV-signal) obtained for [123I]**44** prepared from **10a** without (**left**) and with (**right**) addition of the authentic non-radioactive reference. In the HPLC setup, the UV detector is in row before the γ-detector with Δt_R of 0.17-0.18 min between both detectors.

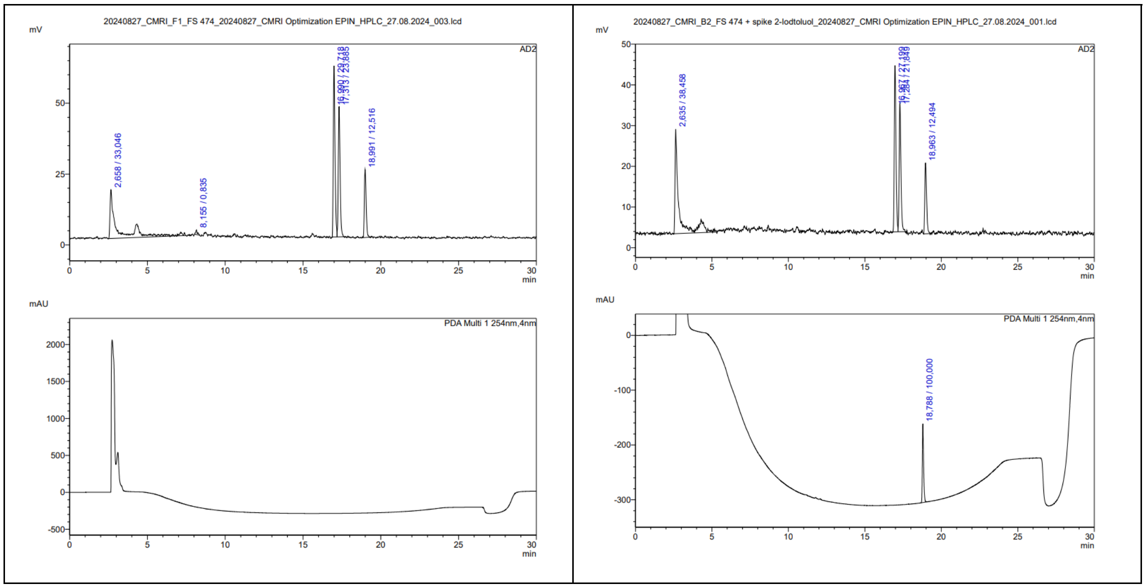

Figure S231: Copy of analytical HPLC chromatograms (Gradient C) of the crude reaction mixture (**upper panel**: signal of gamma-detector; **lower panel**: UV-signal) obtained for [^{123}I]**44** prepared from **10b** without (**left**) and with (**right**) addition of the authentic non-radioactive reference. In the HPLC setup, the UV detector is in row before the γ-detector with Δt_R of 0.17-0.18 min between both detectors.

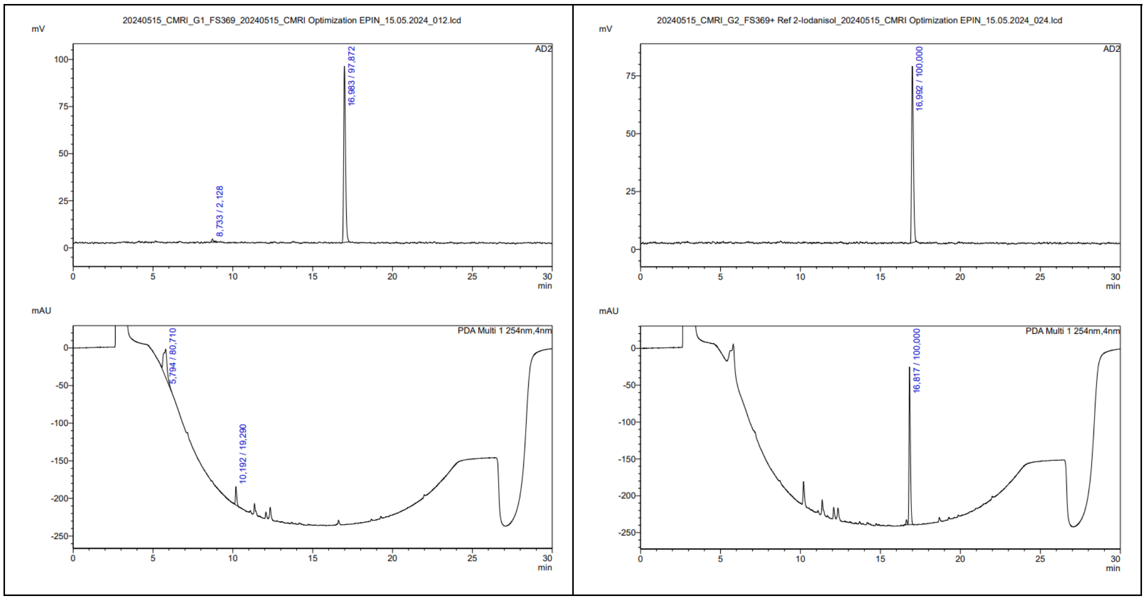

Figure S232: Copy of analytical HPLC chromatograms (Gradient C) of the crude reaction mixture (**upper panel**: signal of gamma-detector; **lower panel**: UV-signal) obtained for [^{123}I]**45** prepared from **11a** without (**left**) and with (**right**) addition of the authentic non-radioactive reference. In the HPLC setup, the UV detector is in row before the γ-detector with Δt_R of 0.17-0.18 min between both detectors.

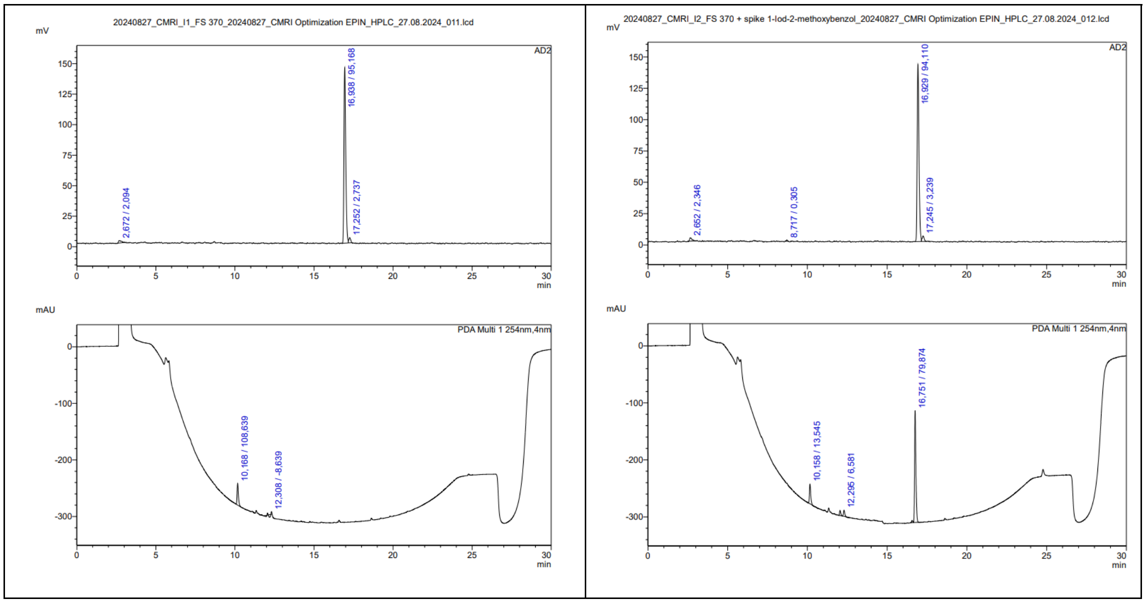

Figure S233: Copy of analytical HPLC chromatograms (Gradient C) of the crude reaction mixture (**upper panel**: signal of gamma-detector; **lower panel**: UV-signal) obtained for [^{123}I]**45** prepared from **11b** without (**left**) and with (**right**) addition of the authentic non-radioactive reference. In the HPLC setup, the UV detector is in row before the γ-detector with Δt_R of 0.17-0.18 min between both detectors.

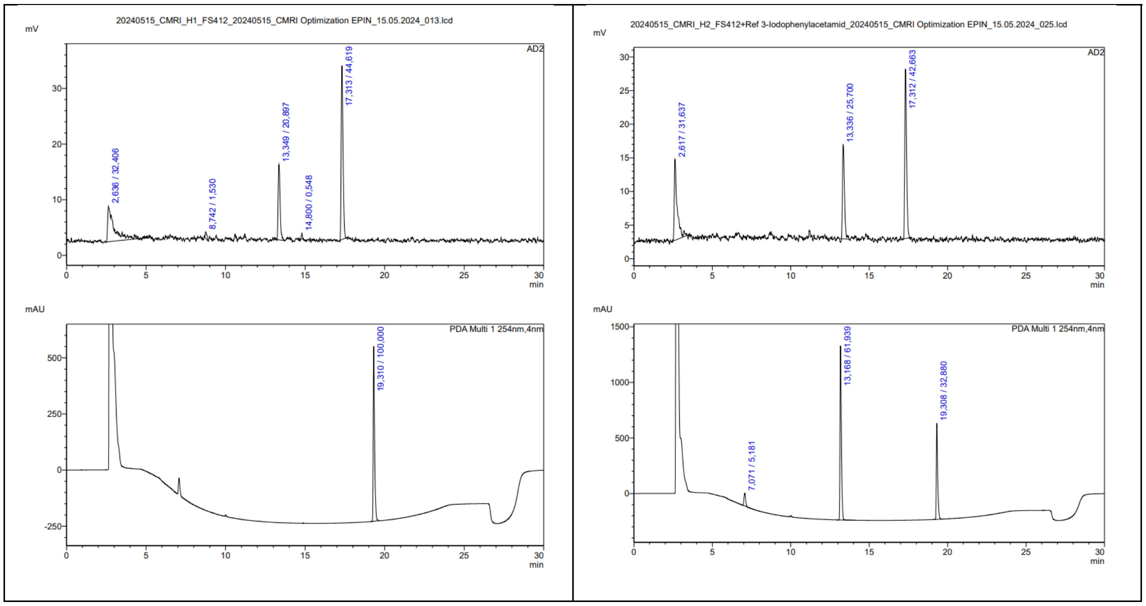

Figure S234: Copy of analytical HPLC chromatograms (Gradient C) of the crude reaction mixture (**upper panel**: signal of gamma-detector; **lower panel**: UV-signal) obtained for [^{123}I]**46** prepared from **12a** without (**left**) and with (**right**) addition of the authentic non-radioactive reference. In the HPLC setup, the UV detector is in row before the γ-detector with Δt_R of 0.17-0.18 min between both detectors.

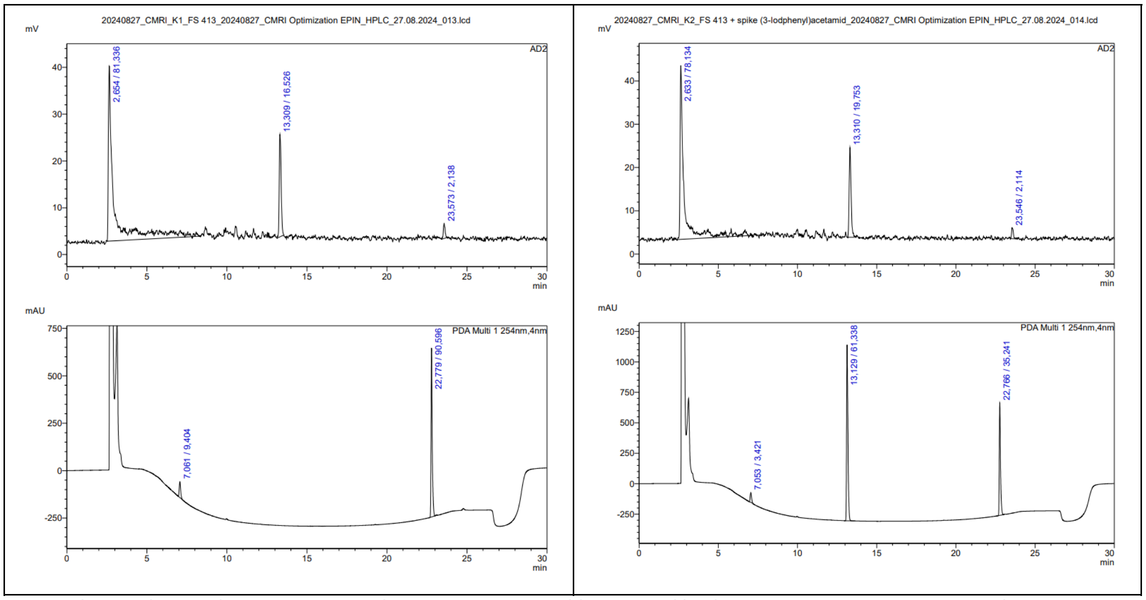

Figure S235: Copy of analytical HPLC chromatograms (Gradient C) of the crude reaction mixture (**upper panel**: signal of gamma-detector; **lower panel**: UV-signal) obtained for [^{123}I]**46** prepared from **12b** without (**left**) and with (**right**) addition of the authentic non-radioactive reference. In the HPLC setup, the UV detector is in row before the γ-detector with Δt_R of 0.17-0.18 min between both detectors.

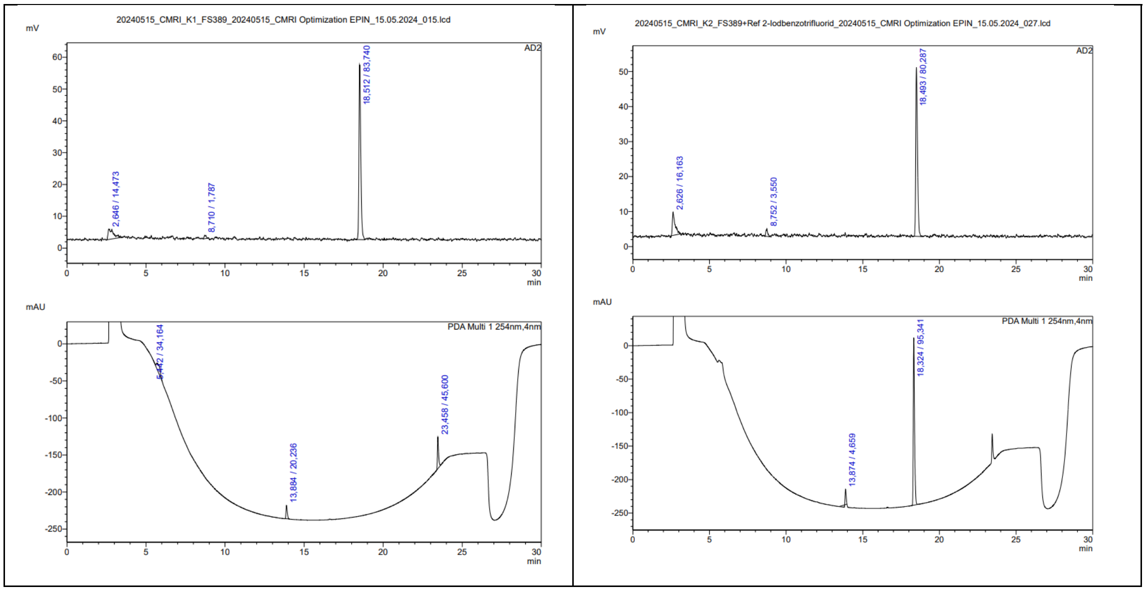

Figure S236: Copy of analytical HPLC chromatograms (Gradient C) of the crude reaction mixture (**upper panel**: signal of gamma-detector; **lower panel**: UV-signal) obtained for [^{123}I]**47** prepared from **13a** without (**left**) and with (**right**) addition of the authentic non-radioactive reference. In the HPLC setup, the UV detector is in row before the γ-detector with Δt_R of 0.17-0.18 min between both detectors.

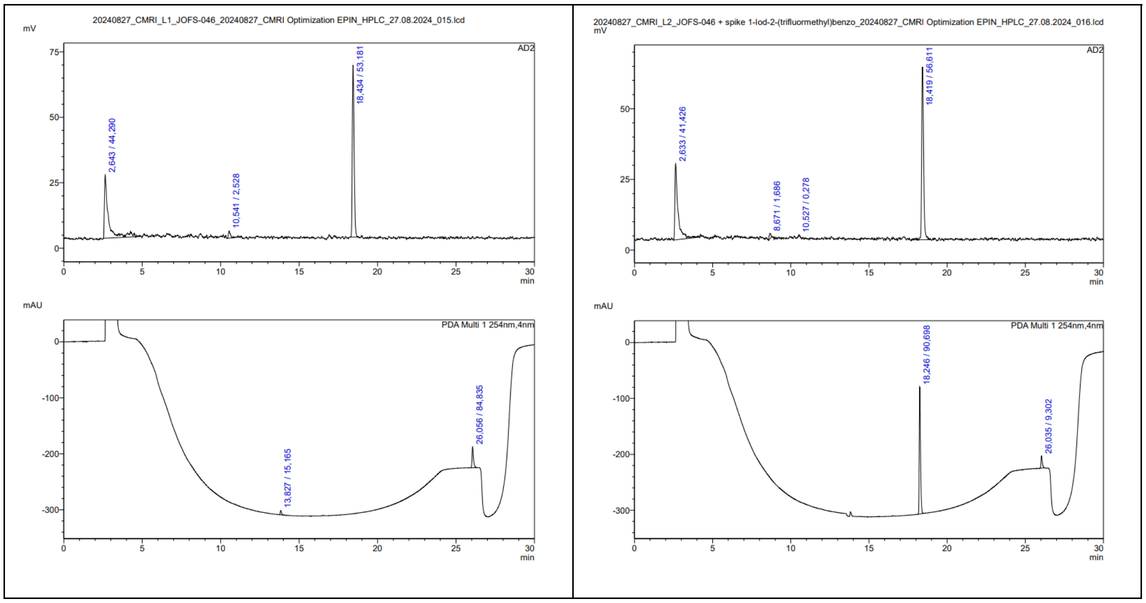

Figure S237: Copy of analytical HPLC chromatograms (Gradient C) of the crude reaction mixture (**upper panel**: signal of gamma-detector; **lower panel**: UV-signal) obtained for [^{123}I]**47** prepared from **13b** without (**left**) and with (**right**) addition of the authentic non-radioactive reference. In the HPLC setup, the UV detector is in row before the γ-detector with Δt_R of 0.17-0.18 min between both detectors.

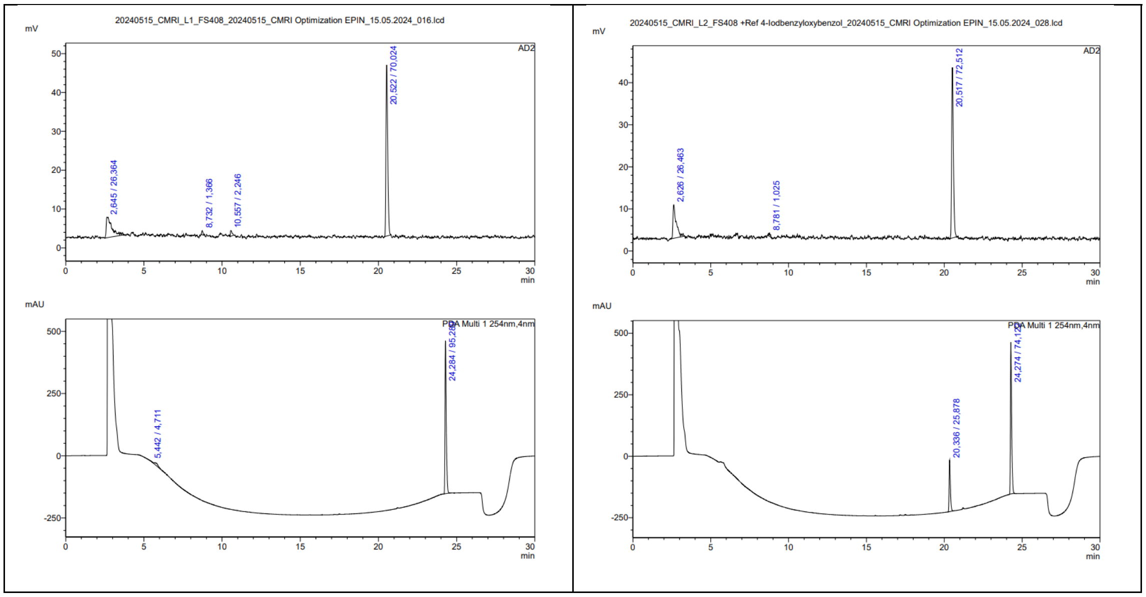

Figure S238: Copy of analytical HPLC chromatograms (Gradient C) of the crude reaction mixture (**upper panel**: signal of gamma-detector; **lower panel**: UV-signal) obtained for [^{123}I]**48** prepared from **14a** without (**left**) and with (**right**) addition of the authentic non-radioactive reference. In the HPLC setup, the UV detector is in row before the γ-detector with Δt_R of 0.17-0.18 min between both detectors.

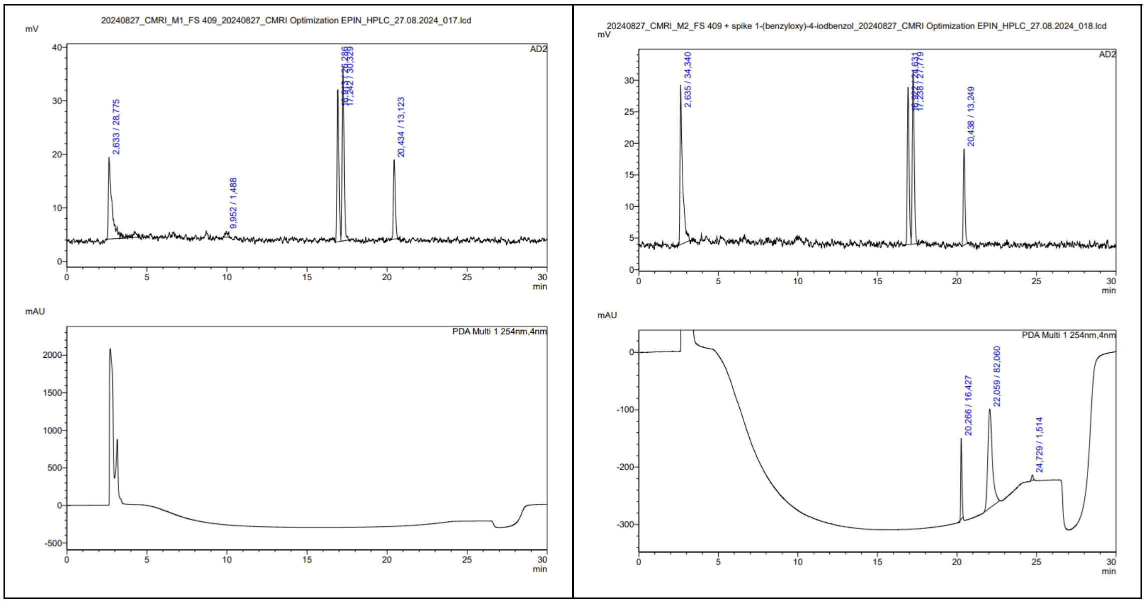

Figure S239: Copy of analytical HPLC chromatograms (Gradient C) of the crude reaction mixture (**upper panel**: signal of gamma-detector; **lower panel**: UV-signal) obtained for [^{123}I]**48** prepared from **14b** without (**left**) and with (**right**) addition of the authentic non-radioactive reference. In the HPLC setup, the UV detector is in row before the γ-detector with Δt$_R$ of 0.17-0.18 min between both detectors.

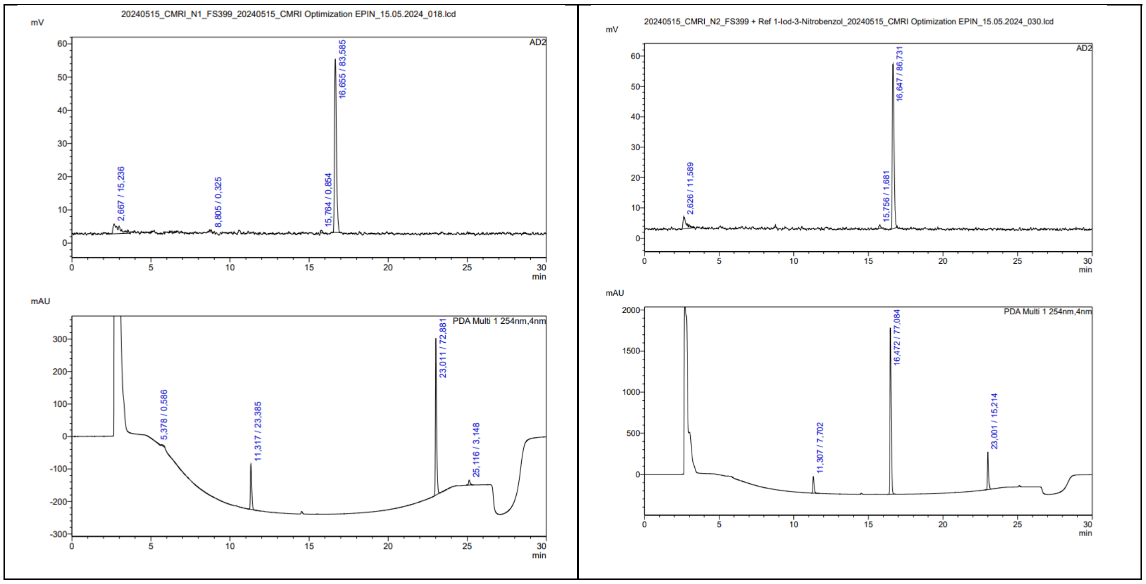

Figure S240: Copy of analytical HPLC chromatograms (Gradient C) of the crude reaction mixture (**upper panel**: signal of gamma-detector; **lower panel**: UV-signal) obtained for [^{123}I]**49** prepared from **15a** without (**left**) and with (**right**) addition of the authentic non-radioactive reference. In the HPLC setup, the UV detector is in row before the γ-detector with Δt$_R$ of 0.17-0.18 min between both detectors.

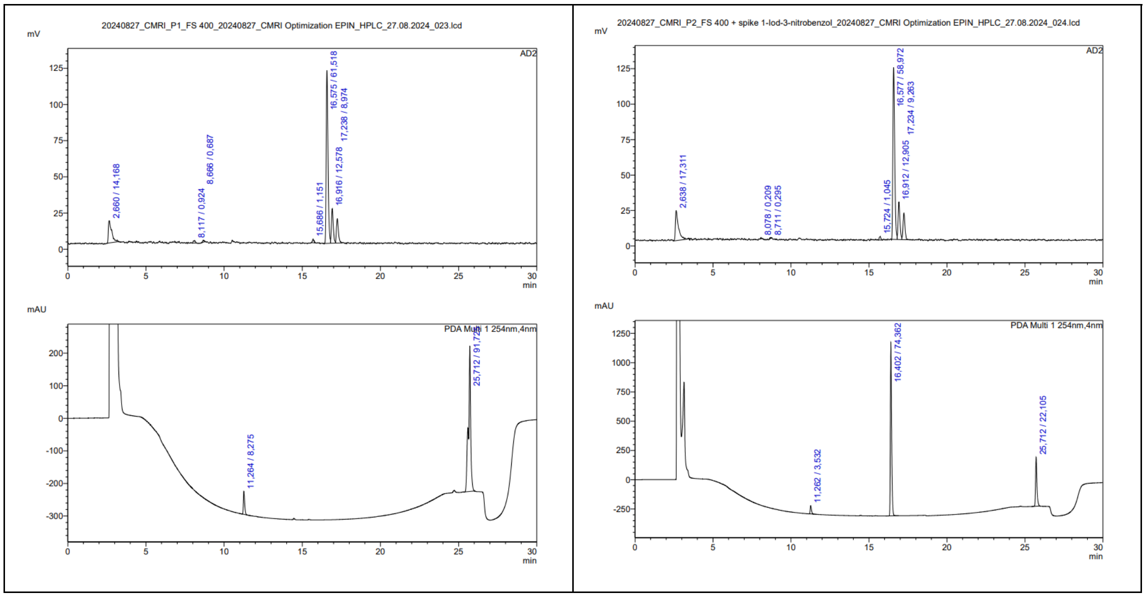

Figure S241: Copy of analytical HPLC chromatograms (Gradient C) of the crude reaction mixture (**upper panel**: signal of gamma-detector; **lower panel**: UV-signal) obtained for [^{123}I]**49** prepared from **15b** without (**left**) and with (**right**) addition of the authentic non-radioactive reference. In the HPLC setup, the UV detector is in row before the γ-detector with Δt$_R$ of 0.17-0.18 min between both detectors.

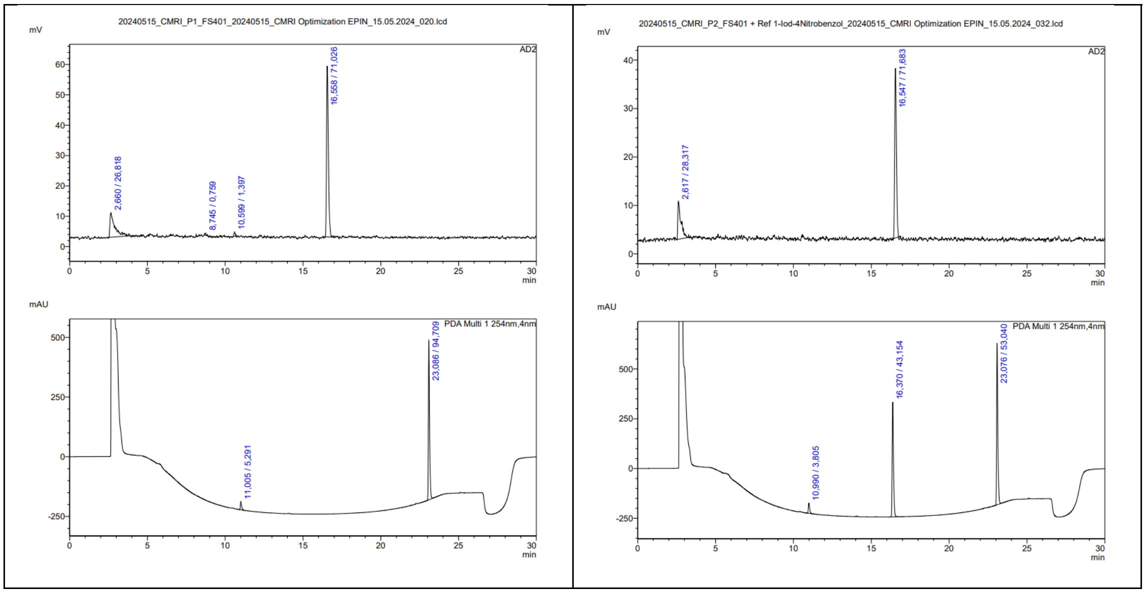

Figure S242: Copy of analytical HPLC chromatograms (Gradient C) of the crude reaction mixture (**upper panel**: signal of gamma-detector; **lower panel**: UV-signal) obtained for [^{123}I]**50** prepared from **16a** without (**left**) and with (**right**) addition of the authentic non-radioactive reference. In the HPLC setup, the UV detector is in row before the γ-detector with Δt$_R$ of 0.17-0.18 min between both detectors.

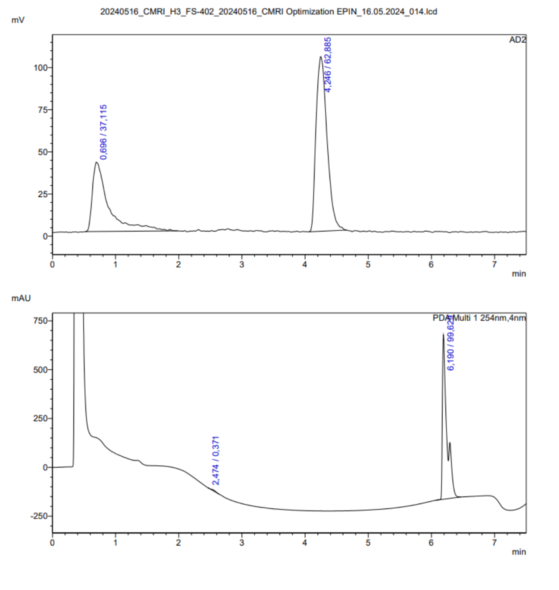

Not determined

Figure S243: Copy of analytical (U)HPLC chromatograms (Gradient A) of the crude reaction mixture (**upper panel**: signal of gamma-detector; **lower panel**: UV-signal) obtained for [^{123}I]**50** prepared from **16b** without addition of the authentic non-radioactive reference. In the HPLC setup, the UV detector is in row before the γ-detector with Δt_R of 0.3 min between both detectors.

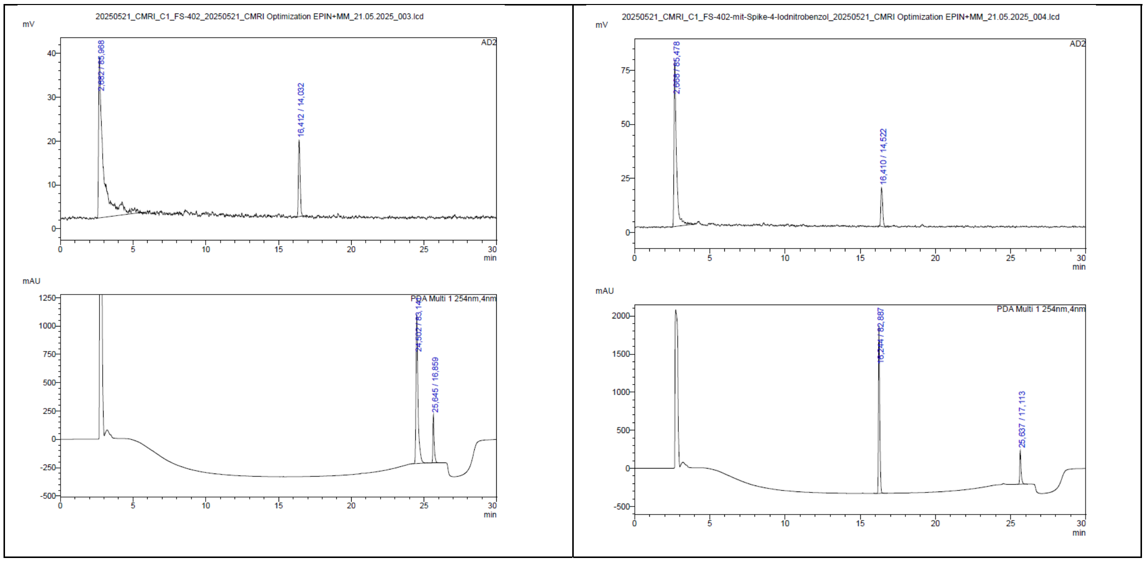

Figure S244: Copy of analytical HPLC chromatograms (Gradient C) of the crude reaction mixture (**upper panel**: signal of gamma-detector; **lower panel**: UV-signal) obtained for [^{123}I]**50** prepared from **16b** without (**left**) and with

(**right**) addition of the authentic non-radioactive reference. In the HPLC setup, the UV detector is in row before the γ-detector with Δt_R of 0.17-0.18 min between both detectors.

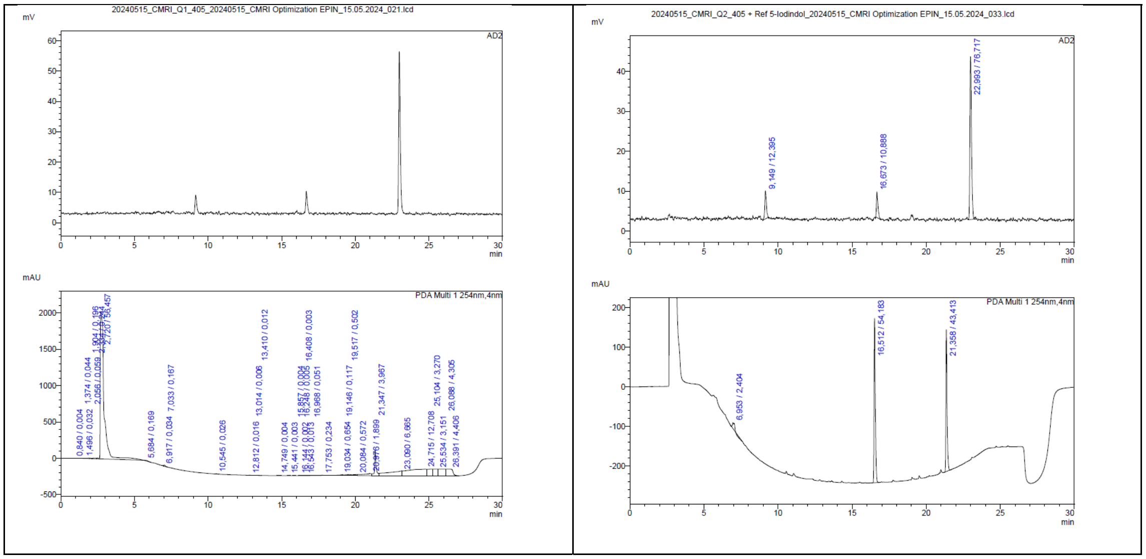

Figure S245: Copy of analytical HPLC chromatograms (Gradient C) of the crude reaction mixture (**upper panel**: signal of gamma-detector; **lower panel**: UV-signal) obtained for [^{123}I]**51** prepared from **17a** without (**left**) and with (**right**) addition of the authentic non-radioactive reference. In the HPLC setup, the UV detector is in row before the γ-detector with Δt_R of 0.17-0.18 min between both detectors.

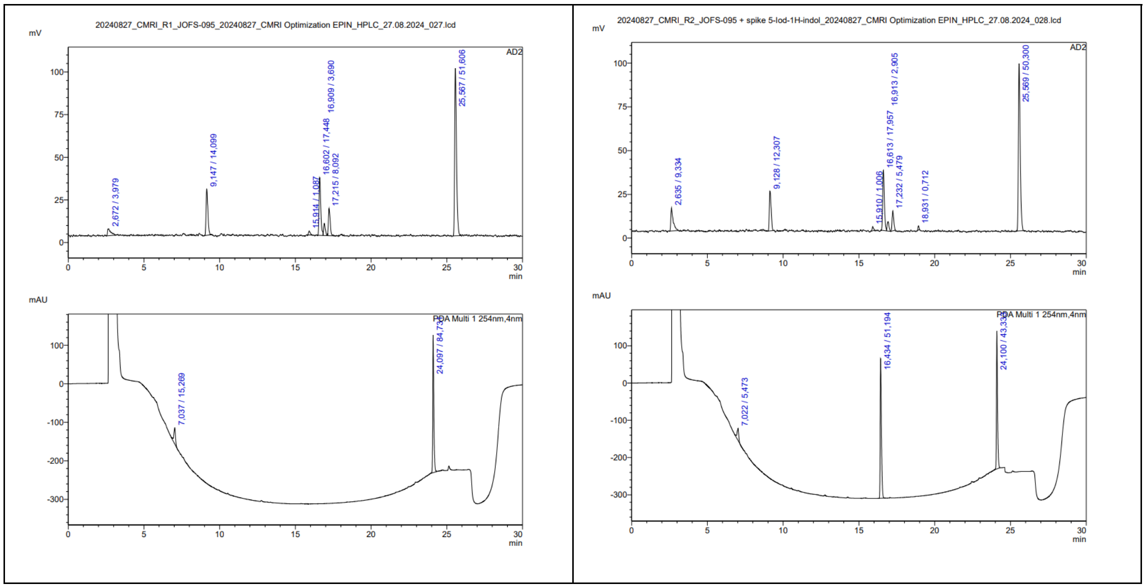

Figure S246: Copy of analytical HPLC chromatograms (Gradient C) of the crude reaction mixture (**upper panel**: signal of gamma-detector; **lower panel**: UV-signal) obtained for [^{123}I]**51** prepared from **17b** without (**left**) and with (**right**) addition of the authentic non-radioactive reference. In the HPLC setup, the UV detector is in row before the γ-detector with Δt_R of 0.17-0.18 min between both detectors.

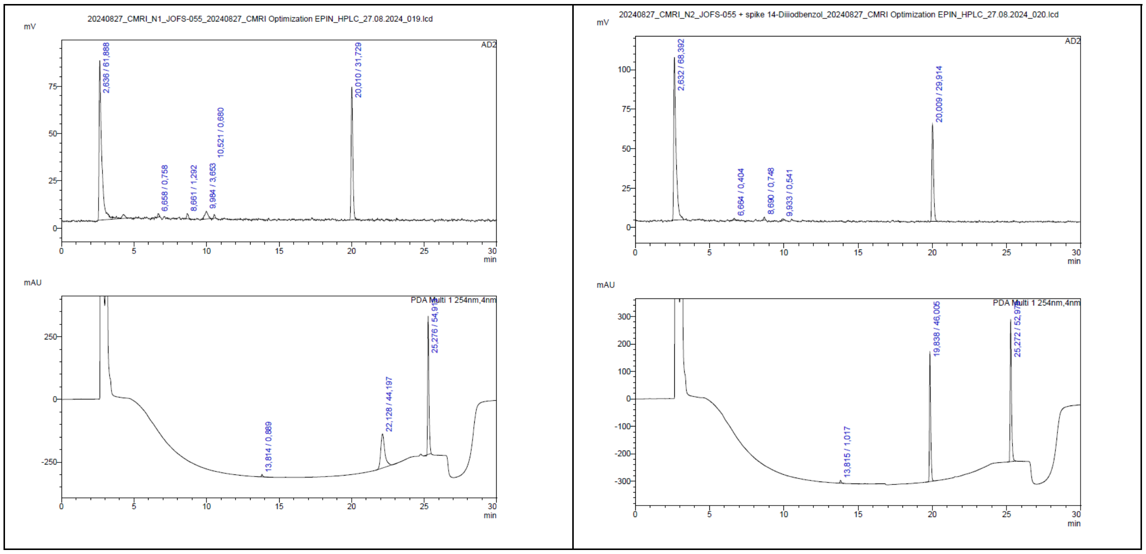

Figure S247: Copy of analytical HPLC chromatograms (Gradient C) of the crude reaction mixture (**upper panel**: signal of gamma-detector; **lower panel**: UV-signal) obtained for [^{123}I]**52** prepared from **18a** without (**left**) and with (**right**) addition of the authentic non-radioactive reference. In the HPLC setup, the UV detector is in row before the γ-detector with Δt_R of 0.17-0.18 min between both detectors.

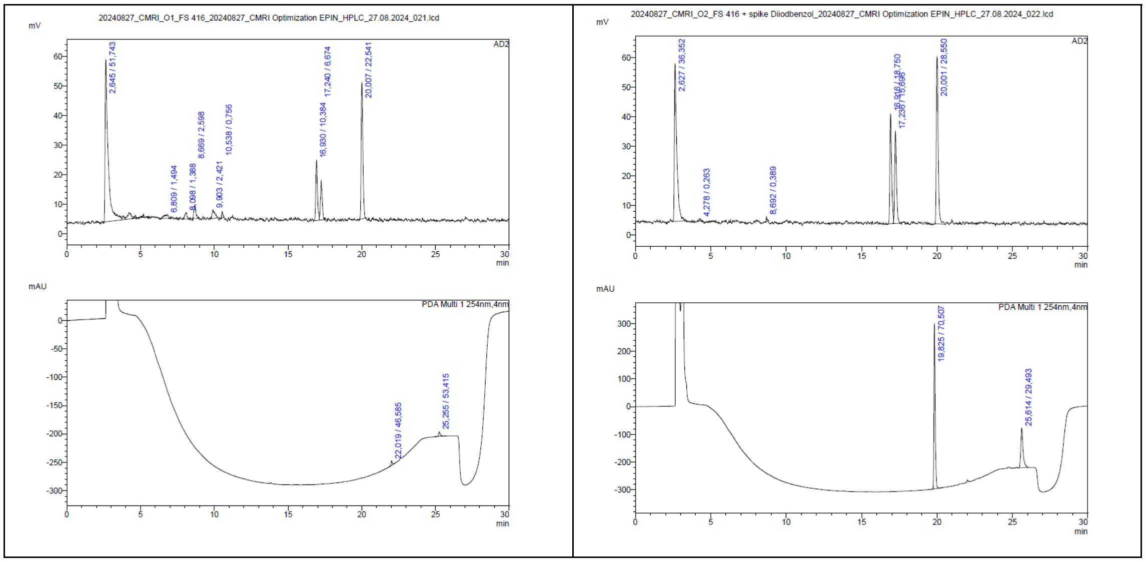

Figure S248: Copy of analytical HPLC chromatograms (Gradient C) of the crude reaction mixture (**upper panel**: signal of gamma-detector; **lower panel**: UV-signal) obtained for [^{123}I]**52** prepared from **18b** without (**left**) and with (**right**) addition of the authentic non-radioactive reference. In the HPLC setup, the UV detector is in row before the γ-detector with Δt_R of 0.17-0.18 min between both detectors.

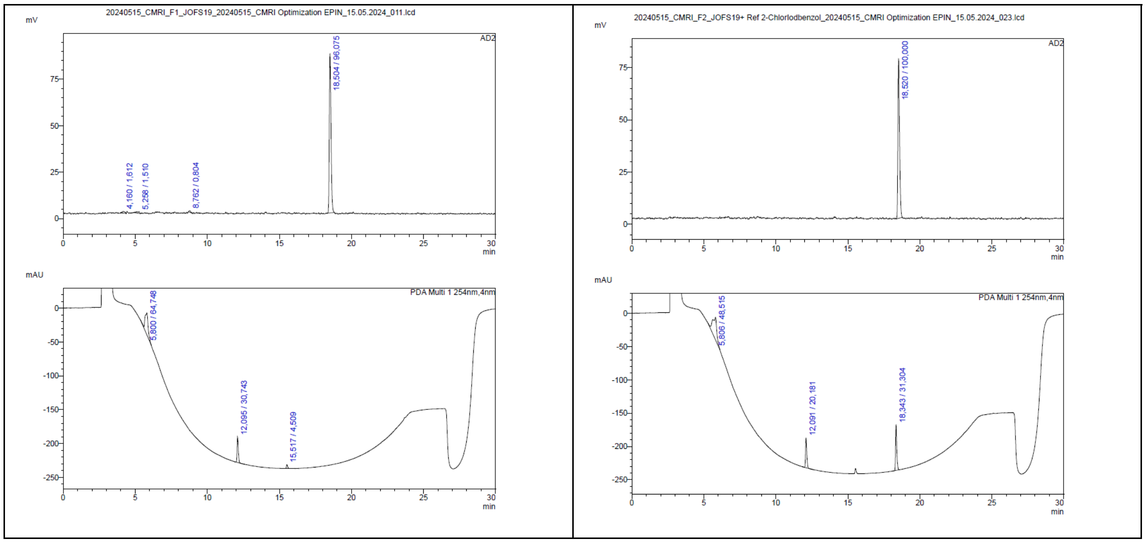

Figure S249: Copy of analytical HPLC chromatograms (Gradient C) of the crude reaction mixture (**upper panel**: signal of gamma-detector; **lower panel**: UV-signal) obtained for [^{123}I]**53** prepared from **19a** without (**left**) and with (**right**) addition of the authentic non-radioactive reference. In the HPLC setup, the UV detector is in row before the γ-detector with Δt_R of 0.17-0.18 min between both detectors.

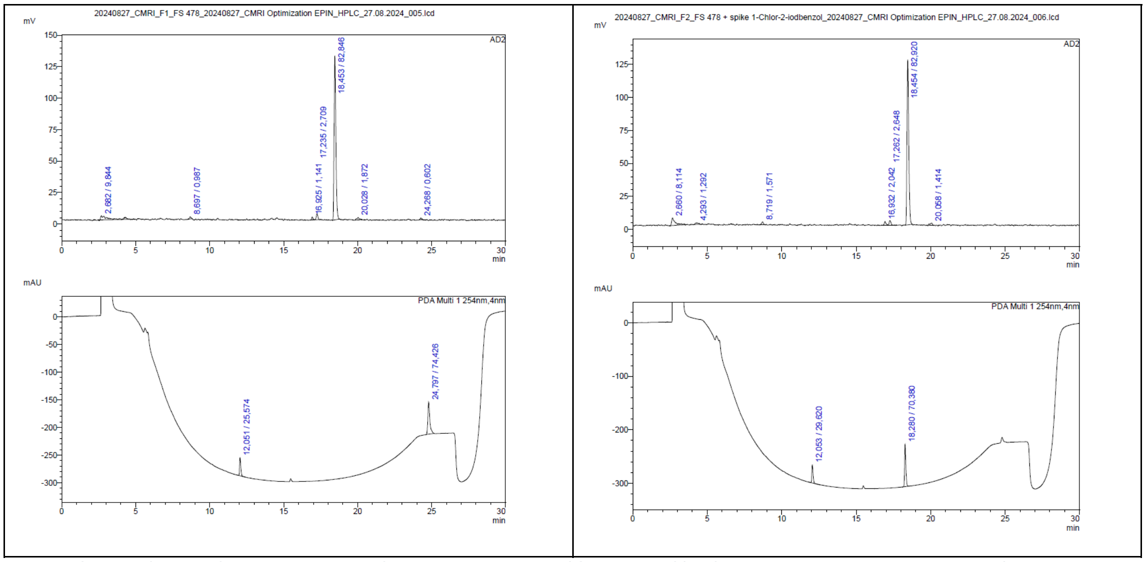

Figure S250: Copy of analytical HPLC chromatograms (Gradient C) of the crude reaction mixture (**upper panel**: signal of gamma-detector; **lower panel**: UV-signal) obtained for [^{123}I]**53** prepared from **19b** without (**left**) and with (**right**) addition of the authentic non-radioactive reference. In the HPLC setup, the UV detector is in row before the γ-detector with Δt$_R$ of 0.17-0.18 min between both detectors.

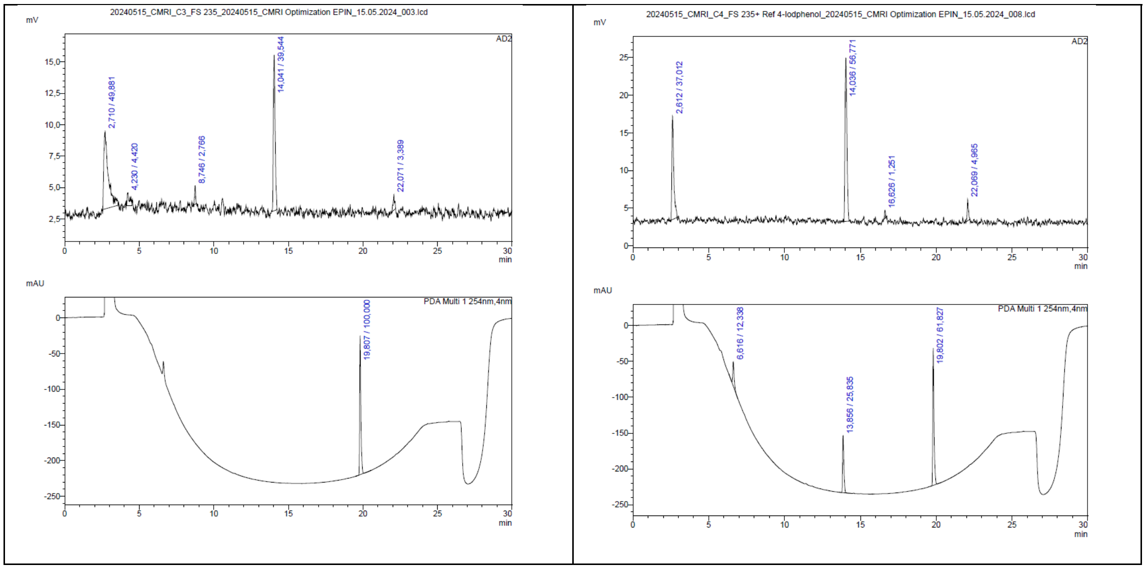

Figure S251: Copy of analytical HPLC chromatograms (Gradient C) of the crude reaction mixture (**upper panel**: signal of gamma-detector; **lower panel**: UV-signal) obtained for [^{123}I]**54** prepared from **20a** without (**left**) and with (**right**) addition of the authentic non-radioactive reference. In the HPLC setup, the UV detector is in row before the γ-detector with Δt$_R$ of 0.17-0.18 min between both detectors.

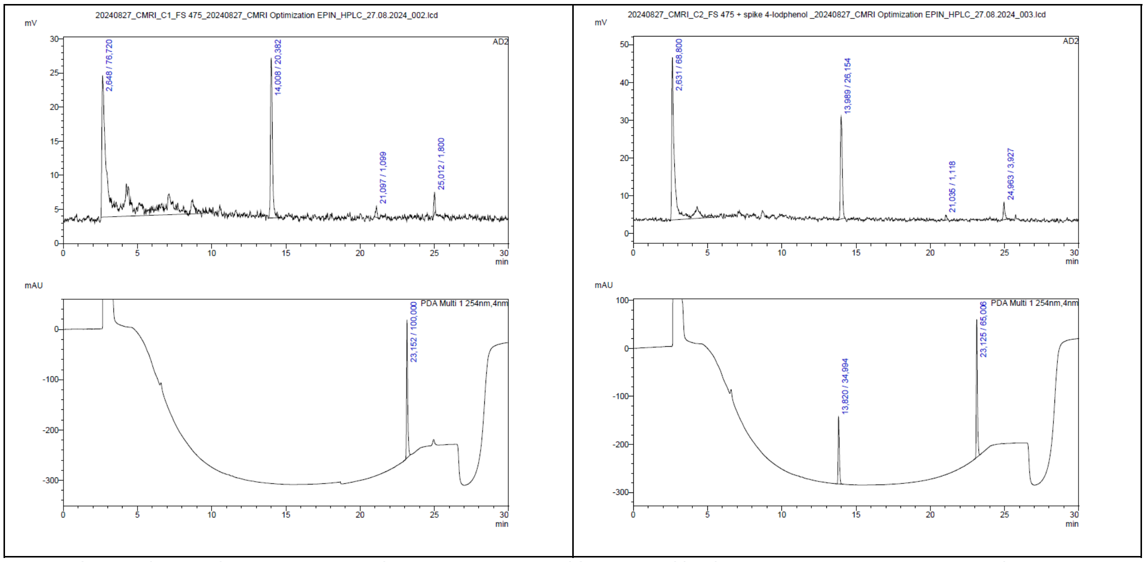

Figure S252: Copy of analytical HPLC chromatograms (Gradient C) of the crude reaction mixture (**upper panel**: signal of gamma-detector; **lower panel**: UV-signal) obtained for [^{123}I]**54** prepared from **20b** without (**left**) and with (**right**) addition of the authentic non-radioactive reference. In the HPLC setup, the UV detector is in row before the γ-detector with Δt_R of 0.17-0.18 min between both detectors.

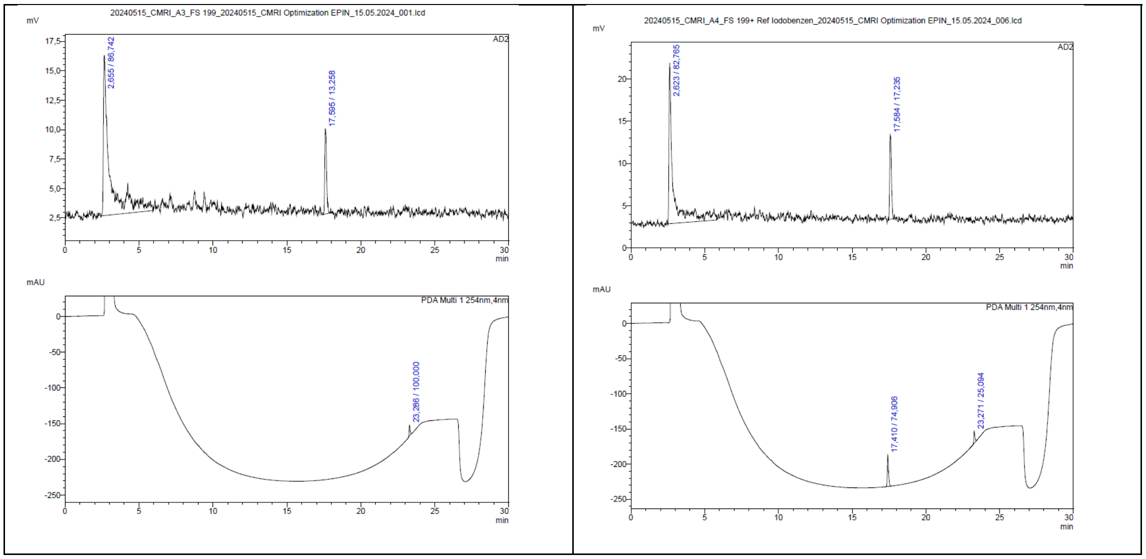

Figure S253: Copy of analytical HPLC chromatograms (Gradient C) of the crude reaction mixture (**upper panel**: signal of gamma-detector; **lower panel**: UV-signal) obtained for [^{123}I]**55** prepared from **21a** without (**left**) and with (**right**) addition of the authentic non-radioactive reference. In the HPLC setup, the UV detector is in row before the γ-detector with Δt_R of 0.17-0.18 min between both detectors.

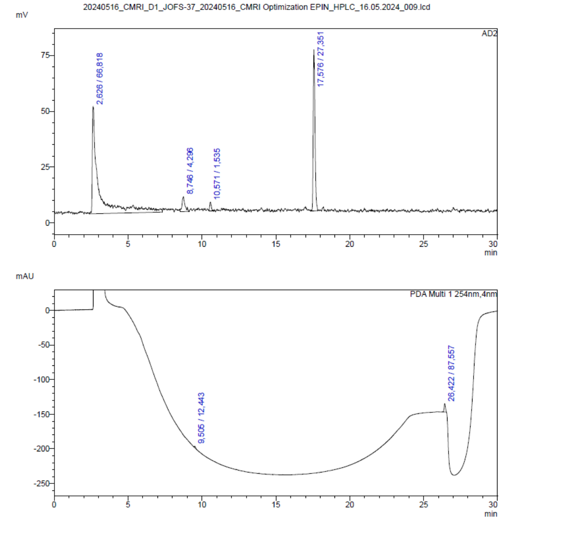

Not determined

Figure S254: Copy of analytical HPLC chromatograms (Gradient C) of the crude reaction mixture (**upper panel**: signal of gamma-detector; **lower panel**: UV-signal) obtained for [^{123}I]**55** prepared from **21b** without addition of the authentic non-radioactive reference. In the HPLC setup, the UV detector is in row before the γ-detector with Δt_R of 0.17-0.18 min between both detectors.

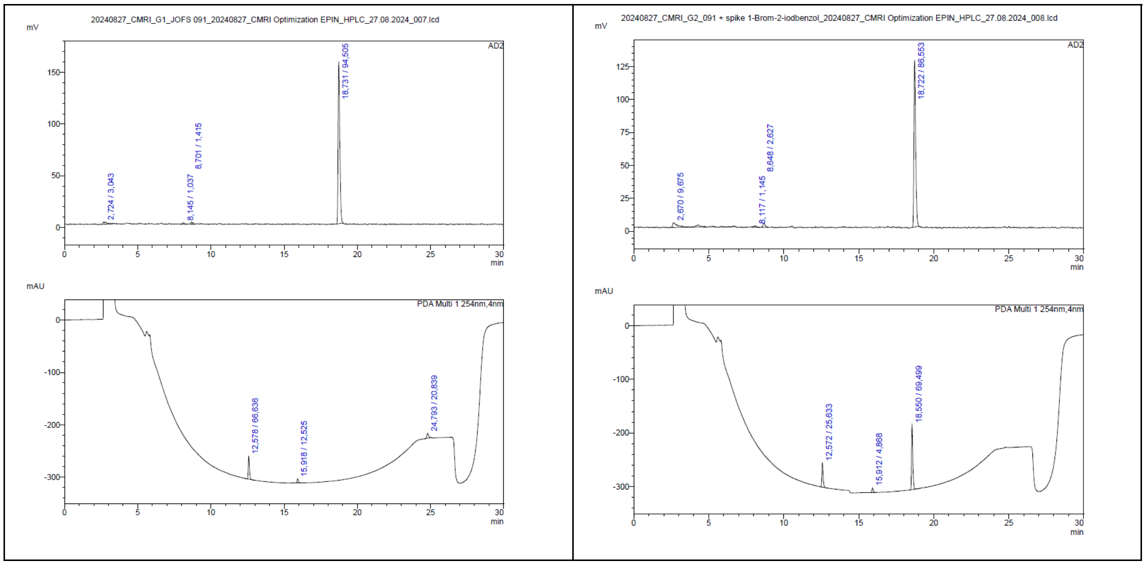

Figure S255: Copy of analytical HPLC chromatograms (Gradient C) of the crude reaction mixture (**upper panel**: signal of gamma-detector; **lower panel**: UV-signal) obtained for [^{123}I]**56** prepared from **22a** without (**left**) and with (**right**) addition of the authentic non-radioactive reference. In the HPLC setup, the UV detector is in row before the γ-detector with Δt_R of 0.17-0.18 min between both detectors.

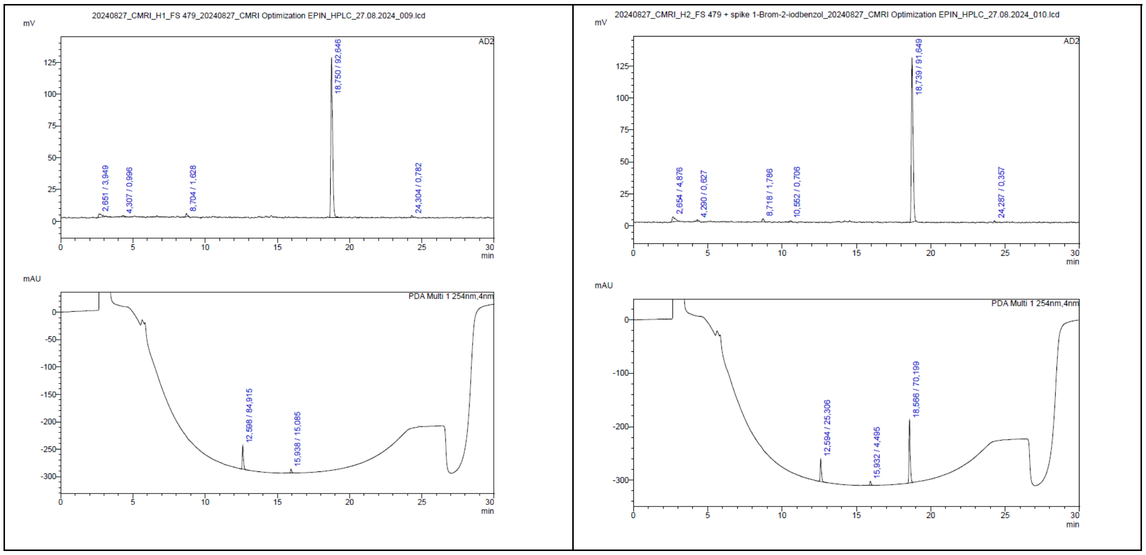

Figure S256: Copy of analytical HPLC chromatograms (Gradient C) of the crude reaction mixture (**upper panel**: signal of gamma-detector; **lower panel**: UV-signal) obtained for [^{123}I]**56** prepared from **22b** without (**left**) and with (**right**) addition of the authentic non-radioactive reference. In the HPLC setup, the UV detector is in row before the γ-detector with Δt$_R$ of 0.17-0.18 min between both detectors.

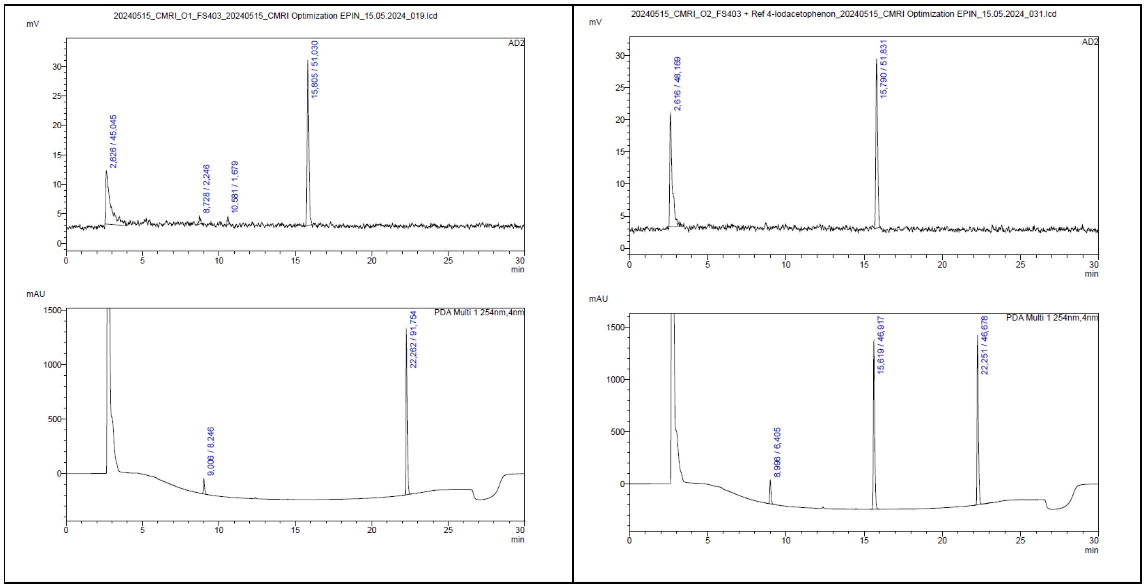

Figure S257: Copy of analytical HPLC chromatograms (Gradient C) of the crude reaction mixture (**upper panel**: signal of gamma-detector; **lower panel**: UV-signal) obtained for [^{123}I]**57** prepared from **23a** without (**left**) and with (**right**) addition of the authentic non-radioactive reference. In the HPLC setup, the UV detector is in row before the γ-detector with Δt$_R$ of 0.17-0.18 min between both detectors.

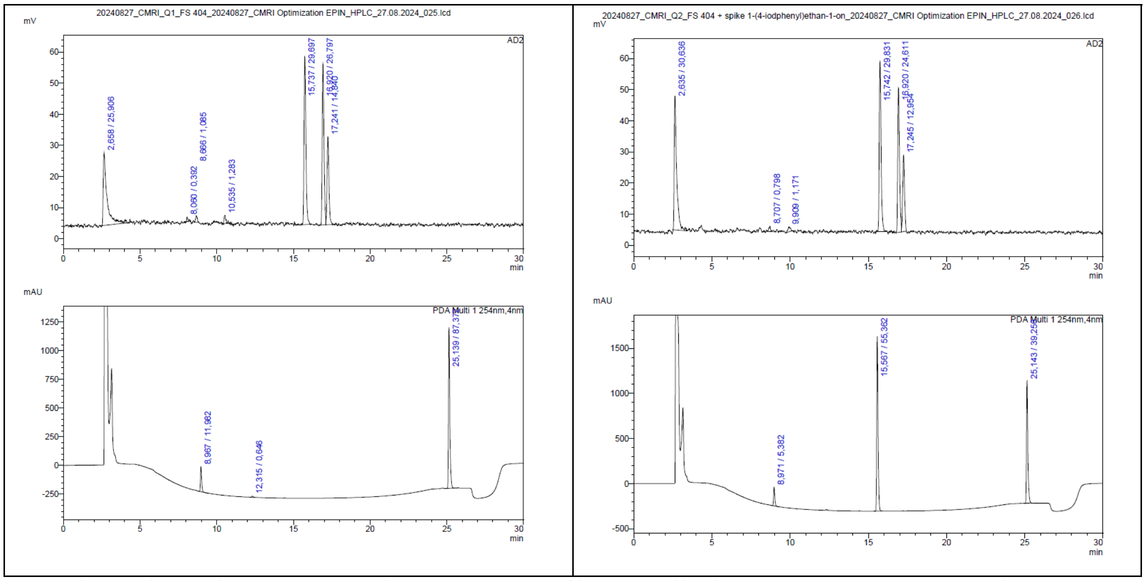

Figure S258: Copy of analytical HPLC chromatograms (Gradient C) of the crude reaction mixture (**upper panel**: signal of gamma-detector; **lower panel**: UV-signal) obtained for [^{123}I]**57** prepared from **23a** without (**left**) and with (**right**) addition of the authentic non-radioactive reference. In the HPLC setup, the UV detector is in row before the γ-detector with Δt_R of 0.17-0.18 min between both detectors.

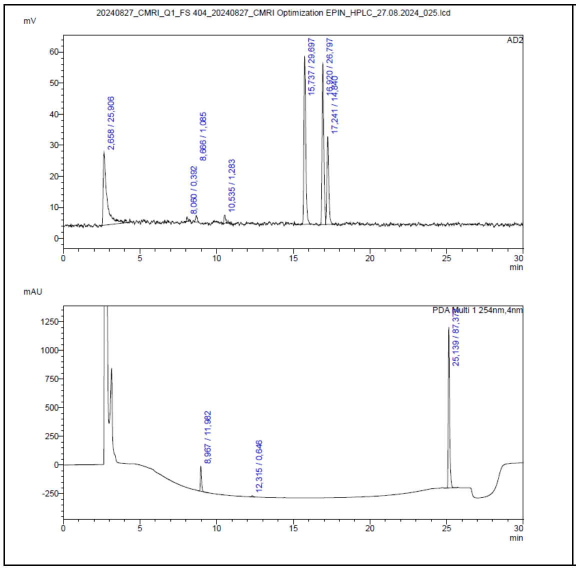

Not determined

Figure S259: Copy of analytical HPLC chromatograms (Gradient C) of the crude reaction mixture (**upper panel**: signal of gamma-detector; **lower panel**: UV-signal) obtained for [^{123}I]**35** prepared from **36** without addition of the authentic non-radioactive reference. In the HPLC setup, the UV detector is in row before the γ-detector with Δt_R of 0.17-0.18 min between both detectors.

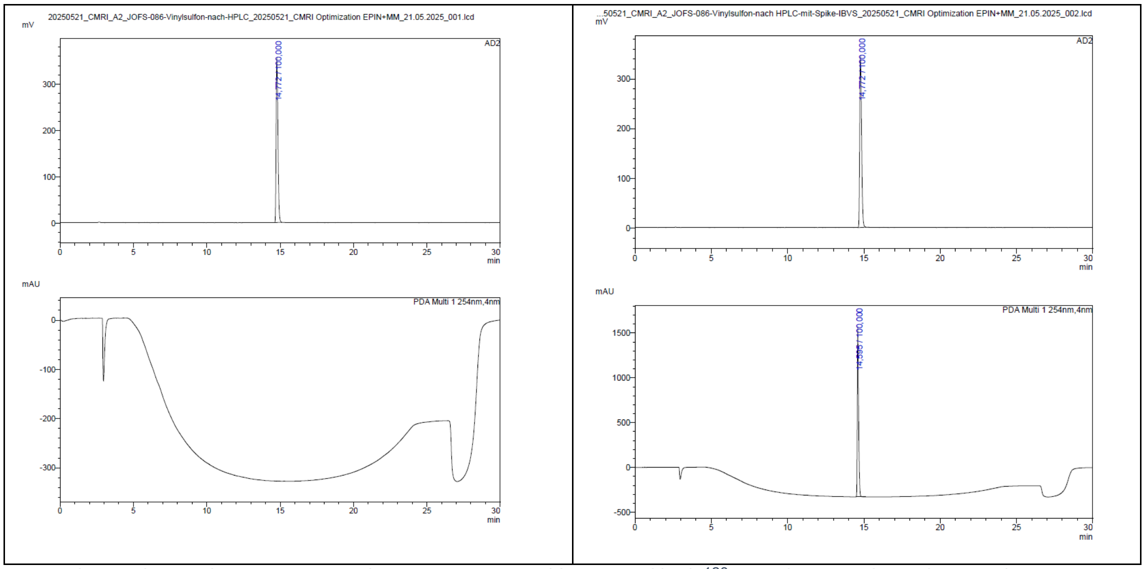

Figure S260: Copy of analytical HPLC chromatograms (Gradient C) of [^{123}I]**35** after HPLC purification (**upper panel**: signal of gamma-detector; **lower panel**: UV-signal) prepared from **36** without (**left**) and with (**right**) addition of the authentic non-radioactive reference. In the HPLC setup, the UV detector is in row before the γ-detector with Δt_R of 0.17-0.18 min between both detectors.

HPLC & UHPLC Chromatograms of ^{18}F-Labeled Compounds

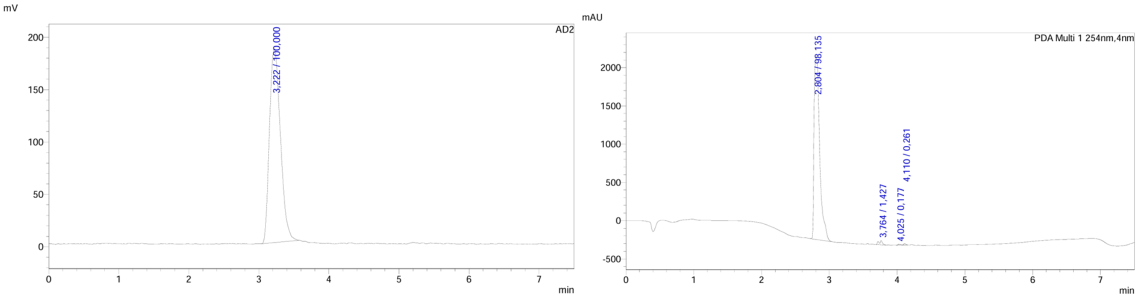

Figure S261: Radio-UHPLC chromatogram of [^{18}F]**33** prepared from **36** (**left**); (separate run) UV UHPLC chromatogram of reference compound **33** (**right**) (Gradient A).

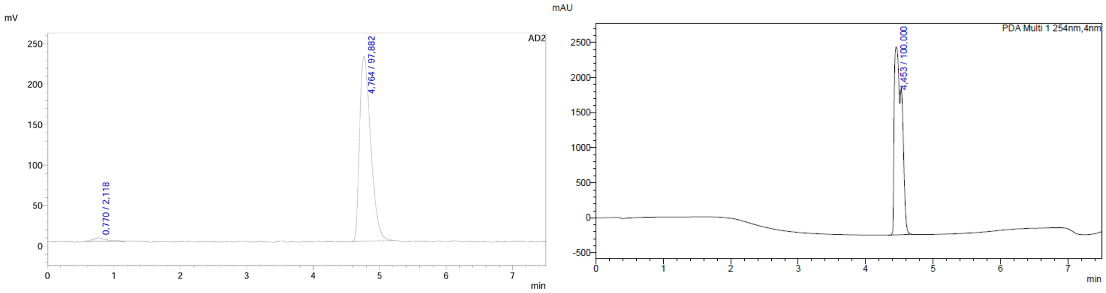

Figure S262: Radio-UHPLC chromatogram of [^{18}F]**58** prepared from **5a** (**left**); (separate run) UV UHPLC chromatogram of reference compound **58** (**right**) (Gradient A).

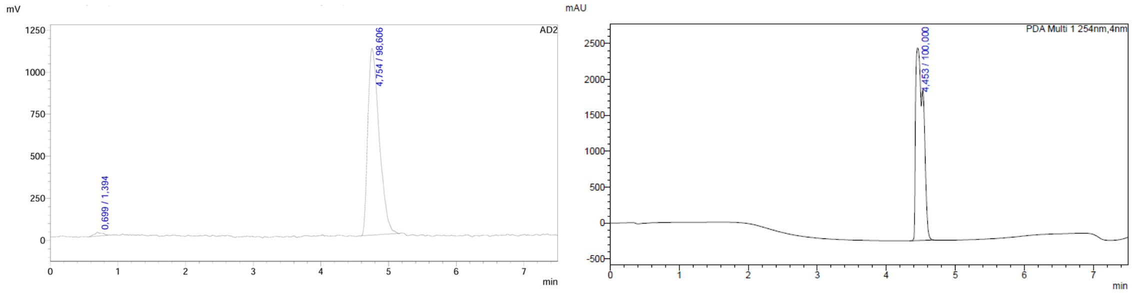

Figure S263: Radio-UHPLC chromatogram of [^{18}F]**58** prepared from **5b** (**left**); (separate run) UV UHPLC chromatogram of reference compound **58** (**right**) (Gradient A).

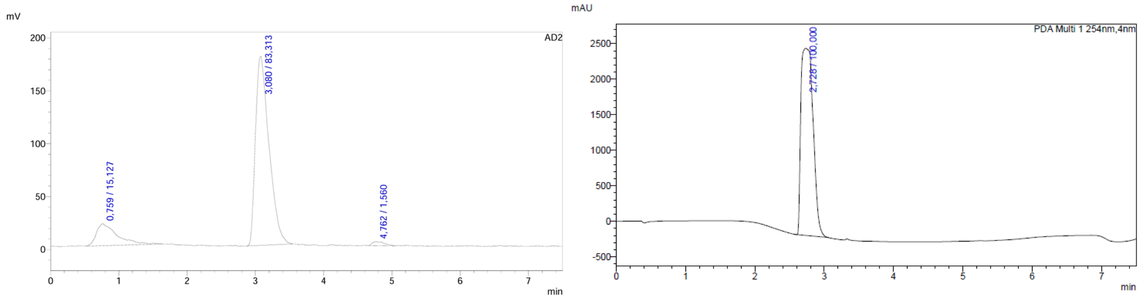

Figure S264: Radio-UHPLC chromatogram of [^{18}F]**59** prepared from **6a** (**left**); (separate run) UV UHPLC chromatogram of reference compound **59** (**right**) (Gradient A).

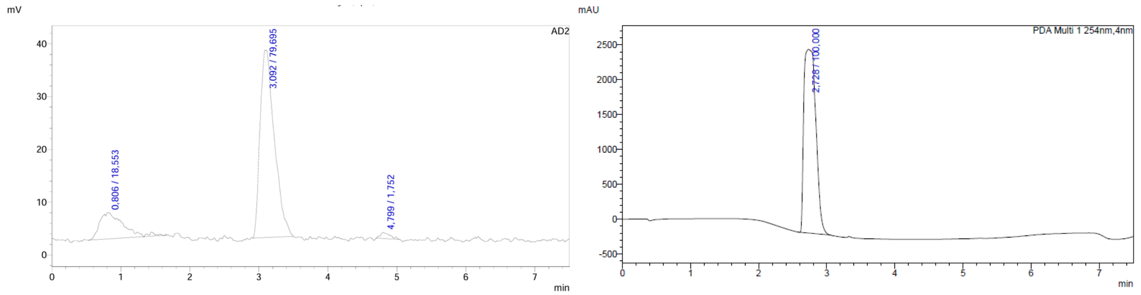

Figure S265: Radio-UHPLC chromatogram of [^{18}F]**59** prepared from **6b** (**left**); (separate run) UV UHPLC chromatogram of reference compound **59** (**right**) (Gradient A).

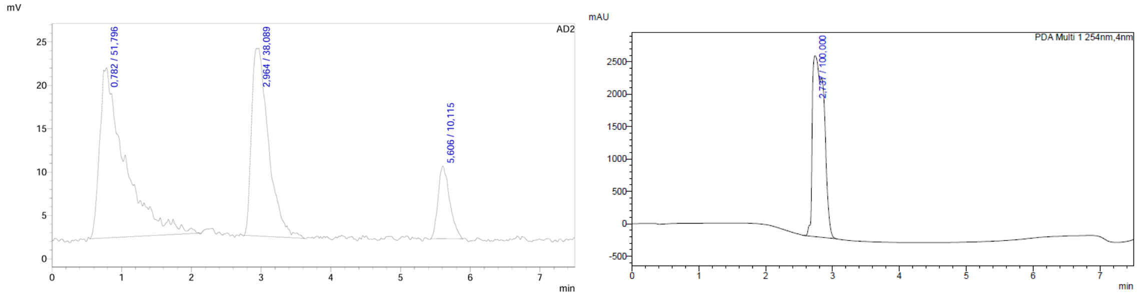

Figure S266: Radio-UHPLC chromatogram of [^{18}F]**60** prepared from **7a** (**left**); (separate run) UV UHPLC chromatogram of reference compound **60** (**right**) (Gradient A).

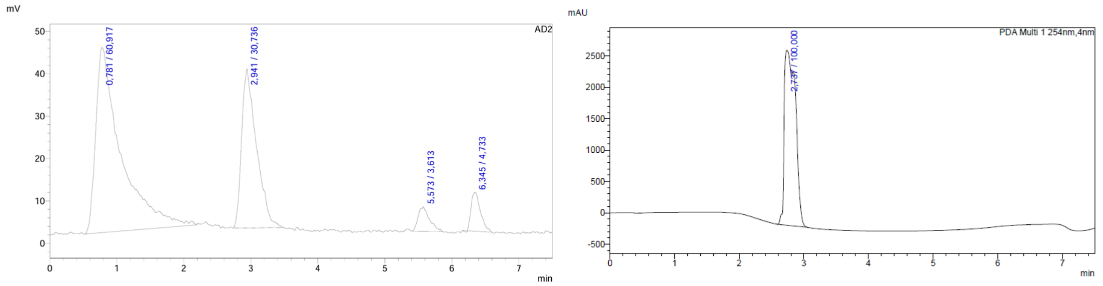

Figure S267: Radio-UHPLC chromatogram of [18F]**60** prepared from **7b** (**left**); (separate run) UV UHPLC chromatogram of reference compound **60** (**right**) (Gradient A).

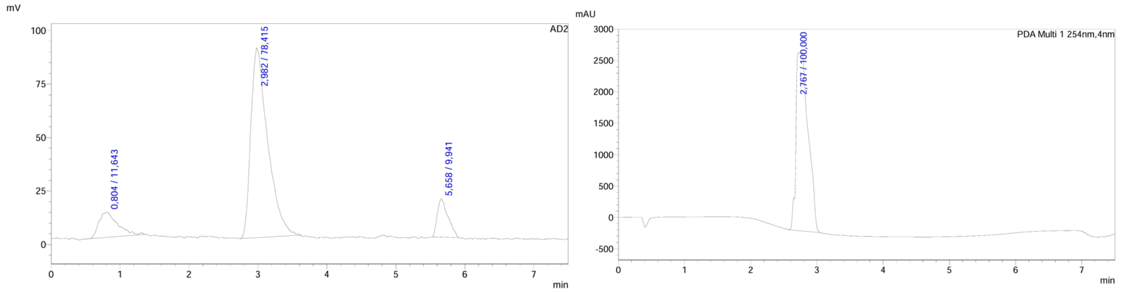

Figure S268: Radio-UHPLC chromatogram of [18F]**61** prepared from **8a** (**left**); (separate run) UV UHPLC chromatogram of reference compound **61** (**right**) (Gradient A).

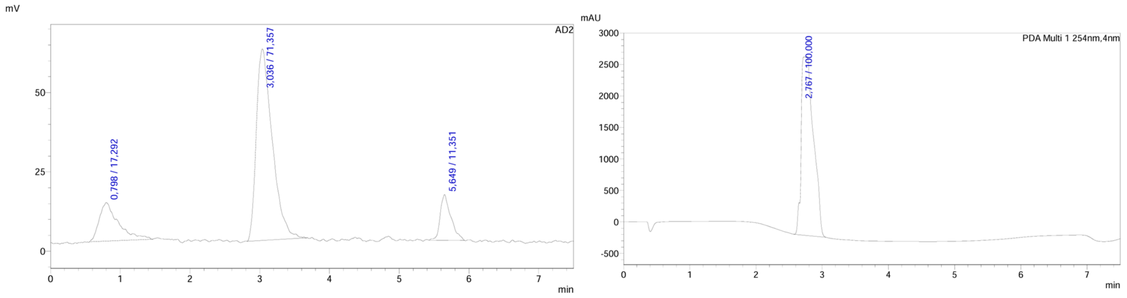

Figure S269: Radio-UHPLC chromatogram of [18F]**61** prepared from **8b** (**left**); (separate run) UV UHPLC chromatogram of reference compound **61** (**right**) (Gradient A).

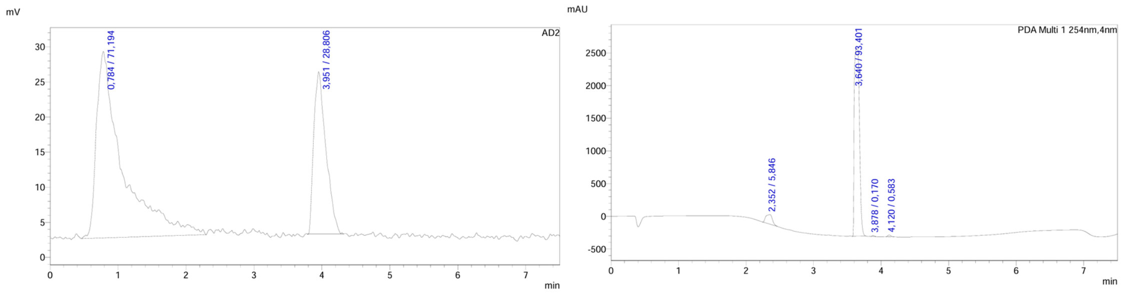

Figure S 270

Figure S265: Radio-UHPLC chromatogram of [18F]**62** prepared from **9a** (**left**); (separate run) UV UHPLC chromatogram of reference compound **62** (**right**) (Gradient A).

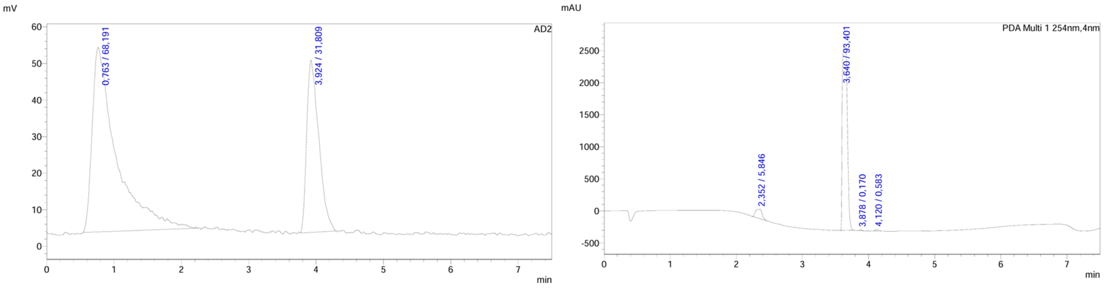

Figure S271: Radio-UHPLC chromatogram of [18F]**62** prepared from **9b** (**left**); (separate run) UV UHPLC chromatogram of reference compound **62** (**right**) (Gradient A).

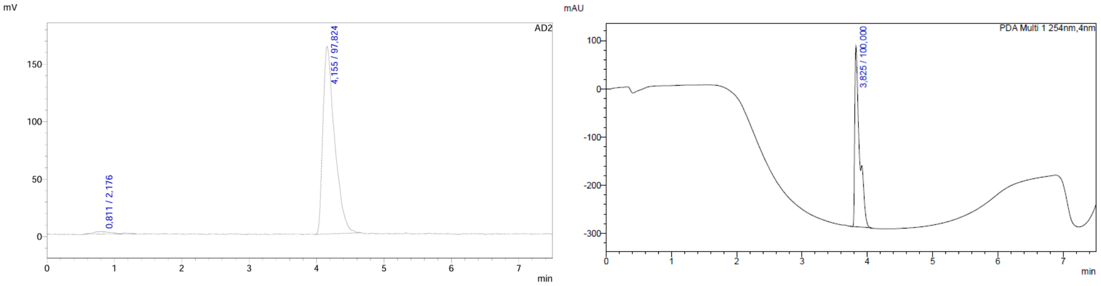

Figure S272: Radio-UHPLC chromatogram of [18F]**63** prepared from **10a** (**left**); (separate run) UV UHPLC chromatogram of reference compound **63** (**right**) (Gradient A).

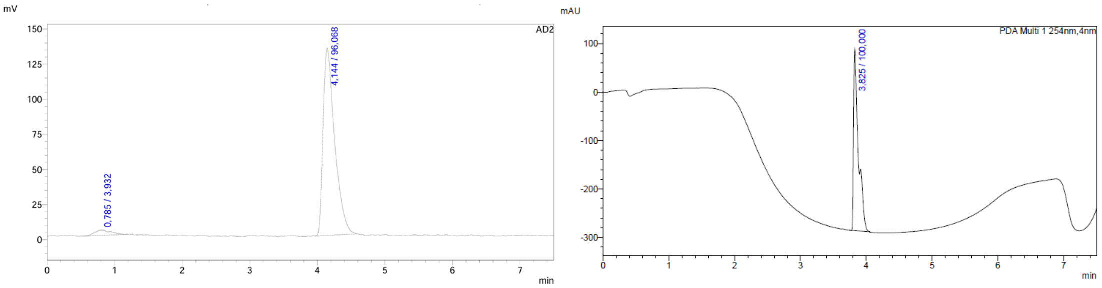

Figure S273: Radio-UHPLC chromatogram of [18F]**63** prepared from **10b** (**left**); (separate run) UV UHPLC chromatogram of reference compound **63** (**right**) (Gradient A).

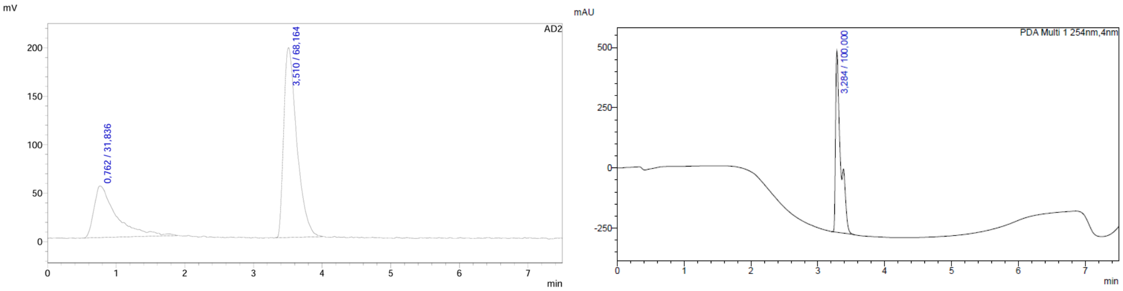

Figure S274: Radio-UHPLC chromatogram of [18F]**64** prepared from **11a** (**left**); (separate run) UV UHPLC chromatogram of reference compound **64** (**right**) (Gradient A).

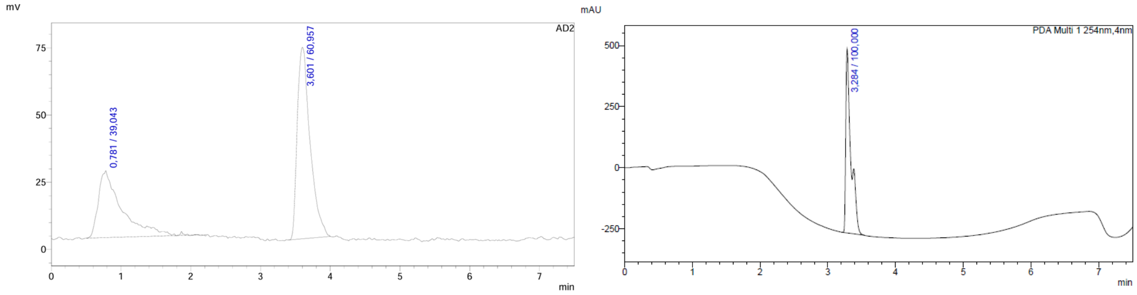

Figure S275: Radio-UHPLC chromatogram of [^{18}F]**64** prepared from **11b** (**left**); (separate run) UV UHPLC chromatogram of reference compound **64** (**right**) (Gradient A).

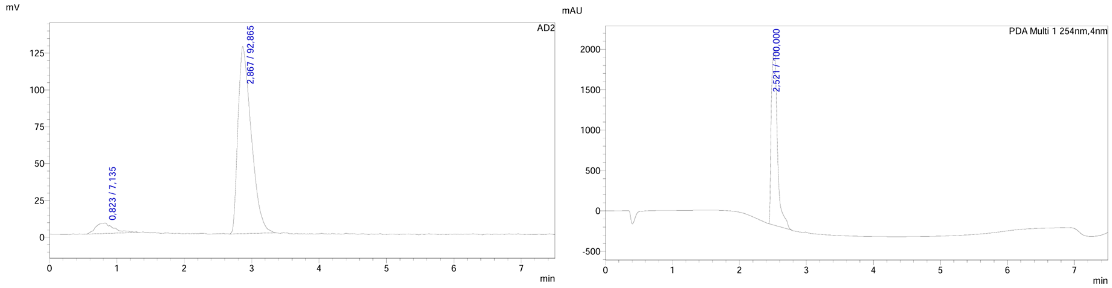

Figure S276: Radio-UHPLC chromatogram of [^{18}F]**65** prepared from **12a** (**left**); (separate run) UV UHPLC chromatogram of reference compound **65** (**right**) (Gradient A).

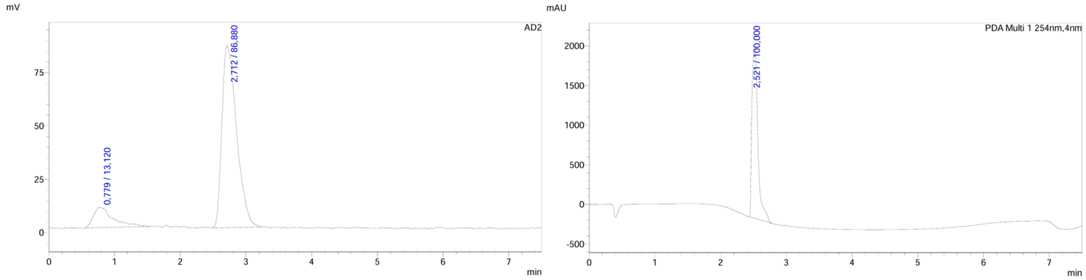

Figure S277: Radio-UHPLC chromatogram of [^{18}F]**65** prepared from **12b** (**left**); (separate run) UV UHPLC chromatogram of reference compound **65** (**right**) (Gradient A).

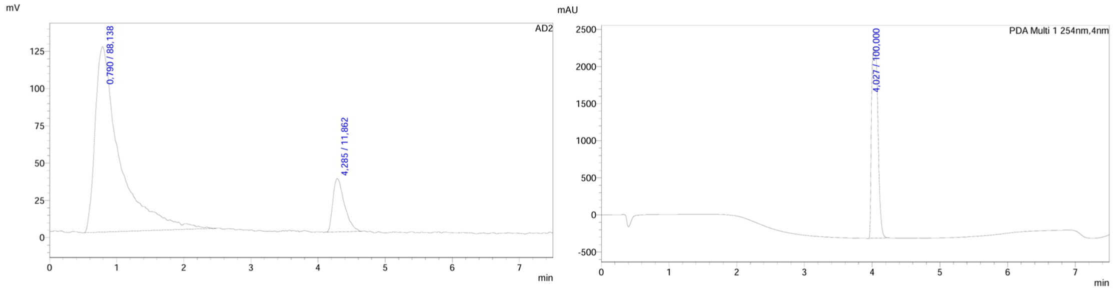

Figure S278: Radio-UHPLC chromatogram of [^{18}F]**66** prepared from **13a** (**left**); (separate run) UV UHPLC chromatogram of reference compound **66** (**right**) (Gradient A).

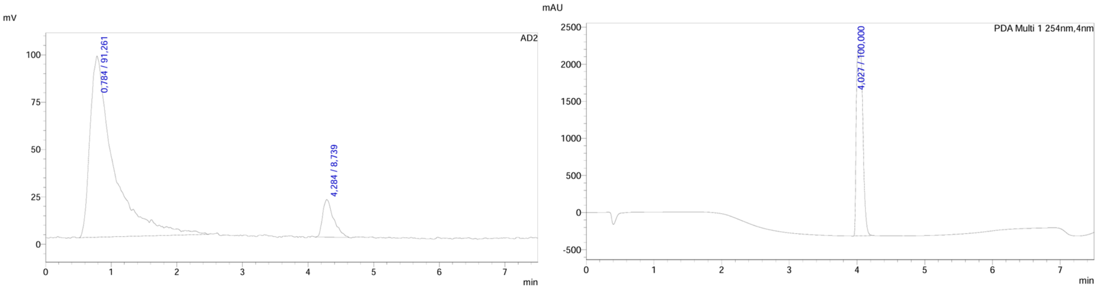

Figure S279: Radio-UHPLC chromatogram of [^{18}F]**66** prepared from **13b** (**left**); (separate run) UV UHPLC chromatogram of reference compound **66** (**right**) (Gradient A).

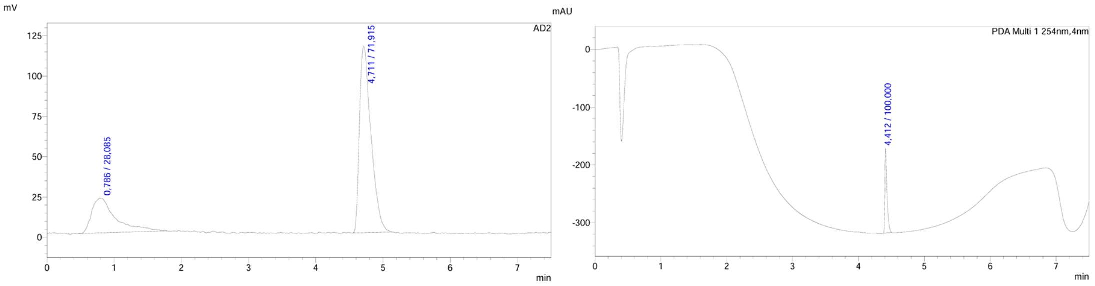

Figure S280: Radio-UHPLC chromatogram of [^{18}F]**67** prepared from **14a** (**left**); (separate run) UV UHPLC chromatogram of reference compound **67** (**right**) (Gradient A).

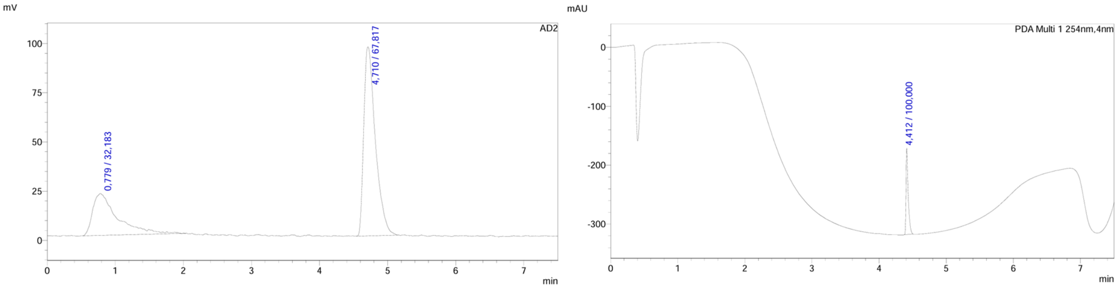

Figure S281: Radio-UHPLC chromatogram of [^{18}F]**67** prepared from **14b** (**left**); (separate run) UV UHPLC chromatogram of reference compound **67** (**right**) (Gradient A).

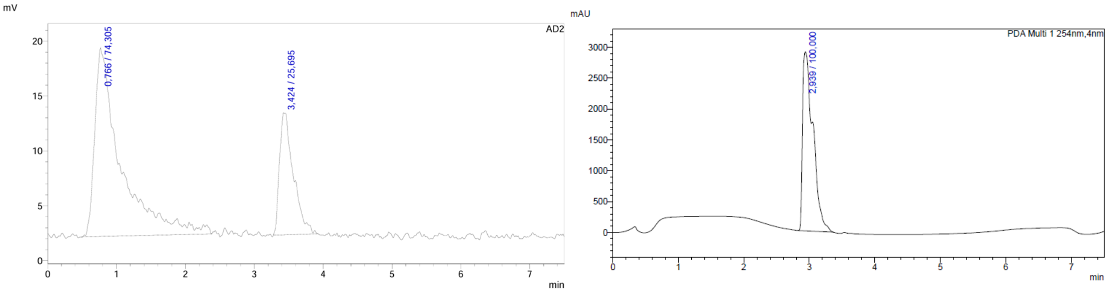

Figure S282: Radio-UHPLC chromatogram of [^{18}F]**68** prepared from **15a** (**left**); (separate run) UV UHPLC chromatogram of reference compound **68** (**right**) (Gradient A).

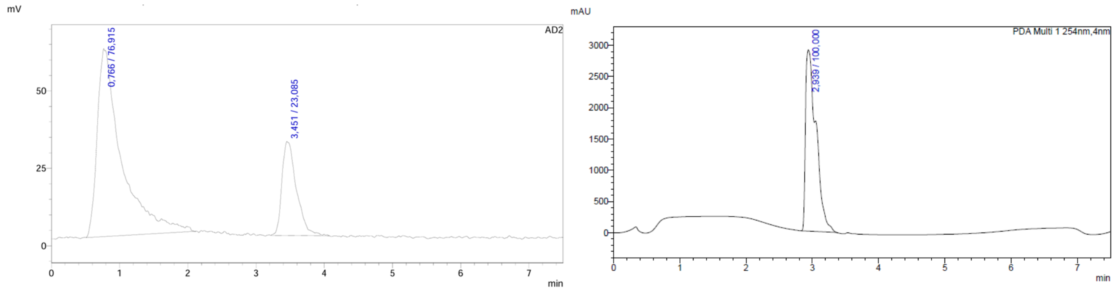

Figure S283: Radio-UHPLC chromatogram of [^{18}F]**68** prepared from **15b** (**left**); (separate run) UV UHPLC chromatogram of reference compound **68** (**right**) (Gradient A).

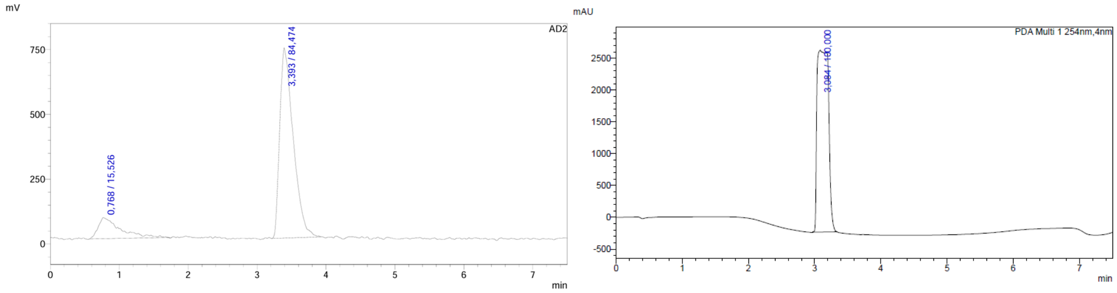

Figure S284: Radio-UHPLC chromatogram of [^{18}F]**69** prepared from **16a** (**left**); (separate run) UV UHPLC chromatogram of reference compound **69** (**right**) (Gradient A).

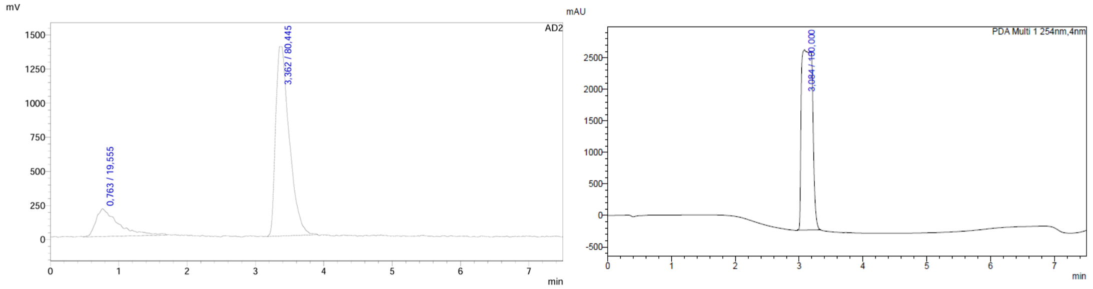

Figure S285: Radio-UHPLC chromatogram of [^{18}F]**67** prepared from **16b** (**left**); (separate run) UV UHPLC chromatogram of reference compound **67** (**right**) (Gradient A).

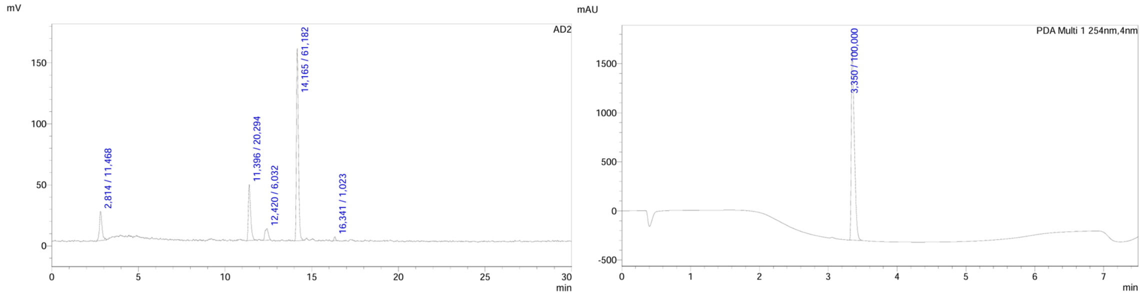

Figure S286: Radio-UHPLC chromatogram of [^{18}F]**70** prepared from **17a** (**left**); (separate run) UV UHPLC chromatogram of reference compound **70** (**right**) (Gradient C).

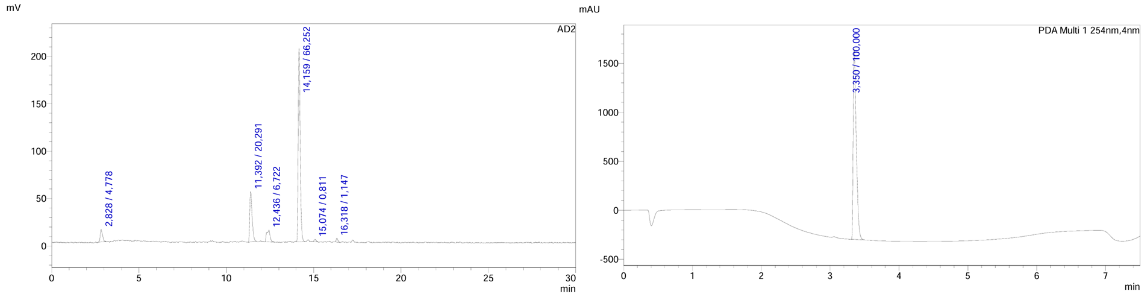

Figure S287: Radio-UHPLC chromatogram of [^{18}F]**70** prepared from **17b** (**left**); (separate run) UV UHPLC chromatogram of reference compound **70** (**right**) (Gradient C).

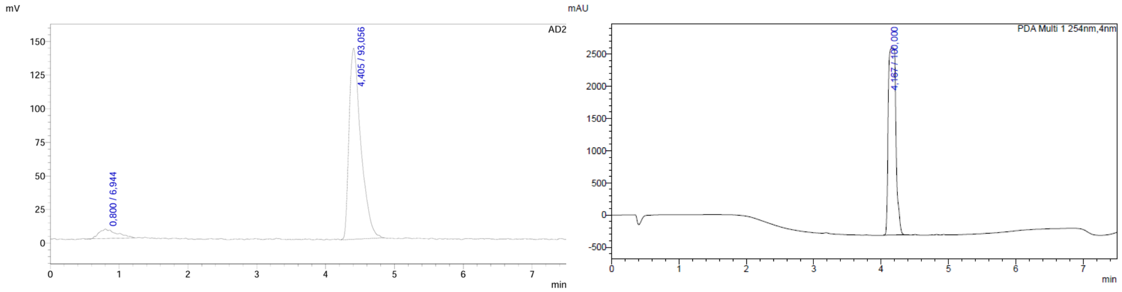

Figure S288: Radio-UHPLC chromatogram of [^{18}F]**71** prepared from **18a** (**left**); (separate run) UV UHPLC chromatogram of reference compound **71** (**right**) (Gradient A).

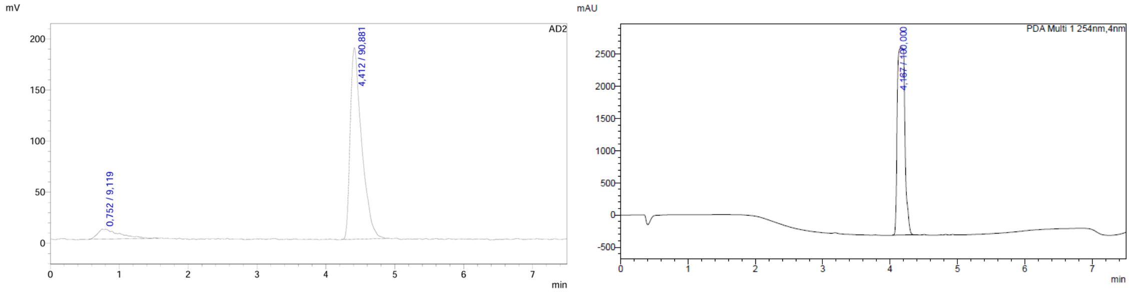

Figure S289: Radio-UHPLC chromatogram of [^{18}F]**71** prepared from **18b** (**left**); (separate run) UV UHPLC chromatogram of reference compound **71** (**right**) (Gradient A).

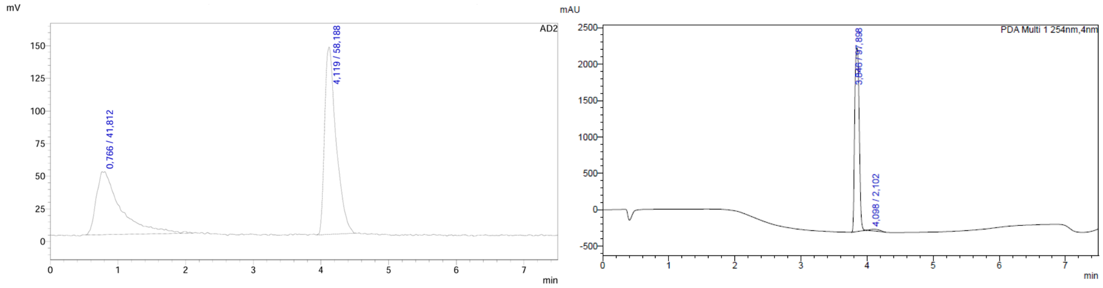

Figure S290: Radio-UHPLC chromatogram of [^{18}F**72** prepared from **19a** (**left**); (separate run) UV UHPLC chromatogram of reference compound **72** (**right**) (Gradient A).

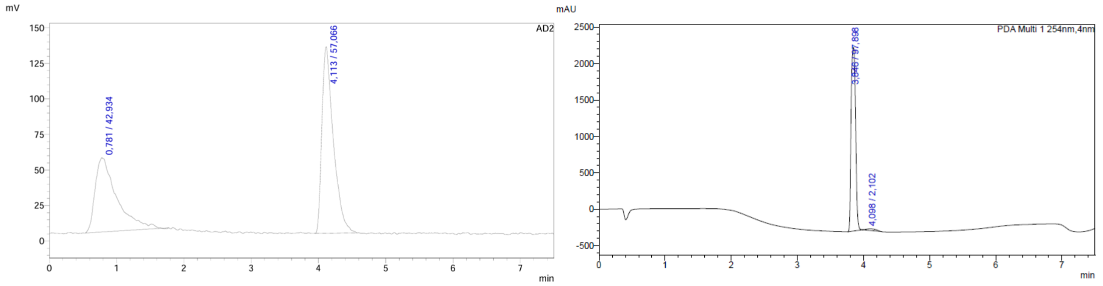

Figure S291: Radio-UHPLC chromatogram of [^{18}F]**72** prepared from **19b** (**left**); (separate run) UV UHPLC chromatogram of reference compound **72** (**right**) (Gradient A).

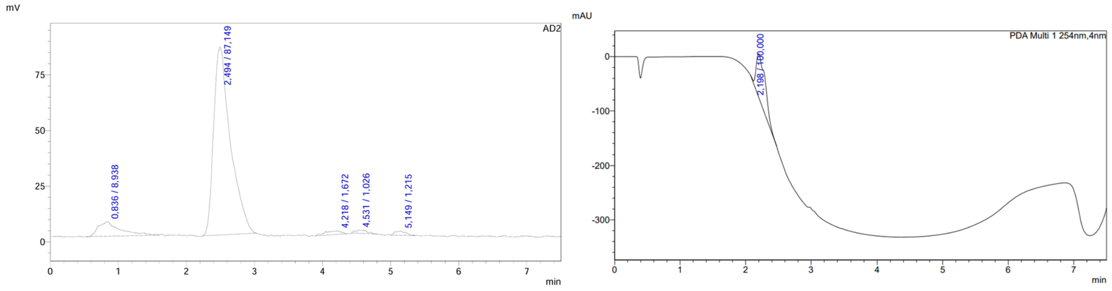

Figure S292: Radio-UHPLC chromatogram of [^{18}F]**73** prepared from **20a** (**left**); (separate run) UV UHPLC chromatogram of reference compound **73** (**right**) (Gradient A).

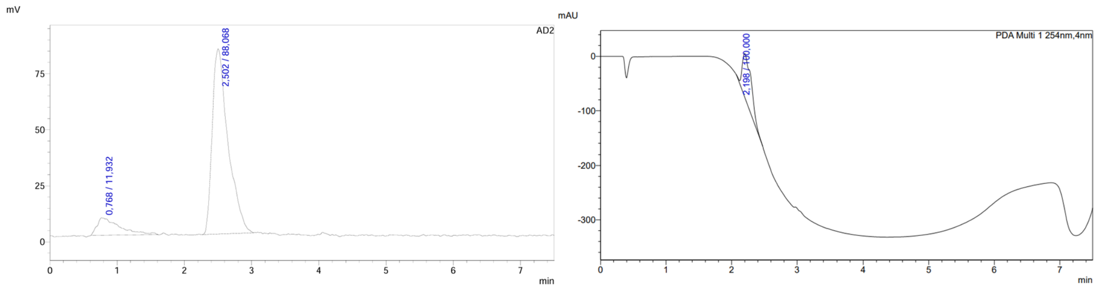

Figure S293: Radio-UHPLC chromatogram of [^{18}F]**73** prepared from **20b** (**left**); (separate run) UV UHPLC chromatogram of reference compound **73** (**right**) (Gradient A).

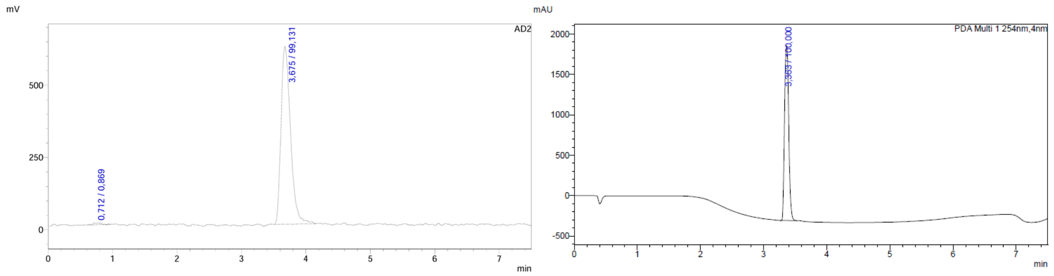

Figure S294: Radio-UHPLC chromatogram of [^{18}F]**74** prepared from **21a** (**left**); (separate run) UV UHPLC chromatogram of reference compound **74** (**right**) (Gradient A).

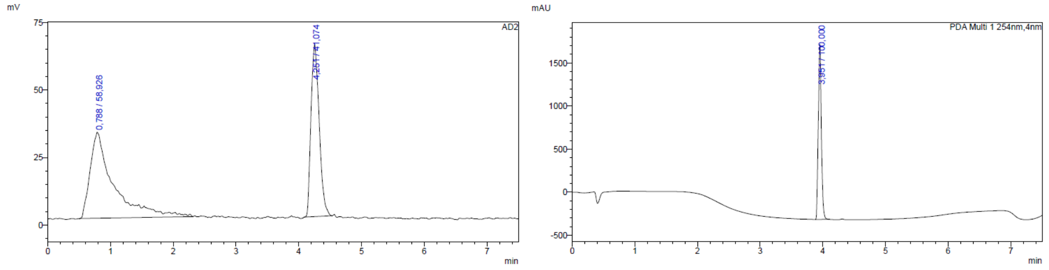

Figure S295: Radio-UHPLC chromatogram of [^{18}F]**75** prepared from **22a** (**left**); (separate run) UV UHPLC chromatogram of reference compound **75** (**right**) (Gradient A).

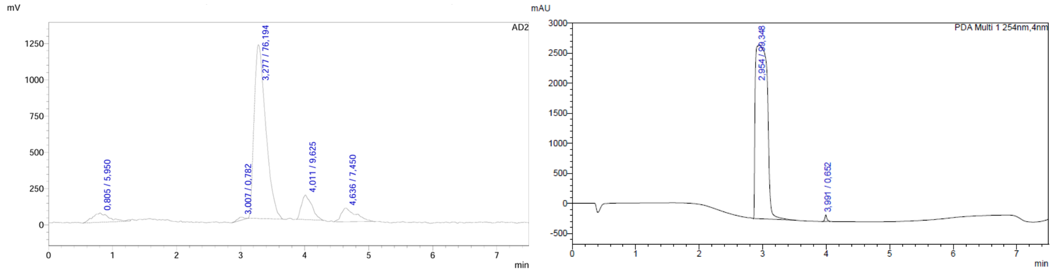

Figure S296: Radio-UHPLC chromatogram of [^{18}F]**76** prepared from **23a** (**left**); (separate run) UV UHPLC chromatogram of reference compound **76** (**right**) (Gradient A).

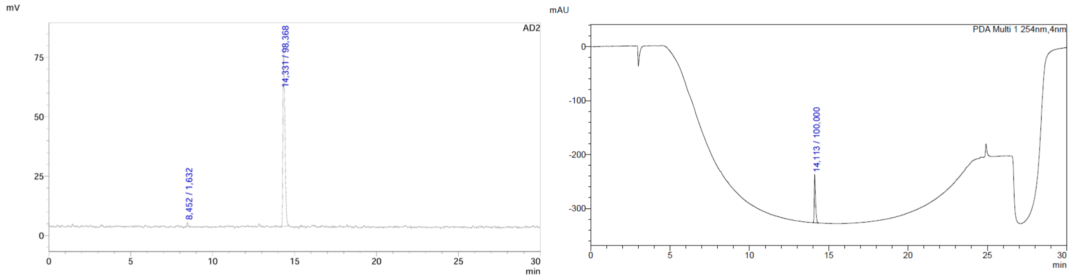

Figure S297: Radio-HPLC chromatogram of [^{18}F]**78** (**left**); (separate run) UV HPLC chromatogram of reference compound **78** (**right**) (Gradient C).

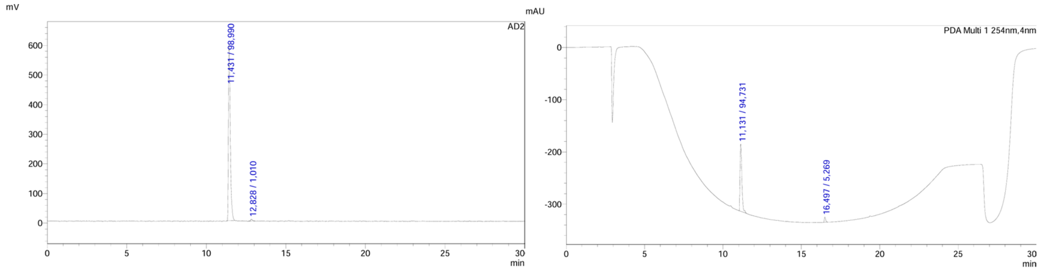

Figure S298: Radio-HPLC chromatogram of [^{18}F]**80** (**left**); (separate run) UV HPLC chromatogram of reference compound **80** (**right**) (Gradient C).

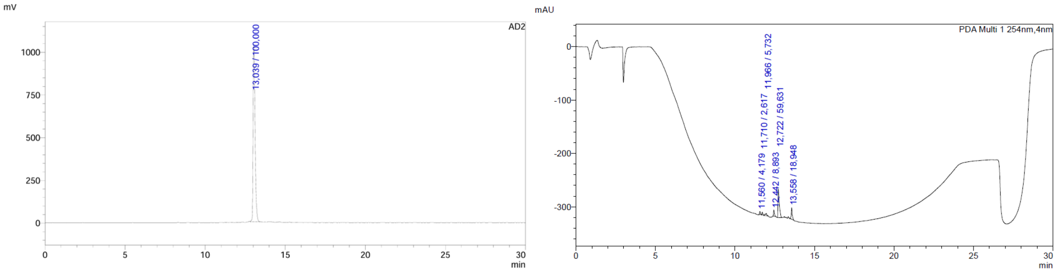

Figure S299: Radio-HPLC chromatogram of [18F]**82** (**left**); (separate run) UV HPLC chromatogram of reference compound **82** (**right**) (Gradient C).

Determination of Molar Activity

ID# : 1
Name : 4-OBn-Ar-F
Quantitative Method : External Standard
Function : f(x)=58,0314*x-232,689
Rr1=0,9996393 Rr2=0,9992788 RSS=1,146914e+004
MeanRF: 4,946090e+001 RFSD: 7,344299e+000 RFRSD: 14,848695
FitType : Linear
ZeroThrough : Not Through
Weighted Regression : None
Detector Name : PDA

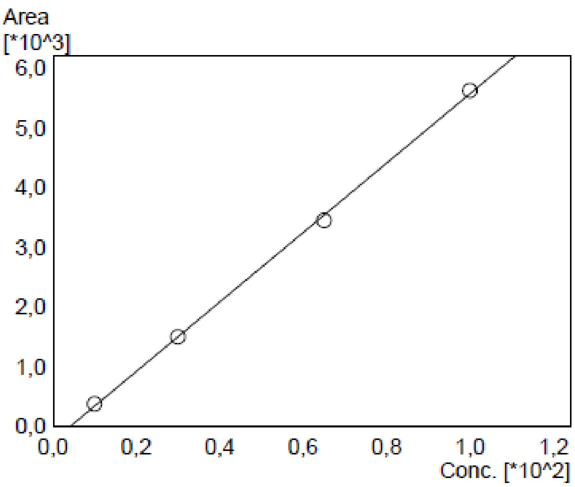

#	Conc.(Ratio)	MeanArea	Area
7	10	383	366
			400
8	30	1506	1533
			1479
9	65	3453	3474
			3433
10	100	5624	5605
			5642

Figure S300: Calibration curve for molar activity determination of [18F]**67**.

Calculation:

A small aliquot of 22.35 MBq of [18F]**67** in MeCN/H_2O (200 µL) was obtained following semi-preparative HPLC purification. Injection of 20 µL (2235 KBq) of the [18F]**67** sample for UHPLC analysis was performed in triplicate using an optimized gradient used to construct the calibration curve (Fig. S300). The mean peak area of the corresponding non-radioactive reference compound **67** observed in the UV channel was plotted along the calibration curve and the concentration of **67** was found to be 1363.67 ± 38.07 pmol (on column). Accordingly, the A_m value for [18F]**67** was determined to be 1.64 ± 0.04 GBq/µmol, from a starting activity of 3.090 GBq with a radiolabeling precursor concentration of 11.5 µmol (4.22 mg of **67**).

References

(1) Kreller, M.; Pietzsch, H.; Walther, M.; Tietze, H.; Kaever, P.; Knieß, T.; Füchtner, F.; Steinbach, J.; Preusche, S. Introduction of the New Center for Radiopharmaceutical Cancer Research at Helmholtz-Zentrum Dresden-Rossendorf. *Instruments* **2019**, *3* (1), 9. https://doi.org/10.3390/instruments3010009.

(2) Klootwyk, B. M.; Ryan, A. E.; Lopez, A.; McCloskey, M. J. R.; Janosko, C. P.; Deiters, A.; Floreancig, P. E. Peroxide-Mediated Release of Organophosphates from Boron-Containing Phosphotriesters: A New Class of Organophosphate Prodrugs. *Org. Lett.* **2023**, *25* (29), 5530–5535. https://doi.org/10.1021/acs.orglett.3c02036.

(3) Oka, N.; Yamada, T.; Sajiki, H.; Akai, S.; Ikawa, T. Aryl Boronic Esters Are Stable on Silica Gel and Reactive under Suzuki–Miyaura Coupling Conditions. *Org. Lett.* **2022**, *24* (19), 3510–3514. https://doi.org/10.1021/acs.orglett.2c01174.

(4) Janaagal, A.; Kushwaha, A.; Jhaldiyal, P.; Dhilip Kumar, T. J.; Gupta, I. Photoredox Catalysis by 21-Thiaporphyrins: A Green and Efficient Approach for C−N Borylation and C−H Arylation. *Chemistry A European J* **2024**, *30* (46). https://doi.org/10.1002/chem.202401623.

(5) Bryce, M. R.; Wang, C.; Batsanov, A. S.; Sage, I. An Improved Synthesis and Structural Characterisation of 2-(4-Acetylthiophenylethynyl)-4-Nitro-5-Phenylethynylaniline: The Molecule Showing High Negative Differential Resistance (NDR). *Synthesis* **2003**, No. 13, 2089–2095. https://doi.org/10.1055/s-2003-41451.

(6) Ma, G.; McDaniel, J. W.; Murphy, J. M. One-Step Synthesis of [^{18} F]Fluoro-4-(Vinylsulfonyl)Benzene: A Thiol Reactive Synthon for Selective Radiofluorination of Peptides. *Org. Lett.* **2021**, *23* (2), 530–534. https://doi.org/10.1021/acs.orglett.0c04054.

(7) Jiang, S.; Ge, Y.; Zhang, A.; Hu, S.; Jin, T.; Chen, J.; Zhang, H.; Qin, Y.; Ma, X.; Tang, J.; Liu, J. Cuprate Ion-Pair Catalyzed Conjugate Borylation of Vinyl Sulfones in a Biphasic System. *Org. Biomol. Chem.* **2025**, *23* (14), 3330–3335. https://doi.org/10.1039/d5ob00242g.

(8) Wang, X.; Xie, G.; Zhao, Y.; Zheng, K.; Fang, Y.; Wang, X. Facile Pinacol Coupling of Aliphatic Ketones by Brook Rearrangement in the Presence of Samarium Species. *Tetrahedron Letters* **2021**, *72*, 153069. https://doi.org/10.1016/j.tetlet.2021.153069.
